# Associations of continuous glucose monitoring-assessed glucose variability with intima-media thickness and ultrasonic tissue characteristics of the carotid arteries: a cross-sectional analysis in patients with type 2 diabetes

**DOI:** 10.1186/s12933-021-01288-5

**Published:** 2021-05-04

**Authors:** Naohiro Taya, Naoto Katakami, Tomoya Mita, Yosuke Okada, Satomi Wakasugi, Hidenori Yoshii, Toshihiko Shiraiwa, Akihito Otsuka, Yutaka Umayahara, Kayoko Ryomoto, Masahiro Hatazaki, Tetsuyuki Yasuda, Tsunehiko Yamamoto, Masahiko Gosho, Iichiro Shimomura, Hirotaka Watada

**Affiliations:** 1 Department of Metabolic Medicine, Osaka University Graduate School of Medicine, 2-2, Yamadaoka, Suita, Osaka 565-0871 Japan; 2 Department of Metabolism & Endocrinology, Juntendo University Graduate School of Medicine, 2-1-1, Hongo, Bunkyo-ku, Tokyo, 113-8421 Japan; 3 First Department of Internal Medicine, School of Medicine, University of Occupational and Environmental Health, 1-1, Iseigaoka, Yahatanishi-ku, Kitakyushu, Fukuoka Japan; 4 Department of Medicine, Diabetology & Endocrinology, Juntendo Tokyo Koto Geriatric Medical Center, 3-3-20, Shinsuna, Koto-ku, Tokyo, Japan; 5 Shiraiwa Medical Clinic, 4-10-24, Houzenji, Kashiwara, Osaka Japan; 6 Department of Internal Medicine, Kawasaki Hospital, 3-3-1, Higashiyamacho, Hyogo-ku, Kobe, Hyogo Japan; 7 Department of Diabetes and Endocrinology, Osaka General Medical Center, 3-1-56, Bandaihigashi, Sumiyoshi-ku, Osaka-shi, Osaka, Japan; 8 Center for Diabetes Mellitus, Osaka Rosai Hospital, 1179-3, Nagasonecho, Kita-ku, Sakai, Osaka Japan; 9 Department of Internal Medicine, Japan Community Health Care Organization Osaka Hospital, 4-2-78, Fukushima, Fukushima-ku, Osaka-shi, Osaka Japan; 10 Department of Diabetes and Endocrinology, Osaka Police Hospital, 10-31, Kitayamacho, Tennoji-ku, Osaka-shi, Osaka Japan; 11 Diabetes and Endocrinology, Kansai Rosai Hospital, 3-1-69, Inabaso, Amagasaki, Hyogo Japan; 12 Department of Biostatistics, Faculty of Medicine, University of Tsukuba, 1-1-1, Tennodai, Tsukuba, Ibaraki Japan

**Keywords:** Type 2 diabetes, Glucose variability, Continuous glucose monitoring, Intima-media thickness, Gray-scale median, Tissue characteristics

## Abstract

**Background:**

The association between glucose variability and the progression of atherosclerosis is not completely understood. We aimed to evaluate the associations of glucose variability with the progression of atherosclerosis in the early stages.

**Methods:**

We conducted a cross-sectional analysis to investigate the associations of glucose variability, assessed by continuous glucose monitoring, with intima-media thickness (IMT) and gray-scale median (GSM) of the carotid arteries, which are different indicators for the progression of atherosclerosis. We used baseline data from a hospital-based multicenter prospective observational cohort study among Japanese patients with type 2 diabetes without a history of cardiovascular diseases aged between 30 and 80 years. Continuous glucose monitoring was performed by Freestyle Libre Pro, and glucose levels obtained every 15 min for a maximum of eight days were used to calculate the metrics of glucose variability. IMT and GSM were evaluated by ultrasonography, and the former indicates thickening of intima-media complex in the carotid artery wall, while the latter indicates tissue characteristics.

**Results:**

Among 600 study participants (age: 64.9 ± 9.2 (mean ± SD) years; 63.2%: men; HbA1c: 7.0 ± 0.8%), participants with a larger intra- and inter-day glucose variability had a lower GSM and most of these associations were statistically significant. No trend based on glucose variability was shown regarding IMT. Standard deviation of glucose (regression coefficient, β = − 5.822; 95% CI − 8.875 to − 2.768, *P* < 0.001), glucose coefficient of variation (β = − 0.418; − 0.685 to − 0.151, *P* = 0.002), mean amplitude of glycemic excursion (β = − 1.689; − 2.567 to − 0.811, *P* < 0.001), mean of daily differences (β = − 6.500; − 9.758 to − 3.241, *P* < 0.001), and interquartile range (β = − 4.289; − 6.964 to − 1.614, *P* = 0.002) had a statistically significant association with mean-GSM after adjustment for conventional cardiovascular risk factors, including HbA1c. No metrics of glucose variability had a statistically significant association with IMT.

**Conclusions:**

Continuous glucose monitoring-assessed glucose variability was associated with the tissue characteristics of the carotid artery wall in type 2 diabetes patients without cardiovascular diseases.

**Supplementary Information:**

The online version contains supplementary material available at 10.1186/s12933-021-01288-5.

## Introduction

Clinical studies have evaluated the associations between glucose variability, as assessed by continuous glucose monitoring (CGM), and cardiovascular disease (CVD) or atherosclerosis. A prospective observational study showed that glucose variability might be a predictor of CVD in patients after acute myocardial infarction [1]. Furthermore, cross-sectional studies have reported that glucose variability is associated with the presence and severity of coronary artery disease (CAD) in subjects at high risk of CAD [2] and coronary plaque vulnerability in patients with CAD [3–5]. These findings suggested that CGM-assessed glucose variability was associated with the progression of atherosclerosis in patients at a relatively developed stage of atherosclerosis.

Carotid ultrasonography is one of the assessment procedures for atherosclerosis and has several advantages, such as noninvasiveness, simplicity, inexpensiveness, and reproducibility. Moreover, it was reported that it could be a prognostic tool for patients with diabetes [6]. Due to these advantages, the indices of carotid ultrasonography have been used as a marker of atherosclerosis in previous clinical studies [7–15]. Intima-media thickness (IMT), a carotid ultrasound measure, increases over time along with the progression of atherosclerosis [16] and is one of the best indices for the detection of early-stage atherosclerosis in patients with diabetes [17]. On the other hand, gray-scale median (GSM) is a semi-quantitative measure of ultrasonic tissue characteristics in the carotid plaque or artery wall, and low GSM values are related to “vulnerable lesion,” such as atheroma and intra-plaque hemorrhage [18–20]. Although both GSM and IMT are indicators of the progress of atherosclerosis, it has been reported that GSM of the intima-media complex and IMT have only a modest correlation with each other [21]. In the earliest stage of atherosclerosis, lipids are deposited in the deep layer of the diffuse intimal thickening, followed by the infiltration of macrophages. Macrophages transform into foam cells, leading to pathologic intimal thickening [22]. Therefore, GSM may represent an earlier stage of atherosclerosis. We have reported that the addition of IMT and/or ultrasonic tissue characteristics of carotid lesions to traditional risk factors improves the predictive ability of future CVD [23–26]. In addition, change over time in IMT or ultrasonic tissue characteristics of carotid lesions is a prognostic factor for CVD and can be a useful surrogate marker [26].

The results of studies on the association between CGM-assessed glucose variability and IMT remain controversial [7–14]. In addition, the association between glucose variability and ultrasonic tissue characteristics in the carotid plaque or artery wall has not yet been investigated. It remains unclear as to whether glucose variability is associated with the progression of atherosclerosis in the early stages. To assess this association, the present study investigated whether CGM-assessed glucose variability is associated with IMT or the ultrasonic tissue characteristics of carotid lesions.

## Methods

### Study design

This study was a sub-analysis of a multicenter prospective observational cohort study to investigate the relationship between CGM-assessed glucose variability and the incidence of cardiovascular events among Japanese patients with type 2 diabetes without a history of CVD. We recruited 1000 patients who had a regular attendance at the outpatient clinics of 34 institutions, and the follow-up period was 5 years, as described previously [27]. We conducted a cross-sectional analysis to investigate the associations of CGM-assessed glucose variability with IMT and ultrasonic tissue characteristics of carotid lesions using baseline data from the prospective observational study.

### Study population

The population of this prospective observational study consisted of Japanese patients with type 2 diabetes. The inclusion and exclusion criteria were as described previously [27]. Briefly, patients aged between 30 and 80 years without a history of CVD, whose antidiabetic medications had not been changed until CGMs for 6 months, before written informed consent was obtained, were included.

A total of 1000 patients who met the eligibility criteria were recruited between May 2018 and March 2019. One patient withdrew their consent. Among the 999 patients, 600 patients whose carotid ultrasound images at baseline were obtained were included in this analysis. The ethics committees of each participating institution approved the study protocol, and the study was conducted in accordance with the principles of the Helsinki Declaration. Written informed consent was obtained from all the participants after providing them a complete explanation regarding the study.

### Measurement of major cardiovascular risk factors

As described previously [27], we obtained information about the duration of diabetes, smoking status (never or ever smoker), comorbidities, and medications from the medical records and/or by questioning the patients. Blood pressure, height, and weight were measured during the physical examination. Blood samples were obtained after overnight fasting, and HbA1c (National Glycohemoglobin Standardization Program), glucose, total cholesterol, HDL cholesterol, triglycerides, creatinine, and uric acid levels were obtained using standard techniques. The estimated glomerular filtration rate (eGFR) (mL/min/1.73 m^2^) was calculated according to the guidelines in the Statement of the Japanese Society of Nephrology [28]. Urine albumin-to-creatinine ratio was measured using spot urine samples.

### Metrics of CGM using the Freestyle Libre Pro

The study participants received CGM using the Freestyle Libre Pro (FLP) (Abbott Japan, Tokyo, Japan). The FLP sensors were placed for 14 days in principle, and the glucose levels obtained every 15 min from 3 to 10 days were used for the analysis. In this study, the following CGM metrics were analyzed: intra-day glucose variability was assessed by standard deviation (SD), glucose coefficient of variation (CV), and mean amplitude of glycemic excursion (MAGE) [29]. Inter-day glucose variability was assessed by the mean of daily differences (MODD) [30] and interquartile range (IQR) [31]. Mean glucose, median glucose, time in range (TIR): 3.9–10.0 mmol/L, time above range (TAR): > 10.0 mmol/L, TAR: > 13.9 mmol/L, time below range (TBR): < 3.9 mmol/L, TBR: < 3.0 mmol/L, high blood glucose index (HBGI), and low blood glucose index (LBGI) [32] were also measured. TIR, TAR, and TBR were recommended as standardized CGM metrics in the International Conference on Advanced Technologies and Treatments for Diabetes 2019 [33]. TIR: 3.9–10.0 mmol/L is the percentage of time spent in the target glucose range between 3.9 and 10.0 mmol/L. TAR: > 10.0 mmol/L and TAR: > 13.9 mmol/L are the percentages of the time above the target glucose range (above 10.0 mmol/L and 13.9 mmol/L, respectively). TBR: < 3.9 mmol/L and TBR: < 3.0 mmol/L are the percentages of the time below target glucose range (below 3.9 mmol/L and 3.0 mmol/L, respectively).

### Measurement of IMT and assessment of ultrasonic tissue characteristics of carotid lesions

Ultrasonographic scans of the carotid artery were performed by expert sonographers using a high-resolution B-mode ultrasound scanner equipped with a high-frequency (> 7.5 MHz) linear transducer, with a limit of detection of < 0.1 mm. The common carotid artery (CCA), carotid bulb, and internal carotid artery were scanned bilaterally in transverse sections, and then in longitudinal sections at different angles (anterior, lateral, and posterior, which approximately corresponded to 60°, 90°, and 150° for the right carotid artery, and 210°, 270°, and 300° for the left carotid artery marked on the Meijer’s Carotid Arc). The site of greatest thickness, including plaque lesions, was then sought along the arterial walls.

IMT is defined as the distance between two parallel echogenic lines corresponding to the vascular lumen and adventitial layer. To avoid inter-reader variability, all scans were stored electronically and emailed to the central office (IMT Evaluation Committee, Osaka, Japan) and were read in a random order by a single experienced reader blinded to the clinical characteristics of the patients, using automated digital edge-detection software (Intimascope; MediaCross, Tokyo, Japan) [34]. The software system averaged approximately 200 IMT values in the segment 2 cm proximal to the dilation of the carotid bulb in the right and left CCAs, and the mean value of the right and left CCAs was defined as mean-IMT. In addition, the greatest IMTs, including plaque lesions, in the right and left CCAs were measured, and then the maximum values of the right and left CCAs were defined as CCA-max-IMT. In this study, localized elevated lesions with a maximum thickness of > 1.0 mm, having a point of inflection on the surface of the intima-media complex, were defined as “carotid plaques.” The intra-reader CV for mean-IMT and CCA-max-IMT measurements was 2.0% and 2.7%, respectively, for 40 consecutively replicated measurements.

The ultrasonic tissue characteristics of carotid lesions were evaluated using the GSM method. The Adobe Photoshop software version 7.0 (Adobe Systems, San Jose, CA, USA) was used for image standardization and calculation of gray-scale values. The standardization of the B-mode image was performed using a curve option, so that the GSM values for the blood ranged between 0 and 5, and for the adventitia between 185 and 195 [35]. The right and left mean-IMT areas (the segment 2 cm proximal to the dilation of the carotid bulb) were then delineated using a freehand tool, and the GSM value of the selected area was read from the entire delineated area. The average values of the right and left carotid arteries were defined as mean-GSM. If atherosclerotic plaque lesions and/or thickened (focal IMT ≥ 1.0 mm) lesions were detected, the GSM of these lesions was also measured using the same method. The lowest values of these lesions were defined as thickened lesion-GSM. If multiple plaque lesions were found in the same individual, the plaque with the greatest thickness was subjected to GSM measurement separately in the left and right carotid arteries, and subsequently, the lower value, “plaque-GSM,” was used as the representative value of the participant’s plaques. To avoid inter-reader variability, all scans were read in a random order by a single reader (K. A.) who was unaware of the clinical characteristics of the patients. The intra-reader CV for the GSM measurements was 2.9%, for 40 consecutive measurements.

### Statistical analysis

If the data were normally distributed, the continuous variables were expressed as means and standard deviations, or as medians and interquartile ranges if non-normally distributed. The categorical variables were expressed as counts and percentages. *P* value < 0.05 was considered statistically significant. All analyses were performed using the SAS software (version 9.4 or; SAS Institute, Cary, NC, USA).

First, the clinical characteristics of the participants in and out of the present analysis were compared. Second, the clinical characteristics and metrics of CGM were compared between the groups based on IMT or GSM. The mean-IMT ≥ 1.0 mm was defined as IMT-thickening, and the participants were categorized by the presence or absence of IMT-thickening. The participants were also categorized based on tertiles of mean-GSM, thickened lesion-GSM, or plaque-GSM. Third, IMTs and GSMs were compared between the groups based on the tertiles of the metrics of glucose variability. For the two-level classification comparisons, unpaired *t*-tests and Chi-square tests were used for the continuous and categorical variables, respectively. For the three-level classification comparisons, a trend analysis was performed using linear and logistic regression models for the continuous and categorical outcomes, respectively.

Next, multivariable regression analyses were performed to investigate whether the metrics of CGM were associated with IMT or GSM. Model 1 was not adjusted, and Models 2–5 were adjusted for the major conventional risk factors for CVD as follows: Model 2 additionally included age and sex; Model 3 additionally included BMI and the duration of diabetes; Model 4 additionally included smoking status, HbA1c, systolic blood pressure, total cholesterol, HDL cholesterol, log-transformed triglycerides, and uric acid; and Model 5 additionally included eGFR and the log-transformed urine albumin-to-creatinine ratio.

## Results

### Clinical characteristics of the study subjects

Among the 999 study patients from the original study, 600 patients with carotid ultrasound images at baseline were included in the present analysis.

The baseline clinical characteristics of the participants in the present analysis (n = 600) are summarized in Table 1. The mean age was 64.9 ± 9.2 (mean ± SD) years, 63.2% were men, and HbA1c was 7.0 ± 0.8% (53.5 ± 9.0 mmol/mol), and the estimated duration of type 2 diabetes was 13.3 ± 8.3 years. There were statistically significant differences between those who underwent carotid ultrasonographic examinations (e.g. measurement of IMT and/or GSM) (n = 600) and those who did not (n = 399) in the following variables: duration of type 2 diabetes, eGFR, urine albumin-to-creatinine ratios, prevalence of dyslipidemia, use of anti-diabetic drugs, angiotensin-converting enzyme inhibitors or angiotensin II receptor blockers, and statins. The participants in the present study also showed lower mean glucose, median glucose, SD, CV, MAGE, TAR: > 10.0 mmol/L, TAR: > 13.9 mmol/L, HBGI, MODD, and IQR, and higher TIR: 3.9–10.0 mmol/L than those in individuals who were excluded (Additional file 1: Table S1).

**Table 1:** Clinical characteristics of all the participants

	All (n = 600)
Sex: male	379 (63.2)
Age (years)	64.9 ± 9.2
Duration of diabetes (years)	11.0 (6.0, 18.0)
Ever smoker	327 (54.5)
Hypertension	347 (57.8)
Dyslipidemia	450 (75.0)
BMI (kg/m^2^)	24.6 ± 3.8
Anti-diabetic medications	894 (89.5)
Insulin therapy	158 (15.8)
ACE-I or ARB use	412 (41.2)
Statin use	508 (51.0)
Systolic BP (mmHg)	132.0 ± 14.8
HbA1c (%)	7.0 ± 0.8
HbA1c (mmol/mol)	53.5 ± 9.0
FPG (mmol/L)	7.63 ± 1.81
AST (U/L)	22.9 ± 8.7
ALT (U/L)	22.9 ± 13.3
γ-GTP (U/L)	31.0 ± 33.6
Uric acid (μmol/L)	308 ± 73
Total cholesterol (mmol/L)	4.77 ± 0.81
HDL cholesterol (mmol/L)	1.55 ± 0.41
Triglycerides (mmol/L)	1.12 (0.80, 1.58)
LDL cholesterol (mmol/L)	2.67 ± 0.69
eGFR (mL/min/1.73 m^2^)	73.4 ± 20.6
u-Alb/Cr (mg/g)	14.4 (6.4, 44.5)
Ultrasonographic scans of the carotid artery	
Mean-IMT (mm)	0.76 ± 0.15
CCA-max-IMT (mm)	1.11 ± 0.44
Mean-GSM	48.7 ± 19.3
Thickened lesion-GSM	43.5 ± 19.5
Plaque-GSM	61.5 ± 29.9
Metrics of CGM	
Mean glucose (mmol/L)	7.62 ± 1.68
Median glucose (mmol/L)	7.25 ± 1.72
SD (mmol/L)	1.96 (1.60, 2.36)
CV (%)	25.7 (22.2, 29.4)
MAGE (mmol/L)	5.12 (4.08, 6.52)
TIR (%): 3.9–10.0 mmol/L	84.6 (70.4, 92.3)
TAR (%): > 10.0 mmol/L	12.8 (4.6, 27.9)
TAR (%): > 13.9 mmol/L	0.4 (0.0, 3.1)
TBR (%): < 3.9 mmol/L	0.1 (0.0, 2.0)
TBR (%): < 3.0 mmol/L	0.0 (0.0, 0.0)
HBGI	4.35 (2.74, 6.84)
LBGI	1.08 (0.44, 2.17)
MODD (mmol/L)	1.63 (1.29, 2.00)
IQR (mmol/L)	2.00 (1.61, 2.46)

Continuous data are presented as means ± standard deviations or medians (interquartile ranges). Categorical data are presented as counts (percentages)

*ACE-I* angiotensin-converting enzyme inhibitor, *ARB* angiotensin II receptor blocker, *BP* blood pressure, *FPG* fasting plasma glucose, *AST* aspartate aminotransferase, *ALT* alanine aminotransferase, *γ-GTP* gamma-glutamyl transpeptidase, *eGFR* estimated glomerular filtration rate, *u-Alb/Cr* urine albumin-to-creatinine ratio, *IMT* intima-media thickness, *CCA* common carotid artery, *GSM* gray-scale median, *CGM* continuous glucose monitoring, *SD* standard deviation, *CV* coefficient of variation, *MAGE* mean amplitude of glycemic excursion, *TIR* time in range, *TAR* time above range, *TBR* time below range, *HBGI* high blood glucose index, *LBGI* low blood glucose index, *MODD* mean of daily differences, *IQR* interquartile range

### Associations between the carotid measures and metrics of CGM

The average of mean-IMT of all the participants was 0.76 ± 0.15 mm and 28 out of 600 participants had IMT-thickening (mean-IMT ≥ 1.0 mm), which indicated that the IMT thickness of the participants in this study was relatively mild. Table 2 shows comparisons of clinical parameters between participants with IMT-thickening (mean-IMT ≥ 1.0 mm, n = 28) and those without IMT-thickening (mean-IMT < 1.0 mm, n = 572). Participants with IMT-thickening had higher systolic blood pressure and urine albumin-to-creatinine ratio than those in participants without IMT-thickening. However, there were no statistically significant differences between the metrics of CGM in participants with and without IMT-thickening.

**Table 2:** Comparisons of clinical parameters between participants with and without intima-media thickness-thickening

	without IMT-thickening (n = 572)	with IMT-thickening (n = 28)	*P* value
Sex: male	359 (62.8)	20 (71.4)	0.353
Age (years)	64.8 ± 9.2	67.5 ± 7.9	0.123
Duration of diabetes (years)	13.3 ± 8.3	14.2 ± 8.6	0.561
Ever smoker	309 (54.0)	18 (64.3)	0.287
BMI (kg/m^2^)	24.6 ± 3.8	24.4 ± 3.1	0.800
Systolic BP (mmHg)	131.6 ± 14.6	139.9 ± 16.6	0.004
HbA1c (%)	7.1 ± 0.8	6.8 ± 0.7	0.108
HbA1c (mmol/mol)	53.6 ± 9.0	50.8 ± 7.4	0.108
FPG (mmol/L)	7.64 ± 1.81	7.47 ± 1.72	0.618
AST (U/L)	23.0 ± 8.8	22.3 ± 7.0	0.690
ALT (U/L)	23.1 ± 13.4	18.6 ± 8.8	0.077
γ-GTP (U/L)	31.3 ± 34.2	25.4 ± 17.2	0.373
Uric acid (μmol/L)	307 ± 73	328 ± 78	0.153
Total cholesterol (mmol/L)	4.77 ± 0.81	4.67 ± 0.70	0.540
HDL cholesterol (mmol/L)	1.55 ± 0.41	1.59 ± 0.40	0.602
log triglycerides (mmol/L)	0.149 ± 0.507	0.082 ± 0.507	0.494
LDL cholesterol (mmol/L)	2.65 ± 0.68	2.64 ± 0.45	0.929
eGFR (mL/min/1.73 m^2^)	70.3 ± 18.3	64.5 ± 15.2	0.099
log u-Alb/Cr (mg/g)	2.84 ± 1.48	3.47 ± 1.70	0.033
Mean glucose (mmol/L)	7.64 ± 1.70	7.35 ± 1.13	0.383
Median glucose (mmol/L)	7.27 ± 1.74	6.93 ± 1.24	0.303
SD (mmol/L)	1.96 ± 0.60	1.92 ± 0.46	0.710
CV (%)	25.7 ± 5.9	26.4 ± 6.1	0.562
MAGE (mmol/L)	5.29 ± 1.98	5.08 ± 1.52	0.587
TIR (%): 3.9–10.0 mmol/L	80.7 ± 18.0	84.1 ± 11.6	0.316
TAR (%): > 10.0 mmol/L	17.2 ± 18.4	13.4 ± 11.6	0.286
TAR (%): > 13.9 mmol/L	3.2 ± 8.2	1.3 ± 2.2	0.219
TBR (%): < 3.9 mmol/L	2.2 ± 4.7	2.5 ± 5.8	0.740
TBR (%): < 3.0 mmol/L	0.3 ± 1.4	0.5 ± 1.8	0.583
HBGI	5.15 ± 4.33	4.50 ± 2.24	0.427
LBGI	1.58 ± 1.65	1.67 ± 1.68	0.787
MODD (mmol/L)	1.69 ± 0.62	1.61 ± 0.46	0.477
IQR (mmol/L)	2.08 ± 0.76	1.90 ± 0.50	0.205

Continuous data are presented as means ± standard deviations. Categorical data are presented as counts (percentages). Unpaired *t*-tests were used to evaluate the differences between the groups with and without intima-media thickness (IMT)-thickening for continuous variables. Chi-square tests were used for categorical variables. Mean-IMT ≥ 1.0 mm was defined as IMT-thickening

*BP* blood pressure, *FPG* fasting plasma glucose, *AST* aspartate aminotransferase, *ALT* alanine aminotransferase, *γ-GTP* gamma-glutamyl transpeptidase, *eGFR* estimated glomerular filtration rate, *u-Alb/Cr* urine albumin-to-creatinine ratio, *SD* standard deviation, *CV* coefficient of variation, *MAGE* mean amplitude of glycemic excursion, *TIR* time in range, *TAR* time above range, *TBR* time below range, *HBGI* high blood glucose index, *LBGI* low blood glucose index, *MODD* mean of daily differences, *IQR* interquartile range

Table 3 shows comparisons of clinical parameters among the mean-GSM tertiles. Participants with a lower mean-GSM were statistically significantly older and a higher percentage of them were women. Furthermore, these participants had a longer duration of diabetes and higher BMI, HbA1c, and triglyceride levels, while their HDL and total cholesterol levels were lower. With regard to the metrics of CGM, these participants had higher mean glucose, median glucose, TAR: > 10.0 mmol/L, TAR: > 13.9 mmol/L, and HBGI with lower TIR: 3.9–10.0 mmol/L. These participants also had a larger SD, and higher MAGE, MODD, and IQR, which indicated larger intra- and inter-day glucose variability. Similarly, participants with a lower thickened lesion-GSM and plaque-GSM also had larger glucose variability (Additional file 1: Tables S2 and S3).

**Table 3:** Comparisons of clinical parameters among mean-gray-scale median tertiles

	Bottom tertile (n = 203)	Middle tertile (n = 199)	Top tertile (n = 197)	*P* for trend
Sex: male	122 (60.1)	119 (59.8)	138 (70.1)	0.040
Age (years)	65.4 ± 8.6	65.8 ± 9.6	63.5 ± 9.2	0.046
Duration of diabetes (years)	14.5 ± 8.3	13.5 ± 8.5	11.9 ± 7.9	0.002
Ever smoker	101 (49.8)	110 (55.3)	116 (58.9)	0.067
BMI (kg/m^2^)	25.3 ± 3.4	24.5 ± 4.3	24.0 ± 3.6	< 0.001
Systolic BP (mmHg)	131.5 ± 13.7	132.6 ± 16.7	131.6 ± 13.8	0.915
HbA1c (%)	7.2 ± 0.9	7.0 ± 0.9	6.9 ± 0.7	0.001
HbA1c (mmol/mol)	55.1 ± 9.8	53.1 ± 9.5	52.2 ± 7.3	0.001
FPG (mmol/L)	7.83 ± 1.82	7.55 ± 1.67	7.52 ± 1.92	0.082
AST (U/L)	23.2 ± 8.4	22.2 ± 7.7	23.4 ± 9.8	0.766
ALT (U/L)	23.7 ± 12.9	21.9 ± 13	23.2 ± 13.9	0.706
γ-GTP (U/L)	30.1 ± 26.9	28.9 ± 30.9	34.1 ± 41.6	0.243
Uric acid (μmol/L)	310 ± 76	300 ± 71	315 ± 73	0.506
Total cholesterol (mmol/L)	4.66 ± 0.80	4.80 ± 0.84	4.84 ± 0.77	0.034
HDL cholesterol (mmol/L)	1.45 ± 0.36	1.60 ± 0.38	1.59 ± 0.46	< 0.001
log triglycerides (mmol/L)	0.240 ± 0.500	0.107 ± 0.491	0.088 ± 0.519	0.002
LDL cholesterol (mmol/L)	2.59 ± 0.68	2.66 ± 0.74	2.71 ± 0.58	0.078
eGFR (mL/min/1.73 m^2^)	69.4 ± 20.2	71.5 ± 16.7	69.3 ± 17.5	0.971
log u-Alb/Cr (mg/g)	2.98 ± 1.53	2.86 ± 1.47	2.76 ± 1.49	0.141
Mean glucose (mmol/L)	7.86 ± 1.77	7.69 ± 1.82	7.33 ± 1.39	0.002
Median glucose (mmol/L)	7.50 ± 1.80	7.28 ± 1.88	6.97 ± 1.40	0.002
SD (mmol/L)	2.04 ± 0.64	2.01 ± 0.56	1.84 ± 0.57	< 0.001
CV (%)	25.9 ± 6.1	26.4 ± 5.7	24.9 ± 5.6	0.096
MAGE (mmol/L)	5.53 ± 2.22	5.38 ± 1.79	4.92 ± 1.81	0.002
TIR (%): 3.9–10.0 mmol/L	77.9 ± 19.0	79.9 ± 18.4	84.7 ± 15.0	< 0.001
TAR (%): > 10.0 mmol/L	19.8 ± 19.5	17.9 ± 18.8	13.3 ± 15.3	< 0.001
TAR (%): > 13.9 mmol/L	3.9 ± 8.0	3.5 ± 9.8	2.0 ± 5.7	0.015
TBR (%): < 3.9 mmol/L	2.3 ± 4.9	2.2 ± 5.1	2.0 ± 4.1	0.614
TBR (%): < 3.0 mmol/L	0.3 ± 1.3	0.4 ± 1.9	0.2 ± 0.8	0.313
HBGI	5.67 ± 4.34	5.42 ± 4.84	4.27 ± 3.34	0.001
LBGI	1.55 ± 1.73	1.65 ± 1.83	1.56 ± 1.35	0.964
MODD (mmol/L)	1.78 ± 0.67	1.70 ± 0.58	1.57 ± 0.56	< 0.001
IQR (mmol/L)	2.16 ± 0.78	2.11 ± 0.78	1.96 ± 0.68	0.006

Continuous data are presented as means ± standard deviations. Categorical data are presented as counts (percentages). Bottom tertile: mean-gray-scale median (GSM) ≤ 38.5, middle tertile: 38.5 < mean-GSM ≤ 52.0, top tertile: 52.0 < mean-GSM

*P* for trend was calculated using linear and logistic regression models for continuous and categorical outcomes, respectively

*BP* blood pressure, *FPG* fasting plasma glucose, *AST* aspartate aminotransferase, *ALT* alanine aminotransferase, *γ-GTP* gamma-glutamyl transpeptidase, *eGFR* estimated glomerular filtration rate, *u-Alb/Cr* urine albumin-to-creatinine ratio, *SD* standard deviation, *CV* coefficient of variation, *MAGE* mean amplitude of glycemic excursion, *TIR* time in range, *TAR* time above range, *TBR* time below range, *HBGI* high blood glucose index, *LBGI* low blood glucose index, *MODD* mean of daily differences, *IQR* interquartile range

**Fig. 1:**
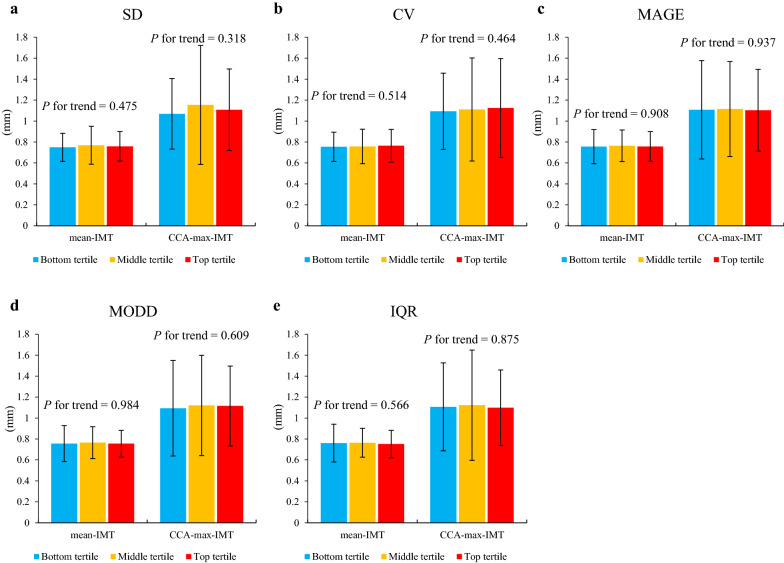
Associations between metrics of glucose variability and intima-media thickness. Mean-intima-media thickness (IMT), and CCA-max-IMT are represented according to the tertiles of **a** standard deviation, **b** coefficient of variation, **c** mean amplitude of glycemic excursion, **d** mean of daily differences, and **e** interquartile range. Data are presented as means ± standard deviations. *P* for trend was calculated using a linear regression model. *SD* standard deviation, *CV* coefficient of variation, *MAGE* mean amplitude of glycemic excursion, *MODD* mean of daily differences, *IQR* interquartile range

**Fig. 2:**
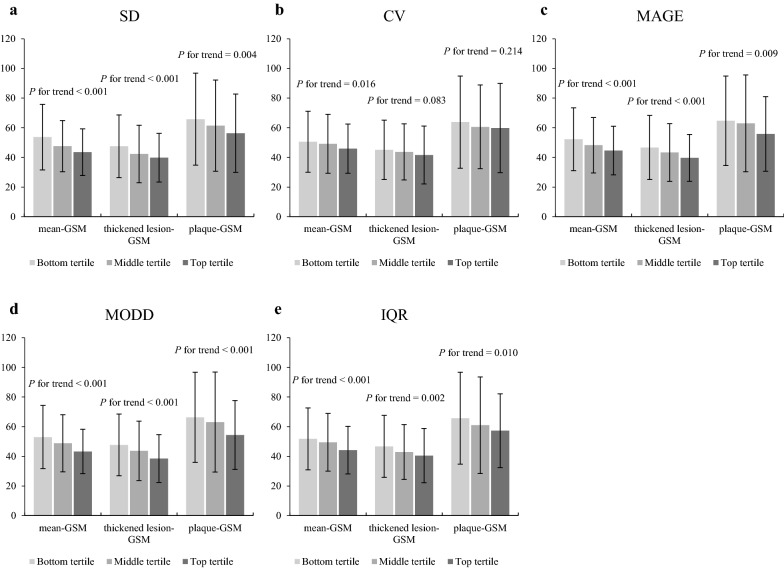
Associations between metrics of glucose variability and gray-scale median. Mean-gray-scale median (GSM), thickened lesion-GSM, and plaque-GSM are represented according to the tertiles of **a** standard deviation, **b** coefficient of variation, **c** mean amplitude of glycemic excursion, **d** mean of daily differences, and **e** interquartile range. Data are presented as means ± standard deviations. *P* for trend was calculated using a linear regression model. *SD* standard deviation, *CV* coefficient of variation, *MAGE* mean amplitude of glycemic excursion, *MODD* mean of daily differences, *IQR* interquartile range

Figures 1 and 2 show the association of the metrics of glucose variability with IMT and GSM, respectively. No trend based on glucose variability was shown regarding IMT, while the participants with larger intra- and inter-day glucose variability showed lower GSM. Most of these associations were statistically significant.

Table 4 shows the results of the linear regression analyses investigating the associations of the metrics of CGM with IMT and GSM. The metrics of CGM showed no statistically significant associations with mean-IMT and CCA-max-IMT. In contrast, mean glucose, median glucose, SD, CV, MAGE, TIR: 3.9–10.0 mmol/L, TAR: > 10.0 mmol/L, TAR: > 13.9 mmol/L, HBGI, MODD, and IQR had a crude association with mean-GSM. Among these metrics of CGM, mean glucose, SD, CV, MAGE, TIR: 3.9–10.0 mmol/L, TAR: > 10.0 mmol/L, HBGI, MODD, and IQR still had a statistically significant association after adjustment for age, sex, BMI, duration of diabetes, smoking status, HbA1c, systolic blood pressure, total cholesterol, HDL cholesterol, log-transformed triglycerides, eGFR, uric acid, and log-transformed urine albumin-to-creatinine ratio. Similar tendencies regarding the associations of the metrics of CGM with thickened lesion-GSM and plaque-GSM were also shown; however, most of them were not statistically significant after adjustment for these parameters (Additional file 1: Table S4). It was confirmed that these conventional risk factors showed no strong correlations with each other (Additional file 1: Table S5), suggesting the absence of multicollinearity.

**Table 4:** Association of the metrics of continuous glucose monitoring with intima-media thickness and gray-scale median

	mean-IMT (n = 600)		CCA-max-IMT (n = 600)		mean-GSM (n = 599)	
	β (95% CI)	*P* value	β (95% CI)	*P* value	β (95% CI)	*P* value
***Mean glucose (mmol/L)***						
Model 1	− 0.001 (− 0.008, 0.006)	0.768	0.005 (− 0.016, 0.026)	0.634	− 1.842 (− 2.750, − 0.934)	< 0.001
Model 2	− 0.002 (− 0.009, 0.005)	0.646	0.004 (− 0.016, 0.025)	0.677	− 1.950 (− 2.851, − 1.049)	< 0.001
Model 3	− 0.002 (− 0.009, 0.005)	0.654	0.002 (− 0.019, 0.023)	0.850	− 1.738 (− 2.629, − 0.846)	< 0.001
Model 4	0.004 (− 0.007, 0.015)	0.471	0.012 (− 0.021, 0.045)	0.459	− 1.987 (− 3.443, − 0.530)	0.008
Model 5	0.006 (− 0.005, 0.017)	0.302	0.016 (− 0.018, 0.050)	0.353	− 1.740 (− 3.200, − 0.280)	0.020
***Median glucose (mmol/L)***						
Model 1	− 0.001 (− 0.009, 0.006)	0.700	0.004 (− 0.017, 0.024)	0.726	− 1.722 (− 2.612, − 0.832)	< 0.001
Model 2	− 0.002 (− 0.008, 0.005)	0.619	0.003 (− 0.017, 0.023)	0.744	− 1.834 (− 2.717, − 0.951)	< 0.001
Model 3	− 0.002 (− 0.009, 0.005)	0.626	0.000 (− 0.020, 0.020)	0.982	− 1.576 (− 2.452, − 0.700)	< 0.001
Model 4	0.004 (− 0.007, 0.014)	0.528	0.007 (− 0.026, 0.039)	0.682	− 1.529 (− 2.960, − 0.098)	0.036
Model 5	0.005 (− 0.006, 0.016)	0.356	0.010 (− 0.023, 0.043)	0.558	− 1.312 (− 2.747, 0.124)	0.073
***SD (mmol/L)***						
Model 1	0.002 (− 0.018, 0.023)	0.841	0.036 (− 0.023, 0.095)	0.231	− 5.810 (− 8.353, − 3.266)	< 0.001
Model 2	− 0.008 (− 0.028, 0.011)	0.405	0.016 (− 0.042, 0.074)	0.589	− 5.460 (− 8.001, − 2.918)	< 0.001
Model 3	− 0.008 (− 0.028, 0.012)	0.416	0.019 (− 0.040, 0.078)	0.532	− 5.450 (− 7.990, − 2.909)	< 0.001
Model 4	− 0.003 (− 0.026, 0.021)	0.830	0.040 (− 0.030, 0.109)	0.265	− 6.119 (− 9.179, − 3.060)	< 0.001
Model 5	− 0.001 (− 0.025, 0.023)	0.937	0.042 (− 0.029, 0.113)	0.243	− 5.900 (− 8.952, − 2.848)	< 0.001
***CV (%)***						
Model 1	0.001 (− 0.001, 0.003)	0.392	0.005 (− 0.002, 0.011)	0.147	− 0.332 (− 0.595, − 0.070)	0.013
Model 2	0.000 (− 0.002, 0.002)	0.659	0.002 (− 0.004, 0.008)	0.557	− 0.266 (− 0.530, − 0.001)	0.049
Model 3	0.000 (− 0.003, 0.002)	0.680	0.003 (− 0.003, 0.009)	0.346	− 0.315 (− 0.580, − 0.050)	0.020
Model 4	− 0.001 (− 0.003, 0.002)	0.639	0.003 (− 0.003, 0.009)	0.306	− 0.418 (− 0.686, − 0.151)	0.002
Model 5	− 0.001 (− 0.003, 0.002)	0.632	0.003 (− 0.003, 0.009)	0.320	− 0.428 (− 0.696, − 0.161)	0.002
***MAGE (mmol/L)***						
Model 1	0.002 (− 0.004, 0.008)	0.568	0.012 (− 0.006, 0.030)	0.181	− 1.665 (− 2.442, − 0.887)	< 0.001
Model 2	− 0.001 (− 0.007, 0.005)	0.636	0.006 (− 0.012, 0.024)	0.503	− 1.605 (− 2.381, − 0.829)	< 0.001
Model 3	− 0.001 (− 0.007, 0.005)	0.652	0.008 (− 0.010, 0.025)	0.395	− 1.666 (− 2.433, − 0.899)	< 0.001
Model 4	0.000 (− 0.006, 0.007)	0.916	0.015 (− 0.005, 0.035)	0.137	− 1.741 (− 2.619, − 0.862)	< 0.001
Model 5	0.001 (− 0.006, 0.008)	0.785	0.017 (− 0.004, 0.037)	0.111	− 1.684 (− 2.562, − 0.807)	< 0.001
***TIR (%): 3.9–10.0 mmol/L***						
Model 1	− 0.001 (− 0.008, 0.006)	0.841	− 0.010 (− 0.030, 0.010)	0.343	1.963 (1.106, 2.821)	< 0.001
Model 2	0.001 (− 0.006, 0.007)	0.787	− 0.007 (− 0.026, 0.013)	0.503	1.990 (1.139, 2.840)	< 0.001
Model 3	0.001 (− 0.006, 0.007)	0.800	− 0.004 (− 0.024, 0.015)	0.668	1.774 (0.927, 2.620)	< 0.001
Model 4	− 0.003 (− 0.013, 0.006)	0.505	− 0.013 (− 0.041, 0.015)	0.357	2.269 (1.031, 3.507)	< 0.001
Model 5	− 0.004 (− 0.014, 0.006)	0.412	− 0.015 (− 0.043, 0.014)	0.318	2.187 (0.954, 3.419)	< 0.001
***TAR (%): >10.0 mmol/L***						
Model 1	0.000 (− 0.001, 0.001)	0.777	0.001 (− 0.002, 0.002)	0.647	− 0.192 (− 0.276, − 0.109)	< 0.001
Model 2	0.000 (− 0.001, 0.000)	0.560	0.000 (− 0.002, 0.002)	0.762	− 0.198 (− 0.281, − 0.115)	< 0.001
Model 3	0.000 (− 0.001, 0.001)	0.567	0.000 (− 0.002, 0.002)	0.962	− 0.177 (− 0.259, − 0.095)	< 0.001
Model 4	0.000 (− 0.001, 0.001)	0.660	0.001 (− 0.002, 0.004)	0.605	− 0.234 (− 0.363, − 0.104)	< 0.001
Model 5	0.000 (− 0.001, 0.001)	0.488	0.001 (− 0.002, 0.004)	0.514	− 0.213 (− 0.342, − 0.084)	0.001
***TAR (%): >13.9 mmol/L***						
Model 1	− 0.001 (− 0.002, 0.001)	0.275	0.000 (− 0.004, 0.004)	1.000	− 0.277 (− 0.469, − 0.085)	0.005
Model 2	− 0.001 (− 0.002, 0.001)	0.201	0.000 (− 0.004, 0.004)	0.954	− 0.293 (− 0.483, − 0.103)	0.003
Model 3	− 0.001 (− 0.002, 0.001)	0.204	0.000 (− 0.005, 0.004)	0.887	− 0.274 (− 0.460, − 0.087)	0.004
Model 4	− 0.001 (− 0.003, 0.002)	0.613	0.001 (− 0.005, 0.007)	0.857	− 0.255 (− 0.518, 0.010)	0.059
Model 5	− 0.001 (− 0.003, 0.002)	0.653	0.001 (− 0.005, 0.007)	0.809	− 0.258 (− 0.519, 0.004)	0.054
***TBR (%): <3.9 mmol/L***						
Model 1	0.002 (0.000, 0.005)	0.065	0.007 (− 0.001, 0.014)	0.071	0.073 (− 0.254, 0.401)	0.660
Model 2	0.002 (− 0.001, 0.004)	0.220	0.005 (− 0.002, 0.012)	0.175	0.122 (− 0.203, 0.448)	0.461
Model 3	0.002 (− 0.001, 0.004)	0.213	0.005 (− 0.002, 0.013)	0.156	0.130 (− 0.190, 0.449)	0.426
Model 4	0.001 (− 0.002, 0.003)	0.519	0.004 (− 0.003, 0.012)	0.260	− 0.089 (− 0.420, 0.242)	0.598
Model 5	0.001 (− 0.002, 0.003)	0.676	0.004 (− 0.004, 0.012)	0.316	− 0.167 (− 0.500, 0.165)	0.324
***TBR (%): <3.0 mmol/L***						
Model 1	0.007 (− 0.002, 0.016)	0.119	0.025 (0.000, 0.051)	0.050	− 0.651 (− 1.765, 0.464)	0.252
Model 2	0.005 (− 0.003, 0.013)	0.223	0.022 (− 0.003, 0.047)	0.087	− 0.500 (− 1.607, 0.608)	0.376
Model 3	0.005 (− 0.003, 0.014)	0.216	0.022 (− 0.003, 0.047)	0.083	− 0.438 (− 1.525, 0.650)	0.429
Model 4	0.005 (− 0.004, 0.013)	0.293	0.023 (− 0.001, 0.048)	0.064	− 0.913 (− 2.010, 0.183)	0.102
Model 5	0.004 (− 0.005, 0.012)	0.385	0.022 (− 0.003, 0.047)	0.088	− 1.014 (− 2.113, 0.086)	0.071
***HBGI***						
Model 1	− 0.001 (− 0.004, 0.002)	0.563	0.002 (− 0.006, 0.010)	0.646	− 0.772 (− 1.131, − 0.414)	< 0.001
Model 2	− 0.001 (− 0.004, 0.001)	0.306	0.001 (− 0.007, 0.009)	0.829	− 0.778 (− 1.133, − 0.423)	< 0.001
Model 3	− 0.001 (− 0.004, 0.001)	0.312	0.001 (− 0.008, 0.009)	0.903	− 0.730 (− 1.081, − 0.379)	< 0.001
Model 4	0.000 (− 0.004, 0.004)	0.937	0.005 (− 0.008, 0.017)	0.463	− 0.979 (− 1.516, − 0.442)	< 0.001
Model 5	0.000 (− 0.004, 0.004)	0.933	0.005 (− 0.007, 0.018)	0.412	− 0.946 (− 1.480, − 0.411)	< 0.001
***LBGI***						
Model 1	0.003 (− 0.005, 0.010)	0.476	0.007 (− 0.015, 0.028)	0.546	0.261 (− 0.676, 1.199)	0.585
Model 2	0.000 (− 0.007, 0.007)	0.947	0.002 (− 0.020, 0.023)	0.888	0.467 (− 0.467, 1.402)	0.327
Model 3	0.000 (− 0.007, 0.007)	0.931	0.003 (− 0.018, 0.024)	0.798	0.452 (− 0.467, 1.372)	0.335
Model 4	− 0.002 (− 0.009, 0.006)	0.648	0.001 (− 0.021, 0.024)	0.899	− 0.345 (− 1.333, 0.643)	0.493
Model 5	− 0.003 (− 0.011, 0.005)	0.454	− 0.001 (− 0.024, 0.022)	0.958	− 0.583 (− 1.580, 0.413)	0.251
***MODD (mmol/L)***						
Model 1	− 0.008 (− 0.028, 0.012)	0.420	− 0.001 (− 0.059, 0.057)	0.968	− 5.395 (− 7.887, − 2.902)	< 0.001
Model 2	− 0.007 (− 0.025, 0.012)	0.495	0.003 (− 0.054, 0.059)	0.929	− 5.610 (− 8.078, − 3.143)	< 0.001
Model 3	− 0.007 (− 0.026, 0.013)	0.508	− 0.001 (− 0.059, 0.056)	0.966	− 5.215 (− 7.702, − 2.728)	< 0.001
Model 4	0.003 (− 0.022, 0.028)	0.810	0.010 (− 0.065, 0.084)	0.800	− 6.702 (− 9.949, − 3.456)	< 0.001
Model 5	0.004 (− 0.022, 0.029)	0.766	0.009 (− 0.067, 0.085)	0.809	− 6.649 (− 9.908, − 3.389)	< 0.001
***IQR (mmol/L)***						
Model 1	− 0.012 (− 0.029, 0.004)	0.135	− 0.014 (− 0.061, 0.034)	0.571	− 3.621 (− 5.658, − 1.585)	< 0.001
Model 2	− 0.010 (− 0.025, 0.006)	0.209	− 0.008 (− 0.054, 0.038)	0.735	− 3.929 (− 5.948, − 1.910)	< 0.001
Model 3	− 0.010 (− 0.026, 0.006)	0.212	− 0.010 (− 0.057, 0.037)	0.676	− 3.676 (− 5.702, − 1.650)	< 0.001
Model 4	− 0.005 (− 0.026, 0.015)	0.607	− 0.009 (− 0.070, 0.051)	0.762	− 4.535 (− 7.207, − 1.862)	< 0.001
Model 5	− 0.004 (− 0.025, 0.017)	0.693	− 0.009 (− 0.071, 0.053)	0.778	− 4.394 (− 7.069, − 1.718)	0.001

Univariable and multivariable linear regression analysis. Model 1 was not adjusted. Model 2 was adjusted for age and sex. Model 3 was adjusted for BMI, duration of diabetes, and the covariates in Model 2. Model 4 was adjusted for smoking status, HbA1c, systolic blood pressure, total cholesterol, HDL cholesterol, log-transformed triglycerides, uric acid, and the covariates in Model 3. Model 5 was adjusted for eGFR, log-transformed urine albumin-to-creatinine ratio, and the covariates in Model 4

*IMT* intima-media thickness, *GSM* gray-scale median, *SD* standard deviation, *CV* coefficient of variation, *MAGE* mean amplitude of glycemic excursion, *TIR* time in range, *TAR* time above range, *TBR* time below range, *HBGI* high blood glucose index, *LBGI* low blood glucose index, *MODD* mean of daily differences, *IQR* interquartile range

In addition, linear regression analysis was performed among the participants in the various subgroups according to their comorbidities or medication use. The associations of the metrics of CGM with GSM were shown to be consistent across most of the subgroups, while in several subgroups, statistical significance was not shown, which may have been due to the small sample size (Additional file 1: Table S6-S29).

## Discussion

To the best of our knowledge, this is the first study to reveal that intra- and inter-day glucose variability assessed by CGM was associated with the indices of ultrasonic tissue characteristics of the carotid artery wall in patients with type 2 diabetes without CVD. Interestingly, the participants with a larger intra- and inter-day glucose variability had lower mean-GSM values that reflected histological changes related to atheromatous plaque and intra-plaque hemorrhage [18–20]. In addition, the associations remained significant after adjusting for HbA1c and other cardiovascular risk factors, which implied that glucose variability was related to the tissue characteristics of the carotid artery wall independent of the major cardiovascular risk factors, including chronic hyperglycemia.

A previous study that evaluated the association between GSM and plaque histology in 52 patients undergoing carotid endarterectomy showed that the percentages of fibrous contents in carotid plaques were positively correlated with GSM, and that the percentages of lipid and hemorrhage contents were negatively correlated [18]. Other clinical studies revealed that unstable plaque, defined as the presence of ulceration, erosion, or intra-plaque hemorrhage, had lower GSM values than stable plaques, and that low GSM values were predictive factors for unstable plaque [19, 20]. We also demonstrated that low GSM plaque was an independent predictor of future CVD events in 287 patients with type 2 diabetes without CVD, and that the addition of a low GSM plaque to the Framingham risk score and IMT improved the risk prediction for CVD events [25]. In a previous meta-analysis, we have also confirmed that plaque-GSM is a predictor of CVD [26].

The findings of this study are consistent with those of previous studies that reported the association between glucose variability and coronary plaque tissue characteristics [3–5]. Okada et al. showed that higher MAGE values were significantly correlated with increased lipid and decreased fibrous plaque contents in coronary plaques among 57 patients with acute coronary syndrome [3]. In other studies that evaluated plaque morphology among patients who underwent percutaneous coronary intervention, the percentage of necrotic core of total plaque volume, a widely used parameter of plaque vulnerability, was correlated with the metrics of glucose variability, such as MAGE, SD, MODD, and continuous overlapping net glycemic action calculated every 1 h [4, 5].

The probable underlying mechanism of glucose variability in the progression of atherosclerosis is the involvement of oxidative stress and endothelial dysfunction. Cross-sectional studies have revealed that metrics of glucose variability assessed by CGM were associated with oxidative stress [36] and endothelial dysfunction [12]. An interventional trial in patients with type 2 diabetes indicated that oscillating glucose administered by the euinsulinemic clamp technique had more deleterious effects on oxidative stress and endothelial function than constant high glucose [37]. Intermittent high glucose enhanced apoptosis related to oxidative stress in the endothelial cells through protein kinase C-dependent activation of NAD(P)H oxidase [38] and expression of adhesion molecules, such as intercellular adhesion molecule-1, vascular cell adhesion molecule-1, and E-selectin [39]. Intermittent high glucose more potently induced monocyte adhesion to the endothelial cells in rat thoracic aorta than constant high glucose [40]. Furthermore, the activity of superoxide dismutase, an antioxidant enzyme, was lower in intermittent high glucose than in constant high glucose [41].

On the other hand, in this study, we found no significant association between IMT and intra- and inter-day glucose variability, as assessed by CGM. Similarly, several previous studies reported that IMT was not associated with the metrics of glucose variability among patients with type 1 and type 2 diabetes [10, 11, 14, 42] and overweight or obese patients [12]. However, in several other studies, IMT was significantly associated with the metrics of glucose variability, such as MAGE and SD in patients with diabetes [7–9, 43]. A meta-analysis of glucose variability in type 2 diabetes showed that it was associated with IMT [44]; however, this meta-analysis included studies that assessed long-term glucose variability (SD of HbA1c and SD of FBG). Recent large cross-sectional analysis of 2215 patients with type 2 diabetes also showed that IMT was associated with SD and MAGE; however, these associations disappeared after adjusting for other clinical parameters, including HbA1c [13]. Thus, the association between IMT and glucose variability remains controversial.

The independent association of the metrics of CGM with GSM but not with IMT in this study may have been due to several reasons.

First, a change in tissue characteristics can precede IMT thickening in the early stages of atherosclerosis. In the human coronary and carotid arteries, diffuse intimal thickening (DIT) occurs from an early age before atherosclerotic lesions develop [45]. In the earliest stage of atherosclerosis, lipids are deposited in the deep layer of the DIT, followed by infiltration of macrophages. Macrophages transform into foam cells, leading to pathologic intimal thickening [22].

Second, glucose variability may be a risk factor with a stronger relationship with GSM than that with IMT. In the present study, participants with a lower mean-GSM were older and a higher percentage were men, they had a longer duration of diabetes, higher BMI, and higher HbA1c and triglyceride levels while their HDL and total cholesterol levels were lower. In contrast, participants with IMT-thickening had higher systolic blood pressure and urine albumin-to-creatinine ratio than those in participants without IMT-thickening. A previous study that investigated the factors associated with IMT and GSM showed that GSM was associated with age, BMI, and HDL cholesterol, while IMT was associated with age, sex, BMI, and systolic blood pressure [46]. Another study revealed that GSM was associated with age, BMI, LDL and HDL cholesterol, diabetes, and smoking, while IMT was associated with age, LDL cholesterol, diabetes, and blood pressure [47]. Other clinical studies showed similar results [21, 48]. Thus, the major risk factors for IMT and GSM may be different.

Third, range restrictions [49] may have affected the results. The present study included only subjects without a history of CVD, and IMT thickness was relatively mild (only 20 participants had IMT-thickening). This restriction may have weakened the association between IMT and glucose variability.

In addition, it was reported that TIR [13] and the duration of hypoglycemia [14], as assessed by CGM, were associated with IMT. In the present study, TIR was associated with GSM but not IMT, and TBR, which was the metrics similar to the duration of hypoglycemia, was not associated with both GSM and IMT. The inconsistent associations with IMT may have been due to TIR in the study participants in the present study being relatively high and TBR being low.

The participants in this study consisted of patients with type 2 diabetes without CVD. Although glucose variability is associated with tissue characteristics in coronary plaque, to the best of our knowledge, no study has investigated the association between glucose variability and tissue characteristics of plaque or artery wall among patients without CVD. This study is the first to report the association between glucose variability and tissue characteristics of the arterial wall in patients with mild atherosclerotic changes. Another strength of this study is that the sample size was relatively larger compared to previous studies that focused on this topic of research [7–14]. In addition, there were no inter-reader differences and a few intra-reader differences in the measurements of IMT and GSM, since a single experienced reader performed the measurements of IMT and GSM.

This study has some limitations.

First, although this study found an association between CGM-assessed glucose variability and tissue characteristics of the carotid artery wall, it is unclear whether this association is causal as this was a cross-sectional study. This will be investigated in an ongoing prospective observational study [27] by evaluating the association between baseline CGM-assessed glucose variability and changes in GSM.

Second, several possible confounding factors, such as insulin resistance, diet, and socioeconomic status, were not assessed. Previous studies have shown that insulin resistance is associated with glucose variability among individuals without diabetes, and the increased glucose variability may have been due to insulin resistance [50, 51]. Basic science research has shown that insulin resistance contributes to the progression of atherosclerosis due to the relative shift in signaling from the phosphatidylinositol 3-kinase to the MAPK pathway, resulting in endothelial dysfunction and vascular damage [52, 53]. Recent clinical studies have implied an association between insulin resistance and carotid atherosclerosis [54, 55]. Therefore, insulin resistance may have been involved in the association between glucose variability and atherosclerosis. In addition, it was reported that a healthy lifestyle, including a healthy diet, moderated the relationship between CVD and its risk factors [56], and that dietary interventions, such as a low carbohydrate diet [57], the consumption of low glycemic index food [58], and food order [59], reduced glucose variability. Thus, it was possible that these diets were associated with the tissue characteristics of the carotid artery wall; however, we did not have sufficient data about these diets. Similarly, socioeconomic status could affect glucose variability and atherosclerosis.

Third, not all of the patients in the prospective observational study underwent carotid ultrasonographic evaluation. There were significant differences in certain variables at baseline between those who underwent carotid ultrasonographic measurements and those who did not (Additional file 1: Table S1), which were not irrelevant to bias.

Fourth, the exclusion criteria of the present study may have limited the generalizability of our findings. Patients whose antidiabetic medication had been changed before the CGM for six months were excluded from the study. These patients tend to have a relatively large long-term glucose variability.

Fifth, the metrics of CGM were derived from the CGM data of a maximum of eight days, which may have been insufficient to assess them as being representative of each patient. Although a recent clinical study revealed that intra-day glucose variability can be assessed using two or three days of CGM with sufficient reliability [60], it is unclear how many days are required for the reliable assessment for inter-day glucose variability.

## Conclusions

In conclusion, this study showed that intra- and inter-day glucose variability was associated with the tissue characteristics of the carotid artery wall in patients with type 2 diabetes without CVD.

## Supplementary Information

**Additional file 1: Table S1.** Clinical characteristics of the participants of the multicenter prospective observational cohort study. **Table S2.** Comparisons of clinical parameters among thickened lesion-gray-scale median tertiles. **Table S3.** Comparisons of clinical parameters among plaque-gray-scale median tertiles. **Table S4.** Associations of the metrics of continuous glucose monitoring with thickened lesion-gray-scale median and plaque-gray-scale median. **Table S5.** Associations between the adjustment factors in the multivariable regression models. **Table S6.** Associations of the metrics of continuous glucose monitoring with intima-media thickness among the participants with hypertension. **Table S7.** Associations of the metrics of continuous glucose monitoring with gray-scale median among the participants with hypertension. **Table S8.** Associations of the metrics of continuous glucose monitoring with intima-media thickness among the participants without hypertension. **Table S9.** Associations of the metrics of continuous glucose monitoring with gray-scale median among the participants without hypertension. **Table S10.** Associations of the metrics of continuous glucose monitoring with intima-media thickness among the participants with dyslipidemia. **Table S11.** Associations of the metrics of continuous glucose monitoring with gray-scale median among the participants with dyslipidemia. **Table S12.** Associations of the metrics of continuous glucose monitoring with intima-media thickness among the participants without dyslipidemia. **Table S13.** Associations of the metrics of continuous glucose monitoring with gray-scale median among the participants without dyslipidemia. **Table S14.** Associations of the metrics of continuous glucose monitoring with intima-media thickness among participants using anti-diabetic medications. **Table S15.** Associations of the metrics of continuous glucose monitoring with gray-scale median among participants using anti-diabetic medications. **Table S16.** Associations of the metrics of continuous glucose monitoring with intima-media thickness among participants not using anti-diabetic medications. **Table S17.** Associations of the metrics of continuous glucose monitoring with gray-scale median among participants not using anti-diabetic medications. **Table S18.** Associations of the metrics of continuous glucose monitoring with intima-media thickness among participants using insulin therapy. **Table S19.** Associations of the metrics of continuous glucose monitoring with gray-scale median among participants using insulin therapy. **Table S20.** Associations of the metrics of continuous glucose monitoring with intima-media thickness among participants not using insulin therapy. **Table S21.** Associations of the metrics of continuous glucose monitoring with gray-scale median among participants not using insulin therapy. **Table S22.** Associations of the metrics of continuous glucose monitoring with intima-media thickness among participants using angiotensin-converting enzyme inhibitors or angiotensin II receptor blockers. **Table S23.** Associations of the metrics of continuous glucose monitoring with gray-scale median among participants using angiotensin-converting enzyme inhibitors or angiotensin II receptor blockers. **Table S24.** Associations of the metrics of continuous glucose monitoring with intima-media thickness among participants not using angiotensin-converting enzyme inhibitors or angiotensin II receptor blockers. **Table S25.** Associations of the metrics of continuous glucose monitoring with gray-scale median among participants not using angiotensin-converting enzyme inhibitors or angiotensin II receptor blockers. **Table S26.** Associations of the metrics of continuous glucose monitoring with intima-media thickness among participants using statins. **Table S27.** Associations of the metrics of continuous glucose monitoring with gray-scale median among participants using statins. **Table S28.** Associations of the metrics of continuous glucose monitoring with intima-media thickness among participants not using statins. **Table S29.** Associations of the metrics of continuous glucose monitoring with gray-scale median among participants not using statins. **Table S30.** List of sites and investigators.

## Data Availability

All data generated or analyzed during this study are not publicly available.

## Abbreviations

CGM: Continuous glucose monitoring
CVD: Cardiovascular disease
CAD: Coronary artery disease
IMT: Intima-media thickness
GSM: Gray-scale median
eGFR: Estimated glomerular filtration rate
FLP: Freestyle Libre Pro
SD: Standard deviation
CV: Coefficient of variation
MAGE: Mean amplitude of glycemic excursion
MODD: Mean of daily differences
IQR: Interquartile range
TIR: Time in range
TAR: Time above range
TBR: Time below range
HBGI: High blood glucose index
LBGI: Low blood glucose index
CCA: Common carotid artery
DIT: Diffuse intimal thickening

## References

[1] Su G, Mi SH, Li Z, Tao H, Yang HX, Zheng H (2013). Prognostic value of early in-hospital glycemic excursion in elderly patients with acute myocardial infarction. Cardiovasc Diabetol.

[2] Su G, Mi S, Tao H, Li Z, Yang H, Zheng H (2011). Association of glycemic variability and the presence and severity of coronary artery disease in patients with type 2 diabetes. Cardiovasc Diabetol.

[3] Okada K, Hibi K, Gohbara M, Kataoka S, Takano K, Akiyama E (2015). Association between blood glucose variability and coronary plaque instability in patients with acute coronary syndromes. Cardiovasc Diabetol.

[4] Kuroda M, Shinke T, Sakaguchi K, Otake H, Takaya T, Hirota Y (2015). Effect of daily glucose fluctuation on coronary plaque vulnerability in patients pre-treated with lipid-lowering therapy: a prospective observational study. JACC Cardiovasc Interv.

[5] Otowa-Suematsu N, Sakaguchi K, Komada H, Nakamura T, Sou A, Hirota Y (2017). Comparison of the relationship between multiple parameters of glycemic variability and coronary plaque vulnerability assessed by virtual histology—intravascular ultrasound. J Diabetes Investig.

[6] Hoke M, Schillinger M, Minar E, Goliasch G, Binder CJ, Mayer FJ (2019). Carotid ultrasound investigation as a prognostic tool for patients with diabetes mellitus. Cardiovasc Diabetol.

[7] Mo Y, Zhou J, Li M, Wang Y, Bao Y, Ma X (2013). Glycemic variability is associated with subclinical atherosclerosis in Chinese type 2 diabetic patients. Cardiovasc Diabetol.

[8] Chen XM, Zhang Y, Shen XP, Huang Q, Ma H, Huang YL (2010). Correlation between glucose fluctuations and carotid intima-media thickness in type 2 diabetes. Diabetes Res Clin Pract.

[9] Zhang X, Xu X, Jiao X, Wu J, Zhou S, Lv X (2013). The effects of glucose fluctuation on the severity of coronary artery disease in type 2 diabetes mellitus. J Diabetes Res.

[10] Cesana F, Giannattasio C, Nava S, Soriano F, Brambilla G, Baroni M (2013). Impact of blood glucose variability on carotid artery intima media thickness and distensibility in type 1 diabetes mellitus. Blood Press.

[11] Di Flaviani A, Picconi F, Di Stefano P, Giordani I, Malandrucco I, Maggio P (2011). Impact of glycemic and blood pressure variability on surrogate measures of cardiovascular outcomes in type 2 diabetic patients. Diabetes Care.

[12] Buscemi S, Re A, Batsis JA, Arnone M, Mattina A, Cerasola G (2010). Glycaemic variability using continuous glucose monitoring and endothelial function in the metabolic syndrome and in Type 2 diabetes. Diabet Med.

[13] Lu J, Ma X, Shen Y, Wu Q, Wang R, Zhang L (2020). Time in range is associated with carotid intima-media thickness in Type 2 diabetes. Diabetes Technol Ther.

[14] Magri CJ, Mintoff D, Camilleri L, Xuereb RG, Galea J, Fava S (2018). Relationship of hyperglycaemia, hypoglycaemia, and glucose variability to atherosclerotic disease in type 2 diabetes. J Diabetes Res.

[15] Xu R, Zhang T, Wan Y, Fan Z, Gao X (2019). Prospective study of hemoglobin A1c and incident carotid artery plaque in Chinese adults without diabetes. Cardiovasc Diabetol.

[16] Pignoli P, Tremoli E, Poli A, Oreste P, Paoletti R (1986). Intimal plus medial thickness of the arterial wall: a direct measurement with ultrasound imaging. Circulation.

[17] Katakami N, Kaneto H, Shimomura I (2014). Carotid ultrasonography: a potent tool for better clinical practice in diagnosis of atherosclerosis in diabetic patients. J Diabetes Investig.

[18] El-Barghouty NM, Levine T, Ladva S, Flanagan A, Nicolaides A (1996). Histological verification of computerised carotid plaque characterisation. Eur J Vasc Endovasc Surg.

[19] Ruiz-Ares G, Fuentes B, Martínez-Sánchez P, Martínez-Martínez M, Díez-Tejedor E (2011). Utility of the assessment of echogenicity in the identification of symptomatic carotid artery atheroma plaques in ischemic stroke patients. Cerebrovasc Dis.

[20] Ruiz-Ares G, Fuentes B, Martínez-Sánchez P, Díez-Tejedor E (2014). A prediction model for unstable carotid atheromatous plaque in acute ischemic stroke patients: proposal and internal validation. Ultrasound Med Biol.

[21] Andersson J, Sundström J, Gustavsson T, Hulthe J, Elmgren A, Zilmer K (2009). Echogenecity of the carotid intima–media complex is related to cardiovascular risk factors, dyslipidemia, oxidative stress and inflammation. The Prospective Investigation of the Vasculature in Uppsala Seniors (PIVUS) study. Atherosclerosis.

[22] Nakashima Y, Wight TN, Sueishi K (2008). Early atherosclerosis in humans: role of diffuse intimal thickening and extracellular matrix proteoglycans. Cardiovasc Res.

[23] Yoshida M, Mita T, Yamamoto R, Shimizu T, Ikeda F, Ohmura C (2012). Combination of the Framingham risk score and carotid intima-media thickness improves the prediction of cardiovascular events in patients with type 2 diabetes. Diabetes Care.

[24] Katakami N, Takahara M, Kaneto H, Sakamoto K, Yoshiuchi K, Irie Y (2012). Ultrasonic tissue characterization of carotid plaque improves the prediction of cardiovascular events in diabetic patients: a pilot study. Diabetes Care.

[25] Irie Y, Katakami N, Kaneto H, Takahara M, Nishio M, Kasami R (2013). The utility of ultrasonic tissue characterization of carotid plaque in the prediction of cardiovascular events in diabetic patients. Atherosclerosis.

[26] Katakami N, Mita T, Gosho M, Takahara M, Irie Y, Yasuda T (2018). Clinical utility of carotid ultrasonography in the prediction of cardiovascular events in patients with diabetes: a combined analysis of data obtained in five longitudinal studies. J Atheroscler Thromb.

[27] Mita T, Katakami N, Okada Y, Yoshii H, Osonoi T, Nishida K (2019). Protocol of a prospective observational study on the relationship between glucose fluctuation and cardiovascular events in patients with Type 2 diabetes. Diabetes Ther.

[28] Matsuo S, Imai E, Horio M, Yasuda Y, Tomita K, Nitta K (2009). Revised equations for estimated GFR from serum creatinine in Japan. Am J Kidney Dis.

[29] Service FJ, Molnar GD, Rosevear JW, Ackerman E, Gatewood LC, Taylor WF (1970). Mean amplitude of glycemic excursions, a measure of diabetic instability. Diabetes.

[30] Molnar GD, Taylor WF, Ho MM (1972). Day-to-day variation of continuously monitored glycaemia: a further measure of diabetic instability. Diabetologia.

[31] Rodbard D (2009). Interpretation of continuous glucose monitoring data: glycemic variability and quality of glycemic control. Diabetes Technol Ther.

[32] Kovatchev BP, Cox DJ, Gonder-Frederick LA, Clarke W (1997). Symmetrization of the blood glucose measurement scale and its applications. Diabetes Care.

[33] Battelino T, Danne T, Bergenstal RM, Amiel SA, Beck R, Biester T (2019). Clinical targets for continuous glucose monitoring data interpretation: recommendations from the international consensus on time in range. Diabetes Care.

[34] Yanase T, Nasu S, Mukuta Y, Shimizu Y, Nishihara T, Okabe T (2006). Evaluation of a new carotid intima-media thickness measurement by B-Mode ultrasonography using an innovative measurement software. Intimascope Am J Hypertens.

[35] Sabetai MM, Tegos TJ, Nicolaides AN, Dhanjil S, Pare GJ, Stevens JM (2000). Reproducibility of computer-quantified carotid plaque echogenicity: can we overcome the subjectivity?. Stroke.

[36] Monnier L, Mas E, Ginet C, Michel F, Villon L, Cristol JP (2006). Activation of oxidative stress by acute glucose fluctuations compared with sustained chronic hyperglycemia in patients with type 2 diabetes. JAMA.

[37] Ceriello A, Esposito K, Piconi L, Ihnat MA, Thorpe JE, Testa R (2008). Oscillating glucose is more deleterious to endothelial function and oxidative stress than mean glucose in normal and type 2 diabetic patients. Diabetes.

[38] Quagliaro L, Piconi L, Assaloni R, Martinelli L, Motz E, Ceriello A (2003). Intermittent high glucose enhances apoptosis related to oxidative stress in human umbilical vein endothelial cells: the role of protein kinase C and NAD(P)H-oxidase activation. Diabetes.

[39] Quagliaro L, Piconi L, Assaloni R, Da Ros R, Maier A, Zuodar G (2005). Intermittent high glucose enhances ICAM-1, VCAM-1 and E-selectin expression in human umbilical vein endothelial cells in culture: the distinct role of protein kinase C and mitochondrial superoxide production. Atherosclerosis.

[40] Azuma K, Kawamori R, Toyofuku Y, Kitahara Y, Sato F, Shimizu T (2006). Repetitive fluctuations in blood glucose enhance monocyte adhesion to the endothelium of rat thoracic aorta. Arterioscler Thromb Vasc Biol.

[41] Ihnat MA, Kaltreider RC, Thorpe JE, Green DE, Kamat CD, Leeper M (2007). Attenuated superoxide dismutase induction in retinal cells in response to intermittent high versus continuous high glucose. American J of Biochem Biotechnol.

[42] Foreman YD, Brouwers MCGJ, Berendschot TTJM, van Dongen MCJM, Eussen SJPM, van Greevenbroek MMJ (2019). The oral glucose tolerance test-derived incremental glucose peak is associated with greater arterial stiffness and maladaptive arterial remodeling: the Maastricht Study. Cardiovasc Diabetol.

[43] Liu M, Ao L, Hu X, Ma J, Bao K, Gu Y (2019). Influence of blood glucose fluctuation, C-peptide level and conventional risk factors on carotid artery intima-media thickness in Chinese Han patients with type 2 diabetes mellitus. Eur J Med Res.

[44] Liang S, Yin H, Wei C, Xie L, He H, Liu X (2017). Glucose variability for cardiovascular risk factors in type 2 diabetes: a meta-analysis. J Diabetes Metab Disord.

[45] Nakashima Y, Chen YX, Kinukawa N, Sueishi K (2002). Distributions of diffuse intimal thickening in human arteries: preferential expression in atherosclerosis-prone arteries from an early age. Virchows Arch.

[46] Peters SA, Lind L, Palmer MK, Grobbee DE, Crouse JR, O'Leary DH (2012). Increased age, high body mass index and low HDL-C levels are related to an echolucent carotid intima-media: the METEOR study. J Intern Med.

[47] Jung M, Parrinello CM, Xue X, Mack WJ, Anastos K, Lazar JM (2015). Echolucency of the carotid artery intima-media complex and intima-media thickness have different cardiovascular risk factor relationships: the Women's Interagency HIV Study. J Am Heart Assoc.

[48] Geovanini GR, Pinheiro de Sousa I, Teixeira SK, Francisco Neto MJ, Gómez Gómez LM, Del Guerra GC (2020). Carotid intima-media thickness and metabolic syndrome in a rural population: results from the Baependi Heart Study. Int J Cardiol Hypertens.

[49] Bland JM, Altman DG (2011). Correlation in restricted ranges of data. BMJ.

[50] Dimova R, Chakarova N, Grozeva G, Kirilov G, Tankova T (2019). The relationship between glucose variability and insulin sensitivity and oxidative stress in subjects with prediabetes. Diabetes Res Clin Pract.

[51] Kaya A, Koçyiğit C, Çatlı G, Özkan EB, Dündar BN (2017). The relationship between glycemic variability and inflammatory markers in obese children with insulin resistance and metabolic syndrome. J Clin Res Pediatr Endocrinol.

[52] Zeng G, Nystrom FH, Ravichandran LV, Cong LN, Kirby M, Mostowski H (2000). Roles for insulin receptor, PI3-kinase, and Akt in insulin-signaling pathways related to production of nitric oxide in human vascular endothelial cells. Circulation.

[53] King GL, Park K, Li Q (2016). Selective insulin resistance and the development of cardiovascular diseases in diabetes: the 2015 Edwin Bierman Award Lecture. Diabetes.

[54] Randrianarisoa E, Lehn-Stefan A, Hieronimus A, Wagner R, Maucher J, Rittig K (2020). Reduced insulin clearance is linked to subclinical atherosclerosis in individuals at risk for type 2 diabetes mellitus. Sci Rep.

[55] Yoon MK, Kang JG, Lee SJ, Ihm SH, Huh KB, Kim CS (2020). Relationships between thigh and waist circumference, hemoglobin glycation index, and carotid plaque in patients with type 2 diabetes. Endocrinol Metab (Seoul).

[56] Lima TR, González-Chica DA, Moreno YMF, Silva DAS (2020). Healthy lifestyle moderates the relationship between cardiovascular disease with blood pressure, body composition, carotid intima-media thickness, and glycated hemoglobin among adults. Appl Physiol Nutr Metab.

[57] Ranjan A, Schmidt S, Damm-Frydenberg C, Holst JJ, Madsbad S, Nørgaard K (2017). Short-term effects of a low carbohydrate diet on glycaemic variables and cardiovascular risk markers in patients with type 1 diabetes: a randomized open-label crossover trial. Diabetes Obes Metab.

[58] Henry CJ, Kaur B, Quek RYC, Camps SG (2017). a low glycaemic index diet incorporating isomaltulose is associated with lower glycaemic response and variability, and promotes fat oxidation in Asians. Nutrients.

[59] Tricò D, Filice E, Trifirò S, Natali A (2016). Manipulating the sequence of food ingestion improves glycemic control in type 2 diabetic patients under free-living conditions. Nutr Diabetes.

[60] Foreman YD, Brouwers MCGJ, van der Kallen CJH, Pagen DME, van Greevenbroek MMJ, Henry RMA (2020). Glucose variability assessed with continuous glucose monitoring: reliability, reference values, and correlations with established glycemic indices-The Maastricht Study. Diabetes Technol Ther.

