# Supplementary material for: Associations of continuous glucose monitoring-assessed glucose variability with intima-media thickness and ultrasonic tissue characteristics of the carotid arteries: a cross-sectional analysis in patients with type 2 diabetes
Source: Cardiovasc Diabetol. 2021 May 4;20:95. doi: 10.1186/s12933-021-01288-5 (PMC8097791; doi:10.1186/s12933-021-01288-5)
Supplement: Supplementary file 1 — Additional file 1: Table S1. Clinical characteristics of the participants of the multicenter prospective observational cohort study. Table S2. Comparisons of clinical parameters among thickened lesion-gray-scale median tertiles. Table S3. Comparisons of clinical parameters among plaque-gray-scale median tertiles. Table S4. Associations of the metrics of continuous glucose monitoring with thickened lesion-gray-scale median and plaque-gray-scale median. Table S5. Associations between the adjustment factors in the multivariable regression models. Table S6. Associations of the metrics of continuous glucose monitoring with intima-media thickness among the participants with hypertension. Table S7. Associations of the metrics of continuous glucose monitoring with gray-scale median among the participants with hypertension. Table S8. Associations of the metrics of continuous glucose monitoring with intima-media thickness among the participants without hypertension. Table S9. Associations of the metrics of continuous glucose monitoring with gray-scale median among the participants without hypertension. Table S10. Associations of the metrics of continuous glucose monitoring with intima-media thickness among the participants with dyslipidemia. Table S11. Associations of the metrics of continuous glucose monitoring with gray-scale median among the participants with dyslipidemia. Table S12. Associations of the metrics of continuous glucose monitoring with intima-media thickness among the participants without dyslipidemia. Table S13. Associations of the metrics of continuous glucose monitoring with gray-scale median among the participants without dyslipidemia. Table S14. Associations of the metrics of continuous glucose monitoring with intima-media thickness among participants using anti-diabetic medications. Table S15. Associations of the metrics of continuous glucose monitoring with gray-scale median among participants using anti-diabetic medications. Table S16. Associations of [file 12933_2021_1288_MOESM1_ESM.doc]

**Associations of Continuous Glucose Monitoring-assessed Glucose Variability with Intima-Media Thickness and Ultrasonic Tissue Characteristics of the Carotid Arteries: A Cross-Sectional Analysis in Patients with Type 2 Diabetes.**

**Additional tables**

**Table S1 Clinical characteristics of the participants in the multicenter prospective observational cohort study**

|  | Participants  (n = 999) | Patients with  IMT or GSM data  (n = 600) | Patients without  IMT and GSM data  (n = 399) | *P* value |
| --- | --- | --- | --- | --- |
| Sex: male | 608 (60.9) | 379 (63.2) | 229 (57.4) | 0.067 |
| Age (years) | 64.6 ± 9.6 | 64.9 ± 9.2 | 64.0 ± 10.3 | 0.144 |
| Duration of diabetes (years) | 11.0 (6.0, 18.0) | 12.0 (7.0, 19.0) | 10.0 (5.0, 18.0) | 0.005 |
| Ever smoker | 537 (53.8) | 327 (54.5) | 210 (52.8) | 0.590 |
| Hypertension | 553 (55.4) | 347 (57.8) | 206 (51.6) | 0.053 |
| Dyslipidemia | 717 (71.8) | 450 (75.0) | 267 (66.9) | 0.005 |
| Anti-diabetic medications | 894 (89.5) | 550 (91.7) | 344 (86.2) | 0.006 |
| Insulin therapy | 158 (15.8) | 97 (16.2) | 61 (15.3) | 0.709 |
| ACE-I or ARB use | 412 (41.2) | 266 (44.3) | 146 (36.6) | 0.015 |
| Statin use | 508 (51.0) | 320 (53.5) | 188 (47.1) | 0.048 |
| BMI (kg/m^2^) | 24.6 ± 3.9 | 24.6 ± 3.8 | 24.6 ± 4.0 | 0.836 |
| Systolic BP (mmHg) | 131.2 ± 14.8 | 132.0 ± 14.8 | 130.1 ± 14.9 | 0.051 |
| HbA1c (%) | 7.1 ± 0.8 | 7.0 ± 0.8 | 7.1 ± 0.8 | 0.366 |
| HbA1c (mmol/mol) | 53.7 ± 8.8 | 53.5 ± 9.0 | 54.0 ± 8.6 | 0.366 |
| FPG (mmol/L) | 7.60 ± 1.81 | 7.63 ± 1.81 | 7.55 ± 1.81 | 0.467 |
| AST (U/L) | 23.3 ± 9.1 | 22.9 ± 8.7 | 23.9 ± 9.7 | 0.085 |
| ALT (U/L) | 23.4 ± 13.2 | 22.9 ± 13.3 | 24.2 ± 13.0 | 0.126 |
| γ-GTP (U/L) | 32.9 ± 34.6 | 31.0 ± 33.6 | 35.7 ± 35.8 | 0.034 |
| Uric acid (μmol/L) | 307 ± 73 | 308 ± 73 | 306 ± 72 | 0.602 |
| Total cholesterol (mmol/L) | 4.81 ± 0.82 | 4.77 ± 0.81 | 4.86 ± 0.83 | 0.070 |
| HDL cholesterol (mmol/L) | 1.56 ± 0.41 | 1.55 ± 0.41 | 1.58 ± 0.40 | 0.180 |
| Triglycerides (mmol/L) | 1.12 (0.80, 1.58) | 1.11 (0.81, 1.56) | 1.13 (0.78, 1.69) | 0.901 |
| LDL cholesterol (mmol/L) | 2.67 ± 0.69 | 2.65 ± 0.67 | 2.69 ± 0.71 | 0.344 |
| eGFR (mL/min/1.73 m^2^) | 73.4 ± 20.6 | 70.0 ± 18.2 | 78.5 ± 22.9 | <0.001 |
| u-Alb/Cr (mg/g) | 14.4 (6.4, 44.5) | 13.8 (6.0, 41.1) | 15.3 (7.0, 50.0) | 0.037 |
| Ultrasonographic scans of the carotid artery | | | | |
| mean-IMT (mm) | 0.76 ± 0.15 | 0.76 ± 0.15 | - | - |
| CCA-max-IMT (mm) | 1.11 ± 0.44 | 1.11 ± 0.44 | - | - |
| mean-GSM | 48.7 ± 19.3 | 48.7 ± 19.3 | - | - |
| thickened lesion-GSM | 43.5 ± 19.5 | 43.5 ± 19.5 | - | - |
| plaque-GSM | 61.5 ± 29.9 | 61.5 ± 29.9 | - | - |
| Metrics of CGM | | | | |
| Mean glucose (mmol/L) | 7.80 ± 1.79 | 7.62 ± 1.68 | 8.06 ± 1.92 | <0.001 |
| Median glucose (mmol/L) | 7.41 ± 1.84 | 7.25 ± 1.72 | 7.66 ± 1.98 | <0.001 |
| SD (mmol/L) | 1.96 (1.60, 2.36) | 1.90 (1.54, 2.32) | 2.04 (1.70, 2.44) | <0.001 |
| CV (%) | 25.7 (22.2, 29.4) | 25.1 (21.8, 29.0) | 26.4 (23.0, 30.0) | 0.001 |
| MAGE (mmol/L) | 5.12 (4.08, 6.52) | 5.04 (3.86, 6.33) | 5.35 (4.33, 6.81) | <0.001 |
| TIR (%): 3.9–10.0 mmol/L | 84.6 (70.4, 92.3) | 85.8 (73.9, 93.6) | 82.2 (67.6, 90.5) | <0.001 |
| TAR (%): >10.0 mmol/L | 12.8 (4.6, 27.9) | 11.5 (3.7, 23.4) | 15.8 (6.4, 31.4) | <0.001 |
| TAR (%): >13.9 mmol/L | 0.4 (0.0, 3.1) | 0.3 (0.0, 2.1) | 0.8 (0.0, 4.2) | <0.001 |
| TBR (%): <3.9 mmol/L | 0.1 (0.0, 2.0) | 0.3 (0.0, 2.1) | 0.1 (0.0, 1.8) | 0.583 |
| TBR (%): <3.0 mmol/L | 0.0 (0.0, 0.0) | 0.0 (0.0, 0.0) | 0.0 (0.0, 0.0) | 0.594 |
| HBGI | 4.35 (2.74, 6.84) | 4.08 (2.55, 6.07) | 4.96 (3.20, 7.44) | <0.001 |
| LBGI | 1.08 (0.44, 2.17) | 1.11 (0.50, 2.17) | 1.03 (0.37, 2.17) | 0.164 |
| MODD (mmol/L) | 1.63 (1.29, 2.00) | 1.57 (1.24, 1.97) | 1.72 (1.37, 2.03) | 0.001 |
| IQR (mmol/L) | 2.00 (1.61, 2.46) | 1.92 (1.58, 2.40) | 2.07 (1.70, 2.52) | 0.001 |

Continuous data are presented as means ± standard deviations or medians (interquartile ranges). Categorical data are presented as counts (percentages). For the continuous data unpaired *t*-tests or Wilcoxon rank sum tests were used to evaluate the differences between the participants included in and excluded from the present analyses. Chi-square tests were used for the categorical data.

ACE-I, angiotensin-converting enzyme inhibitor; ARB, angiotensin II receptor blocker; BP, blood pressure; FPG, fasting plasma glucose; AST, aspartate aminotransferase; ALT, alanine aminotransferase; γ-GTP, gamma-glutamyl transpeptidase; eGFR, estimated glomerular filtration rate; u-Alb/Cr, urine albumin-to-creatinine ratio; IMT, intima-media thickness; CCA, common carotid artery; GSM, gray-scale median; CGM, continuous glucose monitoring; SD, standard deviation; CV, coefficient of variation; MAGE, mean amplitude of glycemic excursion; TIR, time in range; TAR, time above range; TBR, time below range; HBGI, high blood glucose index; LBGI, low blood glucose index; MODD, mean of daily differences; IQR, interquartile range

**Table S2 Comparisons of clinical parameters among thickened lesion-gray-scale median tertiles**

|  | Bottom tertile  (n = 186) | Middle tertile  (n = 194) | Top tertile  (n = 186) | *P* for trend |
| --- | --- | --- | --- | --- |
| Sex: male | 132 (71.0) | 123 (63.4) | 106 (57.0) | 0.005 |
| Age (years) | 66.9 ± 8.3 | 65.3 ± 9.3 | 63.7 ± 9.2 | <0.001 |
| Duration of diabetes (years) | 14.7 ± 8.6 | 13.4 ± 8.1 | 12.3 ± 7.9 | 0.006 |
| Ever smoker | 100 (53.8) | 106 (54.6) | 99 (53.2) | 0.917 |
| BMI (kg/m^2^) | 24.3 ± 3.2 | 24.8 ± 4.0 | 24.5 ± 4.0 | 0.661 |
| Systolic BP (mmHg) | 132.2 ± 14.4 | 131 ± 15.5 | 132.4 ± 14.2 | 0.883 |
| HbA1c (%) | 7.2 ± 0.9 | 7.0 ± 0.8 | 6.9 ± 0.8 | <0.001 |
| HbA1c (mmol/mol) | 55.2 ± 9.8 | 53.3 ± 8.6 | 52.1 ± 8.4 | <0.001 |
| FPG (mmol/L) | 7.83 ± 1.68 | 7.63 ± 1.74 | 7.46 ± 2.06 | 0.047 |
| AST (U/L) | 22.5 ± 8.7 | 23.5 ± 8.4 | 23.0 ± 9.1 | 0.597 |
| ALT (U/L) | 22.3 ± 12.9 | 23.6 ± 14.6 | 22.0 ± 10.6 | 0.865 |
| γ-GTP (U/L) | 33.1 ± 38.7 | 28.9 ± 25.8 | 31.2 ± 37.2 | 0.601 |
| Uric acid (μmol/L) | 310 ± 72 | 314 ± 71 | 302 ± 76 | 0.293 |
| Total cholesterol (mmol/L) | 4.68 ± 0.85 | 4.73 ± 0.76 | 4.86 ± 0.78 | 0.028 |
| HDL cholesterol (mmol/L) | 1.50 ± 0.39 | 1.53 ± 0.37 | 1.63 ± 0.45 | 0.003 |
| log triglycerides (mmol/L) | 0.167 ± 0.465 | 0.154 ± 0.524 | 0.103 ± 0.527 | 0.225 |
| LDL cholesterol (mmol/L) | 2.59 ± 0.70 | 2.64 ± 0.66 | 2.69 ± 0.64 | 0.124 |
| eGFR (mL/min/1.73 m^2^) | 69.1 ± 18.2 | 69.9 ± 19.9 | 69.7 ± 16.0 | 0.730 |
| log u-Alb/Cr (mg/g) | 3.03 ± 1.51 | 2.95 ± 1.59 | 2.61 ± 1.39 | 0.008 |
| Mean glucose (mmol/L) | 7.94 ± 1.82 | 7.65 ± 1.57 | 7.34 ± 1.64 | <0.001 |
| Median glucose (mmol/L) | 7.56 ± 1.87 | 7.26 ± 1.59 | 6.99 ± 1.68 | 0.001 |
| SD (mmol/L) | 2.09 ± 0.65 | 1.98 ± 0.56 | 1.82 ± 0.58 | <0.001 |
| CV (%) | 26.4 ± 6.0 | 25.9 ± 5.5 | 24.9 ± 6.0 | 0.013 |
| MAGE (mmol/L) | 5.65 ± 2.16 | 5.40 ± 1.97 | 4.82 ± 1.72 | <0.001 |
| TIR (%): 3.9–10.0 mmol/L | 77.3 ± 19.3 | 80.2 ± 17.2 | 84.7 ± 16.3 | <0.001 |
| TAR (%): >10.0 mmol/L | 20.5 ± 19.9 | 17.7 ± 17.7 | 13.2 ± 16.3 | <0.001 |
| TAR (%): >13.9 mmol/L | 4.4 ± 9.8 | 3.0 ± 6.0 | 2.2 ± 8.2 | 0.009 |
| TBR (%): <3.9 mmol/L | 2.2 ± 5.4 | 2.1 ± 4.1 | 2.1 ± 4.7 | 0.903 |
| TBR (%): <3.0 mmol/L | 0.4 ± 1.6 | 0.2 ± 1.0 | 0.3 ± 1.6 | 0.865 |
| HBGI | 5.93 ± 4.77 | 5.18 ± 3.56 | 4.36 ± 4.45 | <0.001 |
| LBGI | 1.51 ± 1.67 | 1.55 ± 1.49 | 1.65 ± 1.79 | 0.406 |
| MODD (mmol/L) | 1.79 ± 0.66 | 1.71 ± 0.61 | 1.57 ± 0.56 | <0.001 |
| IQR (mmol/L) | 2.18 ± 0.80 | 2.10 ± 0.73 | 1.96 ± 0.74 | 0.006 |

Continuous variables are presented as means ± standard deviations. Categorical variables are presented as counts (percentages). Bottom tertile: thickened lesion-gray-scale median (GSM) ≤33.0, middle tertile: 33.0 <thickened lesion-GSM ≤50.0, top tertile: 50.0 <thickened lesion-GSM.

*P* for trend was calculated using linear and logistic regression models for continuous and categorical outcomes, respectively.

BP, blood pressure; FPG, fasting plasma glucose; AST, aspartate aminotransferase; ALT, alanine aminotransferase; γ-GTP, gamma-glutamyl transpeptidase; eGFR, estimated glomerular filtration rate; u-Alb/Cr, urine albumin-to-creatinine ratio; SD, standard deviation; CV, coefficient of variation; MAGE, mean amplitude of glycemic excursion; TIR, time in range; TAR, time above range; TBR, time below range; HBGI, high blood glucose index; LBGI, low blood glucose index; MODD, mean of daily differences; IQR, interquartile range

**Table S3 Comparisons of clinical parameters among plaque-gray-scale median tertiles**

|  | Bottom tertile  (n = 163) | Middle tertile  (n = 158) | Top tertile  (n = 169) | *P* for trend |
| --- | --- | --- | --- | --- |
| Sex: male | 113 (69.3) | 106 (67.1) | 92 (54.4) | 0.005 |
| Age (years) | 66.6 ± 8.6 | 64.4 ± 9.8 | 65.5 ± 8.2 | 0.268 |
| Duration of diabetes (years) | 14.9 ± 8.9 | 13.1 ± 8.0 | 12.6 ± 8.3 | 0.015 |
| Ever smoker | 95 (58.3) | 94 (59.5) | 83 (49.1) | 0.091 |
| BMI (kg/m^2^) | 24.5 ± 3.6 | 24.9 ± 4.0 | 24.1 ± 3.7 | 0.341 |
| Systolic BP (mmHg) | 131.5 ± 15.6 | 131.4 ± 15.3 | 134.1 ± 14.4 | 0.127 |
| HbA1c (%) | 7.2 ± 0.9 | 7.0 ± 0.8 | 6.9 ± 0.8 | 0.001 |
| HbA1c (mmol/mol) | 55.2 ± 10.0 | 53.2 ± 8.3 | 52.0 ± 8.9 | 0.001 |
| FPG (mmol/L) | 7.85 ± 1.81 | 7.81 ± 2.10 | 7.21 ± 1.64 | 0.001 |
| AST (U/L) | 22.7 ± 9.2 | 23.0 ± 7.4 | 23.6 ± 9.9 | 0.395 |
| ALT (U/L) | 22.5 ± 14.9 | 23.1 ± 12.9 | 21.8 ± 10.9 | 0.646 |
| γ-GTP (U/L) | 32.0 ± 36.7 | 32.6 ± 33.3 | 28.6 ± 35.1 | 0.366 |
| Uric acid (μmol/L) | 309 ± 75 | 314 ± 73 | 301 ± 68 | 0.290 |
| Total cholesterol (mmol/L) | 4.69 ± 0.89 | 4.79 ± 0.74 | 4.76 ± 0.80 | 0.484 |
| HDL cholesterol (mmol/L) | 1.54 ± 0.39 | 1.55 ± 0.45 | 1.59 ± 0.40 | 0.216 |
| log triglycerides (mmol/L) | 0.098 ± 0.431 | 0.189 ± 0.591 | 0.107 ± 0.479 | 0.891 |
| LDL cholesterol (mmol/L) | 2.61 ± 0.75 | 2.62 ± 0.60 | 2.65 ± 0.67 | 0.631 |
| eGFR (mL/min/1.73 m^2^) | 69.5 ± 19.1 | 68.9 ± 17.9 | 69.6 ± 14.6 | 0.969 |
| log u-Alb/Cr (mg/g) | 3.03 ± 1.66 | 2.94 ± 1.56 | 2.74 ± 1.33 | 0.091 |
| Mean glucose (mmol/L) | 7.92 ± 1.75 | 7.69 ± 1.65 | 7.36 ± 1.74 | 0.003 |
| Median glucose (mmol/L) | 7.55 ± 1.83 | 7.30 ± 1.67 | 7.02 ± 1.77 | 0.006 |
| SD (mmol/L) | 2.07 ± 0.59 | 1.96 ± 0.57 | 1.89 ± 0.63 | 0.005 |
| CV (%) | 26.3 ± 5.5 | 25.6 ± 6.0 | 25.6 ± 6.1 | 0.269 |
| MAGE (mmol/L) | 5.55 ± 1.98 | 5.36 ± 2.06 | 4.95 ± 1.77 | 0.005 |
| TIR (%): 3.9–10.0 mmol/L | 78.4 ± 18.1 | 79.9 ± 18.1 | 83.1 ± 17.6 | 0.016 |
| TAR (%): >10.0 mmol/L | 19.8 ± 18.8 | 18.0 ± 18.6 | 14.3 ± 17.6 | 0.007 |
| TAR (%): >13.9 mmol/L | 4.1 ± 9.6 | 3.0 ± 7.1 | 2.7 ± 8.5 | 0.135 |
| TBR (%): <3.9 mmol/L | 1.9 ± 4.1 | 2.1 ± 5.0 | 2.6 ± 5.5 | 0.168 |
| TBR (%): <3.0 mmol/L | 0.2 ± 1.1 | 0.3 ± 1.3 | 0.4 ± 1.8 | 0.329 |
| HBGI | 5.79 ± 4.60 | 5.19 ± 3.88 | 4.66 ± 4.72 | 0.021 |
| LBGI | 1.44 ± 1.43 | 1.53 ± 1.68 | 1.79 ± 1.91 | 0.060 |
| MODD (mmol/L) | 1.81 ± 0.68 | 1.69 ± 0.58 | 1.58 ± 0.57 | <0.001 |
| IQR (mmol/L) | 2.20 ± 0.82 | 2.10 ± 0.72 | 1.94 ± 0.73 | 0.002 |

Continuous variables are presented as means ± standard deviations. Categorical variables are presented as counts (percentages). Bottom tertile: plaque-gray-scale median (GSM) ≤46.0, middle tertile: 46.0 <plaque-GSM ≤67.0, top tertile: 67.0 <plaque-GSM.

*P* for trend was calculated using linear and logistic regression models for continuous and categorical outcomes, respectively.

BP, blood pressure; FPG, fasting plasma glucose; AST, aspartate aminotransferase; ALT, alanine aminotransferase; γ-GTP, gamma-glutamyl transpeptidase; eGFR, estimated glomerular filtration rate; u-Alb/Cr, urine albumin-to-creatinine ratio; SD, standard deviation; CV, coefficient of variation; MAGE, mean amplitude of glycemic excursion; TIR, time in range; TAR, time above range; TBR, time below range; HBGI, high blood glucose index; LBGI, low blood glucose index; MODD, mean of daily differences; IQR, interquartile range

**Table S4 Associations of the metrics of continuous glucose monitoring with thickened lesion-gray-scale median and plaque-gray-scale median**

|  | thickened lesion-GSM (n = 566) | |  | plaque-GSM (n = 490) | |
| --- | --- | --- | --- | --- | --- |
|  | β (95% CI) | *P* value |  | β (95% CI) | *P* value |
| Mean glucose (mmol/L) | | | | | |
| Model 1 | -1.793 (-2.734, -0.852) | <0.001 |  | -2.850 (-4.368, -1.332) | <0.001 |
| Model 2 | -1.753 (-2.681, -0.824) | <0.001 |  | -2.775 (-4.293, -1.257) | <0.001 |
| Model 3 | -1.666 (-2.602, -0.729) | <0.001 |  | -2.617 (-4.141, -1.094) | <0.001 |
| Model 4 | -1.193 (-2.744, 0.358) | 0.131 |  | -2.605 (-5.245, 0.034) | 0.053 |
| Model 5 | -1.044 (-2.601, 0.513) | 0.188 |  | -2.370 (-5.024, 0.283) | 0.080 |
| Median glucose (mmol/L) | | | | | |
| Model 1 | -1.689 (-2.613, -0.765) | <0.001 |  | -2.650 (-4.135, -1.165) | <0.001 |
| Model 2 | -1.658 (-2.570, -0.746) | <0.001 |  | -2.577 (-4.061, -1.093) | <0.001 |
| Model 3 | -1.566 (-2.487, -0.644) | <0.001 |  | -2.399 (-3.892, -0.905) | 0.002 |
| Model 4 | -0.900 (-2.424, 0.625) | 0.247 |  | -1.962 (-4.537, 0.613) | 0.135 |
| Model 5 | -0.787 (-2.318, 0.743) | 0.313 |  | -1.766 (-4.354, 0.821) | 0.180 |
| SD (mmol/L) | | | | | |
| Model 1 | -5.421 (-8.049, -2.794) | <0.001 |  | -7.353 (-11.709, -2.998) | <0.001 |
| Model 2 | -4.859 (-7.462, -2.256) | <0.001 |  | -7.191 (-11.553, -2.829) | 0.001 |
| Model 3 | -4.669 (-7.331, -2.007) | <0.001 |  | -6.875 (-11.319, -2.430) | 0.003 |
| Model 4 | -3.799 (-7.071, -0.528) | 0.023 |  | -4.886 (-10.401, 0.629) | 0.082 |
| Model 5 | -3.461 (-6.728, -0.193) | 0.038 |  | -4.771 (-10.293, 0.751) | 0.090 |
| CV (%) | | | | | |
| Model 1 | -0.268 (-0.543, 0.006) | 0.055 |  | -0.223 (-0.677, 0.230) | 0.334 |
| Model 2 | -0.194 (-0.468, 0.080) | 0.165 |  | -0.209 (-0.667, 0.248) | 0.369 |
| Model 3 | -0.177 (-0.458, 0.103) | 0.215 |  | -0.187 (-0.655, 0.282) | 0.434 |
| Model 4 | -0.215 (-0.502, 0.072) | 0.142 |  | -0.179 (-0.662, 0.304) | 0.466 |
| Model 5 | -0.196 (-0.483, 0.091) | 0.180 |  | -0.190 (-0.674, 0.295) | 0.442 |
| MAGE (mmol/L) | | | | | |
| Model 1 | -1.513 (-2.317, -0.710) | <0.001 |  | -1.761 (-3.113, -0.409) | 0.011 |
| Model 2 | -1.326 (-2.122, -0.530) | 0.001 |  | -1.650 (-3.004, -0.297) | 0.017 |
| Model 3 | -1.285 (-2.090, -0.481) | 0.002 |  | -1.602 (-2.967, -0.238) | 0.021 |
| Model 4 | -1.021 (-1.963, -0.078) | 0.034 |  | -0.875 (-2.476, 0.726) | 0.283 |
| Model 5 | -0.903 (-1.845, 0.040) | 0.060 |  | -0.825 (-2.431, 0.781) | 0.313 |
| TIR (%): 3.9–10.0 mmol/L | | | | | |
| Model 1 | 1.920 (1.029, 2.810) | <0.001 |  | 2.079 (0.615, 3.542) | 0.005 |
| Model 2 | 1.800 (0.921, 2.679) | <0.001 |  | 1.988 (0.527, 3.450) | 0.008 |
| Model 3 | 1.709 (0.818, 2.600) | <0.001 |  | 1.793 (0.319, 3.267) | 0.017 |
| Model 4 | 1.596 (0.270, 2.922) | 0.018 |  | 0.697 (-1.520, 2.914) | 0.537 |
| Model 5 | 1.436 (0.113, 2.758) | 0.033 |  | 0.537 (-1.678, 2.751) | 0.634 |
| TAR (%): >10.0 mmol/L | | | | | |
| Model 1 | -0.188 (-0.275, -0.101) | <0.001 |  | -0.250 (-0.392, -0.108) | <0.001 |
| Model 2 | -0.181 (-0.267, -0.095) | <0.001 |  | -0.243 (-0.385, -0.101) | <0.001 |
| Model 3 | -0.173 (-0.259, -0.086) | <0.001 |  | -0.227 (-0.370, -0.084) | 0.002 |
| Model 4 | -0.169 (-0.308, -0.031) | 0.017 |  | -0.190 (-0.425, 0.044) | 0.112 |
| Model 5 | -0.152 (-0.291, -0.014) | 0.031 |  | -0.170 (-0.404, 0.064) | 0.155 |
| TAR (%): >13.9 mmol/L | | | | | |
| Model 1 | -0.276 (-0.472, -0.079) | 0.006 |  | -0.396 (-0.707, -0.085) | 0.013 |
| Model 2 | -0.269 (-0.462, -0.075) | 0.007 |  | -0.384 (-0.695, -0.074) | 0.015 |
| Model 3 | -0.259 (-0.453, -0.065) | 0.009 |  | -0.373 (-0.683, -0.063) | 0.018 |
| Model 4 | -0.124 (-0.402, 0.155) | 0.383 |  | -0.097 (-0.550, 0.355) | 0.673 |
| Model 5 | -0.114 (-0.390, 0.163) | 0.419 |  | -0.080 (-0.530, 0.370) | 0.727 |
| TBR (%): <3.9 mmol/L | | | | | |
| Model 1 | 0.063 (-0.278, 0.404) | 0.716 |  | 0.740 (0.201, 1.279) | 0.007 |
| Model 2 | 0.128 (-0.209, 0.465) | 0.455 |  | 0.764 (0.225, 1.303) | 0.006 |
| Model 3 | 0.148 (-0.190, 0.485) | 0.391 |  | 0.809 (0.270, 1.348) | 0.003 |
| Model 4 | -0.030 (-0.383, 0.323) | 0.868 |  | 0.667 (0.096, 1.238) | 0.022 |
| Model 5 | -0.027 (-0.382, 0.328) | 0.881 |  | 0.677 (0.097, 1.257) | 0.022 |
| TBR (%): <3.0 mmol/L | | | | | |
| Model 1 | 0.061 (-1.076, 1.199) | 0.916 |  | 0.768 (-1.064, 2.599) | 0.411 |
| Model 2 | 0.161 (-0.960, 1.282) | 0.778 |  | 0.757 (-1.075, 2.589) | 0.417 |
| Model 3 | 0.225 (-0.899, 1.348) | 0.695 |  | 0.925 (-0.910, 2.759) | 0.322 |
| Model 4 | -0.169 (-1.316, 0.979) | 0.773 |  | 0.523 (-1.365, 2.411) | 0.587 |
| Model 5 | -0.102 (-1.254, 1.051) | 0.863 |  | 0.498 (-1.408, 2.404) | 0.608 |
| HBGI | | | | | |
| Model 1 | -0.709 (-1.078, -0.339) | <0.001 |  | -0.994 (-1.586, -0.402) | 0.001 |
| Model 2 | -0.670 (-1.034, -0.307) | <0.001 |  | -0.966 (-1.557, -0.376) | 0.001 |
| Model 3 | -0.641 (-1.007, -0.274) | <0.001 |  | -0.919 (-1.512, -0.327) | 0.002 |
| Model 4 | -0.531 (-1.104, 0.043) | 0.070 |  | -0.659 (-1.609, 0.291) | 0.174 |
| Model 5 | -0.475 (-1.047, 0.097) | 0.103 |  | -0.598 (-1.548, 0.351) | 0.216 |
| LBGI | | | | | |
| Model 1 | 0.527 (-0.450, 1.504) | 0.290 |  | 1.889 (0.328, 3.450) | 0.018 |
| Model 2 | 0.680 (-0.289, 1.648) | 0.168 |  | 1.884 (0.319, 3.450) | 0.018 |
| Model 3 | 0.738 (-0.234, 1.711) | 0.136 |  | 2.030 (0.458, 3.602) | 0.011 |
| Model 4 | 0.128 (-0.930, 1.186) | 0.812 |  | 1.670 (-0.069, 3.408) | 0.060 |
| Model 5 | 0.160 (-0.908, 1.227) | 0.769 |  | 1.665 (-0.105, 3.434) | 0.065 |
| MODD (mmol/L) | | | | | |
| Model 1 | -5.106 (-7.680, -2.532) | <0.001 |  | -7.518 (-11.757, -3.279) | <0.001 |
| Model 2 | -5.218 (-7.747, -2.689) | <0.001 |  | -7.636 (-11.864, -3.407) | <0.001 |
| Model 3 | -4.983 (-7.581, -2.385) | <0.001 |  | -7.120 (-11.448, -2.792) | 0.001 |
| Model 4 | -4.098 (-7.570, -0.625) | 0.021 |  | -5.501 (-11.440, 0.439) | 0.069 |
| Model 5 | -3.663 (-7.155, -0.170) | 0.040 |  | -5.366 (-11.342, 0.611) | 0.078 |
| IQR (mmol/L) | | | | | |
| Model 1 | -3.359 (-5.460, -1.257) | 0.002 |  | -5.597 (-9.043, -2.150) | 0.002 |
| Model 2 | -3.535 (-5.602, -1.467) | <0.001 |  | -5.713 (-9.158, -2.268) | 0.001 |
| Model 3 | -3.326 (-5.444, -1.208) | 0.002 |  | -5.301 (-8.819, -1.784) | 0.003 |
| Model 4 | -2.195 (-5.042, 0.653) | 0.131 |  | -3.666 (-8.547, 1.214) | 0.141 |
| Model 5 | -1.881 (-4.734, 0.973) | 0.196 |  | -3.550 (-8.451, 1.351) | 0.155 |

Univariable and multivariable linear regression analysis. Model 1 was not adjusted. Model 2 was adjusted for age and sex. Model 3 was adjusted for BMI, duration of diabetes, and the covariates in Model 2. Model 4 was adjusted for smoking status, HbA1c, systolic blood pressure, total cholesterol, HDL cholesterol, log-transformed triglycerides, uric acid, and the covariates in Model 3. Model 5 was adjusted for eGFR, log-transformed urine albumin-to-creatinine ratio, and the covariates in Model 4.

GSM, gray-scale median; SD, standard deviation; CV, coefficient of variation; MAGE, mean amplitude of glycemic excursion; TIR, time in range; TAR, time above range; TBR, time below range; HBGI, high blood glucose index; LBGI, low blood glucose index; MODD, mean of daily differences; IQR, interquartile range

**Table S5 Associations between the adjustment factors in the multivariable regression models**

|  | sex | age | BMI | Dur | Smok | A1c | SBP | TC | HDL | logTG | UA | eGFR |
| --- | --- | --- | --- | --- | --- | --- | --- | --- | --- | --- | --- | --- |
| age | 0.030 | - | - | - | - | - | - | - | - | - | - | - |
| BMI | 0.082 | -0.277 | - | - | - | - | - | - | - | - | - | - |
| Dur | 0.011 | 0.323 | -0.109 | - | - | - | - | - | - | - | - | - |
| Smok | -0.477 | -0.094 | -0.001 | -0.071 | - | - | - | - | - | - | - | - |
| A1c | 0.032 | -0.088 | 0.135 | 0.199 | 0.053 | - | - | - | - | - | - | - |
| SBP | 0.012 | 0.126 | 0.075 | 0.030 | 0.007 | -0.027 | - | - | - | - | - | - |
| TC | 0.105 | 0.001 | -0.058 | -0.087 | -0.090 | 0.035 | 0.074 | - | - | - | - | - |
| HDL | 0.175 | 0.101 | -0.296 | 0.050 | -0.127 | -0.109 | 0.005 | 0.362 | - | - | - | - |
| logTG | -0.020 | -0.094 | 0.273 | -0.133 | 0.109 | 0.193 | 0.087 | 0.172 | -0.435 | - | - | - |
| UA | -0.383 | -0.023 | 0.162 | -0.017 | 0.246 | -0.043 | 0.054 | -0.056 | -0.216 | 0.171 | - | - |
| eGFR | 0.060 | -0.385 | 0.057 | -0.151 | 0.007 | 0.064 | -0.021 | 0.060 | 0.037 | -0.038 | -0.275 | - |
| log u-Alb | 0.022 | 0.076 | 0.149 | 0.132 | 0.072 | 0.210 | 0.181 | -0.022 | -0.088 | 0.198 | 0.128 | -0.152 |

Pearson's correlation coefficients represent the associations between the adjustment factors.

Dur, duration of diabetes; Smok, smoking status; A1c, HbA1c; SBP, systolic blood pressure; TC, total cholesterol; HDL, HDL cholesterol; logTG, log-transformed triglycerides; UA, uric acid; eGFR, estimated glomerular filtration rate; log u-Alb, log-transformed urine albumin-to-creatinine ratio

**Table S6 Associations of the metrics of continuous glucose monitoring with intima-media thickness among the** participants with hypertension

|  | mean-IMT (n = 347) | |  | CCA-max-IMT (n = 347) | |
| --- | --- | --- | --- | --- | --- |
|  | β (95% CI) | *P* value |  | β (95% CI) | *P* value |
| Mean glucose (mmol/L) | | | | | |
| Model 1 | 0.000 (-0.010, 0.011) | 0.937 |  | 0.010 (-0.023, 0.043) | 0.562 |
| Model 2 | -0.001 (-0.011, 0.009) | 0.867 |  | 0.007 (-0.026, 0.040) | 0.667 |
| Model 3 | -0.001 (-0.011, 0.010) | 0.931 |  | 0.003 (-0.030, 0.036) | 0.848 |
| Model 4 | 0.004 (-0.013, 0.020) | 0.667 |  | 0.025 (-0.027, 0.078) | 0.344 |
| Model 5 | 0.007 (-0.010, 0.024) | 0.418 |  | 0.031 (-0.024, 0.085) | 0.272 |
| Median glucose (mmol/L) | | | | | |
| Model 1 | 0.000 (-0.030, 0.029) | 0.981 |  | 0.042 (-0.050, 0.134) | 0.366 |
| Model 2 | -0.010 (-0.038, 0.019) | 0.511 |  | 0.020 (-0.072, 0.111) | 0.674 |
| Model 3 | -0.011 (-0.040, 0.018) | 0.455 |  | 0.012 (-0.082, 0.105) | 0.808 |
| Model 4 | -0.011 (-0.045, 0.023) | 0.520 |  | 0.034 (-0.074, 0.142) | 0.535 |
| Model 5 | -0.007 (-0.042, 0.028) | 0.688 |  | 0.039 (-0.072, 0.150) | 0.488 |
| SD (mmol/L) | | | | | |
| Model 1 | 0.000 (-0.003, 0.003) | 0.945 |  | 0.004 (-0.005, 0.013) | 0.396 |
| Model 2 | -0.001 (-0.004, 0.002) | 0.564 |  | 0.002 (-0.008, 0.011) | 0.742 |
| Model 3 | -0.001 (-0.004, 0.002) | 0.424 |  | 0.002 (-0.008, 0.011) | 0.722 |
| Model 4 | -0.001 (-0.004, 0.002) | 0.398 |  | 0.002 (-0.008, 0.011) | 0.730 |
| Model 5 | -0.001 (-0.004, 0.002) | 0.445 |  | 0.002 (-0.008, 0.011) | 0.708 |
| CV (%) | | | | | |
| Model 1 | 0.001 (-0.008, 0.010) | 0.826 |  | 0.012 (-0.016, 0.039) | 0.402 |
| Model 2 | -0.001 (-0.010, 0.007) | 0.751 |  | 0.006 (-0.021, 0.033) | 0.659 |
| Model 3 | -0.002 (-0.011, 0.007) | 0.669 |  | 0.006 (-0.022, 0.033) | 0.696 |
| Model 4 | -0.002 (-0.012, 0.007) | 0.651 |  | 0.013 (-0.018, 0.043) | 0.412 |
| Model 5 | -0.001 (-0.011, 0.009) | 0.862 |  | 0.015 (-0.016, 0.047) | 0.346 |
| MAGE (mmol/L) | | | | | |
| Model 1 | 0.001 (-0.010, 0.011) | 0.910 |  | 0.009 (-0.024, 0.041) | 0.605 |
| Model 2 | -0.001 (-0.011, 0.009) | 0.905 |  | 0.006 (-0.026, 0.038) | 0.709 |
| Model 3 | 0.000 (-0.010, 0.010) | 0.989 |  | 0.002 (-0.031, 0.034) | 0.922 |
| Model 4 | 0.004 (-0.012, 0.020) | 0.598 |  | 0.021 (-0.030, 0.072) | 0.424 |
| Model 5 | 0.008 (-0.009, 0.024) | 0.375 |  | 0.026 (-0.028, 0.079) | 0.345 |
| TIR (%): 3.9–10.0 mmol/L | | | | | |
| Model 1 | -0.003 (-0.013, 0.007) | 0.613 |  | -0.018 (-0.050, 0.013) | 0.255 |
| Model 2 | 0.000 (-0.010, 0.009) | 0.955 |  | -0.013 (-0.044, 0.018) | 0.420 |
| Model 3 | -0.001 (-0.011, 0.009) | 0.909 |  | -0.009 (-0.040, 0.023) | 0.579 |
| Model 4 | -0.003 (-0.017, 0.011) | 0.714 |  | -0.025 (-0.069, 0.020) | 0.272 |
| Model 5 | -0.004 (-0.019, 0.010) | 0.542 |  | -0.028 (-0.074, 0.018) | 0.227 |
| TAR (%): >10.0 mmol/L | | | | | |
| Model 1 | 0.000 (-0.001, 0.001) | 0.925 |  | 0.001 (-0.002, 0.004) | 0.497 |
| Model 2 | 0.000 (-0.001, 0.001) | 0.809 |  | 0.001 (-0.002, 0.004) | 0.653 |
| Model 3 | 0.000 (-0.001, 0.001) | 0.882 |  | 0.000 (-0.003, 0.003) | 0.859 |
| Model 4 | 0.000 (-0.001, 0.002) | 0.838 |  | 0.002 (-0.003, 0.007) | 0.442 |
| Model 5 | 0.000 (-0.001, 0.002) | 0.593 |  | 0.002 (-0.003, 0.007) | 0.378 |
| TAR (%): >13.9 mmol/L | | | | | |
| Model 1 | -0.001 (-0.003, 0.002) | 0.669 |  | 0.000 (-0.007, 0.008) | 0.949 |
| Model 2 | -0.001 (-0.003, 0.002) | 0.508 |  | 0.000 (-0.007, 0.007) | 0.929 |
| Model 3 | -0.001 (-0.003, 0.002) | 0.545 |  | -0.001 (-0.008, 0.006) | 0.794 |
| Model 4 | -0.001 (-0.004, 0.003) | 0.736 |  | 0.000 (-0.010, 0.010) | 0.998 |
| Model 5 | 0.000 (-0.003, 0.003) | 0.821 |  | 0.000 (-0.010, 0.010) | 0.959 |
| TBR (%): <3.9 mmol/L | | | | | |
| Model 1 | 0.002 (-0.001, 0.006) | 0.166 |  | 0.008 (-0.002, 0.019) | 0.131 |
| Model 2 | 0.002 (-0.002, 0.005) | 0.309 |  | 0.006 (-0.004, 0.017) | 0.236 |
| Model 3 | 0.002 (-0.002, 0.005) | 0.375 |  | 0.007 (-0.004, 0.017) | 0.212 |
| Model 4 | 0.001 (-0.003, 0.004) | 0.658 |  | 0.005 (-0.006, 0.016) | 0.332 |
| Model 5 | 0.001 (-0.003, 0.004) | 0.770 |  | 0.006 (-0.006, 0.017) | 0.324 |
| TBR (%): <3.0 mmol/L | | | | | |
| Model 1 | 0.008 (-0.004, 0.019) | 0.190 |  | 0.034 (-0.002, 0.069) | 0.061 |
| Model 2 | 0.006 (-0.005, 0.017) | 0.277 |  | 0.030 (-0.005, 0.065) | 0.096 |
| Model 3 | 0.006 (-0.005, 0.017) | 0.311 |  | 0.030 (-0.005, 0.065) | 0.095 |
| Model 4 | 0.004 (-0.007, 0.016) | 0.437 |  | 0.032 (-0.003, 0.068) | 0.075 |
| Model 5 | 0.004 (-0.007, 0.016) | 0.484 |  | 0.033 (-0.004, 0.070) | 0.083 |
| HBGI | | | | | |
| Model 1 | -0.001 (-0.005, 0.004) | 0.792 |  | 0.002 (-0.011, 0.015) | 0.730 |
| Model 2 | -0.001 (-0.005, 0.003) | 0.518 |  | 0.001 (-0.012, 0.013) | 0.944 |
| Model 3 | -0.001 (-0.005, 0.003) | 0.527 |  | -0.001 (-0.014, 0.012) | 0.904 |
| Model 4 | -0.001 (-0.007, 0.005) | 0.748 |  | 0.004 (-0.015, 0.023) | 0.664 |
| Model 5 | 0.000 (-0.006, 0.006) | 0.945 |  | 0.005 (-0.014, 0.025) | 0.593 |
| LBGI | | | | | |
| Model 1 | 0.001 (-0.008, 0.011) | 0.783 |  | 0.004 (-0.026, 0.035) | 0.780 |
| Model 2 | 0.000 (-0.010, 0.009) | 0.932 |  | -0.001 (-0.031, 0.029) | 0.954 |
| Model 3 | -0.001 (-0.011, 0.008) | 0.821 |  | 0.000 (-0.030, 0.030) | 0.999 |
| Model 4 | -0.003 (-0.013, 0.007) | 0.537 |  | -0.004 (-0.036, 0.029) | 0.828 |
| Model 5 | -0.004 (-0.015, 0.006) | 0.417 |  | -0.004 (-0.037, 0.029) | 0.803 |
| MODD (mmol/L) | | | | | |
| Model 1 | 0.002 (-0.028, 0.032) | 0.898 |  | -0.001 (-0.096, 0.095) | 0.990 |
| Model 2 | 0.000 (-0.029, 0.029) | 0.996 |  | -0.005 (-0.099, 0.088) | 0.911 |
| Model 3 | -0.001 (-0.031, 0.030) | 0.968 |  | -0.018 (-0.115, 0.078) | 0.708 |
| Model 4 | 0.004 (-0.034, 0.041) | 0.851 |  | -0.012 (-0.132, 0.107) | 0.842 |
| Model 5 | 0.007 (-0.032, 0.046) | 0.718 |  | -0.012 (-0.137, 0.113) | 0.850 |
| IQR (mmol/L) | | | | | |
| Model 1 | -0.007 (-0.031, 0.017) | 0.582 |  | -0.011 (-0.087, 0.066) | 0.785 |
| Model 2 | -0.006 (-0.029, 0.017) | 0.613 |  | -0.008 (-0.083, 0.066) | 0.828 |
| Model 3 | -0.007 (-0.031, 0.017) | 0.568 |  | -0.017 (-0.094, 0.059) | 0.658 |
| Model 4 | -0.008 (-0.039, 0.024) | 0.634 |  | -0.020 (-0.119, 0.078) | 0.687 |
| Model 5 | -0.004 (-0.036, 0.028) | 0.802 |  | -0.019 (-0.121, 0.084) | 0.721 |

Univariable and multivariable linear regression analysis. Model 1 was not adjusted. Model 2 was adjusted for age and sex. Model 3 was adjusted for BMI, duration of diabetes, and the covariates in Model 2. Model 4 was adjusted for smoking status, HbA1c, systolic blood pressure, total cholesterol, HDL cholesterol, log-transformed triglycerides, uric acid, and the covariates in Model 3. Model 5 was adjusted for eGFR, log-transformed urine albumin-to-creatinine ratio, and the covariates in Model 4.

IMT, intima-media thickness; SD, standard deviation; CV, coefficient of variation; MAGE, mean amplitude of glycemic excursion; TIR, time in range; TAR, time above range; TBR, time below range; HBGI, high blood glucose index; LBGI, low blood glucose index; MODD, mean of daily differences; IQR, interquartile range

**Table S7 Associations of the metrics of continuous glucose monitoring with gray-scale median among the participants with hypertension**

|  | mean-GSM  (n = 346) | |  | thickened lesion-GSM  (n = 328) | |  | plaque-GSM  (n = 286) | |
| --- | --- | --- | --- | --- | --- | --- | --- | --- |
|  | β (95 % CI) | *P* value |  | β (95 % CI) | *P* value |  | β (95 % CI) | *P* value |
| Mean glucose (mmol/L) | | | | | | | | |
| Model 1 | -2.000 (-3.250, -0.751) | 0.002 |  | -1.417 (-2.695, -0.139) | 0.030 |  | -2.438 (-4.489, -0.387) | 0.020 |
| Model 2 | -2.086 (-3.323, -0.849) | 0.001 |  | -1.330 (-2.601, -0.058) | 0.040 |  | -2.333 (-4.395, -0.272) | 0.027 |
| Model 3 | -1.763 (-2.997, -0.530) | 0.005 |  | -1.158 (-2.448, 0.133) | 0.078 |  | -1.993 (-4.072, 0.086) | 0.060 |
| Model 4 | -2.254 (-4.255, -0.254) | 0.027 |  | -0.555 (-2.652, 1.542) | 0.603 |  | -2.000 (-5.541, 1.541) | 0.267 |
| Model 5 | -1.627 (-3.634, 0.380) | 0.112 |  | -0.058 (-2.165, 2.049) | 0.957 |  | -1.385 (-4.978, 2.207) | 0.448 |
| Median glucose (mmol/L) | | | | | | | | |
| Model 1 | -6.933 (-10.379, -3.487) | <0.001 |  | -4.500 (-8.071, -0.929) | 0.014 |  | -5.031 (-10.920, 0.857) | 0.094 |
| Model 2 | -6.375 (-9.816, -2.934) | <0.001 |  | -4.000 (-7.557, -0.443) | 0.028 |  | -4.579 (-10.501, 1.344) | 0.129 |
| Model 3 | -5.954 (-9.421, -2.488) | <0.001 |  | -3.430 (-7.089, 0.230) | 0.066 |  | -3.404 (-9.470, 2.662) | 0.270 |
| Model 4 | -6.109 (-10.183, -2.035) | 0.003 |  | -2.236 (-6.546, 2.074) | 0.308 |  | -1.667 (-8.882, 5.549) | 0.650 |
| Model 5 | -5.127 (-9.173, -1.081) | 0.013 |  | -1.074 (-5.360, 3.213) | 0.622 |  | -0.716 (-7.964, 6.531) | 0.846 |
| SD (mmol/L) | | | | | | | | |
| Model 1 | -0.416 (-0.759, -0.073) | 0.018 |  | -0.227 (-0.586, 0.131) | 0.213 |  | -0.009 (-0.595, 0.577) | 0.976 |
| Model 2 | -0.330 (-0.675, 0.014) | 0.060 |  | -0.180 (-0.539, 0.179) | 0.325 |  | 0.028 (-0.563, 0.618) | 0.927 |
| Model 3 | -0.363 (-0.707, -0.019) | 0.039 |  | -0.143 (-0.509, 0.224) | 0.445 |  | 0.093 (-0.508, 0.694) | 0.761 |
| Model 4 | -0.404 (-0.753, -0.055) | 0.024 |  | -0.143 (-0.514, 0.229) | 0.450 |  | 0.035 (-0.587, 0.658) | 0.911 |
| Model 5 | -0.383 (-0.728, -0.039) | 0.029 |  | -0.077 (-0.444, 0.290) | 0.680 |  | 0.068 (-0.554, 0.689) | 0.830 |
| CV (%) | | | | | | | | |
| Model 1 | -1.675 (-2.716, -0.633) | 0.002 |  | -1.180 (-2.246, -0.113) | 0.030 |  | -1.196 (-3.002, 0.611) | 0.194 |
| Model 2 | -1.604 (-2.639, -0.568) | 0.002 |  | -1.035 (-2.095, 0.025) | 0.056 |  | -1.072 (-2.885, 0.741) | 0.245 |
| Model 3 | -1.584 (-2.610, -0.559) | 0.003 |  | -0.921 (-1.994, 0.152) | 0.092 |  | -0.869 (-2.696, 0.957) | 0.350 |
| Model 4 | -1.493 (-2.652, -0.335) | 0.012 |  | -0.639 (-1.852, 0.575) | 0.301 |  | -0.405 (-2.476, 1.667) | 0.701 |
| Model 5 | -1.193 (-2.346, -0.041) | 0.042 |  | -0.290 (-1.499, 0.920) | 0.638 |  | -0.131 (-2.213, 1.952) | 0.902 |
| MAGE (mmol/L) | | | | | | | | |
| Model 1 | -1.884 (-3.106, -0.662) | 0.003 |  | -1.355 (-2.606, -0.104) | 0.034 |  | -2.271 (-4.276, -0.266) | 0.027 |
| Model 2 | -1.965 (-3.174, -0.756) | 0.002 |  | -1.270 (-2.514, -0.025) | 0.046 |  | -2.166 (-4.182, -0.151) | 0.035 |
| Model 3 | -1.603 (-2.814, -0.392) | 0.010 |  | -1.093 (-2.359, 0.174) | 0.091 |  | -1.802 (-3.840, 0.236) | 0.083 |
| Model 4 | -1.848 (-3.804, 0.107) | 0.064 |  | -0.379 (-2.428, 1.671) | 0.716 |  | -1.434 (-4.889, 2.020) | 0.414 |
| Model 5 | -1.273 (-3.231, 0.685) | 0.202 |  | 0.032 (-2.022, 2.086) | 0.976 |  | -0.889 (-4.380, 2.601) | 0.616 |
| TIR (%): 3.9–10.0 mmol/L | | | | | | | | |
| Model 1 | 2.240 (1.061, 3.419) | <0.001 |  | 1.590 (0.382, 2.799) | 0.010 |  | 1.621 (-0.365, 3.606) | 0.109 |
| Model 2 | 2.169 (0.999, 3.338) | <0.001 |  | 1.440 (0.239, 2.641) | 0.019 |  | 1.464 (-0.531, 3.459) | 0.150 |
| Model 3 | 1.864 (0.689, 3.038) | 0.002 |  | 1.255 (0.028, 2.483) | 0.045 |  | 1.064 (-0.957, 3.084) | 0.301 |
| Model 4 | 2.418 (0.743, 4.094) | 0.005 |  | 0.981 (-0.788, 2.749) | 0.276 |  | 0.143 (-2.769, 3.056) | 0.923 |
| Model 5 | 2.083 (0.414, 3.752) | 0.015 |  | 0.429 (-1.336, 2.195) | 0.633 |  | -0.416 (-3.352, 2.520) | 0.781 |
| TAR (%): >10.0 mmol/L | | | | | | | | |
| Model 1 | -0.220 (-0.336, -0.103) | <0.001 |  | -0.157 (-0.276, -0.037) | 0.010 |  | -0.215 (-0.409, -0.020) | 0.030 |
| Model 2 | -0.220 (-0.335, -0.105) | <0.001 |  | -0.146 (-0.265, -0.028) | 0.016 |  | -0.203 (-0.398, -0.008) | 0.042 |
| Model 3 | -0.187 (-0.303, -0.072) | 0.002 |  | -0.129 (-0.250, -0.008) | 0.037 |  | -0.166 (-0.364, 0.032) | 0.100 |
| Model 4 | -0.267 (-0.450, -0.085) | 0.004 |  | -0.105 (-0.297, 0.088) | 0.286 |  | -0.131 (-0.455, 0.193) | 0.426 |
| Model 5 | -0.209 (-0.391, -0.028) | 0.024 |  | -0.047 (-0.239, 0.146) | 0.633 |  | -0.069 (-0.395, 0.256) | 0.675 |
| TAR (%): >13.9 mmol/L | | | | | | | | |
| Model 1 | -0.335 (-0.609, -0.061) | 0.017 |  | -0.181 (-0.457, 0.096) | 0.200 |  | -0.195 (-0.636, 0.246) | 0.385 |
| Model 2 | -0.341 (-0.611, -0.070) | 0.014 |  | -0.165 (-0.439, 0.109) | 0.237 |  | -0.172 (-0.614, 0.270) | 0.445 |
| Model 3 | -0.288 (-0.555, -0.020) | 0.035 |  | -0.136 (-0.412, 0.140) | 0.334 |  | -0.119 (-0.561, 0.323) | 0.596 |
| Model 4 | -0.302 (-0.666, 0.062) | 0.104 |  | -0.007 (-0.384, 0.370) | 0.971 |  | 0.107 (-0.507, 0.721) | 0.731 |
| Model 5 | -0.252 (-0.610, 0.106) | 0.167 |  | 0.057 (-0.315, 0.428) | 0.764 |  | 0.169 (-0.440, 0.777) | 0.586 |
| TBR (%): <3.9 mmol/L | | | | | | | | |
| Model 1 | 0.020 (-0.385, 0.425) | 0.924 |  | 0.019 (-0.398, 0.435) | 0.930 |  | 0.636 (-0.008, 1.279) | 0.053 |
| Model 2 | 0.113 (-0.290, 0.515) | 0.583 |  | 0.071 (-0.346, 0.487) | 0.740 |  | 0.675 (0.029, 1.321) | 0.041 |
| Model 3 | 0.079 (-0.318, 0.476) | 0.695 |  | 0.082 (-0.336, 0.501) | 0.699 |  | 0.679 (0.034, 1.325) | 0.039 |
| Model 4 | -0.103 (-0.522, 0.315) | 0.628 |  | -0.060 (-0.498, 0.379) | 0.789 |  | 0.536 (-0.166, 1.237) | 0.134 |
| Model 5 | -0.193 (-0.610, 0.224) | 0.363 |  | -0.022 (-0.460, 0.416) | 0.923 |  | 0.585 (-0.129, 1.298) | 0.108 |
| TBR (%): <3.0 mmol/L | | | | | | | | |
| Model 1 | -0.426 (-1.788, 0.936) | 0.539 |  | 0.320 (-1.054, 1.694) | 0.647 |  | 0.903 (-1.203, 3.009) | 0.399 |
| Model 2 | -0.124 (-1.481, 1.234) | 0.858 |  | 0.402 (-0.970, 1.775) | 0.564 |  | 0.980 (-1.139, 3.100) | 0.363 |
| Model 3 | -0.138 (-1.471, 1.196) | 0.839 |  | 0.447 (-0.926, 1.819) | 0.522 |  | 1.031 (-1.082, 3.144) | 0.338 |
| Model 4 | -0.599 (-1.969, 0.772) | 0.390 |  | 0.097 (-1.311, 1.505) | 0.892 |  | 0.689 (-1.531, 2.909) | 0.542 |
| Model 5 | -0.543 (-1.905, 0.818) | 0.433 |  | 0.384 (-1.017, 1.784) | 0.590 |  | 0.892 (-1.340, 3.125) | 0.432 |
| HBGI | | | | | | | | |
| Model 1 | -0.875 (-1.367, -0.383) | <0.001 |  | -0.528 (-1.030, -0.026) | 0.039 |  | -0.681 (-1.487, 0.125) | 0.097 |
| Model 2 | -0.856 (-1.343, -0.369) | <0.001 |  | -0.481 (-0.979, 0.017) | 0.058 |  | -0.631 (-1.439, 0.177) | 0.125 |
| Model 3 | -0.762 (-1.247, -0.278) | 0.002 |  | -0.413 (-0.918, 0.091) | 0.108 |  | -0.506 (-1.319, 0.307) | 0.221 |
| Model 4 | -1.046 (-1.760, -0.332) | 0.004 |  | -0.243 (-0.995, 0.510) | 0.526 |  | -0.235 (-1.469, 1.000) | 0.708 |
| Model 5 | -0.895 (-1.603, -0.186) | 0.013 |  | -0.048 (-0.795, 0.700) | 0.900 |  | -0.039 (-1.276, 1.197) | 0.950 |
| LBGI | | | | | | | | |
| Model 1 | -0.023 (-1.182, 1.136) | 0.969 |  | 0.336 (-0.861, 1.532) | 0.582 |  | 1.938 (0.069, 3.807) | 0.042 |
| Model 2 | 0.295 (-0.864, 1.453) | 0.617 |  | 0.445 (-0.758, 1.648) | 0.467 |  | 2.022 (0.137, 3.907) | 0.036 |
| Model 3 | 0.190 (-0.953, 1.333) | 0.744 |  | 0.480 (-0.727, 1.687) | 0.434 |  | 2.071 (0.186, 3.956) | 0.031 |
| Model 4 | -0.414 (-1.639, 0.811) | 0.506 |  | 0.076 (-1.218, 1.369) | 0.908 |  | 1.726 (-0.373, 3.825) | 0.107 |
| Model 5 | -0.672 (-1.890, 0.546) | 0.279 |  | 0.188 (-1.102, 1.477) | 0.775 |  | 1.817 (-0.308, 3.943) | 0.093 |
| MODD (mmol/L) | | | | | | | | |
| Model 1 | -7.280 (-10.841, -3.719) | <0.001 |  | -5.321 (-8.992, -1.650) | 0.005 |  | -5.369 (-11.412, 0.674) | 0.081 |
| Model 2 | -7.167 (-10.682, -3.652) | <0.001 |  | -5.259 (-8.887, -1.631) | 0.005 |  | -5.308 (-11.344, 0.727) | 0.085 |
| Model 3 | -6.572 (-10.151, -2.992) | <0.001 |  | -4.691 (-8.452, -0.931) | 0.015 |  | -4.034 (-10.247, 2.179) | 0.202 |
| Model 4 | -8.055 (-12.561, -3.548) | <0.001 |  | -3.721 (-8.498, 1.057) | 0.126 |  | -1.680 (-9.622, 6.263) | 0.677 |
| Model 5 | -7.211 (-11.749, -2.673) | 0.002 |  | -2.145 (-6.959, 2.668) | 0.381 |  | -0.536 (-8.565, 7.494) | 0.896 |
| IQR (mmol/L) | | | | | | | | |
| Model 1 | -4.552 (-7.428, -1.676) | 0.002 |  | -2.474 (-5.423, 0.476) | 0.100 |  | -2.587 (-7.395, 2.221) | 0.290 |
| Model 2 | -4.696 (-7.531, -1.860) | 0.001 |  | -2.601 (-5.517, 0.316) | 0.080 |  | -2.673 (-7.479, 2.133) | 0.275 |
| Model 3 | -4.217 (-7.080, -1.354) | 0.004 |  | -2.086 (-5.084, 0.912) | 0.172 |  | -1.702 (-6.606, 3.201) | 0.495 |
| Model 4 | -4.969 (-8.717, -1.220) | 0.010 |  | -0.231 (-4.185, 3.723) | 0.909 |  | 0.780 (-5.741, 7.302) | 0.814 |
| Model 5 | -4.251 (-8.003, -0.499) | 0.027 |  | 0.884 (-3.069, 4.837) | 0.660 |  | 1.609 (-4.957, 8.175) | 0.630 |

Univariable and multivariable linear regression analysis. Model 1 was not adjusted. Model 2 was adjusted for age and sex. Model 3 was adjusted for BMI, duration of diabetes, and the covariates in Model 2. Model 4 was adjusted for smoking status, HbA1c, systolic blood pressure, total cholesterol, HDL cholesterol, log-transformed triglycerides, uric acid, and the covariates in Model 3. Model 5 was adjusted for eGFR, log-transformed urine albumin-to-creatinine ratio, and the covariates in Model 4.

GSM, gray-scale median; SD, standard deviation; CV, coefficient of variation; MAGE, mean amplitude of glycemic excursion; TIR, time in range; TAR, time above range; TBR, time below range; HBGI, high blood glucose index; LBGI, low blood glucose index; MODD, mean of daily differences; IQR, interquartile range

**Table S8 Associations of the metrics of continuous glucose monitoring with intima-media thickness among the** participants without hypertension

|  | mean-IMT (n = 253) | |  | CCA-max-IMT (n = 253) | |
| --- | --- | --- | --- | --- | --- |
|  | β (95% CI) | *P* value |  | β (95% CI) | *P* value |
| Mean glucose (mmol/L) | | | | | |
| Model 1 | -0.002 (-0.012, 0.008) | 0.666 |  | 0.003 (-0.019, 0.024) | 0.804 |
| Model 2 | -0.001 (-0.010, 0.007) | 0.742 |  | 0.003 (-0.017, 0.023) | 0.754 |
| Model 3 | -0.001 (-0.009, 0.008) | 0.829 |  | 0.004 (-0.017, 0.024) | 0.735 |
| Model 4 | 0.006 (-0.008, 0.020) | 0.389 |  | 0.001 (-0.031, 0.034) | 0.931 |
| Model 5 | 0.007 (-0.008, 0.021) | 0.368 |  | 0.003 (-0.030, 0.035) | 0.871 |
| Median glucose (mmol/L) | | | | | |
| Model 1 | 0.008 (-0.019, 0.036) | 0.551 |  | 0.039 (-0.021, 0.100) | 0.200 |
| Model 2 | -0.004 (-0.028, 0.020) | 0.742 |  | 0.022 (-0.036, 0.079) | 0.457 |
| Model 3 | 0.000 (-0.024, 0.025) | 0.983 |  | 0.035 (-0.023, 0.093) | 0.239 |
| Model 4 | 0.012 (-0.020, 0.043) | 0.465 |  | 0.057 (-0.014, 0.129) | 0.115 |
| Model 5 | 0.010 (-0.021, 0.042) | 0.522 |  | 0.051 (-0.022, 0.123) | 0.168 |
| SD (mmol/L) | | | | | |
| Model 1 | 0.002 (-0.001, 0.005) | 0.119 |  | 0.006 (-0.001, 0.012) | 0.076 |
| Model 2 | 0.000 (-0.003, 0.003) | 0.894 |  | 0.003 (-0.003, 0.009) | 0.362 |
| Model 3 | 0.001 (-0.002, 0.004) | 0.583 |  | 0.005 (-0.002, 0.011) | 0.134 |
| Model 4 | 0.001 (-0.002, 0.004) | 0.553 |  | 0.006 (0.000, 0.013) | 0.052 |
| Model 5 | 0.001 (-0.002, 0.004) | 0.636 |  | 0.006 (-0.001, 0.012) | 0.091 |
| CV (%) | | | | | |
| Model 1 | 0.004 (-0.004, 0.013) | 0.344 |  | 0.017 (-0.002, 0.036) | 0.070 |
| Model 2 | -0.001 (-0.008, 0.007) | 0.885 |  | 0.011 (-0.007, 0.029) | 0.243 |
| Model 3 | 0.000 (-0.007, 0.008) | 0.920 |  | 0.014 (-0.004, 0.032) | 0.137 |
| Model 4 | 0.004 (-0.005, 0.013) | 0.410 |  | 0.020 (0.000, 0.041) | 0.055 |
| Model 5 | 0.003 (-0.006, 0.013) | 0.467 |  | 0.019 (-0.002, 0.040) | 0.083 |
| MAGE (mmol/L) | | | | | |
| Model 1 | -0.003 (-0.013, 0.006) | 0.479 |  | 0.000 (-0.021, 0.020) | 0.968 |
| Model 2 | -0.002 (-0.011, 0.006) | 0.604 |  | 0.001 (-0.019, 0.021) | 0.933 |
| Model 3 | -0.002 (-0.010, 0.007) | 0.681 |  | 0.001 (-0.019, 0.021) | 0.933 |
| Model 4 | 0.003 (-0.011, 0.017) | 0.631 |  | -0.008 (-0.040, 0.025) | 0.645 |
| Model 5 | 0.004 (-0.010, 0.018) | 0.610 |  | -0.006 (-0.039, 0.026) | 0.706 |
| TIR (%): 3.9–10.0 mmol/L | | | | | |
| Model 1 | 0.001 (-0.008, 0.010) | 0.836 |  | -0.002 (-0.022, 0.019) | 0.886 |
| Model 2 | 0.002 (-0.006, 0.010) | 0.694 |  | 0.000 (-0.019, 0.019) | 0.997 |
| Model 3 | 0.001 (-0.007, 0.009) | 0.803 |  | 0.000 (-0.019, 0.019) | 0.979 |
| Model 4 | -0.005 (-0.017, 0.008) | 0.474 |  | 0.001 (-0.028, 0.030) | 0.946 |
| Model 5 | -0.004 (-0.017, 0.008) | 0.484 |  | 0.002 (-0.027, 0.031) | 0.895 |
| TAR (%): >10.0 mmol/L | | | | | |
| Model 1 | 0.000 (-0.001, 0.001) | 0.696 |  | 0.000 (-0.002, 0.002) | 0.934 |
| Model 2 | 0.000 (-0.001, 0.001) | 0.663 |  | 0.000 (-0.002, 0.002) | 0.963 |
| Model 3 | 0.000 (-0.001, 0.001) | 0.752 |  | 0.000 (-0.002, 0.002) | 0.947 |
| Model 4 | 0.000 (-0.001, 0.002) | 0.503 |  | 0.000 (-0.003, 0.003) | 0.924 |
| Model 5 | 0.000 (-0.001, 0.002) | 0.489 |  | 0.000 (-0.003, 0.003) | 0.936 |
| TAR (%): >13.9 mmol/L | | | | | |
| Model 1 | -0.001 (-0.003, 0.001) | 0.276 |  | 0.000 (-0.004, 0.005) | 0.869 |
| Model 2 | -0.001 (-0.003, 0.001) | 0.257 |  | 0.000 (-0.004, 0.004) | 0.844 |
| Model 3 | -0.001 (-0.003, 0.001) | 0.279 |  | 0.001 (-0.004, 0.005) | 0.794 |
| Model 4 | -0.001 (-0.003, 0.002) | 0.621 |  | 0.001 (-0.005, 0.007) | 0.813 |
| Model 5 | -0.001 (-0.003, 0.002) | 0.576 |  | 0.000 (-0.006, 0.007) | 0.915 |
| TBR (%): <3.9 mmol/L | | | | | |
| Model 1 | 0.002 (-0.002, 0.006) | 0.372 |  | 0.001 (-0.008, 0.010) | 0.792 |
| Model 2 | 0.001 (-0.003, 0.004) | 0.791 |  | -0.001 (-0.010, 0.008) | 0.812 |
| Model 3 | 0.001 (-0.003, 0.004) | 0.720 |  | -0.001 (-0.009, 0.008) | 0.845 |
| Model 4 | 0.000 (-0.004, 0.004) | 0.925 |  | 0.000 (-0.008, 0.009) | 0.929 |
| Model 5 | 0.000 (-0.004, 0.004) | 0.985 |  | -0.001 (-0.009, 0.008) | 0.878 |
| TBR (%): <3.0 mmol/L | | | | | |
| Model 1 | 0.005 (-0.010, 0.019) | 0.522 |  | 0.001 (-0.031, 0.032) | 0.959 |
| Model 2 | 0.001 (-0.011, 0.014) | 0.836 |  | -0.005 (-0.035, 0.025) | 0.750 |
| Model 3 | 0.002 (-0.010, 0.015) | 0.723 |  | -0.004 (-0.033, 0.026) | 0.818 |
| Model 4 | 0.003 (-0.010, 0.016) | 0.647 |  | 0.001 (-0.028, 0.030) | 0.948 |
| Model 5 | 0.002 (-0.011, 0.015) | 0.730 |  | -0.003 (-0.032, 0.027) | 0.856 |
| HBGI | | | | | |
| Model 1 | -0.001 (-0.005, 0.003) | 0.655 |  | 0.003 (-0.006, 0.011) | 0.500 |
| Model 2 | -0.001 (-0.005, 0.002) | 0.500 |  | 0.002 (-0.006, 0.010) | 0.564 |
| Model 3 | -0.001 (-0.004, 0.003) | 0.605 |  | 0.003 (-0.005, 0.011) | 0.472 |
| Model 4 | 0.001 (-0.004, 0.007) | 0.639 |  | 0.007 (-0.006, 0.020) | 0.288 |
| Model 5 | 0.001 (-0.004, 0.007) | 0.684 |  | 0.006 (-0.007, 0.019) | 0.355 |
| LBGI | | | | | |
| Model 1 | 0.004 (-0.008, 0.015) | 0.549 |  | 0.004 (-0.022, 0.030) | 0.786 |
| Model 2 | -0.001 (-0.011, 0.010) | 0.927 |  | -0.003 (-0.027, 0.022) | 0.839 |
| Model 3 | 0.001 (-0.010, 0.011) | 0.891 |  | 0.001 (-0.024, 0.025) | 0.961 |
| Model 4 | -0.001 (-0.013, 0.010) | 0.853 |  | 0.008 (-0.018, 0.035) | 0.546 |
| Model 5 | -0.002 (-0.014, 0.010) | 0.709 |  | 0.004 (-0.023, 0.031) | 0.774 |
| MODD (mmol/L) | | | | | |
| Model 1 | -0.017 (-0.042, 0.008) | 0.179 |  | 0.005 (-0.051, 0.060) | 0.864 |
| Model 2 | -0.012 (-0.034, 0.010) | 0.283 |  | 0.011 (-0.042, 0.064) | 0.681 |
| Model 3 | -0.009 (-0.032, 0.014) | 0.435 |  | 0.018 (-0.035, 0.072) | 0.503 |
| Model 4 | 0.001 (-0.031, 0.032) | 0.977 |  | 0.039 (-0.033, 0.112) | 0.286 |
| Model 5 | -0.001 (-0.032, 0.031) | 0.967 |  | 0.036 (-0.037, 0.109) | 0.331 |
| IQR (mmol/L) | | | | | |
| Model 1 | -0.017 (-0.038, 0.004) | 0.112 |  | -0.010 (-0.055, 0.036) | 0.677 |
| Model 2 | -0.013 (-0.031, 0.005) | 0.164 |  | -0.006 (-0.049, 0.038) | 0.806 |
| Model 3 | -0.010 (-0.029, 0.009) | 0.282 |  | 0.001 (-0.043, 0.046) | 0.953 |
| Model 4 | -0.002 (-0.028, 0.023) | 0.856 |  | 0.011 (-0.048, 0.069) | 0.717 |
| Model 5 | -0.003 (-0.028, 0.023) | 0.821 |  | 0.009 (-0.050, 0.068) | 0.765 |

Univariable and multivariable linear regression analysis. Model 1 was not adjusted. Model 2 was adjusted for age and sex. Model 3 was adjusted for BMI, duration of diabetes, and the covariates in Model 2. Model 4 was adjusted for smoking status, HbA1c, systolic blood pressure, total cholesterol, HDL cholesterol, log-transformed triglycerides, uric acid, and the covariates in Model 3. Model 5 was adjusted for eGFR, log-transformed urine albumin-to-creatinine ratio, and the covariates in Model 4.

IMT, intima-media thickness; SD, standard deviation; CV, coefficient of variation; MAGE, mean amplitude of glycemic excursion; TIR, time in range; TAR, time above range; TBR, time below range; HBGI, high blood glucose index; LBGI, low blood glucose index; MODD, mean of daily differences; IQR, interquartile range

**Table S9 Associations of the metrics of continuous glucose monitoring with gray-scale median among the participants without hypertension**

|  | mean-GSM  (n = 253) | |  | thickened lesion-GSM  (n = 238) | |  | plaque-GSM  (n = 204) | |
| --- | --- | --- | --- | --- | --- | --- | --- | --- |
|  | β (95 % CI) | *P* value |  | β (95 % CI) | *P* value |  | β (95 % CI) | *P* value |
| Mean glucose (mmol/L) | | | | | | | | |
| Model 1 | -1.617 (-2.942, -0.292) | 0.017 |  | -2.273 (-3.669, -0.876) | 0.002 |  | -3.389 (-5.663, -1.115) | 0.004 |
| Model 2 | -1.723 (-3.048, -0.397) | 0.011 |  | -2.293 (-3.659, -0.926) | 0.001 |  | -3.335 (-5.598, -1.071) | 0.004 |
| Model 3 | -1.499 (-2.781, -0.216) | 0.022 |  | -2.261 (-3.636, -0.886) | 0.001 |  | -3.291 (-5.567, -1.016) | 0.005 |
| Model 4 | -1.650 (-3.793, 0.493) | 0.131 |  | -2.176 (-4.567, 0.215) | 0.074 |  | -3.495 (-7.626, 0.637) | 0.097 |
| Model 5 | -1.585 (-3.738, 0.568) | 0.148 |  | -2.141 (-4.553, 0.272) | 0.082 |  | -3.391 (-7.552, 0.770) | 0.110 |
| Median glucose (mmol/L) | | | | | | | | |
| Model 1 | -4.241 (-8.026, -0.456) | 0.028 |  | -6.588 (-10.490, -2.686) | 0.001 |  | -10.415 (-16.916, -3.915) | 0.002 |
| Model 2 | -4.057 (-7.863, -0.252) | 0.037 |  | -5.830 (-9.681, -1.980) | 0.003 |  | -10.456 (-16.915, -3.997) | 0.002 |
| Model 3 | -4.508 (-8.220, -0.796) | 0.018 |  | -6.183 (-10.111, -2.254) | 0.002 |  | -10.984 (-17.582, -4.386) | 0.001 |
| Model 4 | -6.177 (-10.857, -1.497) | 0.010 |  | -6.092 (-11.315, -0.869) | 0.022 |  | -9.569 (-18.515, -0.623) | 0.036 |
| Model 5 | -6.208 (-10.930, -1.486) | 0.010 |  | -6.053 (-11.352, -0.755) | 0.025 |  | -10.281 (-19.354, -1.207) | 0.027 |
| SD (mmol/L) | | | | | | | | |
| Model 1 | -0.194 (-0.606, 0.218) | 0.354 |  | -0.328 (-0.759, 0.102) | 0.134 |  | -0.568 (-1.292, 0.156) | 0.124 |
| Model 2 | -0.146 (-0.565, 0.274) | 0.495 |  | -0.196 (-0.626, 0.235) | 0.371 |  | -0.582 (-1.314, 0.150) | 0.118 |
| Model 3 | -0.253 (-0.665, 0.160) | 0.229 |  | -0.235 (-0.678, 0.209) | 0.298 |  | -0.645 (-1.403, 0.113) | 0.095 |
| Model 4 | -0.441 (-0.861, -0.020) | 0.040 |  | -0.312 (-0.781, 0.158) | 0.192 |  | -0.517 (-1.317, 0.284) | 0.204 |
| Model 5 | -0.454 (-0.880, -0.027) | 0.037 |  | -0.312 (-0.790, 0.167) | 0.201 |  | -0.601 (-1.422, 0.219) | 0.150 |
| CV (%) | | | | | | | | |
| Model 1 | -1.622 (-2.799, -0.444) | 0.007 |  | -1.988 (-3.221, -0.754) | 0.002 |  | -2.541 (-4.598, -0.484) | 0.016 |
| Model 2 | -1.568 (-2.756, -0.381) | 0.010 |  | -1.714 (-2.935, -0.493) | 0.006 |  | -2.528 (-4.578, -0.479) | 0.016 |
| Model 3 | -1.687 (-2.832, -0.541) | 0.004 |  | -1.776 (-3.010, -0.542) | 0.005 |  | -2.615 (-4.690, -0.540) | 0.014 |
| Model 4 | -2.193 (-3.549, -0.837) | 0.002 |  | -1.647 (-3.200, -0.094) | 0.038 |  | -1.512 (-4.139, 1.116) | 0.258 |
| Model 5 | -2.259 (-3.624, -0.893) | 0.001 |  | -1.660 (-3.230, -0.089) | 0.038 |  | -1.708 (-4.375, 0.959) | 0.208 |
| MAGE (mmol/L) | | | | | | | | |
| Model 1 | -1.500 (-2.801, -0.200) | 0.024 |  | -2.122 (-3.499, -0.746) | 0.003 |  | -3.146 (-5.371, -0.922) | 0.006 |
| Model 2 | -1.620 (-2.922, -0.319) | 0.015 |  | -2.177 (-3.524, -0.830) | 0.002 |  | -3.097 (-5.312, -0.882) | 0.006 |
| Model 3 | -1.356 (-2.618, -0.094) | 0.035 |  | -2.137 (-3.494, -0.779) | 0.002 |  | -3.040 (-5.270, -0.811) | 0.008 |
| Model 4 | -1.194 (-3.325, 0.938) | 0.271 |  | -1.831 (-4.211, 0.550) | 0.131 |  | -2.818 (-6.869, 1.233) | 0.172 |
| Model 5 | -1.185 (-3.324, 0.955) | 0.276 |  | -1.812 (-4.212, 0.588) | 0.138 |  | -2.744 (-6.824, 1.336) | 0.186 |
| TIR (%): 3.9–10.0 mmol/L | | | | | | | | |
| Model 1 | 1.599 (0.348, 2.850) | 0.012 |  | 2.343 (1.021, 3.666) | <0.001 |  | 2.665 (0.487, 4.844) | 0.017 |
| Model 2 | 1.694 (0.442, 2.947) | 0.008 |  | 2.252 (0.954, 3.550) | <0.001 |  | 2.552 (0.384, 4.719) | 0.021 |
| Model 3 | 1.439 (0.221, 2.657) | 0.021 |  | 2.225 (0.914, 3.535) | <0.001 |  | 2.500 (0.313, 4.687) | 0.025 |
| Model 4 | 2.002 (0.126, 3.878) | 0.037 |  | 2.422 (0.328, 4.517) | 0.024 |  | 1.112 (-2.497, 4.721) | 0.544 |
| Model 5 | 1.925 (0.039, 3.811) | 0.045 |  | 2.378 (0.262, 4.495) | 0.028 |  | 1.136 (-2.494, 4.766) | 0.538 |
| TAR (%): >10.0 mmol/L | | | | | | | | |
| Model 1 | -0.157 (-0.278, -0.036) | 0.011 |  | -0.227 (-0.355, -0.099) | <0.001 |  | -0.294 (-0.504, -0.084) | 0.006 |
| Model 2 | -0.165 (-0.286, -0.044) | 0.008 |  | -0.222 (-0.348, -0.097) | <0.001 |  | -0.288 (-0.497, -0.079) | 0.007 |
| Model 3 | -0.143 (-0.260, -0.026) | 0.017 |  | -0.220 (-0.346, -0.094) | <0.001 |  | -0.284 (-0.494, -0.074) | 0.008 |
| Model 4 | -0.182 (-0.366, 0.002) | 0.052 |  | -0.240 (-0.446, -0.035) | 0.022 |  | -0.226 (-0.580, 0.128) | 0.209 |
| Model 5 | -0.174 (-0.359, 0.011) | 0.065 |  | -0.236 (-0.443, -0.028) | 0.026 |  | -0.222 (-0.578, 0.133) | 0.219 |
| TAR (%): >13.9 mmol/L | | | | | | | | |
| Model 1 | -0.209 (-0.477, 0.059) | 0.126 |  | -0.379 (-0.659, -0.100) | 0.008 |  | -0.610 (-1.048, -0.172) | 0.007 |
| Model 2 | -0.229 (-0.497, 0.040) | 0.095 |  | -0.378 (-0.651, -0.104) | 0.007 |  | -0.594 (-1.030, -0.158) | 0.008 |
| Model 3 | -0.233 (-0.490, 0.025) | 0.076 |  | -0.384 (-0.658, -0.111) | 0.006 |  | -0.605 (-1.043, -0.167) | 0.007 |
| Model 4 | -0.269 (-0.675, 0.137) | 0.193 |  | -0.299 (-0.750, 0.153) | 0.193 |  | -0.375 (-1.121, 0.370) | 0.322 |
| Model 5 | -0.276 (-0.683, 0.131) | 0.183 |  | -0.300 (-0.756, 0.156) | 0.196 |  | -0.414 (-1.167, 0.339) | 0.279 |
| TBR (%): <3.9 mmol/L | | | | | | | | |
| Model 1 | 0.169 (-0.405, 0.744) | 0.562 |  | 0.153 (-0.456, 0.762) | 0.621 |  | 1.018 (-0.003, 2.039) | 0.051 |
| Model 2 | 0.162 (-0.415, 0.739) | 0.581 |  | 0.261 (-0.336, 0.858) | 0.390 |  | 1.121 (0.107, 2.136) | 0.030 |
| Model 3 | 0.215 (-0.339, 0.770) | 0.445 |  | 0.285 (-0.315, 0.885) | 0.351 |  | 1.202 (0.177, 2.227) | 0.022 |
| Model 4 | -0.090 (-0.656, 0.476) | 0.754 |  | 0.063 (-0.569, 0.695) | 0.844 |  | 1.107 (0.034, 2.181) | 0.043 |
| Model 5 | -0.103 (-0.676, 0.470) | 0.723 |  | 0.067 (-0.576, 0.710) | 0.837 |  | 1.079 (-0.016, 2.174) | 0.053 |
| TBR (%): <3.0 mmol/L | | | | | | | | |
| Model 1 | -1.236 (-3.230, 0.758) | 0.223 |  | -0.574 (-2.649, 1.500) | 0.586 |  | 0.267 (-3.596, 4.130) | 0.892 |
| Model 2 | -1.303 (-3.300, 0.695) | 0.200 |  | -0.336 (-2.370, 1.697) | 0.745 |  | 0.516 (-3.321, 4.353) | 0.791 |
| Model 3 | -1.101 (-3.029, 0.827) | 0.262 |  | -0.270 (-2.318, 1.779) | 0.796 |  | 0.827 (-3.082, 4.736) | 0.677 |
| Model 4 | -1.680 (-3.609, 0.249) | 0.087 |  | -0.674 (-2.795, 1.447) | 0.532 |  | 0.637 (-3.440, 4.715) | 0.758 |
| Model 5 | -1.777 (-3.731, 0.177) | 0.074 |  | -0.703 (-2.863, 1.457) | 0.522 |  | 0.410 (-3.752, 4.571) | 0.846 |
| HBGI | | | | | | | | |
| Model 1 | -0.633 (-1.157, -0.109) | 0.018 |  | -0.938 (-1.485, -0.392) | <0.001 |  | -1.393 (-2.268, -0.518) | 0.002 |
| Model 2 | -0.653 (-1.177, -0.130) | 0.015 |  | -0.904 (-1.438, -0.369) | 0.001 |  | -1.368 (-2.236, -0.499) | 0.002 |
| Model 3 | -0.624 (-1.129, -0.119) | 0.016 |  | -0.910 (-1.448, -0.373) | <0.001 |  | -1.377 (-2.251, -0.503) | 0.002 |
| Model 4 | -0.995 (-1.836, -0.155) | 0.020 |  | -1.055 (-1.996, -0.115) | 0.028 |  | -1.423 (-3.028, 0.182) | 0.082 |
| Model 5 | -0.988 (-1.833, -0.144) | 0.022 |  | -1.045 (-1.996, -0.093) | 0.032 |  | -1.493 (-3.111, 0.124) | 0.070 |
| LBGI | | | | | | | | |
| Model 1 | 0.834 (-0.807, 2.475) | 0.318 |  | 0.927 (-0.804, 2.658) | 0.293 |  | 1.759 (-1.161, 4.678) | 0.236 |
| Model 2 | 0.889 (-0.755, 2.533) | 0.288 |  | 1.220 (-0.473, 2.913) | 0.157 |  | 1.851 (-1.053, 4.754) | 0.210 |
| Model 3 | 0.853 (-0.737, 2.443) | 0.292 |  | 1.236 (-0.475, 2.948) | 0.156 |  | 2.008 (-0.956, 4.971) | 0.183 |
| Model 4 | -0.425 (-2.174, 1.325) | 0.633 |  | 0.306 (-1.637, 2.250) | 0.756 |  | 1.709 (-1.642, 5.061) | 0.316 |
| Model 5 | -0.593 (-2.383, 1.197) | 0.515 |  | 0.305 (-1.693, 2.302) | 0.764 |  | 1.465 (-2.020, 4.950) | 0.408 |
| MODD (mmol/L) | | | | | | | | |
| Model 1 | -3.385 (-6.859, 0.089) | 0.056 |  | -4.868 (-8.489, -1.247) | 0.009 |  | -9.777 (-15.721, -3.834) | 0.001 |
| Model 2 | -3.786 (-7.271, -0.302) | 0.033 |  | -5.134 (-8.679, -1.589) | 0.005 |  | -9.916 (-15.839, -3.992) | 0.001 |
| Model 3 | -3.400 (-6.822, 0.021) | 0.051 |  | -5.236 (-8.858, -1.613) | 0.005 |  | -10.007 (-16.058, -3.956) | 0.001 |
| Model 4 | -5.076 (-9.846, -0.306) | 0.037 |  | -4.507 (-9.835, 0.821) | 0.097 |  | -10.842 (-20.248, -1.436) | 0.024 |
| Model 5 | -5.340 (-10.124, -0.555) | 0.029 |  | -4.558 (-9.937, 0.821) | 0.096 |  | -11.317 (-20.798, -1.836) | 0.020 |
| IQR (mmol/L) | | | | | | | | |
| Model 1 | -2.532 (-5.411, 0.346) | 0.084 |  | -4.330 (-7.329, -1.330) | 0.005 |  | -9.082 (-13.997, -4.166) | <0.001 |
| Model 2 | -2.905 (-5.797, -0.013) | 0.049 |  | -4.520 (-7.459, -1.581) | 0.003 |  | -9.145 (-14.055, -4.235) | <0.001 |
| Model 3 | -2.762 (-5.602, 0.078) | 0.057 |  | -4.691 (-7.699, -1.682) | 0.002 |  | -9.332 (-14.364, -4.299) | <0.001 |
| Model 4 | -4.075 (-7.912, -0.238) | 0.037 |  | -4.533 (-8.791, -0.275) | 0.037 |  | -10.354 (-17.924, -2.784) | 0.008 |
| Model 5 | -4.168 (-8.011, -0.325) | 0.034 |  | -4.526 (-8.817, -0.236) | 0.039 |  | -10.615 (-18.229, -3.001) | 0.007 |

Univariable and multivariable linear regression analysis. Model 1 was not adjusted. Model 2 was adjusted for age and sex. Model 3 was adjusted for BMI, duration of diabetes, and the covariates in Model 2. Model 4 was adjusted for smoking status, HbA1c, systolic blood pressure, total cholesterol, HDL cholesterol, log-transformed triglycerides, uric acid, and the covariates in Model 3. Model 5 was adjusted for eGFR, log-transformed urine albumin-to-creatinine ratio, and the covariates in Model 4.

GSM, gray-scale median; SD, standard deviation; CV, coefficient of variation; MAGE, mean amplitude of glycemic excursion; TIR, time in range; TAR, time above range; TBR, time below range; HBGI, high blood glucose index; LBGI, low blood glucose index; MODD, mean of daily differences; IQR, interquartile range

**Table S10 Associations of the metrics of continuous glucose monitoring with intima-media thickness among the** participants with dyslipidemia

|  | mean-IMT (n = 450) | |  | CCA-max-IMT (n = 450) | |
| --- | --- | --- | --- | --- | --- |
|  | β (95% CI) | *P* value |  | β (95% CI) | *P* value |
| Mean glucose (mmol/L) | | | | | |
| Model 1 | -0.002 (-0.011, 0.007) | 0.628 |  | 0.005 (-0.022, 0.032) | 0.734 |
| Model 2 | -0.002 (-0.010, 0.007) | 0.678 |  | 0.006 (-0.020, 0.032) | 0.660 |
| Model 3 | -0.002 (-0.010, 0.007) | 0.703 |  | 0.002 (-0.024, 0.029) | 0.860 |
| Model 4 | 0.006 (-0.008, 0.021) | 0.373 |  | 0.017 (-0.026, 0.060) | 0.434 |
| Model 5 | 0.009 (-0.005, 0.023) | 0.223 |  | 0.022 (-0.022, 0.065) | 0.336 |
| Median glucose (mmol/L) | | | | | |
| Model 1 | -0.003 (-0.028, 0.023) | 0.839 |  | 0.040 (-0.036, 0.115) | 0.301 |
| Model 2 | -0.012 (-0.036, 0.013) | 0.348 |  | 0.020 (-0.054, 0.094) | 0.599 |
| Model 3 | -0.012 (-0.037, 0.013) | 0.343 |  | 0.018 (-0.058, 0.094) | 0.643 |
| Model 4 | -0.006 (-0.037, 0.025) | 0.699 |  | 0.041 (-0.053, 0.134) | 0.395 |
| Model 5 | -0.004 (-0.035, 0.027) | 0.799 |  | 0.044 (-0.052, 0.139) | 0.371 |
| SD (mmol/L) | | | | | |
| Model 1 | 0.001 (-0.002, 0.003) | 0.635 |  | 0.006 (-0.002, 0.013) | 0.171 |
| Model 2 | -0.001 (-0.003, 0.002) | 0.532 |  | 0.002 (-0.006, 0.010) | 0.569 |
| Model 3 | -0.001 (-0.004, 0.002) | 0.508 |  | 0.003 (-0.005, 0.011) | 0.443 |
| Model 4 | -0.001 (-0.004, 0.002) | 0.442 |  | 0.003 (-0.005, 0.011) | 0.480 |
| Model 5 | -0.001 (-0.004, 0.002) | 0.428 |  | 0.003 (-0.005, 0.011) | 0.504 |
| CV (%) | | | | | |
| Model 1 | 0.001 (-0.006, 0.009) | 0.732 |  | 0.017 (-0.005, 0.039) | 0.134 |
| Model 2 | -0.002 (-0.009, 0.005) | 0.554 |  | 0.010 (-0.012, 0.032) | 0.394 |
| Model 3 | -0.002 (-0.010, 0.005) | 0.546 |  | 0.011 (-0.012, 0.033) | 0.351 |
| Model 4 | 0.000 (-0.009, 0.009) | 0.969 |  | 0.022 (-0.004, 0.048) | 0.101 |
| Model 5 | 0.000 (-0.008, 0.009) | 0.924 |  | 0.024 (-0.003, 0.050) | 0.084 |
| MAGE (mmol/L) | | | | | |
| Model 1 | -0.003 (-0.011, 0.006) | 0.577 |  | 0.003 (-0.024, 0.029) | 0.834 |
| Model 2 | -0.002 (-0.010, 0.007) | 0.664 |  | 0.005 (-0.021, 0.031) | 0.727 |
| Model 3 | -0.002 (-0.010, 0.007) | 0.695 |  | 0.000 (-0.026, 0.026) | 0.988 |
| Model 4 | 0.006 (-0.008, 0.020) | 0.375 |  | 0.011 (-0.031, 0.053) | 0.611 |
| Model 5 | 0.009 (-0.006, 0.023) | 0.230 |  | 0.015 (-0.028, 0.058) | 0.493 |
| TIR (%): 3.9–10.0 mmol/L | | | | | |
| Model 1 | 0.000 (-0.009, 0.008) | 0.984 |  | -0.010 (-0.035, 0.016) | 0.450 |
| Model 2 | 0.001 (-0.007, 0.009) | 0.811 |  | -0.008 (-0.032, 0.017) | 0.549 |
| Model 3 | 0.001 (-0.007, 0.009) | 0.841 |  | -0.004 (-0.029, 0.021) | 0.755 |
| Model 4 | -0.005 (-0.017, 0.007) | 0.394 |  | -0.014 (-0.051, 0.023) | 0.451 |
| Model 5 | -0.006 (-0.019, 0.006) | 0.299 |  | -0.016 (-0.053, 0.021) | 0.393 |
| TAR (%): >10.0 mmol/L | | | | | |
| Model 1 | 0.000 (-0.001, 0.001) | 0.622 |  | 0.000 (-0.002, 0.003) | 0.792 |
| Model 2 | 0.000 (-0.001, 0.001) | 0.546 |  | 0.000 (-0.002, 0.003) | 0.823 |
| Model 3 | 0.000 (-0.001, 0.001) | 0.568 |  | 0.000 (-0.003, 0.002) | 0.961 |
| Model 4 | 0.000 (-0.001, 0.002) | 0.629 |  | 0.001 (-0.003, 0.004) | 0.727 |
| Model 5 | 0.001 (-0.001, 0.002) | 0.467 |  | 0.001 (-0.003, 0.005) | 0.636 |
| TAR (%): >13.9 mmol/L | | | | | |
| Model 1 | -0.001 (-0.003, 0.001) | 0.401 |  | 0.001 (-0.005, 0.006) | 0.854 |
| Model 2 | -0.001 (-0.003, 0.001) | 0.444 |  | 0.001 (-0.005, 0.007) | 0.768 |
| Model 3 | -0.001 (-0.003, 0.001) | 0.461 |  | 0.000 (-0.006, 0.006) | 0.946 |
| Model 4 | 0.000 (-0.003, 0.003) | 0.869 |  | 0.002 (-0.007, 0.010) | 0.682 |
| Model 5 | 0.000 (-0.002, 0.003) | 0.785 |  | 0.002 (-0.006, 0.010) | 0.618 |
| TBR (%): <3.9 mmol/L | | | | | |
| Model 1 | 0.004 (0.000, 0.007) | 0.036 |  | 0.010 (0.000, 0.020) | 0.056 |
| Model 2 | 0.003 (-0.001, 0.006) | 0.127 |  | 0.007 (-0.003, 0.017) | 0.144 |
| Model 3 | 0.003 (-0.001, 0.006) | 0.124 |  | 0.007 (-0.003, 0.017) | 0.155 |
| Model 4 | 0.002 (-0.002, 0.005) | 0.300 |  | 0.006 (-0.004, 0.016) | 0.260 |
| Model 5 | 0.002 (-0.002, 0.005) | 0.368 |  | 0.006 (-0.005, 0.016) | 0.282 |
| TBR (%): <3.0 mmol/L | | | | | |
| Model 1 | 0.018 (0.004, 0.032) | 0.011 |  | 0.060 (0.019, 0.101) | 0.004 |
| Model 2 | 0.014 (0.001, 0.027) | 0.035 |  | 0.051 (0.011, 0.091) | 0.012 |
| Model 3 | 0.014 (0.001, 0.027) | 0.034 |  | 0.049 (0.009, 0.089) | 0.016 |
| Model 4 | 0.013 (0.000, 0.027) | 0.044 |  | 0.050 (0.011, 0.090) | 0.013 |
| Model 5 | 0.013 (0.000, 0.026) | 0.053 |  | 0.049 (0.009, 0.089) | 0.017 |
| HBGI | | | | | |
| Model 1 | -0.001 (-0.005, 0.003) | 0.535 |  | 0.003 (-0.008, 0.014) | 0.627 |
| Model 2 | -0.001 (-0.005, 0.002) | 0.451 |  | 0.002 (-0.008, 0.013) | 0.664 |
| Model 3 | -0.001 (-0.005, 0.002) | 0.465 |  | 0.001 (-0.010, 0.012) | 0.823 |
| Model 4 | 0.001 (-0.005, 0.007) | 0.759 |  | 0.008 (-0.010, 0.025) | 0.388 |
| Model 5 | 0.002 (-0.004, 0.007) | 0.605 |  | 0.009 (-0.009, 0.026) | 0.334 |
| LBGI | | | | | |
| Model 1 | 0.006 (-0.004, 0.015) | 0.251 |  | 0.013 (-0.016, 0.042) | 0.383 |
| Model 2 | 0.002 (-0.007, 0.011) | 0.672 |  | 0.004 (-0.024, 0.033) | 0.766 |
| Model 3 | 0.002 (-0.007, 0.011) | 0.671 |  | 0.004 (-0.024, 0.033) | 0.760 |
| Model 4 | -0.001 (-0.011, 0.009) | 0.866 |  | 0.001 (-0.029, 0.031) | 0.960 |
| Model 5 | -0.002 (-0.012, 0.008) | 0.678 |  | -0.002 (-0.032, 0.029) | 0.926 |
| MODD (mmol/L) | | | | | |
| Model 1 | -0.011 (-0.036, 0.015) | 0.410 |  | -0.002 (-0.077, 0.074) | 0.969 |
| Model 2 | -0.009 (-0.033, 0.015) | 0.453 |  | 0.002 (-0.071, 0.075) | 0.958 |
| Model 3 | -0.009 (-0.034, 0.016) | 0.458 |  | -0.006 (-0.082, 0.069) | 0.872 |
| Model 4 | 0.002 (-0.031, 0.035) | 0.888 |  | 0.006 (-0.094, 0.105) | 0.911 |
| Model 5 | 0.004 (-0.029, 0.037) | 0.811 |  | 0.006 (-0.096, 0.107) | 0.909 |
| IQR (mmol/L) | | | | | |
| Model 1 | -0.015 (-0.035, 0.006) | 0.168 |  | -0.013 (-0.076, 0.049) | 0.675 |
| Model 2 | -0.011 (-0.031, 0.009) | 0.265 |  | -0.005 (-0.066, 0.056) | 0.866 |
| Model 3 | -0.012 (-0.032, 0.009) | 0.264 |  | -0.012 (-0.074, 0.051) | 0.709 |
| Model 4 | -0.006 (-0.033, 0.021) | 0.643 |  | -0.012 (-0.094, 0.070) | 0.772 |
| Model 5 | -0.004 (-0.031, 0.023) | 0.765 |  | -0.011 (-0.094, 0.072) | 0.793 |

Univariable and multivariable linear regression analysis. Model 1 was not adjusted. Model 2 was adjusted for age and sex. Model 3 was adjusted for BMI, duration of diabetes, and the covariates in Model 2. Model 4 was adjusted for smoking status, HbA1c, systolic blood pressure, total cholesterol, HDL cholesterol, log-transformed triglycerides, uric acid, and the covariates in Model 3. Model 5 was adjusted for eGFR, log-transformed urine albumin-to-creatinine ratio, and the covariates in Model 4.

IMT, intima-media thickness; SD, standard deviation; CV, coefficient of variation; MAGE, mean amplitude of glycemic excursion; TIR, time in range; TAR, time above range; TBR, time below range; HBGI, high blood glucose index; LBGI, low blood glucose index; MODD, mean of daily differences; IQR, interquartile range

**Table S11 Associations of the metrics of continuous glucose monitoring with gray-scale median among the participants with dyslipidemia**

|  | mean-GSM  (n = 449) | |  | thickened lesion-GSM  (n = 419) | |  | plaque-GSM  (n = 368) | |
| --- | --- | --- | --- | --- | --- | --- | --- | --- |
|  | β (95 % CI) | *P* value |  | β (95 % CI) | *P* value |  | β (95 % CI) | *P* value |
| Mean glucose (mmol/L) | | | | | | | | |
| Model 1 | -1.970 (-3.050, -0.891) | <0.001 |  | -1.960 (-3.152, -0.768) | 0.001 |  | -3.020 (-4.879, -1.161) | 0.002 |
| Model 2 | -2.094 (-3.163, -1.024) | <0.001 |  | -1.991 (-3.163, -0.820) | <0.001 |  | -2.994 (-4.849, -1.139) | 0.002 |
| Model 3 | -1.833 (-2.899, -0.768) | <0.001 |  | -1.906 (-3.093, -0.718) | 0.002 |  | -2.820 (-4.698, -0.941) | 0.003 |
| Model 4 | -1.776 (-3.549, -0.003) | 0.050 |  | -0.825 (-2.788, 1.138) | 0.409 |  | -2.710 (-6.007, 0.587) | 0.107 |
| Model 5 | -1.467 (-3.242, 0.309) | 0.105 |  | -0.735 (-2.707, 1.237) | 0.464 |  | -2.506 (-5.824, 0.812) | 0.138 |
| Median glucose (mmol/L) | | | | | | | | |
| Model 1 | -6.425 (-9.426, -3.425) | <0.001 |  | -5.850 (-9.155, -2.544) | <0.001 |  | -7.518 (-12.887, -2.149) | 0.006 |
| Model 2 | -5.970 (-8.970, -2.970) | <0.001 |  | -5.245 (-8.509, -1.981) | 0.002 |  | -7.347 (-12.716, -1.978) | 0.007 |
| Model 3 | -5.716 (-8.752, -2.680) | <0.001 |  | -4.991 (-8.363, -1.620) | 0.004 |  | -6.955 (-12.497, -1.413) | 0.014 |
| Model 4 | -5.783 (-9.628, -1.938) | 0.003 |  | -2.927 (-7.207, 1.353) | 0.180 |  | -4.137 (-11.347, 3.073) | 0.260 |
| Model 5 | -5.398 (-9.240, -1.556) | 0.006 |  | -2.437 (-6.727, 1.853) | 0.265 |  | -4.225 (-11.471, 3.020) | 0.252 |
| SD (mmol/L) | | | | | | | | |
| Model 1 | -0.394 (-0.711, -0.078) | 0.015 |  | -0.307 (-0.660, 0.047) | 0.089 |  | -0.215 (-0.779, 0.349) | 0.453 |
| Model 2 | -0.306 (-0.627, 0.014) | 0.061 |  | -0.207 (-0.560, 0.146) | 0.250 |  | -0.190 (-0.759, 0.379) | 0.512 |
| Model 3 | -0.331 (-0.655, -0.007) | 0.045 |  | -0.172 (-0.537, 0.194) | 0.356 |  | -0.156 (-0.743, 0.432) | 0.602 |
| Model 4 | -0.392 (-0.725, -0.058) | 0.021 |  | -0.162 (-0.534, 0.211) | 0.394 |  | -0.107 (-0.721, 0.506) | 0.731 |
| Model 5 | -0.393 (-0.726, -0.059) | 0.021 |  | -0.118 (-0.491, 0.255) | 0.535 |  | -0.129 (-0.745, 0.488) | 0.681 |
| CV (%) | | | | | | | | |
| Model 1 | -1.829 (-2.721, -0.938) | <0.001 |  | -1.797 (-2.772, -0.822) | <0.001 |  | -1.925 (-3.524, -0.326) | 0.018 |
| Model 2 | -1.738 (-2.631, -0.845) | <0.001 |  | -1.545 (-2.512, -0.578) | 0.002 |  | -1.764 (-3.367, -0.161) | 0.031 |
| Model 3 | -1.751 (-2.637, -0.865) | <0.001 |  | -1.489 (-2.469, -0.509) | 0.003 |  | -1.711 (-3.334, -0.089) | 0.039 |
| Model 4 | -1.766 (-2.847, -0.685) | 0.001 |  | -1.050 (-2.253, 0.154) | 0.087 |  | -0.894 (-2.905, 1.116) | 0.382 |
| Model 5 | -1.681 (-2.762, -0.599) | 0.002 |  | -0.875 (-2.083, 0.333) | 0.155 |  | -0.904 (-2.929, 1.122) | 0.381 |
| MAGE (mmol/L) | | | | | | | | |
| Model 1 | -1.898 (-2.963, -0.833) | <0.001 |  | -1.930 (-3.109, -0.751) | 0.001 |  | -2.883 (-4.714, -1.052) | 0.002 |
| Model 2 | -2.037 (-3.093, -0.982) | <0.001 |  | -1.979 (-3.138, -0.819) | <0.001 |  | -2.860 (-4.688, -1.032) | 0.002 |
| Model 3 | -1.731 (-2.787, -0.674) | 0.001 |  | -1.893 (-3.072, -0.715) | 0.002 |  | -2.673 (-4.530, -0.816) | 0.005 |
| Model 4 | -1.421 (-3.170, 0.327) | 0.111 |  | -0.690 (-2.628, 1.247) | 0.484 |  | -2.180 (-5.415, 1.054) | 0.186 |
| Model 5 | -1.169 (-2.918, 0.580) | 0.190 |  | -0.667 (-2.610, 1.276) | 0.500 |  | -2.025 (-5.273, 1.223) | 0.221 |
| TIR (%): 3.9–10.0 mmol/L | | | | | | | | |
| Model 1 | 2.150 (1.149, 3.152) | <0.001 |  | 2.002 (0.890, 3.114) | <0.001 |  | 2.092 (0.324, 3.861) | 0.021 |
| Model 2 | 2.178 (1.185, 3.171) | <0.001 |  | 1.892 (0.798, 2.985) | <0.001 |  | 2.001 (0.235, 3.766) | 0.026 |
| Model 3 | 1.922 (0.927, 2.916) | <0.001 |  | 1.805 (0.691, 2.918) | 0.002 |  | 1.802 (0.010, 3.595) | 0.049 |
| Model 4 | 2.289 (0.786, 3.791) | 0.003 |  | 1.137 (-0.546, 2.821) | 0.185 |  | 0.216 (-2.525, 2.958) | 0.877 |
| Model 5 | 2.219 (0.730, 3.708) | 0.004 |  | 1.006 (-0.671, 2.682) | 0.239 |  | 0.100 (-2.635, 2.835) | 0.943 |
| TAR (%): >10.0 mmol/L | | | | | | | | |
| Model 1 | -0.201 (-0.299, -0.104) | <0.001 |  | -0.191 (-0.299, -0.083) | <0.001 |  | -0.250 (-0.420, -0.079) | 0.004 |
| Model 2 | -0.207 (-0.303, -0.111) | <0.001 |  | -0.186 (-0.292, -0.080) | <0.001 |  | -0.244 (-0.414, -0.074) | 0.005 |
| Model 3 | -0.184 (-0.280, -0.087) | <0.001 |  | -0.178 (-0.286, -0.071) | 0.001 |  | -0.228 (-0.400, -0.056) | 0.010 |
| Model 4 | -0.220 (-0.375, -0.066) | 0.005 |  | -0.115 (-0.287, 0.057) | 0.188 |  | -0.153 (-0.438, 0.132) | 0.292 |
| Model 5 | -0.199 (-0.352, -0.046) | 0.011 |  | -0.103 (-0.274, 0.068) | 0.238 |  | -0.140 (-0.424, 0.144) | 0.334 |
| TAR (%): >13.9 mmol/L | | | | | | | | |
| Model 1 | -0.336 (-0.572, -0.099) | 0.006 |  | -0.326 (-0.584, -0.068) | 0.013 |  | -0.445 (-0.843, -0.047) | 0.029 |
| Model 2 | -0.362 (-0.597, -0.127) | 0.003 |  | -0.335 (-0.588, -0.082) | 0.010 |  | -0.444 (-0.841, -0.046) | 0.029 |
| Model 3 | -0.314 (-0.546, -0.081) | 0.008 |  | -0.320 (-0.575, -0.065) | 0.014 |  | -0.414 (-0.814, -0.014) | 0.042 |
| Model 4 | -0.222 (-0.561, 0.118) | 0.200 |  | -0.058 (-0.429, 0.313) | 0.759 |  | -0.065 (-0.670, 0.540) | 0.832 |
| Model 5 | -0.217 (-0.553, 0.118) | 0.204 |  | -0.052 (-0.420, 0.315) | 0.780 |  | -0.041 (-0.641, 0.559) | 0.893 |
| TBR (%): <3.9 mmol/L | | | | | | | | |
| Model 1 | -0.013 (-0.417, 0.391) | 0.951 |  | 0.033 (-0.418, 0.485) | 0.885 |  | 0.832 (0.160, 1.503) | 0.015 |
| Model 2 | 0.041 (-0.361, 0.443) | 0.842 |  | 0.136 (-0.309, 0.582) | 0.548 |  | 0.886 (0.216, 1.556) | 0.010 |
| Model 3 | 0.073 (-0.323, 0.469) | 0.717 |  | 0.162 (-0.285, 0.609) | 0.476 |  | 0.920 (0.249, 1.590) | 0.007 |
| Model 4 | -0.139 (-0.553, 0.274) | 0.507 |  | -0.022 (-0.483, 0.439) | 0.924 |  | 0.827 (0.111, 1.543) | 0.024 |
| Model 5 | -0.245 (-0.658, 0.168) | 0.244 |  | -0.015 (-0.478, 0.448) | 0.948 |  | 0.846 (0.123, 1.569) | 0.022 |
| TBR (%): <3.0 mmol/L | | | | | | | | |
| Model 1 | -1.062 (-2.731, 0.607) | 0.212 |  | -0.535 (-2.353, 1.283) | 0.563 |  | 1.048 (-1.683, 3.780) | 0.451 |
| Model 2 | -0.861 (-2.522, 0.800) | 0.309 |  | -0.226 (-2.015, 1.563) | 0.804 |  | 1.256 (-1.474, 3.986) | 0.366 |
| Model 3 | -0.660 (-2.296, 0.975) | 0.428 |  | -0.143 (-1.936, 1.649) | 0.875 |  | 1.399 (-1.333, 4.132) | 0.315 |
| Model 4 | -1.074 (-2.726, 0.577) | 0.202 |  | -0.463 (-2.258, 1.332) | 0.612 |  | 1.113 (-1.694, 3.920) | 0.436 |
| Model 5 | -1.120 (-2.759, 0.518) | 0.180 |  | -0.328 (-2.116, 1.460) | 0.719 |  | 1.122 (-1.676, 3.921) | 0.431 |
| HBGI | | | | | | | | |
| Model 1 | -0.920 (-1.359, -0.481) | <0.001 |  | -0.848 (-1.331, -0.366) | <0.001 |  | -1.175 (-1.929, -0.421) | 0.002 |
| Model 2 | -0.928 (-1.363, -0.494) | <0.001 |  | -0.832 (-1.305, -0.359) | <0.001 |  | -1.166 (-1.918, -0.415) | 0.002 |
| Model 3 | -0.840 (-1.273, -0.407) | <0.001 |  | -0.796 (-1.276, -0.316) | 0.001 |  | -1.100 (-1.860, -0.339) | 0.005 |
| Model 4 | -1.063 (-1.766, -0.360) | 0.003 |  | -0.499 (-1.281, 0.283) | 0.211 |  | -0.899 (-2.195, 0.397) | 0.174 |
| Model 5 | -0.996 (-1.695, -0.297) | 0.005 |  | -0.429 (-1.209, 0.352) | 0.281 |  | -0.831 (-2.126, 0.464) | 0.208 |
| LBGI | | | | | | | | |
| Model 1 | 0.075 (-1.100, 1.250) | 0.900 |  | 0.355 (-0.963, 1.672) | 0.597 |  | 1.967 (-0.031, 3.965) | 0.054 |
| Model 2 | 0.349 (-0.827, 1.524) | 0.560 |  | 0.678 (-0.629, 1.984) | 0.309 |  | 2.065 (0.065, 4.065) | 0.043 |
| Model 3 | 0.394 (-0.763, 1.551) | 0.504 |  | 0.743 (-0.567, 2.053) | 0.266 |  | 2.149 (0.143, 4.156) | 0.036 |
| Model 4 | -0.400 (-1.644, 0.845) | 0.528 |  | 0.020 (-1.377, 1.417) | 0.978 |  | 1.747 (-0.481, 3.975) | 0.124 |
| Model 5 | -0.692 (-1.942, 0.559) | 0.278 |  | 0.131 (-1.276, 1.538) | 0.855 |  | 1.738 (-0.516, 3.991) | 0.130 |
| MODD (mmol/L) | | | | | | | | |
| Model 1 | -6.393 (-9.393, -3.393) | <0.001 |  | -6.556 (-9.847, -3.264) | <0.001 |  | -7.879 (-13.181, -2.578) | 0.004 |
| Model 2 | -6.541 (-9.510, -3.573) | <0.001 |  | -6.709 (-9.930, -3.487) | <0.001 |  | -8.135 (-13.420, -2.850) | 0.003 |
| Model 3 | -6.027 (-9.052, -3.001) | <0.001 |  | -6.531 (-9.870, -3.192) | <0.001 |  | -7.675 (-13.142, -2.207) | 0.006 |
| Model 4 | -6.706 (-10.788, -2.625) | 0.001 |  | -4.594 (-9.122, -0.067) | 0.047 |  | -4.867 (-12.573, 2.838) | 0.215 |
| Model 5 | -6.464 (-10.541, -2.387) | 0.002 |  | -4.013 (-8.551, 0.526) | 0.083 |  | -4.728 (-12.451, 2.994) | 0.229 |
| IQR (mmol/L) | | | | | | | | |
| Model 1 | -4.423 (-6.920, -1.926) | <0.001 |  | -4.205 (-6.952, -1.459) | 0.003 |  | -5.164 (-9.571, -0.756) | 0.022 |
| Model 2 | -4.750 (-7.224, -2.276) | <0.001 |  | -4.533 (-7.226, -1.841) | 0.001 |  | -5.462 (-9.871, -1.054) | 0.015 |
| Model 3 | -4.296 (-6.803, -1.788) | <0.001 |  | -4.311 (-7.091, -1.532) | 0.002 |  | -4.983 (-9.528, -0.439) | 0.032 |
| Model 4 | -4.315 (-7.683, -0.946) | 0.012 |  | -1.940 (-5.663, 1.783) | 0.306 |  | -1.892 (-8.201, 4.416) | 0.556 |
| Model 5 | -3.983 (-7.344, -0.621) | 0.020 |  | -1.521 (-5.247, 2.205) | 0.423 |  | -1.750 (-8.068, 4.568) | 0.586 |

Univariable and multivariable linear regression analysis. Model 1 was not adjusted. Model 2 was adjusted for age and sex. Model 3 was adjusted for BMI, duration of diabetes, and the covariates in Model 2. Model 4 was adjusted for smoking status, HbA1c, systolic blood pressure, total cholesterol, HDL cholesterol, log-transformed triglycerides, uric acid, and the covariates in Model 3. Model 5 was adjusted for eGFR, log-transformed urine albumin-to-creatinine ratio, and the covariates in Model 4.

GSM, gray-scale median; SD, standard deviation; CV, coefficient of variation; MAGE, mean amplitude of glycemic excursion; TIR, time in range; TAR, time above range; TBR, time below range; HBGI, high blood glucose index; LBGI, low blood glucose index; MODD, mean of daily differences; IQR, interquartile range

**Table S12 Associations of the metrics of continuous glucose monitoring with intima-media thickness among the** participants without dyslipidemia

|  | mean-IMT (n = 150) | |  | CCA-max-IMT (n = 150) | |
| --- | --- | --- | --- | --- | --- |
|  | β (95% CI) | *P* value |  | β (95% CI) | *P* value |
| Mean glucose (mmol/L) | | | | | |
| Model 1 | 0.002 (-0.010, 0.014) | 0.758 |  | 0.011 (-0.017, 0.039) | 0.449 |
| Model 2 | -0.001 (-0.011, 0.010) | 0.920 |  | 0.007 (-0.020, 0.034) | 0.603 |
| Model 3 | -0.001 (-0.011, 0.010) | 0.901 |  | 0.006 (-0.021, 0.033) | 0.649 |
| Model 4 | 0.003 (-0.015, 0.021) | 0.730 |  | 0.026 (-0.021, 0.074) | 0.268 |
| Model 5 | 0.006 (-0.013, 0.024) | 0.556 |  | 0.032 (-0.017, 0.080) | 0.198 |
| Median glucose (mmol/L) | | | | | |
| Model 1 | 0.016 (-0.019, 0.050) | 0.376 |  | 0.051 (-0.029, 0.132) | 0.212 |
| Model 2 | 0.004 (-0.025, 0.034) | 0.776 |  | 0.033 (-0.043, 0.109) | 0.390 |
| Model 3 | 0.005 (-0.025, 0.035) | 0.757 |  | 0.036 (-0.040, 0.113) | 0.351 |
| Model 4 | 0.008 (-0.028, 0.043) | 0.666 |  | 0.052 (-0.041, 0.145) | 0.271 |
| Model 5 | 0.006 (-0.030, 0.042) | 0.734 |  | 0.051 (-0.043, 0.145) | 0.286 |
| SD (mmol/L) | | | | | |
| Model 1 | 0.002 (-0.002, 0.005) | 0.329 |  | 0.004 (-0.004, 0.012) | 0.289 |
| Model 2 | 0.001 (-0.002, 0.004) | 0.596 |  | 0.003 (-0.005, 0.010) | 0.456 |
| Model 3 | 0.001 (-0.002, 0.004) | 0.560 |  | 0.003 (-0.004, 0.011) | 0.373 |
| Model 4 | 0.001 (-0.003, 0.004) | 0.725 |  | 0.003 (-0.005, 0.011) | 0.467 |
| Model 5 | 0.000 (-0.003, 0.003) | 0.883 |  | 0.003 (-0.006, 0.011) | 0.522 |
| CV (%) | | | | | |
| Model 1 | 0.004 (-0.008, 0.015) | 0.516 |  | 0.003 (-0.024, 0.029) | 0.846 |
| Model 2 | 0.002 (-0.008, 0.011) | 0.720 |  | -0.001 (-0.025, 0.024) | 0.964 |
| Model 3 | 0.002 (-0.008, 0.012) | 0.694 |  | 0.001 (-0.025, 0.026) | 0.961 |
| Model 4 | 0.002 (-0.008, 0.013) | 0.660 |  | 0.001 (-0.027, 0.030) | 0.924 |
| Model 5 | 0.002 (-0.008, 0.013) | 0.660 |  | 0.002 (-0.027, 0.030) | 0.906 |
| MAGE (mmol/L) | | | | | |
| Model 1 | 0.001 (-0.010, 0.013) | 0.821 |  | 0.009 (-0.018, 0.035) | 0.533 |
| Model 2 | -0.001 (-0.011, 0.009) | 0.826 |  | 0.005 (-0.021, 0.030) | 0.717 |
| Model 3 | -0.001 (-0.011, 0.009) | 0.804 |  | 0.004 (-0.022, 0.029) | 0.777 |
| Model 4 | 0.001 (-0.017, 0.018) | 0.933 |  | 0.019 (-0.027, 0.065) | 0.415 |
| Model 5 | 0.004 (-0.014, 0.022) | 0.694 |  | 0.025 (-0.023, 0.072) | 0.300 |
| TIR (%): 3.9–10.0 mmol/L | | | | | |
| Model 1 | -0.003 (-0.015, 0.009) | 0.649 |  | -0.016 (-0.044, 0.012) | 0.259 |
| Model 2 | 0.000 (-0.010, 0.010) | 0.995 |  | -0.012 (-0.038, 0.015) | 0.387 |
| Model 3 | 0.000 (-0.010, 0.011) | 0.976 |  | -0.011 (-0.037, 0.016) | 0.419 |
| Model 4 | -0.001 (-0.016, 0.014) | 0.916 |  | -0.025 (-0.064, 0.014) | 0.210 |
| Model 5 | -0.002 (-0.018, 0.013) | 0.776 |  | -0.029 (-0.070, 0.011) | 0.154 |
| TAR (%): >10.0 mmol/L | | | | | |
| Model 1 | 0.000 (-0.001, 0.001) | 0.687 |  | 0.001 (-0.001, 0.004) | 0.317 |
| Model 2 | 0.000 (-0.001, 0.001) | 0.944 |  | 0.001 (-0.002, 0.004) | 0.414 |
| Model 3 | 0.000 (-0.001, 0.001) | 0.972 |  | 0.001 (-0.002, 0.004) | 0.469 |
| Model 4 | 0.000 (-0.001, 0.002) | 0.629 |  | 0.003 (-0.001, 0.008) | 0.149 |
| Model 5 | 0.001 (-0.001, 0.002) | 0.506 |  | 0.004 (-0.001, 0.008) | 0.117 |
| TAR (%): >13.9 mmol/L | | | | | |
| Model 1 | -0.001 (-0.003, 0.002) | 0.472 |  | 0.000 (-0.006, 0.005) | 0.880 |
| Model 2 | -0.001 (-0.003, 0.001) | 0.186 |  | -0.001 (-0.006, 0.004) | 0.643 |
| Model 3 | -0.001 (-0.003, 0.001) | 0.200 |  | -0.001 (-0.006, 0.004) | 0.719 |
| Model 4 | -0.002 (-0.004, 0.001) | 0.281 |  | 0.000 (-0.008, 0.007) | 0.977 |
| Model 5 | -0.001 (-0.004, 0.002) | 0.332 |  | 0.000 (-0.007, 0.008) | 0.922 |
| TBR (%): <3.9 mmol/L | | | | | |
| Model 1 | 0.000 (-0.004, 0.004) | 0.866 |  | 0.002 (-0.007, 0.011) | 0.679 |
| Model 2 | 0.000 (-0.004, 0.003) | 0.798 |  | 0.001 (-0.008, 0.009) | 0.875 |
| Model 3 | 0.000 (-0.004, 0.003) | 0.829 |  | 0.001 (-0.008, 0.010) | 0.785 |
| Model 4 | -0.002 (-0.005, 0.002) | 0.420 |  | -0.001 (-0.010, 0.009) | 0.921 |
| Model 5 | -0.002 (-0.005, 0.002) | 0.430 |  | 0.000 (-0.010, 0.010) | 0.986 |
| TBR (%): <3.0 mmol/L | | | | | |
| Model 1 | -0.002 (-0.012, 0.009) | 0.778 |  | 0.002 (-0.023, 0.027) | 0.871 |
| Model 2 | -0.002 (-0.011, 0.007) | 0.667 |  | 0.001 (-0.022, 0.025) | 0.912 |
| Model 3 | -0.002 (-0.011, 0.007) | 0.684 |  | 0.002 (-0.022, 0.026) | 0.857 |
| Model 4 | -0.004 (-0.013, 0.006) | 0.464 |  | 0.000 (-0.026, 0.025) | 0.981 |
| Model 5 | -0.004 (-0.014, 0.006) | 0.459 |  | 0.002 (-0.025, 0.029) | 0.906 |
| HBGI | | | | | |
| Model 1 | 0.000 (-0.005, 0.004) | 0.955 |  | 0.003 (-0.008, 0.013) | 0.616 |
| Model 2 | -0.001 (-0.005, 0.003) | 0.511 |  | 0.001 (-0.009, 0.011) | 0.869 |
| Model 3 | -0.001 (-0.005, 0.003) | 0.520 |  | 0.001 (-0.009, 0.011) | 0.844 |
| Model 4 | -0.001 (-0.007, 0.005) | 0.763 |  | 0.005 (-0.010, 0.020) | 0.490 |
| Model 5 | -0.001 (-0.006, 0.005) | 0.843 |  | 0.006 (-0.009, 0.022) | 0.414 |
| LBGI | | | | | |
| Model 1 | -0.002 (-0.013, 0.009) | 0.659 |  | -0.003 (-0.029, 0.023) | 0.811 |
| Model 2 | -0.003 (-0.012, 0.007) | 0.575 |  | -0.004 (-0.028, 0.021) | 0.776 |
| Model 3 | -0.003 (-0.012, 0.007) | 0.603 |  | -0.002 (-0.027, 0.023) | 0.874 |
| Model 4 | -0.005 (-0.016, 0.006) | 0.383 |  | -0.005 (-0.033, 0.024) | 0.756 |
| Model 5 | -0.005 (-0.016, 0.006) | 0.349 |  | -0.004 (-0.033, 0.026) | 0.800 |
| MODD (mmol/L) | | | | | |
| Model 1 | -0.003 (-0.035, 0.029) | 0.854 |  | 0.005 (-0.070, 0.079) | 0.903 |
| Model 2 | 0.002 (-0.026, 0.029) | 0.899 |  | 0.012 (-0.058, 0.082) | 0.727 |
| Model 3 | 0.002 (-0.026, 0.030) | 0.905 |  | 0.012 (-0.059, 0.083) | 0.741 |
| Model 4 | 0.009 (-0.027, 0.045) | 0.610 |  | 0.030 (-0.065, 0.125) | 0.530 |
| Model 5 | 0.014 (-0.024, 0.051) | 0.479 |  | 0.041 (-0.058, 0.140) | 0.411 |
| IQR (mmol/L) | | | | | |
| Model 1 | -0.008 (-0.033, 0.017) | 0.536 |  | -0.008 (-0.066, 0.051) | 0.795 |
| Model 2 | -0.005 (-0.026, 0.017) | 0.648 |  | -0.003 (-0.058, 0.052) | 0.915 |
| Model 3 | -0.005 (-0.027, 0.017) | 0.655 |  | -0.002 (-0.058, 0.054) | 0.944 |
| Model 4 | 0.001 (-0.028, 0.031) | 0.929 |  | 0.010 (-0.067, 0.088) | 0.793 |
| Model 5 | 0.004 (-0.026, 0.035) | 0.785 |  | 0.018 (-0.062, 0.098) | 0.659 |

Univariable and multivariable linear regression analysis. Model 1 was not adjusted. Model 2 was adjusted for age and sex. Model 3 was adjusted for BMI, duration of diabetes, and the covariates in Model 2. Model 4 was adjusted for smoking status, HbA1c, systolic blood pressure, total cholesterol, HDL cholesterol, log-transformed triglycerides, uric acid, and the covariates in Model 3. Model 5 was adjusted for eGFR, log-transformed urine albumin-to-creatinine ratio, and the covariates in Model 4.

IMT, intima-media thickness; SD, standard deviation; CV, coefficient of variation; MAGE, mean amplitude of glycemic excursion; TIR, time in range; TAR, time above range; TBR, time below range; HBGI, high blood glucose index; LBGI, low blood glucose index; MODD, mean of daily differences; IQR, interquartile range

**Table S13 Associations of the metrics of continuous glucose monitoring with gray-scale median among participants without dyslipidemia**

|  | mean-GSM  (n = 150) | |  | thickened lesion-GSM  (n = 147) | |  | plaque-GSM  (n = 122) | |
| --- | --- | --- | --- | --- | --- | --- | --- | --- |
|  | β (95 % CI) | *P* value |  | β (95 % CI) | *P* value |  | β (95 % CI) | *P* value |
| Mean glucose (mmol/L) | | | | | | | | |
| Model 1 | -1.540 (-3.254, 0.174) | 0.078 |  | -1.292 (-2.704, 0.120) | 0.072 |  | -2.145 (-4.746, 0.457) | 0.105 |
| Model 2 | -1.711 (-3.422, 0.001) | 0.050 |  | -1.210 (-2.629, 0.209) | 0.094 |  | -2.050 (-4.683, 0.582) | 0.126 |
| Model 3 | -1.559 (-3.233, 0.116) | 0.068 |  | -1.139 (-2.560, 0.283) | 0.115 |  | -2.037 (-4.648, 0.575) | 0.125 |
| Model 4 | -2.439 (-5.142, 0.265) | 0.077 |  | -1.336 (-3.852, 1.180) | 0.295 |  | -1.483 (-6.169, 3.202) | 0.532 |
| Model 5 | -2.340 (-5.090, 0.410) | 0.095 |  | -1.403 (-3.968, 1.162) | 0.281 |  | -1.125 (-5.890, 3.640) | 0.641 |
| Median glucose (mmol/L) | | | | | | | | |
| Model 1 | -4.359 (-9.294, 0.576) | 0.083 |  | -3.893 (-7.947, 0.160) | 0.060 |  | -5.817 (-13.234, 1.601) | 0.123 |
| Model 2 | -4.327 (-9.256, 0.601) | 0.085 |  | -3.579 (-7.647, 0.488) | 0.084 |  | -5.857 (-13.353, 1.638) | 0.124 |
| Model 3 | -4.733 (-9.552, 0.086) | 0.054 |  | -3.708 (-7.796, 0.380) | 0.075 |  | -6.289 (-13.769, 1.191) | 0.099 |
| Model 4 | -5.073 (-10.407, 0.261) | 0.062 |  | -4.204 (-9.159, 0.750) | 0.096 |  | -3.155 (-12.170, 5.860) | 0.489 |
| Model 5 | -5.282 (-10.604, 0.040) | 0.052 |  | -4.380 (-9.348, 0.588) | 0.083 |  | -3.674 (-12.792, 5.444) | 0.426 |
| SD (mmol/L) | | | | | | | | |
| Model 1 | -0.197 (-0.681, 0.287) | 0.423 |  | -0.141 (-0.541, 0.259) | 0.487 |  | -0.147 (-0.897, 0.603) | 0.698 |
| Model 2 | -0.171 (-0.654, 0.312) | 0.485 |  | -0.117 (-0.517, 0.284) | 0.566 |  | -0.162 (-0.923, 0.598) | 0.673 |
| Model 3 | -0.254 (-0.728, 0.220) | 0.292 |  | -0.146 (-0.550, 0.259) | 0.478 |  | -0.203 (-0.967, 0.562) | 0.601 |
| Model 4 | -0.299 (-0.775, 0.178) | 0.217 |  | -0.240 (-0.684, 0.204) | 0.286 |  | -0.046 (-0.871, 0.778) | 0.911 |
| Model 5 | -0.350 (-0.830, 0.131) | 0.152 |  | -0.271 (-0.722, 0.179) | 0.236 |  | -0.124 (-0.971, 0.723) | 0.772 |
| CV (%) | | | | | | | | |
| Model 1 | -1.161 (-2.780, 0.458) | 0.159 |  | -0.520 (-1.872, 0.832) | 0.448 |  | -1.009 (-3.534, 1.516) | 0.430 |
| Model 2 | -1.146 (-2.757, 0.466) | 0.162 |  | -0.476 (-1.825, 0.873) | 0.487 |  | -1.035 (-3.579, 1.509) | 0.422 |
| Model 3 | -1.330 (-2.910, 0.249) | 0.098 |  | -0.518 (-1.879, 0.844) | 0.454 |  | -1.105 (-3.650, 1.440) | 0.392 |
| Model 4 | -1.345 (-2.963, 0.272) | 0.102 |  | -0.551 (-2.073, 0.971) | 0.475 |  | -0.330 (-3.144, 2.483) | 0.816 |
| Model 5 | -1.399 (-3.008, 0.211) | 0.088 |  | -0.623 (-2.145, 0.900) | 0.420 |  | -0.410 (-3.241, 2.421) | 0.774 |
| MAGE (mmol/L) | | | | | | | | |
| Model 1 | -1.321 (-2.968, 0.326) | 0.115 |  | -1.082 (-2.440, 0.277) | 0.118 |  | -1.893 (-4.383, 0.598) | 0.135 |
| Model 2 | -1.465 (-3.109, 0.179) | 0.080 |  | -0.998 (-2.363, 0.366) | 0.150 |  | -1.806 (-4.325, 0.712) | 0.158 |
| Model 3 | -1.290 (-2.901, 0.321) | 0.116 |  | -0.916 (-2.284, 0.452) | 0.188 |  | -1.740 (-4.241, 0.760) | 0.171 |
| Model 4 | -1.857 (-4.487, 0.773) | 0.165 |  | -0.755 (-3.201, 1.691) | 0.542 |  | -1.042 (-5.535, 3.450) | 0.646 |
| Model 5 | -1.715 (-4.424, 0.994) | 0.213 |  | -0.795 (-3.320, 1.730) | 0.534 |  | -0.553 (-5.173, 4.067) | 0.813 |
| TIR (%): 3.9–10.0 mmol/L | | | | | | | | |
| Model 1 | 1.476 (-0.222, 3.174) | 0.088 |  | 1.567 (0.179, 2.956) | 0.027 |  | 1.681 (-0.916, 4.278) | 0.202 |
| Model 2 | 1.526 (-0.165, 3.218) | 0.077 |  | 1.484 (0.094, 2.874) | 0.037 |  | 1.663 (-0.953, 4.278) | 0.211 |
| Model 3 | 1.380 (-0.284, 3.044) | 0.103 |  | 1.407 (0.006, 2.807) | 0.049 |  | 1.515 (-1.091, 4.121) | 0.252 |
| Model 4 | 1.804 (-0.460, 4.068) | 0.117 |  | 1.787 (-0.299, 3.873) | 0.092 |  | 0.330 (-3.585, 4.245) | 0.868 |
| Model 5 | 1.757 (-0.548, 4.061) | 0.134 |  | 1.928 (-0.200, 4.056) | 0.075 |  | 0.044 (-3.954, 4.042) | 0.983 |
| TAR (%): >10.0 mmol/L | | | | | | | | |
| Model 1 | -0.169 (-0.337, 0.000) | 0.050 |  | -0.168 (-0.306, -0.030) | 0.018 |  | -0.216 (-0.474, 0.043) | 0.102 |
| Model 2 | -0.180 (-0.348, -0.012) | 0.036 |  | -0.161 (-0.300, -0.023) | 0.023 |  | -0.209 (-0.470, 0.052) | 0.115 |
| Model 3 | -0.159 (-0.324, 0.006) | 0.059 |  | -0.152 (-0.291, -0.012) | 0.033 |  | -0.198 (-0.457, 0.062) | 0.134 |
| Model 4 | -0.244 (-0.495, 0.007) | 0.056 |  | -0.232 (-0.463, 0.000) | 0.050 |  | -0.132 (-0.572, 0.309) | 0.555 |
| Model 5 | -0.232 (-0.485, 0.022) | 0.073 |  | -0.235 (-0.469, -0.001) | 0.049 |  | -0.108 (-0.553, 0.336) | 0.630 |
| TAR (%): >13.9 mmol/L | | | | | | | | |
| Model 1 | -0.165 (-0.498, 0.169) | 0.331 |  | -0.166 (-0.439, 0.107) | 0.230 |  | -0.268 (-0.751, 0.216) | 0.275 |
| Model 2 | -0.174 (-0.506, 0.158) | 0.303 |  | -0.150 (-0.423, 0.123) | 0.279 |  | -0.260 (-0.747, 0.227) | 0.293 |
| Model 3 | -0.219 (-0.543, 0.105) | 0.184 |  | -0.169 (-0.442, 0.105) | 0.225 |  | -0.318 (-0.804, 0.167) | 0.197 |
| Model 4 | -0.259 (-0.691, 0.173) | 0.237 |  | -0.120 (-0.521, 0.281) | 0.555 |  | -0.008 (-0.697, 0.681) | 0.982 |
| Model 5 | -0.260 (-0.697, 0.177) | 0.241 |  | -0.144 (-0.551, 0.264) | 0.487 |  | 0.029 (-0.671, 0.729) | 0.935 |
| TBR (%): <3.9 mmol/L | | | | | | | | |
| Model 1 | 0.239 (-0.327, 0.805) | 0.405 |  | 0.131 (-0.333, 0.596) | 0.577 |  | 0.535 (-0.343, 1.413) | 0.230 |
| Model 2 | 0.309 (-0.257, 0.874) | 0.283 |  | 0.147 (-0.319, 0.613) | 0.533 |  | 0.498 (-0.402, 1.399) | 0.275 |
| Model 3 | 0.245 (-0.313, 0.802) | 0.388 |  | 0.133 (-0.338, 0.604) | 0.578 |  | 0.562 (-0.347, 1.470) | 0.223 |
| Model 4 | 0.099 (-0.463, 0.662) | 0.728 |  | 0.047 (-0.473, 0.567) | 0.858 |  | 0.429 (-0.531, 1.389) | 0.377 |
| Model 5 | 0.089 (-0.490, 0.667) | 0.762 |  | 0.001 (-0.536, 0.538) | 0.997 |  | 0.540 (-0.475, 1.555) | 0.294 |
| TBR (%): <3.0 mmol/L | | | | | | | | |
| Model 1 | -0.316 (-1.843, 1.210) | 0.683 |  | 0.649 (-0.598, 1.897) | 0.305 |  | 0.724 (-1.620, 3.069) | 0.542 |
| Model 2 | -0.121 (-1.652, 1.409) | 0.876 |  | 0.636 (-0.618, 1.890) | 0.318 |  | 0.559 (-1.845, 2.963) | 0.646 |
| Model 3 | -0.220 (-1.726, 1.285) | 0.773 |  | 0.643 (-0.621, 1.908) | 0.316 |  | 0.806 (-1.621, 3.233) | 0.512 |
| Model 4 | -0.595 (-2.064, 0.874) | 0.425 |  | 0.443 (-0.916, 1.802) | 0.520 |  | 0.363 (-2.125, 2.852) | 0.773 |
| Model 5 | -0.743 (-2.284, 0.797) | 0.342 |  | 0.315 (-1.117, 1.748) | 0.664 |  | 0.583 (-2.123, 3.289) | 0.670 |
| HBGI | | | | | | | | |
| Model 1 | -0.488 (-1.123, 0.147) | 0.131 |  | -0.393 (-0.914, 0.128) | 0.139 |  | -0.537 (-1.474, 0.400) | 0.259 |
| Model 2 | -0.517 (-1.150, 0.116) | 0.109 |  | -0.355 (-0.877, 0.167) | 0.181 |  | -0.516 (-1.462, 0.430) | 0.282 |
| Model 3 | -0.538 (-1.155, 0.079) | 0.087 |  | -0.360 (-0.882, 0.162) | 0.175 |  | -0.561 (-1.501, 0.378) | 0.239 |
| Model 4 | -0.715 (-1.575, 0.146) | 0.103 |  | -0.322 (-1.125, 0.481) | 0.428 |  | 0.093 (-1.316, 1.502) | 0.896 |
| Model 5 | -0.728 (-1.597, 0.141) | 0.100 |  | -0.377 (-1.192, 0.438) | 0.362 |  | 0.159 (-1.275, 1.593) | 0.826 |
| LBGI | | | | | | | | |
| Model 1 | 0.590 (-0.987, 2.168) | 0.461 |  | 0.844 (-0.444, 2.132) | 0.197 |  | 1.746 (-0.665, 4.156) | 0.154 |
| Model 2 | 0.781 (-0.794, 2.357) | 0.329 |  | 0.830 (-0.464, 2.123) | 0.207 |  | 1.628 (-0.836, 4.092) | 0.193 |
| Model 3 | 0.589 (-0.975, 2.154) | 0.458 |  | 0.819 (-0.498, 2.137) | 0.221 |  | 1.906 (-0.607, 4.418) | 0.136 |
| Model 4 | 0.014 (-1.625, 1.652) | 0.987 |  | 0.496 (-1.018, 2.010) | 0.518 |  | 1.740 (-1.014, 4.494) | 0.213 |
| Model 5 | -0.084 (-1.759, 1.590) | 0.921 |  | 0.374 (-1.184, 1.932) | 0.636 |  | 1.973 (-0.918, 4.864) | 0.179 |
| MODD (mmol/L) | | | | | | | | |
| Model 1 | -3.228 (-7.771, 1.316) | 0.162 |  | -1.914 (-5.671, 1.844) | 0.316 |  | -6.473 (-13.329, 0.382) | 0.064 |
| Model 2 | -3.687 (-8.211, 0.837) | 0.109 |  | -1.989 (-5.746, 1.768) | 0.297 |  | -6.400 (-13.303, 0.504) | 0.069 |
| Model 3 | -3.527 (-7.995, 0.940) | 0.121 |  | -1.841 (-5.643, 1.961) | 0.340 |  | -6.134 (-13.067, 0.798) | 0.082 |
| Model 4 | -5.361 (-10.781, 0.058) | 0.052 |  | -1.810 (-6.899, 3.278) | 0.483 |  | -5.032 (-14.318, 4.254) | 0.285 |
| Model 5 | -5.417 (-11.013, 0.179) | 0.058 |  | -2.141 (-7.409, 3.127) | 0.423 |  | -4.327 (-13.957, 5.304) | 0.375 |
| IQR (mmol/L) | | | | | | | | |
| Model 1 | -2.057 (-5.634, 1.521) | 0.258 |  | -1.586 (-4.531, 1.359) | 0.289 |  | -6.032 (-11.360, -0.704) | 0.027 |
| Model 2 | -2.460 (-6.027, 1.106) | 0.175 |  | -1.619 (-4.567, 1.329) | 0.280 |  | -5.950 (-11.321, -0.578) | 0.030 |
| Model 3 | -2.602 (-6.123, 0.919) | 0.146 |  | -1.610 (-4.597, 1.377) | 0.288 |  | -5.921 (-11.324, -0.518) | 0.032 |
| Model 4 | -4.120 (-8.539, 0.299) | 0.067 |  | -1.753 (-5.894, 2.387) | 0.404 |  | -5.697 (-13.317, 1.923) | 0.141 |
| Model 5 | -4.190 (-8.718, 0.337) | 0.069 |  | -2.076 (-6.333, 2.180) | 0.336 |  | -5.107 (-13.049, 2.835) | 0.205 |

Univariable and multivariable linear regression analysis. Model 1 was not adjusted. Model 2 was adjusted for age and sex. Model 3 was adjusted for BMI, duration of diabetes, and the covariates in Model 2. Model 4 was adjusted for smoking status, HbA1c, systolic blood pressure, total cholesterol, HDL cholesterol, log-transformed triglycerides, uric acid, and the covariates in Model 3. Model 5 was adjusted for eGFR, log-transformed urine albumin-to-creatinine ratio, and the covariates in Model 4.

GSM, gray-scale median; SD, standard deviation; CV, coefficient of variation; MAGE, mean amplitude of glycemic excursion; TIR, time in range; TAR, time above range; TBR, time below range; HBGI, high blood glucose index; LBGI, low blood glucose index; MODD, mean of daily differences; IQR, interquartile range

**Table S14 Associations of the metrics of continuous glucose monitoring with intima-media thickness among participants using anti-diabetic medications**

|  | mean-IMT (n = 550) | |  | CCA-max-IMT (n = 550) | |
| --- | --- | --- | --- | --- | --- |
|  | β (95% CI) | *P* value |  | β (95% CI) | *P* value |
| Mean glucose (mmol/L) | | | | | |
| Model 1 | -0.003 (-0.010, 0.005) | 0.526 |  | 0.002 (-0.020, 0.025) | 0.847 |
| Model 2 | -0.003 (-0.010, 0.004) | 0.397 |  | 0.001 (-0.021, 0.023) | 0.918 |
| Model 3 | -0.003 (-0.010, 0.004) | 0.411 |  | -0.002 (-0.024, 0.021) | 0.891 |
| Model 4 | 0.003 (-0.008, 0.015) | 0.578 |  | 0.010 (-0.025, 0.045) | 0.568 |
| Model 5 | 0.006 (-0.006, 0.017) | 0.365 |  | 0.014 (-0.022, 0.050) | 0.445 |
| Median glucose (mmol/L) | | | | | |
| Model 1 | 0.001 (-0.021, 0.022) | 0.963 |  | 0.035 (-0.028, 0.098) | 0.280 |
| Model 2 | -0.010 (-0.030, 0.011) | 0.358 |  | 0.015 (-0.047, 0.077) | 0.640 |
| Model 3 | -0.009 (-0.030, 0.012) | 0.381 |  | 0.017 (-0.046, 0.079) | 0.605 |
| Model 4 | -0.001 (-0.026, 0.023) | 0.909 |  | 0.046 (-0.029, 0.120) | 0.227 |
| Model 5 | 0.001 (-0.024, 0.026) | 0.952 |  | 0.049 (-0.027, 0.125) | 0.202 |
| SD (mmol/L) | | | | | |
| Model 1 | 0.001 (-0.001, 0.003) | 0.335 |  | 0.005 (-0.001, 0.012) | 0.121 |
| Model 2 | 0.000 (-0.002, 0.002) | 0.848 |  | 0.003 (-0.004, 0.009) | 0.437 |
| Model 3 | 0.000 (-0.002, 0.002) | 0.890 |  | 0.004 (-0.003, 0.010) | 0.269 |
| Model 4 | 0.000 (-0.002, 0.002) | 0.807 |  | 0.004 (-0.002, 0.011) | 0.213 |
| Model 5 | 0.000 (-0.002, 0.002) | 0.824 |  | 0.004 (-0.003, 0.011) | 0.219 |
| CV (%) | | | | | |
| Model 1 | 0.001 (-0.005, 0.008) | 0.692 |  | 0.012 (-0.007, 0.031) | 0.219 |
| Model 2 | -0.002 (-0.008, 0.004) | 0.577 |  | 0.006 (-0.013, 0.025) | 0.533 |
| Model 3 | -0.002 (-0.008, 0.005) | 0.601 |  | 0.007 (-0.012, 0.026) | 0.442 |
| Model 4 | 0.001 (-0.007, 0.008) | 0.892 |  | 0.017 (-0.005, 0.038) | 0.121 |
| Model 5 | 0.001 (-0.006, 0.008) | 0.736 |  | 0.019 (-0.003, 0.040) | 0.094 |
| MAGE (mmol/L) | | | | | |
| Model 1 | -0.003 (-0.011, 0.004) | 0.421 |  | 0.000 (-0.022, 0.022) | 0.995 |
| Model 2 | -0.004 (-0.011, 0.004) | 0.333 |  | -0.001 (-0.022, 0.021) | 0.951 |
| Model 3 | -0.003 (-0.011, 0.004) | 0.345 |  | -0.004 (-0.026, 0.018) | 0.722 |
| Model 4 | 0.002 (-0.009, 0.014) | 0.717 |  | 0.003 (-0.032, 0.037) | 0.870 |
| Model 5 | 0.004 (-0.008, 0.016) | 0.488 |  | 0.006 (-0.029, 0.041) | 0.729 |
| TIR (%): 3.9–10.0 mmol/L | | | | | |
| Model 1 | 0.001 (-0.007, 0.008) | 0.866 |  | -0.007 (-0.029, 0.014) | 0.496 |
| Model 2 | 0.003 (-0.004, 0.009) | 0.475 |  | -0.004 (-0.025, 0.017) | 0.723 |
| Model 3 | 0.002 (-0.005, 0.009) | 0.497 |  | -0.001 (-0.022, 0.020) | 0.911 |
| Model 4 | -0.002 (-0.012, 0.008) | 0.703 |  | -0.012 (-0.042, 0.018) | 0.426 |
| Model 5 | -0.003 (-0.013, 0.007) | 0.565 |  | -0.014 (-0.044, 0.017) | 0.373 |
| TAR (%): >10.0 mmol/L | | | | | |
| Model 1 | 0.000 (-0.001, 0.001) | 0.536 |  | 0.000 (-0.002, 0.002) | 0.829 |
| Model 2 | 0.000 (-0.001, 0.000) | 0.329 |  | 0.000 (-0.002, 0.002) | 0.981 |
| Model 3 | 0.000 (-0.001, 0.000) | 0.342 |  | 0.000 (-0.002, 0.002) | 0.805 |
| Model 4 | 0.000 (-0.001, 0.001) | 0.813 |  | 0.001 (-0.003, 0.004) | 0.679 |
| Model 5 | 0.000 (-0.001, 0.001) | 0.608 |  | 0.001 (-0.002, 0.004) | 0.582 |
| TAR (%): >13.9 mmol/L | | | | | |
| Model 1 | -0.001 (-0.003, 0.001) | 0.167 |  | 0.000 (-0.005, 0.004) | 0.878 |
| Model 2 | -0.001 (-0.003, 0.000) | 0.103 |  | -0.001 (-0.005, 0.004) | 0.800 |
| Model 3 | -0.001 (-0.003, 0.000) | 0.106 |  | -0.001 (-0.005, 0.004) | 0.724 |
| Model 4 | -0.001 (-0.003, 0.001) | 0.515 |  | 0.001 (-0.006, 0.007) | 0.852 |
| Model 5 | -0.001 (-0.003, 0.002) | 0.575 |  | 0.001 (-0.006, 0.007) | 0.793 |
| TBR (%): <3.9 mmol/L | | | | | |
| Model 1 | 0.003 (0.000, 0.005) | 0.074 |  | 0.007 (-0.001, 0.016) | 0.077 |
| Model 2 | 0.002 (-0.001, 0.004) | 0.272 |  | 0.005 (-0.003, 0.013) | 0.202 |
| Model 3 | 0.002 (-0.001, 0.004) | 0.257 |  | 0.006 (-0.002, 0.014) | 0.160 |
| Model 4 | 0.001 (-0.002, 0.003) | 0.678 |  | 0.005 (-0.004, 0.013) | 0.281 |
| Model 5 | 0.000 (-0.002, 0.003) | 0.822 |  | 0.004 (-0.004, 0.013) | 0.324 |
| TBR (%): <3.0 mmol/L | | | | | |
| Model 1 | 0.007 (-0.003, 0.015) | 0.157 |  | 0.024 (-0.003, 0.050) | 0.075 |
| Model 2 | 0.005 (-0.004, 0.013) | 0.270 |  | 0.020 (-0.005, 0.046) | 0.121 |
| Model 3 | 0.005 (-0.004, 0.013) | 0.259 |  | 0.021 (-0.005, 0.047) | 0.107 |
| Model 4 | 0.004 (-0.005, 0.012) | 0.396 |  | 0.022 (-0.004, 0.048) | 0.091 |
| Model 5 | 0.003 (-0.006, 0.012) | 0.471 |  | 0.021 (-0.005, 0.047) | 0.114 |
| HBGI | | | | | |
| Model 1 | -0.001 (-0.004, 0.002) | 0.385 |  | 0.001 (-0.008, 0.010) | 0.781 |
| Model 2 | -0.002 (-0.005, 0.001) | 0.184 |  | 0.000 (-0.009, 0.009) | 0.980 |
| Model 3 | -0.002 (-0.005, 0.001) | 0.193 |  | 0.000 (-0.009, 0.008) | 0.929 |
| Model 4 | 0.000 (-0.005, 0.004) | 0.873 |  | 0.005 (-0.008, 0.018) | 0.448 |
| Model 5 | 0.000 (-0.004, 0.004) | 0.966 |  | 0.006 (-0.007, 0.019) | 0.388 |
| LBGI | | | | | |
| Model 1 | 0.002 (-0.005, 0.010) | 0.552 |  | 0.006 (-0.016, 0.029) | 0.578 |
| Model 2 | 0.000 (-0.007, 0.007) | 0.986 |  | 0.002 (-0.021, 0.024) | 0.887 |
| Model 3 | 0.000 (-0.007, 0.008) | 0.959 |  | 0.004 (-0.019, 0.026) | 0.761 |
| Model 4 | -0.002 (-0.010, 0.006) | 0.552 |  | 0.002 (-0.022, 0.025) | 0.886 |
| Model 5 | -0.004 (-0.012, 0.005) | 0.396 |  | 0.000 (-0.024, 0.024) | 0.998 |
| MODD (mmol/L) | | | | | |
| Model 1 | -0.010 (-0.030, 0.011) | 0.364 |  | -0.008 (-0.070, 0.053) | 0.791 |
| Model 2 | -0.008 (-0.027, 0.012) | 0.445 |  | -0.004 (-0.064, 0.056) | 0.898 |
| Model 3 | -0.007 (-0.027, 0.013) | 0.475 |  | -0.007 (-0.068, 0.054) | 0.821 |
| Model 4 | 0.005 (-0.021, 0.031) | 0.690 |  | 0.011 (-0.068, 0.089) | 0.790 |
| Model 5 | 0.007 (-0.020, 0.034) | 0.604 |  | 0.012 (-0.069, 0.092) | 0.778 |
| IQR (mmol/L) | | | | | |
| Model 1 | -0.013 (-0.030, 0.004) | 0.140 |  | -0.018 (-0.067, 0.032) | 0.490 |
| Model 2 | -0.010 (-0.026, 0.006) | 0.206 |  | -0.012 (-0.061, 0.036) | 0.623 |
| Model 3 | -0.010 (-0.026, 0.006) | 0.220 |  | -0.014 (-0.063, 0.035) | 0.579 |
| Model 4 | -0.003 (-0.025, 0.018) | 0.778 |  | -0.008 (-0.072, 0.057) | 0.811 |
| Model 5 | -0.001 (-0.023, 0.021) | 0.912 |  | -0.007 (-0.073, 0.059) | 0.842 |

Univariable and multivariable linear regression analysis. Model 1 was not adjusted. Model 2 was adjusted for age and sex. Model 3 was adjusted for BMI, duration of diabetes, and the covariates in Model 2. Model 4 was adjusted for smoking status, HbA1c, systolic blood pressure, total cholesterol, HDL cholesterol, log-transformed triglycerides, uric acid, and the covariates in Model 3. Model 5 was adjusted for eGFR, log-transformed urine albumin-to-creatinine ratio, and the covariates in Model 4.

IMT, intima-media thickness; SD, standard deviation; CV, coefficient of variation; MAGE, mean amplitude of glycemic excursion; TIR, time in range; TAR, time above range; TBR, time below range; HBGI, high blood glucose index; LBGI, low blood glucose index; MODD, mean of daily differences; IQR, interquartile range

**Table S15 Associations of the metrics of continuous glucose monitoring with gray-scale median among participants using anti-diabetic medications**

|  | mean-GSM  (n = 549) | |  | thickened lesion-GSM  (n = 524) | |  | plaque-GSM  (n = 455) | |
| --- | --- | --- | --- | --- | --- | --- | --- | --- |
|  | β (95 % CI) | *P* value |  | β (95 % CI) | *P* value |  | β (95 % CI) | *P* value |
| Mean glucose (mmol/L) | | | | | | | | |
| Model 1 | -2.107 (-3.060, -1.154) | <0.001 |  | -1.967 (-2.937, -0.998) | <0.001 |  | -3.086 (-4.654, -1.518) | <0.001 |
| Model 2 | -2.242 (-3.187, -1.296) | <0.001 |  | -1.899 (-2.858, -0.941) | <0.001 |  | -2.949 (-4.514, -1.383) | <0.001 |
| Model 3 | -1.995 (-2.930, -1.061) | <0.001 |  | -1.817 (-2.784, -0.851) | <0.001 |  | -2.793 (-4.364, -1.222) | <0.001 |
| Model 4 | -2.297 (-3.805, -0.790) | 0.003 |  | -1.238 (-2.820, 0.344) | 0.125 |  | -2.559 (-5.243, 0.124) | 0.062 |
| Model 5 | -2.046 (-3.563, -0.530) | 0.008 |  | -1.103 (-2.696, 0.490) | 0.174 |  | -2.324 (-5.024, 0.377) | 0.092 |
| Median glucose (mmol/L) | | | | | | | | |
| Model 1 | -6.804 (-9.453, -4.155) | <0.001 |  | -5.886 (-8.582, -3.190) | <0.001 |  | -7.991 (-12.484, -3.498) | <0.001 |
| Model 2 | -6.480 (-9.128, -3.833) | <0.001 |  | -5.286 (-7.959, -2.613) | <0.001 |  | -7.822 (-12.310, -3.335) | <0.001 |
| Model 3 | -6.257 (-8.901, -3.613) | <0.001 |  | -5.078 (-7.807, -2.350) | <0.001 |  | -7.407 (-11.973, -2.842) | 0.002 |
| Model 4 | -6.995 (-10.169, -3.821) | <0.001 |  | -4.086 (-7.426, -0.746) | 0.017 |  | -5.344 (-10.997, 0.309) | 0.064 |
| Model 5 | -6.663 (-9.838, -3.488) | <0.001 |  | -3.655 (-7.000, -0.310) | 0.032 |  | -5.187 (-10.853, 0.479) | 0.073 |
| SD (mmol/L) | | | | | | | | |
| Model 1 | -0.408 (-0.683, -0.133) | 0.004 |  | -0.287 (-0.568, -0.006) | 0.046 |  | -0.250 (-0.720, 0.220) | 0.297 |
| Model 2 | -0.341 (-0.618, -0.064) | 0.016 |  | -0.215 (-0.496, 0.066) | 0.133 |  | -0.249 (-0.722, 0.225) | 0.303 |
| Model 3 | -0.370 (-0.647, -0.092) | 0.009 |  | -0.196 (-0.484, 0.092) | 0.181 |  | -0.214 (-0.699, 0.272) | 0.388 |
| Model 4 | -0.471 (-0.751, -0.192) | <0.001 |  | -0.233 (-0.527, 0.061) | 0.120 |  | -0.220 (-0.720, 0.280) | 0.387 |
| Model 5 | -0.468 (-0.748, -0.189) | 0.001 |  | -0.203 (-0.497, 0.091) | 0.176 |  | -0.225 (-0.726, 0.277) | 0.379 |
| CV (%) | | | | | | | | |
| Model 1 | -1.966 (-2.774, -1.158) | <0.001 |  | -1.639 (-2.459, -0.819) | <0.001 |  | -1.856 (-3.249, -0.464) | 0.009 |
| Model 2 | -1.926 (-2.733, -1.120) | <0.001 |  | -1.442 (-2.255, -0.630) | <0.001 |  | -1.708 (-3.099, -0.317) | 0.016 |
| Model 3 | -1.938 (-2.733, -1.142) | <0.001 |  | -1.400 (-2.221, -0.580) | <0.001 |  | -1.639 (-3.040, -0.238) | 0.022 |
| Model 4 | -2.024 (-2.935, -1.113) | <0.001 |  | -1.084 (-2.042, -0.125) | 0.027 |  | -0.885 (-2.526, 0.755) | 0.289 |
| Model 5 | -1.963 (-2.876, -1.051) | <0.001 |  | -0.957 (-1.919, 0.006) | 0.051 |  | -0.833 (-2.480, 0.815) | 0.321 |
| MAGE (mmol/L) | | | | | | | | |
| Model 1 | -1.948 (-2.881, -1.014) | <0.001 |  | -1.832 (-2.783, -0.880) | <0.001 |  | -2.883 (-4.416, -1.351) | <0.001 |
| Model 2 | -2.088 (-3.014, -1.162) | <0.001 |  | -1.777 (-2.717, -0.837) | <0.001 |  | -2.751 (-4.282, -1.221) | <0.001 |
| Model 3 | -1.809 (-2.727, -0.891) | <0.001 |  | -1.692 (-2.642, -0.743) | <0.001 |  | -2.581 (-4.119, -1.043) | 0.001 |
| Model 4 | -1.796 (-3.276, -0.316) | 0.017 |  | -0.910 (-2.464, 0.643) | 0.250 |  | -1.954 (-4.571, 0.663) | 0.143 |
| Model 5 | -1.580 (-3.068, -0.092) | 0.037 |  | -0.808 (-2.372, 0.755) | 0.310 |  | -1.754 (-4.386, 0.878) | 0.191 |
| TIR (%): 3.9–10.0 mmol/L | | | | | | | | |
| Model 1 | 2.170 (1.275, 3.064) | <0.001 |  | 2.012 (1.096, 2.928) | <0.001 |  | 2.514 (1.009, 4.019) | 0.001 |
| Model 2 | 2.192 (1.304, 3.080) | <0.001 |  | 1.865 (0.960, 2.770) | <0.001 |  | 2.381 (0.880, 3.881) | 0.002 |
| Model 3 | 1.935 (1.053, 2.818) | <0.001 |  | 1.776 (0.859, 2.693) | <0.001 |  | 2.188 (0.676, 3.700) | 0.005 |
| Model 4 | 2.325 (1.036, 3.615) | <0.001 |  | 1.478 (0.120, 2.837) | 0.033 |  | 1.294 (-0.984, 3.572) | 0.265 |
| Model 5 | 2.217 (0.931, 3.503) | <0.001 |  | 1.291 (-0.068, 2.649) | 0.063 |  | 1.129 (-1.148, 3.406) | 0.330 |
| TAR (%): >10.0 mmol/L | | | | | | | | |
| Model 1 | -0.215 (-0.302, -0.127) | <0.001 |  | -0.200 (-0.289, -0.110) | <0.001 |  | -0.278 (-0.424, -0.132) | <0.001 |
| Model 2 | -0.221 (-0.308, -0.135) | <0.001 |  | -0.190 (-0.278, -0.101) | <0.001 |  | -0.266 (-0.412, -0.120) | <0.001 |
| Model 3 | -0.196 (-0.282, -0.110) | <0.001 |  | -0.182 (-0.271, -0.092) | <0.001 |  | -0.250 (-0.396, -0.103) | <0.001 |
| Model 4 | -0.249 (-0.383, -0.115) | <0.001 |  | -0.161 (-0.303, -0.020) | 0.025 |  | -0.214 (-0.454, 0.025) | 0.079 |
| Model 5 | -0.227 (-0.361, -0.094) | <0.001 |  | -0.145 (-0.286, -0.003) | 0.045 |  | -0.194 (-0.433, 0.045) | 0.111 |
| TAR (%): >13.9 mmol/L | | | | | | | | |
| Model 1 | -0.299 (-0.497, -0.101) | 0.003 |  | -0.286 (-0.486, -0.086) | 0.005 |  | -0.449 (-0.766, -0.133) | 0.005 |
| Model 2 | -0.313 (-0.509, -0.116) | 0.002 |  | -0.274 (-0.471, -0.077) | 0.006 |  | -0.430 (-0.745, -0.115) | 0.008 |
| Model 3 | -0.289 (-0.482, -0.097) | 0.003 |  | -0.265 (-0.462, -0.068) | 0.008 |  | -0.419 (-0.733, -0.105) | 0.009 |
| Model 4 | -0.242 (-0.515, 0.030) | 0.082 |  | -0.101 (-0.385, 0.182) | 0.483 |  | -0.151 (-0.612, 0.310) | 0.519 |
| Model 5 | -0.243 (-0.513, 0.028) | 0.079 |  | -0.088 (-0.370, 0.194) | 0.540 |  | -0.134 (-0.592, 0.325) | 0.567 |
| TBR (%): <3.9 mmol/L | | | | | | | | |
| Model 1 | 0.117 (-0.234, 0.467) | 0.514 |  | 0.110 (-0.245, 0.465) | 0.543 |  | 0.578 (0.007, 1.149) | 0.047 |
| Model 2 | 0.188 (-0.162, 0.537) | 0.292 |  | 0.174 (-0.176, 0.525) | 0.329 |  | 0.592 (0.021, 1.163) | 0.042 |
| Model 3 | 0.193 (-0.150, 0.537) | 0.270 |  | 0.192 (-0.160, 0.544) | 0.284 |  | 0.638 (0.065, 1.210) | 0.029 |
| Model 4 | -0.017 (-0.371, 0.336) | 0.923 |  | 0.006 (-0.360, 0.373) | 0.973 |  | 0.450 (-0.153, 1.053) | 0.143 |
| Model 5 | -0.086 (-0.442, 0.270) | 0.634 |  | 0.032 (-0.338, 0.402) | 0.864 |  | 0.456 (-0.156, 1.068) | 0.144 |
| TBR (%): <3.0 mmol/L | | | | | | | | |
| Model 1 | -0.661 (-1.795, 0.472) | 0.252 |  | 0.083 (-1.057, 1.223) | 0.886 |  | 0.622 (-1.220, 2.463) | 0.507 |
| Model 2 | -0.510 (-1.637, 0.617) | 0.374 |  | 0.173 (-0.949, 1.296) | 0.762 |  | 0.565 (-1.272, 2.402) | 0.546 |
| Model 3 | -0.462 (-1.568, 0.643) | 0.412 |  | 0.225 (-0.900, 1.349) | 0.694 |  | 0.719 (-1.119, 2.557) | 0.442 |
| Model 4 | -0.950 (-2.060, 0.161) | 0.093 |  | -0.182 (-1.329, 0.964) | 0.755 |  | 0.255 (-1.634, 2.144) | 0.791 |
| Model 5 | -1.017 (-2.132, 0.097) | 0.074 |  | -0.055 (-1.208, 1.098) | 0.926 |  | 0.250 (-1.657, 2.157) | 0.797 |
| HBGI | | | | | | | | |
| Model 1 | -0.846 (-1.216, -0.475) | <0.001 |  | -0.740 (-1.116, -0.364) | <0.001 |  | -1.084 (-1.688, -0.480) | <0.001 |
| Model 2 | -0.853 (-1.220, -0.486) | <0.001 |  | -0.694 (-1.065, -0.323) | <0.001 |  | -1.043 (-1.645, -0.441) | <0.001 |
| Model 3 | -0.787 (-1.149, -0.425) | <0.001 |  | -0.664 (-1.037, -0.291) | <0.001 |  | -0.992 (-1.595, -0.389) | 0.001 |
| Model 4 | -1.008 (-1.564, -0.452) | <0.001 |  | -0.494 (-1.078, 0.090) | 0.097 |  | -0.730 (-1.700, 0.240) | 0.140 |
| Model 5 | -0.966 (-1.521, -0.411) | <0.001 |  | -0.431 (-1.015, 0.153) | 0.148 |  | -0.669 (-1.638, 0.300) | 0.176 |
| LBGI | | | | | | | | |
| Model 1 | 0.335 (-0.642, 1.311) | 0.501 |  | 0.691 (-0.306, 1.687) | 0.174 |  | 1.712 (0.108, 3.316) | 0.037 |
| Model 2 | 0.578 (-0.398, 1.553) | 0.245 |  | 0.814 (-0.173, 1.801) | 0.106 |  | 1.634 (0.026, 3.242) | 0.046 |
| Model 3 | 0.574 (-0.388, 1.535) | 0.242 |  | 0.875 (-0.119, 1.868) | 0.084 |  | 1.798 (0.179, 3.416) | 0.030 |
| Model 4 | -0.223 (-1.245, 0.800) | 0.669 |  | 0.247 (-0.826, 1.320) | 0.651 |  | 1.279 (-0.495, 3.053) | 0.157 |
| Model 5 | -0.422 (-1.455, 0.610) | 0.422 |  | 0.346 (-0.739, 1.431) | 0.531 |  | 1.277 (-0.525, 3.080) | 0.164 |
| MODD (mmol/L) | | | | | | | | |
| Model 1 | -5.845 (-8.434, -3.256) | <0.001 |  | -5.237 (-7.864, -2.610) | <0.001 |  | -7.989 (-12.332, -3.646) | <0.001 |
| Model 2 | -6.154 (-8.717, -3.591) | <0.001 |  | -5.359 (-7.941, -2.777) | <0.001 |  | -8.029 (-12.352, -3.707) | <0.001 |
| Model 3 | -5.648 (-8.221, -3.076) | <0.001 |  | -5.118 (-7.763, -2.473) | <0.001 |  | -7.463 (-11.872, -3.053) | <0.001 |
| Model 4 | -6.934 (-10.284, -3.585) | <0.001 |  | -3.997 (-7.527, -0.468) | 0.027 |  | -5.436 (-11.460, 0.588) | 0.077 |
| Model 5 | -6.882 (-10.260, -3.503) | <0.001 |  | -3.473 (-7.045, 0.099) | 0.057 |  | -5.293 (-11.380, 0.794) | 0.088 |
| IQR (mmol/L) | | | | | | | | |
| Model 1 | -4.075 (-6.183, -1.967) | <0.001 |  | -3.473 (-5.616, -1.331) | 0.002 |  | -5.891 (-9.416, -2.367) | 0.001 |
| Model 2 | -4.431 (-6.521, -2.341) | <0.001 |  | -3.639 (-5.749, -1.529) | <0.001 |  | -5.918 (-9.435, -2.402) | 0.001 |
| Model 3 | -4.076 (-6.168, -1.985) | <0.001 |  | -3.427 (-5.583, -1.272) | 0.002 |  | -5.465 (-9.044, -1.886) | 0.003 |
| Model 4 | -4.827 (-7.586, -2.068) | <0.001 |  | -2.100 (-5.003, 0.803) | 0.156 |  | -3.464 (-8.423, 1.495) | 0.170 |
| Model 5 | -4.703 (-7.478, -1.928) | <0.001 |  | -1.715 (-4.641, 1.211) | 0.250 |  | -3.344 (-8.346, 1.657) | 0.189 |

Univariable and multivariable linear regression analysis. Model 1 was not adjusted. Model 2 was adjusted for age and sex. Model 3 was adjusted for BMI, duration of diabetes, and the covariates in Model 2. Model 4 was adjusted for smoking status, HbA1c, systolic blood pressure, total cholesterol, HDL cholesterol, log-transformed triglycerides, uric acid, and the covariates in Model 3. Model 5 was adjusted for eGFR, log-transformed urine albumin-to-creatinine ratio, and the covariates in Model 4.

GSM, gray-scale median; SD, standard deviation; CV, coefficient of variation; MAGE, mean amplitude of glycemic excursion; TIR, time in range; TAR, time above range; TBR, time below range; HBGI, high blood glucose index; LBGI, low blood glucose index; MODD, mean of daily differences; IQR, interquartile range

**Table S16 Associations of the metrics of continuous glucose monitoring with intima-media thickness among participants not using anti-diabetic medications**

|  | mean-IMT (n = 50) | |  | CCA-max-IMT (n = 50) | |
| --- | --- | --- | --- | --- | --- |
|  | β (95% CI) | *P* value |  | β (95% CI) | *P* value |
| Mean glucose (mmol/L) | | | | | |
| Model 1 | 0.009 (-0.016, 0.035) | 0.476 |  | 0.014 (-0.029, 0.057) | 0.516 |
| Model 2 | 0.017 (-0.005, 0.039) | 0.132 |  | 0.025 (-0.015, 0.064) | 0.220 |
| Model 3 | 0.021 (-0.003, 0.046) | 0.087 |  | 0.036 (-0.007, 0.079) | 0.100 |
| Model 4 | 0.023 (-0.027, 0.073) | 0.361 |  | 0.053 (-0.045, 0.150) | 0.282 |
| Model 5 | 0.023 (-0.029, 0.075) | 0.374 |  | 0.054 (-0.047, 0.155) | 0.286 |
| Median glucose (mmol/L) | | | | | |
| Model 1 | 0.009 (-0.066, 0.084) | 0.807 |  | -0.003 (-0.129, 0.123) | 0.965 |
| Model 2 | 0.007 (-0.058, 0.072) | 0.828 |  | -0.005 (-0.122, 0.111) | 0.927 |
| Model 3 | 0.014 (-0.063, 0.091) | 0.712 |  | 0.009 (-0.126, 0.145) | 0.890 |
| Model 4 | -0.012 (-0.103, 0.078) | 0.782 |  | -0.023 (-0.201, 0.154) | 0.793 |
| Model 5 | -0.010 (-0.115, 0.096) | 0.852 |  | 0.009 (-0.198, 0.216) | 0.931 |
| SD (mmol/L) | | | | | |
| Model 1 | -0.001 (-0.009, 0.006) | 0.749 |  | -0.004 (-0.016, 0.009) | 0.567 |
| Model 2 | -0.004 (-0.010, 0.003) | 0.263 |  | -0.007 (-0.019, 0.005) | 0.230 |
| Model 3 | -0.004 (-0.011, 0.003) | 0.270 |  | -0.008 (-0.020, 0.004) | 0.203 |
| Model 4 | -0.003 (-0.010, 0.005) | 0.455 |  | -0.005 (-0.020, 0.010) | 0.479 |
| Model 5 | -0.004 (-0.013, 0.005) | 0.399 |  | -0.004 (-0.022, 0.014) | 0.652 |
| CV (%) | | | | | |
| Model 1 | 0.008 (-0.016, 0.031) | 0.507 |  | 0.009 (-0.030, 0.048) | 0.646 |
| Model 2 | 0.004 (-0.016, 0.025) | 0.676 |  | 0.005 (-0.032, 0.041) | 0.807 |
| Model 3 | 0.006 (-0.017, 0.028) | 0.603 |  | 0.008 (-0.032, 0.048) | 0.681 |
| Model 4 | 0.005 (-0.020, 0.030) | 0.691 |  | 0.012 (-0.037, 0.060) | 0.633 |
| Model 5 | 0.008 (-0.020, 0.035) | 0.573 |  | 0.023 (-0.031, 0.077) | 0.398 |
| MAGE (mmol/L) | | | | | |
| Model 1 | 0.012 (-0.014, 0.038) | 0.357 |  | 0.019 (-0.025, 0.062) | 0.394 |
| Model 2 | 0.022 (-0.001, 0.044) | 0.058 |  | 0.032 (-0.008, 0.072) | 0.118 |
| Model 3 | 0.026 (0.002, 0.050) | 0.037 |  | 0.043 (0.000, 0.086) | 0.049 |
| Model 4 | 0.038 (-0.014, 0.090) | 0.144 |  | 0.080 (-0.021, 0.181) | 0.116 |
| Model 5 | 0.039 (-0.016, 0.094) | 0.157 |  | 0.079 (-0.028, 0.186) | 0.142 |
| TIR (%): 3.9–10.0 mmol/L | | | | | |
| Model 1 | -0.014 (-0.040, 0.011) | 0.267 |  | -0.018 (-0.062, 0.025) | 0.401 |
| Model 2 | -0.024 (-0.046, -0.001) | 0.038 |  | -0.031 (-0.071, 0.010) | 0.137 |
| Model 3 | -0.030 (-0.054, -0.005) | 0.019 |  | -0.043 (-0.087, 0.001) | 0.056 |
| Model 4 | -0.036 (-0.072, 0.001) | 0.057 |  | -0.063 (-0.136, 0.009) | 0.083 |
| Model 5 | -0.036 (-0.075, 0.002) | 0.064 |  | -0.070 (-0.145, 0.006) | 0.071 |
| TAR (%): >10.0 mmol/L | | | | | |
| Model 1 | 0.001 (-0.001, 0.004) | 0.368 |  | 0.001 (-0.003, 0.006) | 0.525 |
| Model 2 | 0.002 (0.000, 0.004) | 0.081 |  | 0.002 (-0.002, 0.006) | 0.224 |
| Model 3 | 0.003 (0.000, 0.005) | 0.042 |  | 0.004 (-0.001, 0.008) | 0.091 |
| Model 4 | 0.003 (-0.001, 0.008) | 0.120 |  | 0.006 (-0.003, 0.015) | 0.167 |
| Model 5 | 0.004 (-0.001, 0.008) | 0.127 |  | 0.006 (-0.003, 0.015) | 0.158 |
| TAR (%): >13.9 mmol/L | | | | | |
| Model 1 | 0.004 (-0.003, 0.011) | 0.252 |  | 0.003 (-0.009, 0.015) | 0.624 |
| Model 2 | 0.007 (0.000, 0.013) | 0.037 |  | 0.006 (-0.005, 0.018) | 0.291 |
| Model 3 | 0.009 (0.002, 0.015) | 0.018 |  | 0.009 (-0.004, 0.022) | 0.157 |
| Model 4 | 0.007 (-0.003, 0.017) | 0.167 |  | 0.006 (-0.015, 0.026) | 0.574 |
| Model 5 | 0.007 (-0.004, 0.017) | 0.189 |  | 0.006 (-0.015, 0.027) | 0.557 |
| TBR (%): <3.9 mmol/L | | | | | |
| Model 1 | 0.002 (-0.005, 0.009) | 0.614 |  | 0.003 (-0.009, 0.015) | 0.609 |
| Model 2 | 0.002 (-0.004, 0.009) | 0.493 |  | 0.004 (-0.008, 0.015) | 0.539 |
| Model 3 | 0.002 (-0.005, 0.009) | 0.521 |  | 0.003 (-0.009, 0.015) | 0.635 |
| Model 4 | 0.003 (-0.003, 0.010) | 0.315 |  | 0.007 (-0.007, 0.020) | 0.333 |
| Model 5 | 0.003 (-0.004, 0.011) | 0.364 |  | 0.007 (-0.007, 0.021) | 0.317 |
| TBR (%): <3.0 mmol/L | | | | | |
| Model 1 | 0.040 (-0.071, 0.152) | 0.468 |  | 0.057 (-0.130, 0.244) | 0.541 |
| Model 2 | 0.055 (-0.041, 0.152) | 0.256 |  | 0.077 (-0.096, 0.250) | 0.375 |
| Model 3 | 0.055 (-0.045, 0.154) | 0.271 |  | 0.078 (-0.097, 0.254) | 0.374 |
| Model 4 | 0.081 (-0.014, 0.176) | 0.093 |  | 0.134 (-0.054, 0.323) | 0.156 |
| Model 5 | 0.083 (-0.020, 0.185) | 0.109 |  | 0.152 (-0.049, 0.353) | 0.134 |
| HBGI | | | | | |
| Model 1 | 0.006 (-0.007, 0.019) | 0.338 |  | 0.005 (-0.016, 0.026) | 0.638 |
| Model 2 | 0.009 (-0.002, 0.019) | 0.118 |  | 0.008 (-0.011, 0.028) | 0.398 |
| Model 3 | 0.012 (-0.001, 0.024) | 0.061 |  | 0.014 (-0.008, 0.037) | 0.205 |
| Model 4 | 0.011 (-0.008, 0.029) | 0.256 |  | 0.012 (-0.025, 0.048) | 0.515 |
| Model 5 | 0.011 (-0.008, 0.031) | 0.254 |  | 0.015 (-0.024, 0.054) | 0.428 |
| LBGI | | | | | |
| Model 1 | 0.008 (-0.021, 0.037) | 0.562 |  | 0.007 (-0.042, 0.056) | 0.777 |
| Model 2 | 0.002 (-0.024, 0.028) | 0.889 |  | -0.003 (-0.049, 0.044) | 0.901 |
| Model 3 | 0.001 (-0.026, 0.029) | 0.926 |  | -0.008 (-0.057, 0.040) | 0.725 |
| Model 4 | 0.015 (-0.022, 0.052) | 0.425 |  | 0.020 (-0.053, 0.092) | 0.588 |
| Model 5 | 0.012 (-0.029, 0.053) | 0.559 |  | 0.020 (-0.060, 0.100) | 0.609 |
| MODD (mmol/L) | | | | | |
| Model 1 | -0.053 (-0.151, 0.045) | 0.281 |  | -0.087 (-0.252, 0.078) | 0.293 |
| Model 2 | -0.015 (-0.104, 0.074) | 0.731 |  | -0.036 (-0.194, 0.123) | 0.654 |
| Model 3 | -0.014 (-0.118, 0.090) | 0.790 |  | -0.026 (-0.209, 0.157) | 0.776 |
| Model 4 | -0.053 (-0.164, 0.058) | 0.337 |  | -0.073 (-0.291, 0.145) | 0.502 |
| Model 5 | -0.044 (-0.164, 0.076) | 0.457 |  | -0.042 (-0.279, 0.195) | 0.720 |
| IQR (mmol/L) | | | | | |
| Model 1 | -0.051 (-0.127, 0.024) | 0.178 |  | -0.069 (-0.197, 0.059) | 0.283 |
| Model 2 | -0.020 (-0.089, 0.049) | 0.566 |  | -0.026 (-0.149, 0.097) | 0.673 |
| Model 3 | -0.021 (-0.102, 0.060) | 0.599 |  | -0.018 (-0.161, 0.125) | 0.802 |
| Model 4 | -0.042 (-0.127, 0.043) | 0.320 |  | -0.036 (-0.205, 0.132) | 0.664 |
| Model 5 | -0.031 (-0.125, 0.063) | 0.506 |  | -0.002 (-0.187, 0.184) | 0.986 |

Univariable and multivariable linear regression analysis. Model 1 was not adjusted. Model 2 was adjusted for age and sex. Model 3 was adjusted for BMI, duration of diabetes, and the covariates in Model 2. Model 4 was adjusted for smoking status, HbA1c, systolic blood pressure, total cholesterol, HDL cholesterol, log-transformed triglycerides, uric acid, and the covariates in Model 3. Model 5 was adjusted for eGFR, log-transformed urine albumin-to-creatinine ratio, and the covariates in Model 4.

IMT, intima-media thickness; SD, standard deviation; CV, coefficient of variation; MAGE, mean amplitude of glycemic excursion; TIR, time in range; TAR, time above range; TBR, time below range; HBGI, high blood glucose index; LBGI, low blood glucose index; MODD, mean of daily differences; IQR, interquartile range

**Table S17 Associations of the metrics of continuous glucose monitoring with gray-scale median among participants not using anti-diabetic medications**

|  | mean-GSM  (n = 50) | |  | thickened lesion-GSM  (n = 42) | |  | plaque-GSM  (n = 35) | |
| --- | --- | --- | --- | --- | --- | --- | --- | --- |
|  | β (95 % CI) | *P* value |  | β (95 % CI) | *P* value |  | β (95 % CI) | *P* value |
| Mean glucose (mmol/L) | | | | | | | | |
| Model 1 | 1.707 (-1.380, 4.793) | 0.272 |  | 1.151 (-2.878, 5.181) | 0.567 |  | 1.138 (-5.588, 7.864) | 0.733 |
| Model 2 | 1.573 (-1.595, 4.741) | 0.323 |  | 0.815 (-3.396, 5.026) | 0.697 |  | 1.788 (-4.713, 8.290) | 0.579 |
| Model 3 | 0.324 (-3.075, 3.724) | 0.849 |  | 1.092 (-3.797, 5.980) | 0.653 |  | 1.434 (-5.960, 8.827) | 0.695 |
| Model 4 | -0.564 (-8.191, 7.063) | 0.882 |  | -8.905 (-20.599, 2.789) | 0.130 |  | -12.268 (-35.150, 10.615) | 0.277 |
| Model 5 | -0.036 (-7.426, 7.355) | 0.992 |  | -4.110 (-16.059, 7.838) | 0.485 |  | -7.926 (-33.336, 17.484) | 0.521 |
| Median glucose (mmol/L) | | | | | | | | |
| Model 1 | 9.205 (0.470, 17.940) | 0.039 |  | 3.242 (-8.669, 15.153) | 0.585 |  | 3.610 (-15.567, 22.787) | 0.704 |
| Model 2 | 9.438 (0.621, 18.254) | 0.036 |  | 3.082 (-9.157, 15.321) | 0.613 |  | 6.295 (-12.084, 24.675) | 0.490 |
| Model 3 | 6.203 (-4.026, 16.431) | 0.228 |  | 4.822 (-10.470, 20.113) | 0.527 |  | 5.746 (-16.179, 27.670) | 0.596 |
| Model 4 | 4.944 (-8.590, 18.479) | 0.463 |  | 6.579 (-13.659, 26.817) | 0.510 |  | 0.786 (-33.854, 35.425) | 0.963 |
| Model 5 | -0.104 (-14.893, 14.685) | 0.989 |  | 0.715 (-19.685, 21.116) | 0.943 |  | -9.202 (-48.096, 29.693) | 0.625 |
| SD (mmol/L) | | | | | | | | |
| Model 1 | 0.752 (-0.124, 1.628) | 0.091 |  | 0.089 (-1.184, 1.362) | 0.889 |  | 0.167 (-1.689, 2.022) | 0.856 |
| Model 2 | 0.852 (-0.041, 1.744) | 0.061 |  | 0.195 (-1.138, 1.528) | 0.768 |  | 0.309 (-1.465, 2.084) | 0.725 |
| Model 3 | 0.640 (-0.299, 1.579) | 0.177 |  | 0.238 (-1.225, 1.701) | 0.743 |  | 0.238 (-1.658, 2.134) | 0.799 |
| Model 4 | 0.570 (-0.544, 1.683) | 0.306 |  | 1.225 (-0.288, 2.738) | 0.108 |  | 0.463 (-2.055, 2.980) | 0.706 |
| Model 5 | -0.039 (-1.340, 1.262) | 0.952 |  | 0.532 (-1.163, 2.228) | 0.524 |  | -0.546 (-3.866, 2.774) | 0.734 |
| CV (%) | | | | | | | | |
| Model 1 | 3.202 (0.504, 5.900) | 0.021 |  | 1.362 (-2.671, 5.394) | 0.499 |  | 0.070 (-6.059, 6.200) | 0.981 |
| Model 2 | 3.476 (0.749, 6.204) | 0.014 |  | 1.462 (-2.770, 5.694) | 0.489 |  | 1.373 (-4.566, 7.311) | 0.641 |
| Model 3 | 2.751 (-0.174, 5.676) | 0.065 |  | 1.819 (-2.987, 6.626) | 0.448 |  | 1.022 (-5.557, 7.600) | 0.753 |
| Model 4 | 2.682 (-0.945, 6.309) | 0.142 |  | 3.800 (-1.857, 9.458) | 0.179 |  | 0.179 (-9.953, 10.312) | 0.971 |
| Model 5 | 2.133 (-1.695, 5.960) | 0.265 |  | 3.198 (-2.265, 8.660) | 0.239 |  | -1.672 (-12.648, 9.304) | 0.753 |
| MAGE (mmol/L) | | | | | | | | |
| Model 1 | 1.454 (-1.715, 4.622) | 0.361 |  | 1.118 (-3.025, 5.262) | 0.589 |  | 1.581 (-5.255, 8.417) | 0.641 |
| Model 2 | 1.283 (-1.986, 4.552) | 0.434 |  | 0.730 (-3.625, 5.084) | 0.736 |  | 2.256 (-4.358, 8.871) | 0.492 |
| Model 3 | 0.114 (-3.321, 3.549) | 0.947 |  | 0.923 (-3.993, 5.840) | 0.706 |  | 1.974 (-5.383, 9.332) | 0.587 |
| Model 4 | -0.701 (-8.746, 7.343) | 0.861 |  | -9.487 (-20.749, 1.775) | 0.095 |  | -7.236 (-29.895, 15.423) | 0.513 |
| Model 5 | 1.052 (-6.845, 8.949) | 0.788 |  | -3.789 (-16.118, 8.541) | 0.533 |  | 0.513 (-27.560, 28.587) | 0.970 |
| TIR (%): 3.9–10.0 mmol/L | | | | | | | | |
| Model 1 | -1.294 (-4.464, 1.876) | 0.416 |  | 0.044 (-3.979, 4.068) | 0.982 |  | -5.880 (-12.218, 0.458) | 0.068 |
| Model 2 | -1.003 (-4.289, 2.283) | 0.542 |  | 0.425 (-3.763, 4.614) | 0.838 |  | -5.942 (-12.128, 0.244) | 0.059 |
| Model 3 | 0.465 (-3.053, 3.983) | 0.791 |  | 0.483 (-4.309, 5.276) | 0.839 |  | -6.190 (-13.033, 0.653) | 0.075 |
| Model 4 | 2.977 (-2.718, 8.672) | 0.296 |  | 5.284 (-2.293, 12.860) | 0.164 |  | -7.306 (-20.400, 5.789) | 0.258 |
| Model 5 | 4.063 (-1.461, 9.587) | 0.144 |  | 5.257 (-1.660, 12.174) | 0.130 |  | -6.541 (-19.771, 6.690) | 0.313 |
| TAR (%): >10.0 mmol/L | | | | | | | | |
| Model 1 | 0.159 (-0.148, 0.466) | 0.303 |  | 0.034 (-0.360, 0.427) | 0.864 |  | 0.272 (-0.381, 0.925) | 0.403 |
| Model 2 | 0.140 (-0.176, 0.455) | 0.378 |  | -0.003 (-0.414, 0.407) | 0.987 |  | 0.309 (-0.324, 0.942) | 0.327 |
| Model 3 | -0.008 (-0.354, 0.339) | 0.964 |  | 0.001 (-0.485, 0.488) | 0.995 |  | 0.305 (-0.426, 1.037) | 0.400 |
| Model 4 | -0.281 (-0.953, 0.392) | 0.403 |  | -0.870 (-1.793, 0.053) | 0.064 |  | -0.312 (-2.073, 1.449) | 0.716 |
| Model 5 | -0.310 (-0.959, 0.338) | 0.337 |  | -0.657 (-1.543, 0.230) | 0.140 |  | -0.168 (-1.951, 1.616) | 0.846 |
| TAR (%): >13.9 mmol/L | | | | | | | | |
| Model 1 | 0.308 (-0.577, 1.193) | 0.488 |  | 0.075 (-1.034, 1.184) | 0.892 |  | 1.260 (-0.504, 3.023) | 0.156 |
| Model 2 | 0.207 (-0.723, 1.136) | 0.657 |  | -0.034 (-1.210, 1.141) | 0.953 |  | 1.037 (-0.702, 2.776) | 0.233 |
| Model 3 | -0.241 (-1.238, 0.755) | 0.628 |  | -0.025 (-1.387, 1.338) | 0.971 |  | 1.090 (-0.923, 3.103) | 0.277 |
| Model 4 | -0.889 (-2.411, 0.632) | 0.243 |  | -0.642 (-2.491, 1.208) | 0.483 |  | 0.274 (-3.012, 3.560) | 0.864 |
| Model 5 | -1.127 (-2.574, 0.319) | 0.122 |  | -0.759 (-2.450, 0.933) | 0.364 |  | 0.378 (-2.913, 3.669) | 0.812 |
| TBR (%): <3.9 mmol/L | | | | | | | | |
| Model 1 | -0.301 (-1.185, 0.584) | 0.498 |  | -0.407 (-1.692, 0.878) | 0.526 |  | 2.207 (0.608, 3.806) | 0.008 |
| Model 2 | -0.387 (-1.300, 0.525) | 0.398 |  | -0.392 (-1.713, 0.928) | 0.551 |  | 1.924 (0.332, 3.516) | 0.019 |
| Model 3 | -0.255 (-1.159, 0.649) | 0.572 |  | -0.426 (-1.824, 0.971) | 0.540 |  | 2.179 (0.507, 3.852) | 0.012 |
| Model 4 | -0.305 (-1.339, 0.729) | 0.554 |  | 0.081 (-1.309, 1.471) | 0.905 |  | 3.036 (1.017, 5.056) | 0.005 |
| Model 5 | -0.552 (-1.556, 0.451) | 0.271 |  | -0.421 (-1.744, 0.902) | 0.518 |  | 2.896 (0.640, 5.152) | 0.015 |
| TBR (%): <3.0 mmol/L | | | | | | | | |
| Model 1 | 1.414 (-12.212, 15.040) | 0.836 |  | 1.483 (-17.611, 20.578) | 0.876 |  | 36.421 (11.124, 61.718) | 0.006 |
| Model 2 | 0.808 (-13.031, 14.646) | 0.907 |  | 0.975 (-18.524, 20.475) | 0.920 |  | 37.384 (13.748, 61.020) | 0.003 |
| Model 3 | 2.362 (-11.197, 15.920) | 0.727 |  | 0.759 (-20.400, 21.917) | 0.942 |  | 43.314 (18.284, 68.345) | 0.001 |
| Model 4 | 2.001 (-12.865, 16.867) | 0.786 |  | 8.093 (-11.926, 28.112) | 0.414 |  | 52.505 (26.154, 78.857) | <0.001 |
| Model 5 | -1.482 (-16.402, 13.439) | 0.841 |  | -1.164 (-21.277, 18.948) | 0.906 |  | 53.704 (25.853, 81.555) | <0.001 |
| HBGI | | | | | | | | |
| Model 1 | 0.837 (-0.686, 2.360) | 0.275 |  | 0.174 (-1.783, 2.130) | 0.859 |  | 1.318 (-1.808, 4.445) | 0.397 |
| Model 2 | 0.762 (-0.792, 2.316) | 0.329 |  | 0.036 (-1.983, 2.056) | 0.971 |  | 1.406 (-1.610, 4.422) | 0.349 |
| Model 3 | -0.021 (-1.762, 1.720) | 0.981 |  | 0.086 (-2.364, 2.536) | 0.944 |  | 1.413 (-2.161, 4.987) | 0.425 |
| Model 4 | -0.765 (-3.574, 2.044) | 0.584 |  | -0.846 (-4.908, 3.215) | 0.672 |  | -0.674 (-7.661, 6.313) | 0.843 |
| Model 5 | -1.406 (-4.169, 1.357) | 0.308 |  | -0.909 (-4.655, 2.838) | 0.622 |  | -0.862 (-7.904, 6.180) | 0.800 |
| LBGI | | | | | | | | |
| Model 1 | -1.039 (-4.568, 2.491) | 0.557 |  | -2.880 (-7.937, 2.177) | 0.256 |  | 5.496 (-1.742, 12.734) | 0.132 |
| Model 2 | -1.168 (-4.843, 2.506) | 0.525 |  | -2.613 (-7.919, 2.694) | 0.325 |  | 3.985 (-3.182, 11.152) | 0.265 |
| Model 3 | -0.516 (-4.198, 3.167) | 0.779 |  | -2.826 (-8.468, 2.816) | 0.316 |  | 4.684 (-2.902, 12.270) | 0.217 |
| Model 4 | -0.528 (-6.116, 5.060) | 0.849 |  | 1.848 (-5.875, 9.572) | 0.627 |  | 13.233 (0.899, 25.568) | 0.037 |
| Model 5 | -3.257 (-8.883, 2.368) | 0.247 |  | -2.630 (-10.461, 5.201) | 0.496 |  | 15.554 (0.006, 31.102) | 0.050 |
| MODD (mmol/L) | | | | | | | | |
| Model 1 | 4.615 (-7.427, 16.658) | 0.445 |  | 1.399 (-14.092, 16.891) | 0.856 |  | 5.946 (-18.922, 30.815) | 0.630 |
| Model 2 | 4.174 (-8.370, 16.718) | 0.506 |  | 0.112 (-16.230, 16.454) | 0.989 |  | 11.502 (-13.046, 36.051) | 0.347 |
| Model 3 | -2.726 (-16.707, 11.254) | 0.696 |  | 0.569 (-19.851, 20.989) | 0.955 |  | 11.765 (-17.632, 41.161) | 0.420 |
| Model 4 | -2.852 (-19.669, 13.965) | 0.733 |  | 4.228 (-17.116, 25.571) | 0.688 |  | 15.183 (-28.322, 58.689) | 0.475 |
| Model 5 | -3.701 (-20.628, 13.226) | 0.659 |  | -0.333 (-21.637, 20.971) | 0.975 |  | 3.232 (-45.055, 51.520) | 0.890 |
| IQR (mmol/L) | | | | | | | | |
| Model 1 | 7.389 (-1.758, 16.536) | 0.111 |  | 1.661 (-10.314, 13.635) | 0.781 |  | 3.363 (-16.801, 23.527) | 0.736 |
| Model 2 | 6.912 (-2.646, 16.470) | 0.152 |  | 0.553 (-12.012, 13.117) | 0.930 |  | 6.664 (-13.277, 26.605) | 0.501 |
| Model 3 | 2.529 (-8.407, 13.464) | 0.643 |  | 1.054 (-14.360, 16.468) | 0.890 |  | 5.800 (-17.834, 29.434) | 0.619 |
| Model 4 | 1.937 (-11.035, 14.909) | 0.764 |  | 1.481 (-14.053, 17.014) | 0.846 |  | 6.351 (-27.786, 40.487) | 0.702 |
| Model 5 | 2.044 (-11.230, 15.319) | 0.756 |  | -2.474 (-18.220, 13.272) | 0.749 |  | -7.302 (-46.625, 32.022) | 0.701 |

Univariable and multivariable linear regression analysis. Model 1 was not adjusted. Model 2 was adjusted for age and sex. Model 3 was adjusted for BMI, duration of diabetes, and the covariates in Model 2. Model 4 was adjusted for smoking status, HbA1c, systolic blood pressure, total cholesterol, HDL cholesterol, log-transformed triglycerides, uric acid, and the covariates in Model 3. Model 5 was adjusted for eGFR, log-transformed urine albumin-to-creatinine ratio, and the covariates in Model 4.

GSM, gray-scale median; SD, standard deviation; CV, coefficient of variation; MAGE, mean amplitude of glycemic excursion; TIR, time in range; TAR, time above range; TBR, time below range; HBGI, high blood glucose index; LBGI, low blood glucose index; MODD, mean of daily differences; IQR, interquartile range

**Table S18 Associations of the metrics of continuous glucose monitoring with intima-media thickness among participants using insulin therapy**

|  | mean-IMT (n = 97) | |  | CCA-max-IMT (n = 97) | |
| --- | --- | --- | --- | --- | --- |
|  | β (95% CI) | *P* value |  | β (95% CI) | *P* value |
| Mean glucose (mmol/L) | | | | | |
| Model 1 | -0.013 (-0.028, 0.002) | 0.097 |  | -0.033 (-0.089, 0.023) | 0.244 |
| Model 2 | -0.010 (-0.024, 0.004) | 0.159 |  | -0.028 (-0.084, 0.028) | 0.327 |
| Model 3 | -0.010 (-0.024, 0.004) | 0.174 |  | -0.026 (-0.082, 0.031) | 0.365 |
| Model 4 | 0.002 (-0.022, 0.026) | 0.853 |  | -0.005 (-0.100, 0.090) | 0.917 |
| Model 5 | 0.003 (-0.022, 0.027) | 0.841 |  | -0.002 (-0.099, 0.094) | 0.962 |
| Median glucose (mmol/L) | | | | | |
| Model 1 | -0.036 (-0.084, 0.011) | 0.130 |  | -0.073 (-0.246, 0.100) | 0.405 |
| Model 2 | -0.031 (-0.074, 0.012) | 0.152 |  | -0.064 (-0.236, 0.107) | 0.458 |
| Model 3 | -0.030 (-0.074, 0.013) | 0.173 |  | -0.056 (-0.229, 0.118) | 0.525 |
| Model 4 | -0.013 (-0.067, 0.040) | 0.623 |  | -0.002 (-0.212, 0.209) | 0.989 |
| Model 5 | -0.015 (-0.070, 0.040) | 0.593 |  | 0.007 (-0.208, 0.223) | 0.947 |
| SD (mmol/L) | | | | | |
| Model 1 | 0.000 (-0.005, 0.005) | 0.994 |  | 0.004 (-0.015, 0.022) | 0.694 |
| Model 2 | 0.000 (-0.005, 0.005) | 0.906 |  | 0.003 (-0.016, 0.022) | 0.740 |
| Model 3 | 0.000 (-0.005, 0.005) | 0.986 |  | 0.004 (-0.015, 0.023) | 0.653 |
| Model 4 | -0.001 (-0.006, 0.005) | 0.778 |  | 0.005 (-0.016, 0.026) | 0.615 |
| Model 5 | -0.001 (-0.007, 0.005) | 0.721 |  | 0.006 (-0.016, 0.027) | 0.581 |
| CV (%) | | | | | |
| Model 1 | -0.005 (-0.018, 0.007) | 0.410 |  | -0.017 (-0.063, 0.029) | 0.464 |
| Model 2 | -0.005 (-0.017, 0.007) | 0.388 |  | -0.017 (-0.062, 0.029) | 0.468 |
| Model 3 | -0.004 (-0.016, 0.007) | 0.467 |  | -0.013 (-0.060, 0.033) | 0.570 |
| Model 4 | 0.001 (-0.013, 0.015) | 0.878 |  | -0.001 (-0.056, 0.053) | 0.965 |
| Model 5 | 0.001 (-0.014, 0.015) | 0.946 |  | 0.001 (-0.056, 0.057) | 0.980 |
| MAGE (mmol/L) | | | | | |
| Model 1 | -0.012 (-0.026, 0.003) | 0.117 |  | -0.029 (-0.082, 0.025) | 0.289 |
| Model 2 | -0.009 (-0.023, 0.004) | 0.173 |  | -0.024 (-0.078, 0.029) | 0.368 |
| Model 3 | -0.009 (-0.023, 0.004) | 0.185 |  | -0.023 (-0.077, 0.031) | 0.398 |
| Model 4 | 0.003 (-0.021, 0.026) | 0.823 |  | -0.002 (-0.094, 0.091) | 0.970 |
| Model 5 | 0.003 (-0.021, 0.027) | 0.824 |  | 0.000 (-0.094, 0.094) | 1.000 |
| TIR (%): 3.9–10.0 mmol/L | | | | | |
| Model 1 | 0.006 (-0.010, 0.022) | 0.449 |  | 0.005 (-0.052, 0.062) | 0.856 |
| Model 2 | 0.005 (-0.009, 0.019) | 0.470 |  | 0.004 (-0.052, 0.060) | 0.899 |
| Model 3 | 0.004 (-0.010, 0.019) | 0.559 |  | -0.001 (-0.058, 0.056) | 0.962 |
| Model 4 | -0.008 (-0.028, 0.011) | 0.405 |  | -0.044 (-0.121, 0.033) | 0.263 |
| Model 5 | -0.008 (-0.028, 0.013) | 0.446 |  | -0.047 (-0.126, 0.032) | 0.239 |
| TAR (%): >10.0 mmol/L | | | | | |
| Model 1 | -0.001 (-0.002, 0.001) | 0.187 |  | -0.003 (-0.008, 0.003) | 0.335 |
| Model 2 | -0.001 (-0.002, 0.001) | 0.246 |  | -0.002 (-0.008, 0.003) | 0.403 |
| Model 3 | -0.001 (-0.002, 0.001) | 0.287 |  | -0.002 (-0.007, 0.003) | 0.475 |
| Model 4 | 0.001 (-0.002, 0.003) | 0.630 |  | 0.001 (-0.007, 0.009) | 0.809 |
| Model 5 | 0.001 (-0.002, 0.003) | 0.634 |  | 0.001 (-0.007, 0.010) | 0.768 |
| TAR (%): >13.9 mmol/L | | | | | |
| Model 1 | -0.002 (-0.005, 0.000) | 0.097 |  | -0.002 (-0.011, 0.007) | 0.650 |
| Model 2 | -0.002 (-0.004, 0.001) | 0.141 |  | -0.001 (-0.011, 0.008) | 0.777 |
| Model 3 | -0.002 (-0.004, 0.001) | 0.156 |  | -0.001 (-0.010, 0.009) | 0.858 |
| Model 4 | 0.000 (-0.004, 0.004) | 0.939 |  | 0.007 (-0.008, 0.021) | 0.368 |
| Model 5 | 0.000 (-0.004, 0.004) | 0.909 |  | 0.007 (-0.008, 0.022) | 0.343 |
| TBR (%): <3.9 mmol/L | | | | | |
| Model 1 | 0.004 (0.000, 0.008) | 0.075 |  | 0.018 (0.003, 0.033) | 0.019 |
| Model 2 | 0.003 (-0.001, 0.007) | 0.154 |  | 0.017 (0.002, 0.033) | 0.031 |
| Model 3 | 0.003 (-0.001, 0.007) | 0.125 |  | 0.018 (0.003, 0.034) | 0.022 |
| Model 4 | 0.002 (-0.003, 0.007) | 0.382 |  | 0.019 (0.001, 0.036) | 0.037 |
| Model 5 | 0.002 (-0.003, 0.007) | 0.463 |  | 0.020 (0.002, 0.039) | 0.031 |
| TBR (%): <3.0 mmol/L | | | | | |
| Model 1 | 0.008 (-0.003, 0.019) | 0.170 |  | 0.039 (-0.001, 0.080) | 0.058 |
| Model 2 | 0.006 (-0.004, 0.017) | 0.250 |  | 0.037 (-0.004, 0.078) | 0.078 |
| Model 3 | 0.007 (-0.004, 0.017) | 0.217 |  | 0.039 (-0.002, 0.080) | 0.064 |
| Model 4 | 0.004 (-0.008, 0.016) | 0.480 |  | 0.037 (-0.009, 0.083) | 0.110 |
| Model 5 | 0.004 (-0.009, 0.016) | 0.573 |  | 0.040 (-0.008, 0.088) | 0.101 |
| HBGI | | | | | |
| Model 1 | -0.005 (-0.010, 0.001) | 0.094 |  | -0.008 (-0.028, 0.013) | 0.451 |
| Model 2 | -0.004 (-0.009, 0.001) | 0.146 |  | -0.006 (-0.026, 0.014) | 0.563 |
| Model 3 | -0.004 (-0.009, 0.002) | 0.171 |  | -0.005 (-0.025, 0.016) | 0.652 |
| Model 4 | 0.001 (-0.008, 0.009) | 0.905 |  | 0.010 (-0.023, 0.043) | 0.545 |
| Model 5 | 0.000 (-0.008, 0.009) | 0.939 |  | 0.011 (-0.022, 0.045) | 0.505 |
| LBGI | | | | | |
| Model 1 | 0.005 (-0.008, 0.017) | 0.461 |  | 0.022 (-0.022, 0.066) | 0.330 |
| Model 2 | 0.004 (-0.008, 0.015) | 0.544 |  | 0.020 (-0.025, 0.065) | 0.373 |
| Model 3 | 0.004 (-0.008, 0.015) | 0.517 |  | 0.021 (-0.024, 0.067) | 0.354 |
| Model 4 | 0.001 (-0.012, 0.015) | 0.858 |  | 0.024 (-0.029, 0.077) | 0.371 |
| Model 5 | 0.000 (-0.014, 0.014) | 0.998 |  | 0.025 (-0.031, 0.081) | 0.371 |
| MODD (mmol/L) | | | | | |
| Model 1 | -0.040 (-0.079, -0.001) | 0.046 |  | -0.117 (-0.260, 0.026) | 0.108 |
| Model 2 | -0.023 (-0.060, 0.014) | 0.218 |  | -0.088 (-0.234, 0.057) | 0.230 |
| Model 3 | -0.022 (-0.059, 0.015) | 0.242 |  | -0.083 (-0.230, 0.063) | 0.262 |
| Model 4 | -0.002 (-0.050, 0.047) | 0.950 |  | -0.049 (-0.238, 0.140) | 0.607 |
| Model 5 | -0.004 (-0.055, 0.046) | 0.862 |  | -0.043 (-0.241, 0.156) | 0.669 |
| IQR (mmol/L) | | | | | |
| Model 1 | -0.041 (-0.074, -0.008) | 0.016 |  | -0.106 (-0.228, 0.016) | 0.086 |
| Model 2 | -0.024 (-0.056, 0.007) | 0.128 |  | -0.079 (-0.204, 0.047) | 0.216 |
| Model 3 | -0.023 (-0.055, 0.009) | 0.152 |  | -0.072 (-0.199, 0.055) | 0.262 |
| Model 4 | -0.008 (-0.051, 0.035) | 0.714 |  | -0.041 (-0.211, 0.129) | 0.630 |
| Model 5 | -0.011 (-0.056, 0.035) | 0.639 |  | -0.034 (-0.212, 0.144) | 0.705 |

Univariable and multivariable linear regression analysis. Model 1 was not adjusted. Model 2 was adjusted for age and sex. Model 3 was adjusted for BMI, duration of diabetes, and the covariates in Model 2. Model 4 was adjusted for smoking status, HbA1c, systolic blood pressure, total cholesterol, HDL cholesterol, log-transformed triglycerides, uric acid, and the covariates in Model 3. Model 5 was adjusted for eGFR, log-transformed urine albumin-to-creatinine ratio, and the covariates in Model 4.

IMT, intima-media thickness; SD, standard deviation; CV, coefficient of variation; MAGE, mean amplitude of glycemic excursion; TIR, time in range; TAR, time above range; TBR, time below range; HBGI, high blood glucose index; LBGI, low blood glucose index; MODD, mean of daily differences; IQR, interquartile range

**Table S19 Associations of the metrics of continuous glucose monitoring with gray-scale median among participants using insulin therapy**

|  | mean-GSM  (n = 96) | |  | thickened lesion-GSM  (n = 92) | |  | plaque-GSM  (n = 85) | |
| --- | --- | --- | --- | --- | --- | --- | --- | --- |
|  | β (95 % CI) | *P* value |  | β (95 % CI) | *P* value |  | β (95 % CI) | *P* value |
| Mean glucose (mmol/L) | | | | | | | | |
| Model 1 | -0.492 (-1.958, 0.975) | 0.507 |  | -0.550 (-1.954, 0.854) | 0.439 |  | -2.068 (-4.446, 0.311) | 0.088 |
| Model 2 | -0.607 (-2.096, 0.883) | 0.421 |  | -0.659 (-2.073, 0.755) | 0.357 |  | -2.079 (-4.495, 0.336) | 0.091 |
| Model 3 | -0.728 (-2.176, 0.719) | 0.320 |  | -0.650 (-2.085, 0.785) | 0.370 |  | -2.072 (-4.528, 0.385) | 0.097 |
| Model 4 | -1.563 (-3.702, 0.576) | 0.150 |  | -1.283 (-3.608, 1.042) | 0.275 |  | -1.372 (-6.032, 3.288) | 0.559 |
| Model 5 | -1.590 (-3.764, 0.584) | 0.149 |  | -1.393 (-3.732, 0.947) | 0.239 |  | -1.127 (-5.751, 3.497) | 0.628 |
| Median glucose (mmol/L) | | | | | | | | |
| Model 1 | 0.522 (-4.040, 5.084) | 0.821 |  | 2.050 (-2.272, 6.371) | 0.349 |  | 0.977 (-6.813, 8.767) | 0.804 |
| Model 2 | 0.470 (-4.128, 5.069) | 0.839 |  | 1.929 (-2.387, 6.246) | 0.377 |  | 0.737 (-7.146, 8.620) | 0.853 |
| Model 3 | -0.011 (-4.513, 4.490) | 0.996 |  | 2.019 (-2.382, 6.421) | 0.364 |  | 0.831 (-7.194, 8.856) | 0.837 |
| Model 4 | -1.815 (-6.683, 3.052) | 0.460 |  | 2.995 (-2.152, 8.141) | 0.250 |  | 5.339 (-4.897, 15.574) | 0.302 |
| Model 5 | -1.811 (-6.786, 3.164) | 0.471 |  | 2.959 (-2.258, 8.176) | 0.262 |  | 4.414 (-5.824, 14.651) | 0.392 |
| SD (mmol/L) | | | | | | | | |
| Model 1 | 0.227 (-0.259, 0.714) | 0.356 |  | 0.522 (0.058, 0.987) | 0.028 |  | 0.913 (0.087, 1.739) | 0.031 |
| Model 2 | 0.266 (-0.235, 0.768) | 0.294 |  | 0.565 (0.092, 1.037) | 0.020 |  | 0.890 (0.048, 1.733) | 0.039 |
| Model 3 | 0.254 (-0.235, 0.743) | 0.306 |  | 0.578 (0.096, 1.060) | 0.019 |  | 0.898 (0.042, 1.753) | 0.040 |
| Model 4 | 0.048 (-0.441, 0.538) | 0.845 |  | 0.606 (0.095, 1.118) | 0.021 |  | 0.764 (-0.234, 1.762) | 0.131 |
| Model 5 | 0.058 (-0.442, 0.559) | 0.817 |  | 0.631 (0.115, 1.147) | 0.017 |  | 0.608 (-0.406, 1.623) | 0.235 |
| CV (%) | | | | | | | | |
| Model 1 | -0.613 (-1.823, 0.597) | 0.317 |  | 0.632 (-0.516, 1.781) | 0.277 |  | 0.632 (-1.512, 2.776) | 0.559 |
| Model 2 | -0.606 (-1.823, 0.611) | 0.325 |  | 0.622 (-0.522, 1.767) | 0.283 |  | 0.609 (-1.555, 2.773) | 0.577 |
| Model 3 | -0.652 (-1.844, 0.539) | 0.280 |  | 0.646 (-0.524, 1.815) | 0.276 |  | 0.642 (-1.568, 2.853) | 0.565 |
| Model 4 | -1.043 (-2.298, 0.213) | 0.102 |  | 0.921 (-0.412, 2.255) | 0.173 |  | 1.419 (-1.304, 4.141) | 0.302 |
| Model 5 | -1.051 (-2.344, 0.242) | 0.110 |  | 0.985 (-0.372, 2.342) | 0.152 |  | 1.088 (-1.645, 3.822) | 0.429 |
| MAGE (mmol/L) | | | | | | | | |
| Model 1 | -0.475 (-1.879, 0.930) | 0.504 |  | -0.633 (-1.980, 0.713) | 0.353 |  | -2.144 (-4.416, 0.127) | 0.064 |
| Model 2 | -0.573 (-1.998, 0.852) | 0.426 |  | -0.723 (-2.077, 0.631) | 0.291 |  | -2.153 (-4.456, 0.150) | 0.067 |
| Model 3 | -0.661 (-2.043, 0.721) | 0.345 |  | -0.716 (-2.088, 0.656) | 0.303 |  | -2.145 (-4.484, 0.194) | 0.072 |
| Model 4 | -1.387 (-3.474, 0.700) | 0.190 |  | -1.430 (-3.719, 0.859) | 0.217 |  | -1.528 (-6.096, 3.040) | 0.507 |
| Model 5 | -1.397 (-3.514, 0.720) | 0.193 |  | -1.484 (-3.786, 0.817) | 0.203 |  | -1.424 (-5.948, 3.100) | 0.532 |
| TIR (%): 3.9–10.0 mmol/L | | | | | | | | |
| Model 1 | 0.498 (-0.988, 1.984) | 0.507 |  | 0.303 (-1.127, 1.732) | 0.675 |  | 1.087 (-1.358, 3.531) | 0.379 |
| Model 2 | 0.519 (-0.973, 2.012) | 0.491 |  | 0.301 (-1.123, 1.726) | 0.675 |  | 1.105 (-1.361, 3.571) | 0.375 |
| Model 3 | 0.656 (-0.811, 2.123) | 0.377 |  | 0.288 (-1.172, 1.749) | 0.696 |  | 1.100 (-1.433, 3.633) | 0.390 |
| Model 4 | 1.004 (-0.780, 2.787) | 0.266 |  | 0.314 (-1.635, 2.262) | 0.749 |  | -0.065 (-3.781, 3.651) | 0.972 |
| Model 5 | 0.995 (-0.832, 2.822) | 0.281 |  | 0.237 (-1.750, 2.224) | 0.813 |  | 0.377 (-3.336, 4.090) | 0.840 |
| TAR (%): >10.0 mmol/L | | | | | | | | |
| Model 1 | -0.047 (-0.187, 0.092) | 0.502 |  | -0.035 (-0.170, 0.100) | 0.605 |  | -0.154 (-0.383, 0.075) | 0.184 |
| Model 2 | -0.055 (-0.196, 0.086) | 0.443 |  | -0.041 (-0.175, 0.094) | 0.552 |  | -0.154 (-0.386, 0.078) | 0.190 |
| Model 3 | -0.066 (-0.203, 0.072) | 0.346 |  | -0.040 (-0.177, 0.098) | 0.569 |  | -0.154 (-0.390, 0.083) | 0.200 |
| Model 4 | -0.113 (-0.301, 0.074) | 0.233 |  | -0.058 (-0.264, 0.148) | 0.576 |  | -0.034 (-0.438, 0.371) | 0.868 |
| Model 5 | -0.114 (-0.305, 0.076) | 0.236 |  | -0.062 (-0.269, 0.145) | 0.555 |  | -0.031 (-0.432, 0.369) | 0.877 |
| TAR (%): >13.9 mmol/L | | | | | | | | |
| Model 1 | -0.065 (-0.307, 0.176) | 0.592 |  | -0.129 (-0.357, 0.099) | 0.264 |  | -0.308 (-0.692, 0.077) | 0.115 |
| Model 2 | -0.080 (-0.325, 0.164) | 0.515 |  | -0.145 (-0.373, 0.083) | 0.211 |  | -0.309 (-0.700, 0.083) | 0.121 |
| Model 3 | -0.122 (-0.361, 0.117) | 0.311 |  | -0.144 (-0.378, 0.090) | 0.224 |  | -0.310 (-0.711, 0.092) | 0.129 |
| Model 4 | -0.219 (-0.544, 0.105) | 0.182 |  | -0.271 (-0.618, 0.077) | 0.125 |  | -0.207 (-0.873, 0.459) | 0.538 |
| Model 5 | -0.220 (-0.551, 0.111) | 0.189 |  | -0.279 (-0.630, 0.072) | 0.118 |  | -0.216 (-0.877, 0.444) | 0.516 |
| TBR (%): <3.9 mmol/L | | | | | | | | |
| Model 1 | 0.029 (-0.376, 0.434) | 0.886 |  | 0.067 (-0.317, 0.451) | 0.729 |  | 0.471 (-0.183, 1.125) | 0.156 |
| Model 2 | 0.074 (-0.346, 0.493) | 0.728 |  | 0.115 (-0.280, 0.510) | 0.564 |  | 0.465 (-0.207, 1.138) | 0.173 |
| Model 3 | 0.069 (-0.340, 0.477) | 0.739 |  | 0.121 (-0.282, 0.523) | 0.553 |  | 0.476 (-0.210, 1.162) | 0.171 |
| Model 4 | 0.009 (-0.405, 0.423) | 0.966 |  | 0.106 (-0.336, 0.548) | 0.635 |  | 0.173 (-0.653, 1.000) | 0.677 |
| Model 5 | 0.029 (-0.408, 0.466) | 0.895 |  | 0.179 (-0.283, 0.641) | 0.443 |  | -0.059 (-0.918, 0.801) | 0.892 |
| TBR (%): <3.0 mmol/L | | | | | | | | |
| Model 1 | 0.092 (-0.991, 1.175) | 0.867 |  | 0.414 (-0.608, 1.435) | 0.423 |  | 1.288 (-0.427, 3.003) | 0.139 |
| Model 2 | 0.170 (-0.934, 1.274) | 0.760 |  | 0.503 (-0.529, 1.535) | 0.336 |  | 1.265 (-0.490, 3.020) | 0.155 |
| Model 3 | 0.171 (-0.902, 1.244) | 0.752 |  | 0.515 (-0.535, 1.564) | 0.332 |  | 1.279 (-0.503, 3.061) | 0.157 |
| Model 4 | -0.129 (-1.204, 0.945) | 0.811 |  | 0.455 (-0.678, 1.587) | 0.426 |  | 0.589 (-1.522, 2.699) | 0.580 |
| Model 5 | -0.090 (-1.223, 1.042) | 0.874 |  | 0.670 (-0.511, 1.851) | 0.262 |  | 0.005 (-2.189, 2.198) | 0.997 |
| HBGI | | | | | | | | |
| Model 1 | -0.150 (-0.686, 0.386) | 0.579 |  | -0.141 (-0.650, 0.368) | 0.584 |  | -0.577 (-1.445, 0.292) | 0.190 |
| Model 2 | -0.180 (-0.720, 0.361) | 0.511 |  | -0.173 (-0.681, 0.336) | 0.502 |  | -0.589 (-1.469, 0.291) | 0.187 |
| Model 3 | -0.249 (-0.778, 0.280) | 0.352 |  | -0.168 (-0.688, 0.352) | 0.522 |  | -0.588 (-1.489, 0.313) | 0.198 |
| Model 4 | -0.518 (-1.268, 0.231) | 0.173 |  | -0.254 (-1.071, 0.564) | 0.538 |  | -0.140 (-1.733, 1.454) | 0.862 |
| Model 5 | -0.520 (-1.284, 0.245) | 0.180 |  | -0.264 (-1.092, 0.564) | 0.528 |  | -0.238 (-1.822, 1.345) | 0.765 |
| LBGI | | | | | | | | |
| Model 1 | 0.605 (-0.549, 1.759) | 0.300 |  | 0.952 (-0.148, 2.053) | 0.089 |  | 1.765 (-0.109, 3.638) | 0.064 |
| Model 2 | 0.717 (-0.474, 1.909) | 0.235 |  | 1.069 (-0.057, 2.196) | 0.063 |  | 1.709 (-0.211, 3.630) | 0.080 |
| Model 3 | 0.760 (-0.394, 1.913) | 0.194 |  | 1.074 (-0.068, 2.215) | 0.065 |  | 1.706 (-0.241, 3.652) | 0.085 |
| Model 4 | 0.199 (-1.018, 1.417) | 0.745 |  | 1.053 (-0.248, 2.354) | 0.111 |  | 1.234 (-1.197, 3.665) | 0.315 |
| Model 5 | 0.285 (-1.011, 1.581) | 0.662 |  | 1.394 (0.037, 2.751) | 0.044 |  | 0.549 (-2.015, 3.113) | 0.670 |
| MODD (mmol/L) | | | | | | | | |
| Model 1 | 0.524 (-3.308, 4.354) | 0.787 |  | 0.584 (-3.050, 4.218) | 0.750 |  | -1.057 (-7.394, 5.281) | 0.741 |
| Model 2 | 0.101 (-3.826, 4.028) | 0.959 |  | 0.010 (-3.679, 3.699) | 0.996 |  | -1.402 (-7.972, 5.170) | 0.672 |
| Model 3 | -0.114 (-3.937, 3.708) | 0.953 |  | 0.039 (-3.707, 3.785) | 0.984 |  | -1.363 (-8.037, 5.311) | 0.685 |
| Model 4 | -2.176 (-6.564, 2.212) | 0.327 |  | 0.782 (-3.964, 5.528) | 0.744 |  | 2.984 (-6.055, 12.023) | 0.512 |
| Model 5 | -2.205 (-6.791, 2.381) | 0.341 |  | 0.936 (-3.993, 5.865) | 0.706 |  | 1.903 (-7.260, 11.065) | 0.680 |
| IQR (mmol/L) | | | | | | | | |
| Model 1 | 0.496 (-2.769, 3.761) | 0.764 |  | 0.295 (-2.808, 3.399) | 0.850 |  | -1.821 (-7.243, 3.601) | 0.506 |
| Model 2 | 0.047 (-3.335, 3.429) | 0.978 |  | -0.308 (-3.486, 2.870) | 0.848 |  | -2.171 (-7.845, 3.503) | 0.449 |
| Model 3 | -0.138 (-3.440, 3.165) | 0.934 |  | -0.282 (-3.519, 2.954) | 0.863 |  | -2.145 (-7.920, 3.630) | 0.462 |
| Model 4 | -1.830 (-5.795, 2.134) | 0.361 |  | 0.128 (-4.190, 4.445) | 0.953 |  | 0.779 (-7.447, 9.005) | 0.851 |
| Model 5 | -1.860 (-5.992, 2.272) | 0.373 |  | 0.129 (-4.351, 4.608) | 0.955 |  | -0.063 (-8.374, 8.248) | 0.988 |

Univariable and multivariable linear regression analysis. Model 1 was not adjusted. Model 2 was adjusted for age and sex. Model 3 was adjusted for BMI, duration of diabetes, and the covariates in Model 2. Model 4 was adjusted for smoking status, HbA1c, systolic blood pressure, total cholesterol, HDL cholesterol, log-transformed triglycerides, uric acid, and the covariates in Model 3. Model 5 was adjusted for eGFR, log-transformed urine albumin-to-creatinine ratio, and the covariates in Model 4.

GSM, gray-scale median; SD, standard deviation; CV, coefficient of variation; MAGE, mean amplitude of glycemic excursion; TIR, time in range; TAR, time above range; TBR, time below range; HBGI, high blood glucose index; LBGI, low blood glucose index; MODD, mean of daily differences; IQR, interquartile range

**Table S20 Associations of the metrics of continuous glucose monitoring with intima-media thickness among participants not using insulin therapy**

|  | mean-IMT (n = 503) | |  | CCA-max-IMT (n = 503) | |
| --- | --- | --- | --- | --- | --- |
|  | β (95% CI) | *P* value |  | β (95% CI) | *P* value |
| Mean glucose (mmol/L) | | | | | |
| Model 1 | 0.003 (-0.006, 0.011) | 0.539 |  | 0.015 (-0.008, 0.039) | 0.193 |
| Model 2 | 0.002 (-0.006, 0.010) | 0.669 |  | 0.014 (-0.009, 0.037) | 0.231 |
| Model 3 | 0.002 (-0.006, 0.010) | 0.646 |  | 0.011 (-0.012, 0.033) | 0.351 |
| Model 4 | 0.004 (-0.010, 0.017) | 0.606 |  | 0.015 (-0.021, 0.052) | 0.415 |
| Model 5 | 0.006 (-0.008, 0.020) | 0.381 |  | 0.020 (-0.018, 0.057) | 0.297 |
| Median glucose (mmol/L) | | | | | |
| Model 1 | 0.013 (-0.011, 0.038) | 0.282 |  | 0.062 (-0.005, 0.129) | 0.068 |
| Model 2 | 0.000 (-0.023, 0.023) | 0.997 |  | 0.036 (-0.030, 0.101) | 0.282 |
| Model 3 | 0.001 (-0.023, 0.025) | 0.917 |  | 0.046 (-0.020, 0.112) | 0.168 |
| Model 4 | 0.000 (-0.027, 0.028) | 0.991 |  | 0.049 (-0.027, 0.125) | 0.204 |
| Model 5 | 0.004 (-0.024, 0.031) | 0.800 |  | 0.056 (-0.021, 0.133) | 0.153 |
| SD (mmol/L) | | | | | |
| Model 1 | 0.001 (-0.001, 0.004) | 0.312 |  | 0.004 (-0.002, 0.011) | 0.190 |
| Model 2 | 0.000 (-0.003, 0.002) | 0.777 |  | 0.001 (-0.005, 0.008) | 0.705 |
| Model 3 | 0.000 (-0.003, 0.002) | 0.863 |  | 0.004 (-0.003, 0.010) | 0.281 |
| Model 4 | 0.000 (-0.003, 0.002) | 0.828 |  | 0.003 (-0.003, 0.010) | 0.320 |
| Model 5 | 0.000 (-0.003, 0.002) | 0.901 |  | 0.004 (-0.003, 0.010) | 0.287 |
| CV (%) | | | | | |
| Model 1 | 0.005 (-0.003, 0.012) | 0.219 |  | 0.022 (0.001, 0.043) | 0.037 |
| Model 2 | 0.001 (-0.007, 0.008) | 0.885 |  | 0.014 (-0.006, 0.034) | 0.180 |
| Model 3 | 0.001 (-0.007, 0.008) | 0.819 |  | 0.018 (-0.002, 0.039) | 0.073 |
| Model 4 | 0.001 (-0.007, 0.009) | 0.837 |  | 0.022 (0.000, 0.044) | 0.046 |
| Model 5 | 0.002 (-0.006, 0.010) | 0.618 |  | 0.025 (0.003, 0.048) | 0.027 |
| MAGE (mmol/L) | | | | | |
| Model 1 | 0.002 (-0.006, 0.011) | 0.629 |  | 0.013 (-0.010, 0.036) | 0.279 |
| Model 2 | 0.002 (-0.007, 0.010) | 0.706 |  | 0.012 (-0.011, 0.034) | 0.300 |
| Model 3 | 0.002 (-0.006, 0.010) | 0.686 |  | 0.008 (-0.015, 0.030) | 0.501 |
| Model 4 | 0.002 (-0.011, 0.015) | 0.748 |  | 0.005 (-0.031, 0.041) | 0.779 |
| Model 5 | 0.004 (-0.009, 0.018) | 0.513 |  | 0.009 (-0.028, 0.046) | 0.631 |
| TIR (%): 3.9–10.0 mmol/L | | | | | |
| Model 1 | -0.003 (-0.011, 0.005) | 0.491 |  | -0.012 (-0.035, 0.010) | 0.279 |
| Model 2 | -0.001 (-0.009, 0.007) | 0.790 |  | -0.009 (-0.031, 0.013) | 0.424 |
| Model 3 | -0.001 (-0.009, 0.007) | 0.750 |  | -0.006 (-0.028, 0.016) | 0.572 |
| Model 4 | -0.001 (-0.013, 0.010) | 0.807 |  | -0.002 (-0.034, 0.030) | 0.884 |
| Model 5 | -0.003 (-0.015, 0.009) | 0.611 |  | -0.005 (-0.038, 0.027) | 0.746 |
| TAR (%): >10.0 mmol/L | | | | | |
| Model 1 | 0.000 (-0.001, 0.001) | 0.667 |  | 0.001 (-0.001, 0.003) | 0.278 |
| Model 2 | 0.000 (-0.001, 0.001) | 0.883 |  | 0.001 (-0.001, 0.003) | 0.363 |
| Model 3 | 0.000 (-0.001, 0.001) | 0.855 |  | 0.001 (-0.001, 0.003) | 0.528 |
| Model 4 | 0.000 (-0.001, 0.001) | 0.913 |  | 0.001 (-0.003, 0.004) | 0.709 |
| Model 5 | 0.000 (-0.001, 0.002) | 0.668 |  | 0.001 (-0.002, 0.004) | 0.565 |
| TAR (%): >13.9 mmol/L | | | | | |
| Model 1 | 0.000 (-0.002, 0.002) | 0.946 |  | 0.001 (-0.005, 0.006) | 0.770 |
| Model 2 | 0.000 (-0.002, 0.002) | 0.771 |  | 0.000 (-0.005, 0.006) | 0.876 |
| Model 3 | 0.000 (-0.002, 0.002) | 0.777 |  | 0.000 (-0.005, 0.005) | 0.963 |
| Model 4 | 0.000 (-0.003, 0.002) | 0.843 |  | -0.002 (-0.009, 0.005) | 0.549 |
| Model 5 | 0.000 (-0.003, 0.002) | 0.918 |  | -0.002 (-0.009, 0.005) | 0.603 |
| TBR (%): <3.9 mmol/L | | | | | |
| Model 1 | 0.002 (-0.002, 0.005) | 0.313 |  | -0.001 (-0.010, 0.009) | 0.887 |
| Model 2 | 0.001 (-0.002, 0.004) | 0.636 |  | -0.003 (-0.012, 0.006) | 0.565 |
| Model 3 | 0.001 (-0.002, 0.004) | 0.596 |  | -0.002 (-0.011, 0.007) | 0.719 |
| Model 4 | 0.001 (-0.003, 0.004) | 0.698 |  | -0.003 (-0.012, 0.006) | 0.536 |
| Model 5 | 0.000 (-0.003, 0.004) | 0.796 |  | -0.003 (-0.012, 0.006) | 0.501 |
| TBR (%): <3.0 mmol/L | | | | | |
| Model 1 | 0.007 (-0.008, 0.022) | 0.381 |  | -0.002 (-0.043, 0.040) | 0.936 |
| Model 2 | 0.004 (-0.010, 0.019) | 0.543 |  | -0.006 (-0.047, 0.034) | 0.753 |
| Model 3 | 0.005 (-0.010, 0.019) | 0.505 |  | -0.002 (-0.042, 0.038) | 0.924 |
| Model 4 | 0.007 (-0.007, 0.021) | 0.319 |  | 0.003 (-0.036, 0.042) | 0.888 |
| Model 5 | 0.007 (-0.008, 0.021) | 0.357 |  | 0.001 (-0.039, 0.040) | 0.968 |
| HBGI | | | | | |
| Model 1 | 0.001 (-0.003, 0.004) | 0.694 |  | 0.005 (-0.005, 0.015) | 0.307 |
| Model 2 | 0.000 (-0.004, 0.003) | 0.915 |  | 0.003 (-0.006, 0.013) | 0.492 |
| Model 3 | 0.000 (-0.004, 0.003) | 0.952 |  | 0.003 (-0.007, 0.012) | 0.545 |
| Model 4 | 0.000 (-0.005, 0.005) | 0.998 |  | 0.003 (-0.011, 0.017) | 0.671 |
| Model 5 | 0.001 (-0.004, 0.006) | 0.814 |  | 0.004 (-0.010, 0.018) | 0.574 |
| LBGI | | | | | |
| Model 1 | 0.002 (-0.008, 0.012) | 0.709 |  | -0.005 (-0.032, 0.021) | 0.701 |
| Model 2 | -0.002 (-0.011, 0.008) | 0.746 |  | -0.012 (-0.038, 0.014) | 0.356 |
| Model 3 | -0.001 (-0.011, 0.008) | 0.785 |  | -0.007 (-0.033, 0.018) | 0.570 |
| Model 4 | -0.002 (-0.012, 0.008) | 0.683 |  | -0.011 (-0.038, 0.017) | 0.449 |
| Model 5 | -0.003 (-0.013, 0.007) | 0.553 |  | -0.012 (-0.040, 0.016) | 0.384 |
| MODD (mmol/L) | | | | | |
| Model 1 | 0.005 (-0.022, 0.031) | 0.733 |  | 0.037 (-0.035, 0.110) | 0.312 |
| Model 2 | 0.004 (-0.021, 0.029) | 0.753 |  | 0.037 (-0.034, 0.108) | 0.304 |
| Model 3 | 0.005 (-0.020, 0.031) | 0.681 |  | 0.038 (-0.033, 0.109) | 0.288 |
| Model 4 | 0.006 (-0.026, 0.039) | 0.704 |  | 0.024 (-0.065, 0.113) | 0.595 |
| Model 5 | 0.010 (-0.023, 0.042) | 0.559 |  | 0.028 (-0.062, 0.118) | 0.542 |
| IQR (mmol/L) | | | | | |
| Model 1 | -0.004 (-0.025, 0.017) | 0.719 |  | 0.009 (-0.048, 0.066) | 0.755 |
| Model 2 | -0.003 (-0.023, 0.017) | 0.763 |  | 0.011 (-0.044, 0.067) | 0.688 |
| Model 3 | -0.002 (-0.022, 0.018) | 0.832 |  | 0.014 (-0.041, 0.070) | 0.609 |
| Model 4 | -0.004 (-0.030, 0.021) | 0.744 |  | -0.008 (-0.078, 0.062) | 0.822 |
| Model 5 | -0.001 (-0.026, 0.025) | 0.956 |  | -0.004 (-0.075, 0.067) | 0.918 |

Univariable and multivariable linear regression analysis. Model 1 was not adjusted. Model 2 was adjusted for age and sex. Model 3 was adjusted for BMI, duration of diabetes, and the covariates in Model 2. Model 4 was adjusted for smoking status, HbA1c, systolic blood pressure, total cholesterol, HDL cholesterol, log-transformed triglycerides, uric acid, and the covariates in Model 3. Model 5 was adjusted for eGFR, log-transformed urine albumin-to-creatinine ratio, and the covariates in Model 4.

IMT, intima-media thickness; SD, standard deviation; CV, coefficient of variation; MAGE, mean amplitude of glycemic excursion; TIR, time in range; TAR, time above range; TBR, time below range; HBGI, high blood glucose index; LBGI, low blood glucose index; MODD, mean of daily differences; IQR, interquartile range

**Table S21 Associations of the metrics of continuous glucose monitoring with gray-scale median among participants not using insulin therapy**

|  | mean-GSM  (n = 503) | |  | thickened lesion-GSM  (n = 474) | |  | plaque-GSM  (n = 405) | |
| --- | --- | --- | --- | --- | --- | --- | --- | --- |
|  | β (95 % CI) | *P* value |  | β (95 % CI) | *P* value |  | β (95 % CI) | *P* value |
| Mean glucose (mmol/L) | | | | | | | | |
| Model 1 | -1.913 (-3.010, -0.815) | <0.001 |  | -1.672 (-2.811, -0.533) | 0.004 |  | -2.299 (-4.156, -0.443) | 0.015 |
| Model 2 | -2.040 (-3.129, -0.950) | <0.001 |  | -1.568 (-2.694, -0.442) | 0.006 |  | -2.167 (-4.026, -0.309) | 0.022 |
| Model 3 | -1.820 (-2.903, -0.736) | 0.001 |  | -1.532 (-2.668, -0.395) | 0.008 |  | -2.030 (-3.898, -0.162) | 0.033 |
| Model 4 | -2.559 (-4.362, -0.756) | 0.005 |  | -1.479 (-3.403, 0.444) | 0.131 |  | -2.985 (-6.256, 0.286) | 0.074 |
| Model 5 | -2.292 (-4.101, -0.483) | 0.013 |  | -1.326 (-3.257, 0.605) | 0.178 |  | -2.790 (-6.091, 0.511) | 0.097 |
| Median glucose (mmol/L) | | | | | | | | |
| Model 1 | -6.256 (-9.387, -3.126) | <0.001 |  | -5.420 (-8.652, -2.188) | 0.001 |  | -6.239 (-11.625, -0.853) | 0.023 |
| Model 2 | -6.020 (-9.157, -2.884) | <0.001 |  | -4.575 (-7.785, -1.366) | 0.005 |  | -5.897 (-11.304, -0.491) | 0.033 |
| Model 3 | -6.287 (-9.421, -3.152) | <0.001 |  | -4.722 (-7.991, -1.453) | 0.005 |  | -6.059 (-11.536, -0.583) | 0.030 |
| Model 4 | -6.818 (-10.541, -3.095) | <0.001 |  | -4.187 (-8.158, -0.216) | 0.039 |  | -5.689 (-12.401, 1.022) | 0.096 |
| Model 5 | -6.562 (-10.267, -2.856) | <0.001 |  | -4.049 (-8.003, -0.095) | 0.045 |  | -5.532 (-12.246, 1.182) | 0.106 |
| SD (mmol/L) | | | | | | | | |
| Model 1 | -0.332 (-0.641, -0.023) | 0.035 |  | -0.260 (-0.582, 0.061) | 0.112 |  | -0.176 (-0.707, 0.356) | 0.517 |
| Model 2 | -0.279 (-0.590, 0.033) | 0.079 |  | -0.165 (-0.486, 0.155) | 0.311 |  | -0.148 (-0.684, 0.389) | 0.589 |
| Model 3 | -0.376 (-0.689, -0.062) | 0.019 |  | -0.191 (-0.521, 0.139) | 0.257 |  | -0.204 (-0.755, 0.348) | 0.468 |
| Model 4 | -0.434 (-0.751, -0.117) | 0.007 |  | -0.212 (-0.549, 0.125) | 0.217 |  | -0.195 (-0.763, 0.374) | 0.502 |
| Model 5 | -0.445 (-0.760, -0.129) | 0.006 |  | -0.215 (-0.550, 0.121) | 0.209 |  | -0.198 (-0.766, 0.371) | 0.495 |
| CV (%) | | | | | | | | |
| Model 1 | -1.673 (-2.645, -0.702) | <0.001 |  | -1.722 (-2.726, -0.719) | <0.001 |  | -1.629 (-3.308, 0.050) | 0.057 |
| Model 2 | -1.668 (-2.642, -0.694) | <0.001 |  | -1.439 (-2.437, -0.440) | 0.005 |  | -1.445 (-3.132, 0.242) | 0.093 |
| Model 3 | -1.848 (-2.814, -0.881) | <0.001 |  | -1.494 (-2.505, -0.484) | 0.004 |  | -1.548 (-3.250, 0.154) | 0.074 |
| Model 4 | -1.827 (-2.912, -0.742) | 0.001 |  | -1.352 (-2.513, -0.192) | 0.022 |  | -1.231 (-3.185, 0.724) | 0.216 |
| Model 5 | -1.703 (-2.784, -0.622) | 0.002 |  | -1.237 (-2.394, -0.080) | 0.036 |  | -1.189 (-3.149, 0.771) | 0.234 |
| MAGE (mmol/L) | | | | | | | | |
| Model 1 | -1.764 (-2.852, -0.676) | 0.002 |  | -1.492 (-2.625, -0.359) | 0.010 |  | -1.962 (-3.799, -0.124) | 0.036 |
| Model 2 | -1.904 (-2.984, -0.823) | <0.001 |  | -1.407 (-2.527, -0.288) | 0.014 |  | -1.831 (-3.670, 0.009) | 0.051 |
| Model 3 | -1.633 (-2.711, -0.556) | 0.003 |  | -1.363 (-2.495, -0.230) | 0.018 |  | -1.664 (-3.518, 0.190) | 0.078 |
| Model 4 | -1.971 (-3.755, -0.186) | 0.030 |  | -0.933 (-2.830, 0.964) | 0.334 |  | -1.861 (-5.067, 1.346) | 0.255 |
| Model 5 | -1.714 (-3.507, 0.078) | 0.061 |  | -0.775 (-2.681, 1.132) | 0.425 |  | -1.684 (-4.921, 1.552) | 0.307 |
| TIR (%): 3.9–10.0 mmol/L | | | | | | | | |
| Model 1 | 1.978 (0.927, 3.028) | <0.001 |  | 1.749 (0.657, 2.841) | 0.002 |  | 1.265 (-0.549, 3.078) | 0.171 |
| Model 2 | 2.068 (1.024, 3.112) | <0.001 |  | 1.578 (0.498, 2.658) | 0.004 |  | 1.109 (-0.706, 2.924) | 0.230 |
| Model 3 | 1.866 (0.824, 2.909) | <0.001 |  | 1.553 (0.459, 2.647) | 0.006 |  | 0.953 (-0.877, 2.782) | 0.307 |
| Model 4 | 2.714 (1.143, 4.284) | <0.001 |  | 1.708 (0.020, 3.397) | 0.047 |  | 0.112 (-2.742, 2.966) | 0.938 |
| Model 5 | 2.590 (1.033, 4.148) | 0.001 |  | 1.552 (-0.124, 3.228) | 0.069 |  | -0.115 (-2.960, 2.731) | 0.937 |
| TAR (%): >10.0 mmol/L | | | | | | | | |
| Model 1 | -0.201 (-0.302, -0.099) | <0.001 |  | -0.180 (-0.286, -0.074) | <0.001 |  | -0.192 (-0.367, -0.018) | 0.031 |
| Model 2 | -0.209 (-0.310, -0.108) | <0.001 |  | -0.168 (-0.273, -0.064) | 0.002 |  | -0.181 (-0.356, -0.006) | 0.043 |
| Model 3 | -0.188 (-0.289, -0.088) | <0.001 |  | -0.166 (-0.271, -0.060) | 0.002 |  | -0.167 (-0.343, 0.009) | 0.063 |
| Model 4 | -0.291 (-0.451, -0.130) | <0.001 |  | -0.207 (-0.379, -0.035) | 0.018 |  | -0.203 (-0.496, 0.090) | 0.174 |
| Model 5 | -0.266 (-0.426, -0.106) | 0.001 |  | -0.189 (-0.360, -0.018) | 0.031 |  | -0.181 (-0.474, 0.112) | 0.225 |
| TAR (%): >13.9 mmol/L | | | | | | | | |
| Model 1 | -0.307 (-0.566, -0.047) | 0.021 |  | -0.225 (-0.491, 0.042) | 0.098 |  | -0.226 (-0.653, 0.201) | 0.299 |
| Model 2 | -0.329 (-0.587, -0.071) | 0.013 |  | -0.201 (-0.464, 0.062) | 0.134 |  | -0.200 (-0.626, 0.227) | 0.357 |
| Model 3 | -0.293 (-0.548, -0.039) | 0.024 |  | -0.194 (-0.458, 0.070) | 0.149 |  | -0.182 (-0.609, 0.244) | 0.401 |
| Model 4 | -0.292 (-0.641, 0.056) | 0.100 |  | -0.081 (-0.448, 0.285) | 0.663 |  | 0.073 (-0.524, 0.671) | 0.809 |
| Model 5 | -0.290 (-0.635, 0.054) | 0.098 |  | -0.067 (-0.430, 0.295) | 0.715 |  | 0.098 (-0.496, 0.692) | 0.746 |
| TBR (%): <3.9 mmol/L | | | | | | | | |
| Model 1 | 0.264 (-0.174, 0.701) | 0.237 |  | 0.284 (-0.177, 0.745) | 0.226 |  | 1.303 (0.575, 2.030) | <0.001 |
| Model 2 | 0.272 (-0.163, 0.707) | 0.219 |  | 0.370 (-0.084, 0.824) | 0.110 |  | 1.353 (0.627, 2.078) | <0.001 |
| Model 3 | 0.249 (-0.182, 0.678) | 0.257 |  | 0.370 (-0.088, 0.827) | 0.113 |  | 1.380 (0.649, 2.110) | <0.001 |
| Model 4 | 0.043 (-0.406, 0.492) | 0.850 |  | 0.225 (-0.256, 0.705) | 0.359 |  | 1.369 (0.594, 2.143) | <0.001 |
| Model 5 | -0.057 (-0.504, 0.390) | 0.803 |  | 0.206 (-0.273, 0.686) | 0.398 |  | 1.407 (0.628, 2.186) | <0.001 |
| TBR (%): <3.0 mmol/L | | | | | | | | |
| Model 1 | -0.842 (-2.812, 1.128) | 0.402 |  | 1.019 (-0.991, 3.030) | 0.320 |  | 2.955 (-0.632, 6.542) | 0.106 |
| Model 2 | -0.796 (-2.751, 1.158) | 0.424 |  | 1.163 (-0.811, 3.137) | 0.248 |  | 2.992 (-0.588, 6.572) | 0.101 |
| Model 3 | -0.917 (-2.849, 1.016) | 0.352 |  | 1.150 (-0.837, 3.138) | 0.256 |  | 3.103 (-0.500, 6.705) | 0.091 |
| Model 4 | -1.107 (-3.051, 0.837) | 0.264 |  | 0.931 (-1.093, 2.955) | 0.367 |  | 3.043 (-0.632, 6.717) | 0.104 |
| Model 5 | -1.183 (-3.105, 0.740) | 0.227 |  | 0.970 (-1.032, 2.972) | 0.342 |  | 3.037 (-0.619, 6.692) | 0.103 |
| HBGI | | | | | | | | |
| Model 1 | -0.850 (-1.303, -0.397) | <0.001 |  | -0.678 (-1.146, -0.210) | 0.005 |  | -0.759 (-1.515, -0.003) | 0.049 |
| Model 2 | -0.867 (-1.318, -0.417) | <0.001 |  | -0.605 (-1.067, -0.143) | 0.010 |  | -0.704 (-1.460, 0.052) | 0.068 |
| Model 3 | -0.818 (-1.265, -0.371) | <0.001 |  | -0.597 (-1.063, -0.132) | 0.012 |  | -0.673 (-1.432, 0.085) | 0.082 |
| Model 4 | -1.190 (-1.859, -0.522) | <0.001 |  | -0.619 (-1.334, 0.096) | 0.090 |  | -0.627 (-1.811, 0.558) | 0.299 |
| Model 5 | -1.149 (-1.812, -0.485) | <0.001 |  | -0.571 (-1.281, 0.140) | 0.115 |  | -0.551 (-1.734, 0.632) | 0.360 |
| LBGI | | | | | | | | |
| Model 1 | 0.598 (-0.665, 1.861) | 0.353 |  | 0.984 (-0.333, 2.300) | 0.143 |  | 3.246 (1.114, 5.378) | 0.003 |
| Model 2 | 0.746 (-0.511, 2.003) | 0.244 |  | 1.211 (-0.087, 2.508) | 0.067 |  | 3.304 (1.170, 5.438) | 0.002 |
| Model 3 | 0.556 (-0.688, 1.801) | 0.380 |  | 1.183 (-0.127, 2.493) | 0.077 |  | 3.270 (1.118, 5.421) | 0.003 |
| Model 4 | -0.151 (-1.514, 1.212) | 0.828 |  | 0.659 (-0.786, 2.104) | 0.371 |  | 3.330 (0.929, 5.732) | 0.007 |
| Model 5 | -0.440 (-1.800, 0.920) | 0.525 |  | 0.649 (-0.792, 2.089) | 0.377 |  | 3.378 (0.965, 5.790) | 0.006 |
| MODD (mmol/L) | | | | | | | | |
| Model 1 | -6.422 (-9.832, -3.012) | <0.001 |  | -4.888 (-8.410, -1.366) | 0.007 |  | -6.017 (-11.986, -0.048) | 0.048 |
| Model 2 | -6.933 (-10.319, -3.548) | <0.001 |  | -4.729 (-8.204, -1.254) | 0.008 |  | -5.879 (-11.838, 0.080) | 0.053 |
| Model 3 | -6.713 (-10.108, -3.318) | <0.001 |  | -4.773 (-8.312, -1.234) | 0.008 |  | -5.662 (-11.703, 0.379) | 0.066 |
| Model 4 | -8.098 (-12.468, -3.727) | <0.001 |  | -3.583 (-8.252, 1.085) | 0.132 |  | -5.362 (-13.627, 2.903) | 0.203 |
| Model 5 | -7.712 (-12.057, -3.368) | <0.001 |  | -3.172 (-7.812, 1.468) | 0.180 |  | -5.144 (-13.407, 3.119) | 0.222 |
| IQR (mmol/L) | | | | | | | | |
| Model 1 | -3.770 (-6.446, -1.095) | 0.006 |  | -2.548 (-5.306, 0.209) | 0.070 |  | -3.622 (-8.236, 0.992) | 0.124 |
| Model 2 | -4.290 (-6.952, -1.628) | 0.002 |  | -2.500 (-5.225, 0.225) | 0.072 |  | -3.536 (-8.152, 1.079) | 0.133 |
| Model 3 | -4.219 (-6.883, -1.555) | 0.002 |  | -2.522 (-5.295, 0.250) | 0.074 |  | -3.389 (-8.065, 1.287) | 0.155 |
| Model 4 | -4.705 (-8.160, -1.251) | 0.008 |  | -0.908 (-4.565, 2.749) | 0.626 |  | -2.228 (-8.687, 4.231) | 0.498 |
| Model 5 | -4.370 (-7.799, -0.940) | 0.013 |  | -0.757 (-4.386, 2.872) | 0.682 |  | -2.172 (-8.623, 4.279) | 0.508 |

Univariable and multivariable linear regression analysis. Model 1 was not adjusted. Model 2 was adjusted for age and sex. Model 3 was adjusted for BMI, duration of diabetes, and the covariates in Model 2. Model 4 was adjusted for smoking status, HbA1c, systolic blood pressure, total cholesterol, HDL cholesterol, log-transformed triglycerides, uric acid, and the covariates in Model 3. Model 5 was adjusted for eGFR, log-transformed urine albumin-to-creatinine ratio, and the covariates in Model 4.

GSM, gray-scale median; SD, standard deviation; CV, coefficient of variation; MAGE, mean amplitude of glycemic excursion; TIR, time in range; TAR, time above range; TBR, time below range; HBGI, high blood glucose index; LBGI, low blood glucose index; MODD, mean of daily differences; IQR, interquartile range

**Table S22 Associations of the metrics of continuous glucose monitoring with intima-media thickness among participants using angiotensin-converting enzyme inhibitors or angiotensin II receptor blockers**

|  | mean-IMT (n = 266) | |  | CCA-max-IMT (n = 266) | |
| --- | --- | --- | --- | --- | --- |
|  | β (95% CI) | *P* value |  | β (95% CI) | *P* value |
| Mean glucose (mmol/L) | | | | | |
| Model 1 | 0.004 (-0.008, 0.016) | 0.512 |  | 0.023 (-0.018, 0.065) | 0.274 |
| Model 2 | 0.005 (-0.007, 0.016) | 0.400 |  | 0.026 (-0.014, 0.067) | 0.199 |
| Model 3 | 0.006 (-0.005, 0.018) | 0.293 |  | 0.022 (-0.019, 0.064) | 0.285 |
| Model 4 | 0.009 (-0.009, 0.027) | 0.318 |  | 0.042 (-0.021, 0.106) | 0.192 |
| Model 5 | 0.013 (-0.005, 0.031) | 0.163 |  | 0.048 (-0.019, 0.115) | 0.157 |
| Median glucose (mmol/L) | | | | | |
| Model 1 | 0.014 (-0.019, 0.046) | 0.402 |  | 0.093 (-0.019, 0.205) | 0.103 |
| Model 2 | 0.007 (-0.024, 0.038) | 0.656 |  | 0.073 (-0.036, 0.182) | 0.189 |
| Model 3 | 0.004 (-0.028, 0.036) | 0.813 |  | 0.064 (-0.048, 0.177) | 0.262 |
| Model 4 | 0.000 (-0.035, 0.036) | 0.989 |  | 0.074 (-0.052, 0.201) | 0.248 |
| Model 5 | 0.005 (-0.031, 0.041) | 0.780 |  | 0.082 (-0.050, 0.213) | 0.225 |
| SD (mmol/L) | | | | | |
| Model 1 | 0.001 (-0.002, 0.004) | 0.415 |  | 0.008 (-0.003, 0.018) | 0.165 |
| Model 2 | 0.000 (-0.003, 0.003) | 0.866 |  | 0.005 (-0.006, 0.015) | 0.398 |
| Model 3 | -0.001 (-0.004, 0.003) | 0.745 |  | 0.004 (-0.006, 0.015) | 0.423 |
| Model 4 | -0.001 (-0.004, 0.003) | 0.741 |  | 0.004 (-0.006, 0.015) | 0.423 |
| Model 5 | 0.000 (-0.003, 0.003) | 0.836 |  | 0.005 (-0.007, 0.016) | 0.405 |
| CV (%) | | | | | |
| Model 1 | 0.003 (-0.006, 0.013) | 0.498 |  | 0.024 (-0.010, 0.057) | 0.162 |
| Model 2 | 0.002 (-0.008, 0.011) | 0.710 |  | 0.020 (-0.013, 0.052) | 0.234 |
| Model 3 | 0.001 (-0.009, 0.010) | 0.897 |  | 0.018 (-0.015, 0.052) | 0.279 |
| Model 4 | -0.001 (-0.011, 0.009) | 0.866 |  | 0.025 (-0.012, 0.062) | 0.183 |
| Model 5 | 0.001 (-0.010, 0.011) | 0.901 |  | 0.029 (-0.010, 0.068) | 0.139 |
| MAGE (mmol/L) | | | | | |
| Model 1 | 0.004 (-0.008, 0.016) | 0.512 |  | 0.021 (-0.020, 0.062) | 0.309 |
| Model 2 | 0.005 (-0.007, 0.016) | 0.406 |  | 0.024 (-0.016, 0.064) | 0.233 |
| Model 3 | 0.006 (-0.005, 0.018) | 0.281 |  | 0.020 (-0.021, 0.061) | 0.342 |
| Model 4 | 0.009 (-0.008, 0.027) | 0.305 |  | 0.037 (-0.027, 0.100) | 0.253 |
| Model 5 | 0.013 (-0.005, 0.031) | 0.156 |  | 0.042 (-0.024, 0.109) | 0.212 |
| TIR (%): 3.9–10.0 mmol/L | | | | | |
| Model 1 | -0.007 (-0.017, 0.004) | 0.225 |  | -0.033 (-0.070, 0.004) | 0.079 |
| Model 2 | -0.005 (-0.016, 0.005) | 0.307 |  | -0.030 (-0.066, 0.007) | 0.108 |
| Model 3 | -0.006 (-0.017, 0.004) | 0.251 |  | -0.026 (-0.063, 0.011) | 0.162 |
| Model 4 | -0.006 (-0.020, 0.008) | 0.400 |  | -0.038 (-0.088, 0.012) | 0.133 |
| Model 5 | -0.008 (-0.022, 0.006) | 0.258 |  | -0.041 (-0.092, 0.011) | 0.123 |
| TAR (%): >10.0 mmol/L | | | | | |
| Model 1 | 0.000 (-0.001, 0.001) | 0.513 |  | 0.002 (-0.001, 0.006) | 0.224 |
| Model 2 | 0.000 (-0.001, 0.001) | 0.518 |  | 0.002 (-0.001, 0.006) | 0.216 |
| Model 3 | 0.000 (-0.001, 0.002) | 0.398 |  | 0.002 (-0.002, 0.006) | 0.307 |
| Model 4 | 0.001 (-0.001, 0.002) | 0.526 |  | 0.003 (-0.002, 0.009) | 0.250 |
| Model 5 | 0.001 (-0.001, 0.002) | 0.338 |  | 0.004 (-0.002, 0.009) | 0.229 |
| TAR (%): >13.9 mmol/L | | | | | |
| Model 1 | 0.001 (-0.002, 0.004) | 0.657 |  | 0.005 (-0.006, 0.015) | 0.392 |
| Model 2 | 0.001 (-0.002, 0.004) | 0.524 |  | 0.005 (-0.005, 0.016) | 0.297 |
| Model 3 | 0.001 (-0.002, 0.004) | 0.352 |  | 0.005 (-0.006, 0.015) | 0.402 |
| Model 4 | 0.001 (-0.003, 0.005) | 0.480 |  | 0.004 (-0.010, 0.018) | 0.538 |
| Model 5 | 0.002 (-0.002, 0.006) | 0.399 |  | 0.004 (-0.010, 0.019) | 0.560 |
| TBR (%): <3.9 mmol/L | | | | | |
| Model 1 | 0.003 (0.000, 0.006) | 0.089 |  | 0.009 (-0.002, 0.021) | 0.106 |
| Model 2 | 0.002 (-0.001, 0.005) | 0.254 |  | 0.006 (-0.005, 0.018) | 0.270 |
| Model 3 | 0.002 (-0.002, 0.005) | 0.371 |  | 0.006 (-0.005, 0.018) | 0.268 |
| Model 4 | 0.001 (-0.002, 0.004) | 0.524 |  | 0.007 (-0.005, 0.018) | 0.272 |
| Model 5 | 0.001 (-0.002, 0.004) | 0.535 |  | 0.007 (-0.006, 0.019) | 0.286 |
| TBR (%): <3.0 mmol/L | | | | | |
| Model 1 | 0.008 (-0.003, 0.019) | 0.149 |  | 0.035 (-0.002, 0.072) | 0.066 |
| Model 2 | 0.006 (-0.005, 0.017) | 0.260 |  | 0.029 (-0.008, 0.065) | 0.128 |
| Model 3 | 0.005 (-0.005, 0.016) | 0.331 |  | 0.028 (-0.009, 0.065) | 0.133 |
| Model 4 | 0.004 (-0.006, 0.015) | 0.423 |  | 0.033 (-0.004, 0.071) | 0.080 |
| Model 5 | 0.004 (-0.006, 0.015) | 0.426 |  | 0.033 (-0.006, 0.072) | 0.094 |
| HBGI | | | | | |
| Model 1 | 0.002 (-0.004, 0.007) | 0.540 |  | 0.011 (-0.007, 0.029) | 0.225 |
| Model 2 | 0.002 (-0.003, 0.007) | 0.526 |  | 0.011 (-0.006, 0.029) | 0.212 |
| Model 3 | 0.002 (-0.003, 0.007) | 0.446 |  | 0.009 (-0.009, 0.027) | 0.305 |
| Model 4 | 0.002 (-0.005, 0.009) | 0.619 |  | 0.014 (-0.011, 0.039) | 0.272 |
| Model 5 | 0.003 (-0.004, 0.010) | 0.411 |  | 0.015 (-0.011, 0.041) | 0.253 |
| LBGI | | | | | |
| Model 1 | 0.003 (-0.006, 0.013) | 0.509 |  | 0.009 (-0.024, 0.043) | 0.580 |
| Model 2 | 0.000 (-0.009, 0.010) | 0.956 |  | 0.000 (-0.033, 0.033) | 0.993 |
| Model 3 | -0.001 (-0.010, 0.008) | 0.827 |  | 0.000 (-0.034, 0.033) | 0.982 |
| Model 4 | -0.002 (-0.012, 0.008) | 0.717 |  | 0.001 (-0.035, 0.036) | 0.969 |
| Model 5 | -0.002 (-0.012, 0.008) | 0.659 |  | 0.000 (-0.037, 0.037) | 0.989 |
| MODD (mmol/L) | | | | | |
| Model 1 | 0.019 (-0.015, 0.054) | 0.264 |  | 0.046 (-0.073, 0.164) | 0.447 |
| Model 2 | 0.022 (-0.011, 0.054) | 0.192 |  | 0.053 (-0.062, 0.168) | 0.367 |
| Model 3 | 0.021 (-0.013, 0.055) | 0.226 |  | 0.038 (-0.083, 0.159) | 0.538 |
| Model 4 | 0.019 (-0.022, 0.059) | 0.362 |  | 0.024 (-0.121, 0.169) | 0.745 |
| Model 5 | 0.026 (-0.016, 0.068) | 0.225 |  | 0.023 (-0.133, 0.178) | 0.772 |
| IQR (mmol/L) | | | | | |
| Model 1 | 0.007 (-0.022, 0.035) | 0.652 |  | 0.023 (-0.076, 0.122) | 0.646 |
| Model 2 | 0.012 (-0.016, 0.039) | 0.411 |  | 0.038 (-0.058, 0.135) | 0.436 |
| Model 3 | 0.010 (-0.018, 0.039) | 0.483 |  | 0.026 (-0.074, 0.127) | 0.608 |
| Model 4 | 0.005 (-0.030, 0.039) | 0.783 |  | 0.002 (-0.123, 0.126) | 0.977 |
| Model 5 | 0.012 (-0.025, 0.048) | 0.523 |  | 0.001 (-0.132, 0.134) | 0.988 |

Univariable and multivariable linear regression analysis. Model 1 was not adjusted. Model 2 was adjusted for age and sex. Model 3 was adjusted for BMI, duration of diabetes, and the covariates in Model 2. Model 4 was adjusted for smoking status, HbA1c, systolic blood pressure, total cholesterol, HDL cholesterol, log-transformed triglycerides, uric acid, and the covariates in Model 3. Model 5 was adjusted for eGFR, log-transformed urine albumin-to-creatinine ratio, and the covariates in Model 4.

IMT, intima-media thickness; SD, standard deviation; CV, coefficient of variation; MAGE, mean amplitude of glycemic excursion; TIR, time in range; TAR, time above range; TBR, time below range; HBGI, high blood glucose index; LBGI, low blood glucose index; MODD, mean of daily differences; IQR, interquartile range

**Table S23 Associations of the metrics of continuous glucose monitoring with gray-scale median among participants using angiotensin-converting enzyme inhibitors or angiotensin II receptor blockers**

|  | mean-GSM  (n = 265) | |  | thickened lesion-GSM  (n = 250) | |  | plaque-GSM  (n = 215) | |
| --- | --- | --- | --- | --- | --- | --- | --- | --- |
|  | β (95 % CI) | *P* value |  | β (95 % CI) | *P* value |  | β (95 % CI) | *P* value |
| Mean glucose (mmol/L) | | | | | | | | |
| Model 1 | -2.043 (-3.585, -0.501) | 0.010 |  | -2.017 (-3.614, -0.420) | 0.014 |  | -3.673 (-6.293, -1.053) | 0.006 |
| Model 2 | -2.199 (-3.714, -0.683) | 0.005 |  | -2.140 (-3.728, -0.552) | 0.008 |  | -3.699 (-6.330, -1.067) | 0.006 |
| Model 3 | -1.676 (-3.195, -0.156) | 0.031 |  | -1.840 (-3.461, -0.219) | 0.026 |  | -3.149 (-5.816, -0.482) | 0.021 |
| Model 4 | -1.890 (-4.312, 0.532) | 0.126 |  | -0.850 (-3.420, 1.720) | 0.515 |  | -4.032 (-8.518, 0.454) | 0.078 |
| Model 5 | -1.371 (-3.843, 1.100) | 0.275 |  | -0.302 (-2.915, 2.310) | 0.820 |  | -3.293 (-7.899, 1.313) | 0.160 |
| Median glucose (mmol/L) | | | | | | | | |
| Model 1 | -8.075 (-12.178, -3.971) | <0.001 |  | -6.573 (-10.851, -2.296) | 0.003 |  | -7.330 (-14.524, -0.136) | 0.046 |
| Model 2 | -7.540 (-11.607, -3.474) | <0.001 |  | -6.347 (-10.608, -2.086) | 0.004 |  | -7.262 (-14.513, -0.010) | 0.050 |
| Model 3 | -7.082 (-11.179, -2.986) | <0.001 |  | -5.484 (-9.861, -1.107) | 0.014 |  | -5.741 (-13.135, 1.654) | 0.127 |
| Model 4 | -7.240 (-11.976, -2.504) | 0.003 |  | -3.554 (-8.652, 1.544) | 0.171 |  | -3.883 (-12.611, 4.845) | 0.381 |
| Model 5 | -6.838 (-11.652, -2.023) | 0.006 |  | -2.296 (-7.451, 2.860) | 0.381 |  | -2.781 (-11.699, 6.137) | 0.539 |
| SD (mmol/L) | | | | | | | | |
| Model 1 | -0.534 (-0.930, -0.137) | 0.008 |  | -0.373 (-0.790, 0.045) | 0.080 |  | -0.071 (-0.758, 0.617) | 0.840 |
| Model 2 | -0.450 (-0.845, -0.054) | 0.026 |  | -0.324 (-0.744, 0.096) | 0.130 |  | -0.054 (-0.750, 0.642) | 0.879 |
| Model 3 | -0.522 (-0.917, -0.127) | 0.010 |  | -0.284 (-0.713, 0.145) | 0.194 |  | 0.001 (-0.707, 0.708) | 0.998 |
| Model 4 | -0.560 (-0.962, -0.158) | 0.007 |  | -0.271 (-0.706, 0.163) | 0.219 |  | -0.051 (-0.785, 0.684) | 0.892 |
| Model 5 | -0.570 (-0.978, -0.162) | 0.006 |  | -0.188 (-0.626, 0.250) | 0.398 |  | 0.008 (-0.737, 0.753) | 0.983 |
| CV (%) | | | | | | | | |
| Model 1 | -1.914 (-3.157, -0.672) | 0.003 |  | -1.668 (-2.954, -0.381) | 0.011 |  | -1.387 (-3.630, 0.856) | 0.224 |
| Model 2 | -1.903 (-3.129, -0.677) | 0.002 |  | -1.622 (-2.901, -0.343) | 0.013 |  | -1.386 (-3.639, 0.868) | 0.227 |
| Model 3 | -1.875 (-3.088, -0.662) | 0.003 |  | -1.432 (-2.724, -0.139) | 0.030 |  | -1.077 (-3.340, 1.187) | 0.350 |
| Model 4 | -1.809 (-3.196, -0.423) | 0.011 |  | -0.983 (-2.466, 0.499) | 0.193 |  | -0.360 (-2.987, 2.266) | 0.787 |
| Model 5 | -1.775 (-3.193, -0.358) | 0.014 |  | -0.663 (-2.172, 0.847) | 0.388 |  | -0.063 (-2.749, 2.624) | 0.963 |
| MAGE (mmol/L) | | | | | | | | |
| Model 1 | -1.909 (-3.424, -0.393) | 0.014 |  | -2.010 (-3.582, -0.437) | 0.012 |  | -3.479 (-6.055, -0.903) | 0.008 |
| Model 2 | -2.050 (-3.540, -0.560) | 0.007 |  | -2.119 (-3.683, -0.556) | 0.008 |  | -3.497 (-6.084, -0.909) | 0.008 |
| Model 3 | -1.469 (-2.973, 0.035) | 0.055 |  | -1.808 (-3.412, -0.203) | 0.027 |  | -2.898 (-5.534, -0.262) | 0.031 |
| Model 4 | -1.358 (-3.770, 1.055) | 0.269 |  | -0.712 (-3.268, 1.843) | 0.583 |  | -3.299 (-7.735, 1.137) | 0.144 |
| Model 5 | -0.818 (-3.281, 1.646) | 0.514 |  | -0.254 (-2.850, 2.342) | 0.847 |  | -2.620 (-7.155, 1.916) | 0.256 |
| TIR (%): 3.9–10.0 mmol/L | | | | | | | | |
| Model 1 | 2.387 (1.015, 3.758) | <0.001 |  | 2.307 (0.890, 3.723) | 0.002 |  | 2.560 (0.167, 4.953) | 0.036 |
| Model 2 | 2.308 (0.956, 3.659) | <0.001 |  | 2.257 (0.850, 3.664) | 0.002 |  | 2.527 (0.121, 4.933) | 0.040 |
| Model 3 | 1.900 (0.545, 3.255) | 0.006 |  | 1.989 (0.553, 3.424) | 0.007 |  | 2.033 (-0.392, 4.457) | 0.100 |
| Model 4 | 2.525 (0.653, 4.398) | 0.008 |  | 1.678 (-0.328, 3.683) | 0.101 |  | 1.661 (-1.736, 5.058) | 0.336 |
| Model 5 | 2.181 (0.279, 4.083) | 0.025 |  | 1.124 (-0.903, 3.151) | 0.276 |  | 1.013 (-2.454, 4.480) | 0.565 |
| TAR (%): >10.0 mmol/L | | | | | | | | |
| Model 1 | -0.224 (-0.360, -0.088) | 0.001 |  | -0.216 (-0.356, -0.075) | 0.003 |  | -0.316 (-0.550, -0.082) | 0.008 |
| Model 2 | -0.227 (-0.361, -0.094) | <0.001 |  | -0.219 (-0.359, -0.080) | 0.002 |  | -0.315 (-0.551, -0.080) | 0.009 |
| Model 3 | -0.183 (-0.317, -0.049) | 0.008 |  | -0.194 (-0.336, -0.051) | 0.008 |  | -0.268 (-0.506, -0.030) | 0.027 |
| Model 4 | -0.263 (-0.471, -0.055) | 0.013 |  | -0.159 (-0.381, 0.063) | 0.160 |  | -0.322 (-0.707, 0.063) | 0.100 |
| Model 5 | -0.218 (-0.428, -0.007) | 0.043 |  | -0.105 (-0.329, 0.119) | 0.357 |  | -0.258 (-0.649, 0.133) | 0.195 |
| TAR (%): >13.9 mmol/L | | | | | | | | |
| Model 1 | -0.509 (-0.901, -0.118) | 0.011 |  | -0.455 (-0.855, -0.055) | 0.026 |  | -0.516 (-1.186, 0.154) | 0.131 |
| Model 2 | -0.545 (-0.930, -0.160) | 0.006 |  | -0.487 (-0.884, -0.090) | 0.016 |  | -0.523 (-1.196, 0.151) | 0.127 |
| Model 3 | -0.405 (-0.790, -0.019) | 0.040 |  | -0.420 (-0.825, -0.015) | 0.042 |  | -0.387 (-1.068, 0.294) | 0.263 |
| Model 4 | -0.441 (-0.965, 0.084) | 0.099 |  | -0.217 (-0.767, 0.334) | 0.438 |  | -0.139 (-1.089, 0.811) | 0.773 |
| Model 5 | -0.328 (-0.855, 0.200) | 0.222 |  | -0.100 (-0.651, 0.451) | 0.721 |  | 0.011 (-0.938, 0.961) | 0.982 |
| TBR (%): <3.9 mmol/L | | | | | | | | |
| Model 1 | -0.090 (-0.520, 0.341) | 0.683 |  | -0.105 (-0.554, 0.344) | 0.645 |  | 0.588 (-0.110, 1.286) | 0.098 |
| Model 2 | 0.017 (-0.412, 0.446) | 0.937 |  | -0.027 (-0.480, 0.427) | 0.908 |  | 0.625 (-0.081, 1.331) | 0.082 |
| Model 3 | -0.028 (-0.449, 0.392) | 0.895 |  | -0.019 (-0.473, 0.434) | 0.933 |  | 0.606 (-0.096, 1.308) | 0.090 |
| Model 4 | -0.214 (-0.661, 0.234) | 0.348 |  | -0.209 (-0.683, 0.265) | 0.385 |  | 0.428 (-0.344, 1.200) | 0.276 |
| Model 5 | -0.222 (-0.675, 0.231) | 0.335 |  | -0.145 (-0.620, 0.330) | 0.548 |  | 0.514 (-0.270, 1.298) | 0.197 |
| TBR (%): <3.0 mmol/L | | | | | | | | |
| Model 1 | -0.568 (-1.977, 0.841) | 0.428 |  | 0.236 (-1.203, 1.676) | 0.747 |  | 0.782 (-1.443, 3.007) | 0.489 |
| Model 2 | -0.216 (-1.621, 1.189) | 0.763 |  | 0.383 (-1.067, 1.833) | 0.603 |  | 0.867 (-1.393, 3.127) | 0.450 |
| Model 3 | -0.261 (-1.634, 1.113) | 0.709 |  | 0.416 (-1.026, 1.858) | 0.570 |  | 0.882 (-1.360, 3.124) | 0.439 |
| Model 4 | -0.749 (-2.172, 0.674) | 0.301 |  | -0.086 (-1.566, 1.393) | 0.909 |  | 0.371 (-2.007, 2.749) | 0.759 |
| Model 5 | -0.660 (-2.102, 0.781) | 0.368 |  | 0.227 (-1.260, 1.714) | 0.764 |  | 0.670 (-1.739, 3.079) | 0.584 |
| HBGI | | | | | | | | |
| Model 1 | -1.203 (-1.864, -0.543) | <0.001 |  | -1.065 (-1.747, -0.382) | 0.002 |  | -1.419 (-2.558, -0.280) | 0.015 |
| Model 2 | -1.206 (-1.855, -0.557) | <0.001 |  | -1.089 (-1.766, -0.413) | 0.002 |  | -1.423 (-2.567, -0.280) | 0.015 |
| Model 3 | -1.006 (-1.659, -0.354) | 0.003 |  | -0.957 (-1.649, -0.265) | 0.007 |  | -1.170 (-2.329, -0.012) | 0.048 |
| Model 4 | -1.415 (-2.353, -0.476) | 0.003 |  | -0.765 (-1.775, 0.246) | 0.137 |  | -1.199 (-2.932, 0.534) | 0.174 |
| Model 5 | -1.227 (-2.181, -0.273) | 0.012 |  | -0.468 (-1.491, 0.556) | 0.369 |  | -0.835 (-2.605, 0.936) | 0.353 |
| LBGI | | | | | | | | |
| Model 1 | -0.398 (-1.654, 0.858) | 0.533 |  | -0.135 (-1.443, 1.173) | 0.839 |  | 1.766 (-0.282, 3.813) | 0.091 |
| Model 2 | -0.044 (-1.299, 1.212) | 0.946 |  | 0.072 (-1.253, 1.398) | 0.915 |  | 1.874 (-0.204, 3.953) | 0.077 |
| Model 3 | -0.165 (-1.398, 1.067) | 0.792 |  | 0.103 (-1.222, 1.428) | 0.878 |  | 1.894 (-0.174, 3.963) | 0.072 |
| Model 4 | -0.764 (-2.102, 0.573) | 0.261 |  | -0.481 (-1.895, 0.934) | 0.504 |  | 1.442 (-0.884, 3.769) | 0.223 |
| Model 5 | -0.848 (-2.207, 0.511) | 0.220 |  | -0.282 (-1.708, 1.144) | 0.697 |  | 1.672 (-0.692, 4.036) | 0.165 |
| MODD (mmol/L) | | | | | | | | |
| Model 1 | -8.588 (-12.923, -4.253) | <0.001 |  | -7.152 (-11.663, -2.641) | 0.002 |  | -7.019 (-14.660, 0.622) | 0.072 |
| Model 2 | -8.737 (-12.994, -4.481) | <0.001 |  | -7.482 (-11.956, -3.008) | 0.001 |  | -7.249 (-14.938, 0.440) | 0.065 |
| Model 3 | -7.927 (-12.314, -3.539) | <0.001 |  | -6.449 (-11.126, -1.771) | 0.007 |  | -5.210 (-13.136, 2.716) | 0.196 |
| Model 4 | -9.065 (-14.475, -3.655) | 0.001 |  | -4.529 (-10.355, 1.298) | 0.127 |  | -2.487 (-12.460, 7.487) | 0.623 |
| Model 5 | -8.628 (-14.283, -2.974) | 0.003 |  | -2.650 (-8.721, 3.421) | 0.391 |  | -0.563 (-10.954, 9.827) | 0.915 |
| IQR (mmol/L) | | | | | | | | |
| Model 1 | -5.811 (-9.479, -2.142) | 0.002 |  | -4.600 (-8.398, -0.801) | 0.018 |  | -4.960 (-11.345, 1.426) | 0.127 |
| Model 2 | -6.257 (-9.862, -2.652) | <0.001 |  | -5.125 (-8.904, -1.347) | 0.008 |  | -5.254 (-11.700, 1.192) | 0.110 |
| Model 3 | -5.513 (-9.193, -1.833) | 0.003 |  | -4.210 (-8.125, -0.296) | 0.035 |  | -3.630 (-10.221, 2.962) | 0.279 |
| Model 4 | -6.125 (-10.809, -1.441) | 0.011 |  | -1.549 (-6.580, 3.482) | 0.545 |  | -0.993 (-9.540, 7.554) | 0.819 |
| Model 5 | -5.515 (-10.391, -0.640) | 0.027 |  | 0.043 (-5.162, 5.248) | 0.987 |  | 0.559 (-8.339, 9.457) | 0.901 |

Univariable and multivariable linear regression analysis. Model 1 was not adjusted. Model 2 was adjusted for age and sex. Model 3 was adjusted for BMI, duration of diabetes, and the covariates in Model 2. Model 4 was adjusted for smoking status, HbA1c, systolic blood pressure, total cholesterol, HDL cholesterol, log-transformed triglycerides, uric acid, and the covariates in Model 3. Model 5 was adjusted for eGFR, log-transformed urine albumin-to-creatinine ratio, and the covariates in Model 4.

GSM, gray-scale median; SD, standard deviation; CV, coefficient of variation; MAGE, mean amplitude of glycemic excursion; TIR, time in range; TAR, time above range; TBR, time below range; HBGI, high blood glucose index; LBGI, low blood glucose index; MODD, mean of daily differences; IQR, interquartile range

**Table S24 Associations of the metrics of continuous glucose monitoring with intima-media thickness among participants not using angiotensin-converting enzyme inhibitors or angiotensin II receptor blockers**

|  | mean-IMT (n = 334) | |  | CCA-max-IMT (n = 334) | |
| --- | --- | --- | --- | --- | --- |
|  | β (95% CI) | *P* value |  | β (95% CI) | *P* value |
| Mean glucose (mmol/L) | | | | | |
| Model 1 | -0.004 (-0.013, 0.005) | 0.415 |  | -0.004 (-0.025, 0.017) | 0.708 |
| Model 2 | -0.006 (-0.014, 0.003) | 0.190 |  | -0.007 (-0.028, 0.013) | 0.469 |
| Model 3 | -0.005 (-0.013, 0.004) | 0.255 |  | -0.007 (-0.027, 0.013) | 0.515 |
| Model 4 | 0.002 (-0.013, 0.016) | 0.818 |  | 0.000 (-0.034, 0.034) | 0.992 |
| Model 5 | 0.003 (-0.012, 0.017) | 0.728 |  | 0.002 (-0.032, 0.036) | 0.916 |
| Median glucose (mmol/L) | | | | | |
| Model 1 | -0.005 (-0.031, 0.022) | 0.712 |  | 0.002 (-0.058, 0.062) | 0.955 |
| Model 2 | -0.019 (-0.043, 0.006) | 0.131 |  | -0.017 (-0.076, 0.041) | 0.563 |
| Model 3 | -0.014 (-0.039, 0.011) | 0.280 |  | -0.004 (-0.063, 0.055) | 0.893 |
| Model 4 | -0.001 (-0.033, 0.031) | 0.934 |  | 0.028 (-0.046, 0.102) | 0.457 |
| Model 5 | -0.001 (-0.034, 0.031) | 0.930 |  | 0.026 (-0.049, 0.100) | 0.494 |
| SD (mmol/L) | | | | | |
| Model 1 | 0.001 (-0.002, 0.003) | 0.683 |  | 0.002 (-0.005, 0.008) | 0.596 |
| Model 2 | -0.001 (-0.004, 0.002) | 0.420 |  | 0.000 (-0.007, 0.006) | 0.913 |
| Model 3 | -0.001 (-0.003, 0.002) | 0.742 |  | 0.002 (-0.005, 0.008) | 0.640 |
| Model 4 | 0.000 (-0.003, 0.003) | 0.885 |  | 0.003 (-0.004, 0.009) | 0.394 |
| Model 5 | 0.000 (-0.003, 0.003) | 0.813 |  | 0.002 (-0.004, 0.009) | 0.479 |
| CV (%) | | | | | |
| Model 1 | 0.001 (-0.007, 0.009) | 0.784 |  | 0.006 (-0.013, 0.024) | 0.539 |
| Model 2 | -0.003 (-0.011, 0.004) | 0.377 |  | -0.001 (-0.019, 0.018) | 0.961 |
| Model 3 | -0.002 (-0.010, 0.005) | 0.575 |  | 0.003 (-0.015, 0.021) | 0.744 |
| Model 4 | 0.001 (-0.008, 0.010) | 0.758 |  | 0.012 (-0.009, 0.033) | 0.260 |
| Model 5 | 0.002 (-0.007, 0.011) | 0.707 |  | 0.012 (-0.009, 0.033) | 0.243 |
| MAGE (mmol/L) | | | | | |
| Model 1 | -0.004 (-0.013, 0.005) | 0.340 |  | -0.006 (-0.026, 0.015) | 0.594 |
| Model 2 | -0.006 (-0.014, 0.003) | 0.172 |  | -0.008 (-0.028, 0.011) | 0.403 |
| Model 3 | -0.005 (-0.013, 0.003) | 0.222 |  | -0.008 (-0.028, 0.011) | 0.408 |
| Model 4 | 0.000 (-0.014, 0.014) | 0.973 |  | -0.006 (-0.039, 0.027) | 0.703 |
| Model 5 | 0.001 (-0.013, 0.015) | 0.897 |  | -0.005 (-0.038, 0.028) | 0.770 |
| TIR (%): 3.9–10.0 mmol/L | | | | | |
| Model 1 | 0.004 (-0.005, 0.013) | 0.435 |  | 0.007 (-0.013, 0.028) | 0.479 |
| Model 2 | 0.006 (-0.002, 0.014) | 0.164 |  | 0.012 (-0.008, 0.031) | 0.254 |
| Model 3 | 0.005 (-0.004, 0.013) | 0.250 |  | 0.011 (-0.009, 0.030) | 0.296 |
| Model 4 | -0.002 (-0.015, 0.012) | 0.831 |  | 0.005 (-0.026, 0.037) | 0.731 |
| Model 5 | -0.002 (-0.015, 0.012) | 0.830 |  | 0.006 (-0.025, 0.037) | 0.711 |
| TAR (%): >10.0 mmol/L | | | | | |
| Model 1 | 0.000 (-0.001, 0.001) | 0.389 |  | -0.001 (-0.003, 0.001) | 0.503 |
| Model 2 | -0.001 (-0.001, 0.000) | 0.167 |  | -0.001 (-0.003, 0.001) | 0.301 |
| Model 3 | -0.001 (-0.001, 0.000) | 0.239 |  | -0.001 (-0.003, 0.001) | 0.336 |
| Model 4 | 0.000 (-0.001, 0.001) | 0.857 |  | -0.001 (-0.004, 0.003) | 0.765 |
| Model 5 | 0.000 (-0.001, 0.002) | 0.786 |  | 0.000 (-0.003, 0.003) | 0.812 |
| TAR (%): >13.9 mmol/L | | | | | |
| Model 1 | -0.001 (-0.003, 0.001) | 0.153 |  | -0.001 (-0.005, 0.003) | 0.594 |
| Model 2 | -0.002 (-0.003, 0.000) | 0.055 |  | -0.002 (-0.006, 0.002) | 0.409 |
| Model 3 | -0.002 (-0.003, 0.000) | 0.075 |  | -0.001 (-0.005, 0.003) | 0.514 |
| Model 4 | -0.001 (-0.003, 0.002) | 0.474 |  | 0.001 (-0.005, 0.007) | 0.692 |
| Model 5 | -0.001 (-0.004, 0.001) | 0.405 |  | 0.001 (-0.005, 0.007) | 0.759 |
| TBR (%): <3.9 mmol/L | | | | | |
| Model 1 | 0.001 (-0.003, 0.005) | 0.612 |  | 0.000 (-0.010, 0.009) | 0.940 |
| Model 2 | 0.000 (-0.004, 0.004) | 0.859 |  | -0.002 (-0.011, 0.008) | 0.734 |
| Model 3 | 0.001 (-0.003, 0.005) | 0.738 |  | -0.001 (-0.010, 0.008) | 0.822 |
| Model 4 | 0.000 (-0.004, 0.004) | 0.927 |  | -0.001 (-0.010, 0.009) | 0.905 |
| Model 5 | 0.000 (-0.005, 0.004) | 0.848 |  | -0.002 (-0.012, 0.008) | 0.695 |
| TBR (%): <3.0 mmol/L | | | | | |
| Model 1 | 0.003 (-0.012, 0.019) | 0.674 |  | -0.003 (-0.038, 0.033) | 0.874 |
| Model 2 | 0.001 (-0.014, 0.015) | 0.947 |  | -0.008 (-0.042, 0.026) | 0.647 |
| Model 3 | 0.002 (-0.012, 0.016) | 0.790 |  | -0.005 (-0.039, 0.029) | 0.756 |
| Model 4 | 0.001 (-0.013, 0.016) | 0.860 |  | -0.005 (-0.039, 0.029) | 0.786 |
| Model 5 | 0.000 (-0.015, 0.015) | 0.986 |  | -0.009 (-0.043, 0.026) | 0.613 |
| HBGI | | | | | |
| Model 1 | -0.002 (-0.005, 0.002) | 0.305 |  | -0.001 (-0.009, 0.006) | 0.730 |
| Model 2 | -0.003 (-0.006, 0.000) | 0.082 |  | -0.003 (-0.011, 0.005) | 0.439 |
| Model 3 | -0.002 (-0.006, 0.001) | 0.134 |  | -0.002 (-0.010, 0.005) | 0.584 |
| Model 4 | -0.001 (-0.006, 0.005) | 0.827 |  | 0.004 (-0.009, 0.016) | 0.585 |
| Model 5 | -0.001 (-0.006, 0.005) | 0.796 |  | 0.003 (-0.009, 0.016) | 0.623 |
| LBGI | | | | | |
| Model 1 | 0.001 (-0.011, 0.012) | 0.929 |  | -0.004 (-0.031, 0.023) | 0.779 |
| Model 2 | -0.001 (-0.012, 0.010) | 0.826 |  | -0.006 (-0.032, 0.020) | 0.652 |
| Model 3 | 0.000 (-0.011, 0.011) | 0.975 |  | -0.002 (-0.028, 0.023) | 0.852 |
| Model 4 | -0.002 (-0.014, 0.010) | 0.713 |  | -0.001 (-0.028, 0.027) | 0.966 |
| Model 5 | -0.004 (-0.017, 0.008) | 0.493 |  | -0.005 (-0.033, 0.023) | 0.735 |
| MODD (mmol/L) | | | | | |
| Model 1 | -0.022 (-0.047, 0.002) | 0.077 |  | -0.021 (-0.077, 0.035) | 0.459 |
| Model 2 | -0.022 (-0.045, 0.001) | 0.057 |  | -0.022 (-0.077, 0.032) | 0.419 |
| Model 3 | -0.018 (-0.041, 0.005) | 0.119 |  | -0.016 (-0.071, 0.038) | 0.561 |
| Model 4 | -0.008 (-0.040, 0.025) | 0.651 |  | 0.014 (-0.062, 0.089) | 0.724 |
| Model 5 | -0.008 (-0.041, 0.025) | 0.629 |  | 0.012 (-0.064, 0.088) | 0.759 |
| IQR (mmol/L) | | | | | |
| Model 1 | -0.021 (-0.040, -0.001) | 0.039 |  | -0.026 (-0.071, 0.019) | 0.249 |
| Model 2 | -0.020 (-0.038, -0.002) | 0.028 |  | -0.027 (-0.071, 0.016) | 0.215 |
| Model 3 | -0.017 (-0.036, 0.001) | 0.068 |  | -0.021 (-0.065, 0.023) | 0.341 |
| Model 4 | -0.010 (-0.035, 0.016) | 0.468 |  | -0.002 (-0.062, 0.058) | 0.941 |
| Model 5 | -0.010 (-0.036, 0.016) | 0.466 |  | -0.003 (-0.063, 0.057) | 0.925 |

Univariable and multivariable linear regression analysis. Model 1 was not adjusted. Model 2 was adjusted for age and sex. Model 3 was adjusted for BMI, duration of diabetes, and the covariates in Model 2. Model 4 was adjusted for smoking status, HbA1c, systolic blood pressure, total cholesterol, HDL cholesterol, log-transformed triglycerides, uric acid, and the covariates in Model 3. Model 5 was adjusted for eGFR, log-transformed urine albumin-to-creatinine ratio, and the covariates in Model 4.

IMT, intima-media thickness; SD, standard deviation; CV, coefficient of variation; MAGE, mean amplitude of glycemic excursion; TIR, time in range; TAR, time above range; TBR, time below range; HBGI, high blood glucose index; LBGI, low blood glucose index; MODD, mean of daily differences; IQR, interquartile range

**Table S25 Associations of the metrics of continuous glucose monitoring with gray-scale median among participants not using angiotensin-converting enzyme inhibitors or angiotensin II receptor blockers**

|  | mean-GSM  (n = 334) | |  | thickened lesion-GSM  (n = 316) | |  | plaque-GSM  (n = 275) | |
| --- | --- | --- | --- | --- | --- | --- | --- | --- |
|  | β (95 % CI) | *P* value |  | β (95 % CI) | *P* value |  | β (95 % CI) | *P* value |
| Mean glucose (mmol/L) | | | | | | | | |
| Model 1 | -1.690 (-2.805, -0.575) | 0.003 |  | -1.634 (-2.788, -0.481) | 0.006 |  | -2.332 (-4.175, -0.490) | 0.013 |
| Model 2 | -1.755 (-2.871, -0.639) | 0.002 |  | -1.453 (-2.580, -0.325) | 0.012 |  | -2.127 (-3.956, -0.297) | 0.023 |
| Model 3 | -1.624 (-2.711, -0.536) | 0.004 |  | -1.448 (-2.580, -0.316) | 0.012 |  | -2.102 (-3.935, -0.270) | 0.025 |
| Model 4 | -2.021 (-3.862, -0.181) | 0.031 |  | -1.723 (-3.700, 0.255) | 0.088 |  | -1.706 (-5.056, 1.644) | 0.317 |
| Model 5 | -1.717 (-3.554, 0.120) | 0.067 |  | -1.636 (-3.636, 0.365) | 0.109 |  | -1.645 (-5.040, 1.750) | 0.341 |
| Median glucose (mmol/L) | | | | | | | | |
| Model 1 | -4.150 (-7.376, -0.923) | 0.012 |  | -4.571 (-7.881, -1.261) | 0.007 |  | -7.352 (-12.781, -1.922) | 0.008 |
| Model 2 | -3.869 (-7.124, -0.615) | 0.020 |  | -3.558 (-6.817, -0.298) | 0.033 |  | -6.760 (-12.172, -1.348) | 0.015 |
| Model 3 | -4.024 (-7.227, -0.822) | 0.014 |  | -3.833 (-7.152, -0.515) | 0.024 |  | -7.193 (-12.691, -1.695) | 0.011 |
| Model 4 | -4.901 (-8.937, -0.864) | 0.017 |  | -4.053 (-8.357, 0.250) | 0.065 |  | -5.383 (-12.679, 1.913) | 0.147 |
| Model 5 | -4.464 (-8.486, -0.441) | 0.030 |  | -4.005 (-8.357, 0.347) | 0.071 |  | -5.780 (-13.159, 1.599) | 0.124 |
| SD (mmol/L) | | | | | | | | |
| Model 1 | -0.151 (-0.502, 0.201) | 0.400 |  | -0.178 (-0.541, 0.186) | 0.338 |  | -0.384 (-0.990, 0.223) | 0.214 |
| Model 2 | -0.091 (-0.448, 0.266) | 0.616 |  | -0.055 (-0.415, 0.305) | 0.764 |  | -0.329 (-0.939, 0.281) | 0.289 |
| Model 3 | -0.139 (-0.490, 0.213) | 0.439 |  | -0.084 (-0.452, 0.284) | 0.652 |  | -0.386 (-1.010, 0.238) | 0.225 |
| Model 4 | -0.275 (-0.635, 0.085) | 0.133 |  | -0.151 (-0.536, 0.235) | 0.443 |  | -0.289 (-0.949, 0.372) | 0.390 |
| Model 5 | -0.268 (-0.626, 0.090) | 0.142 |  | -0.154 (-0.544, 0.235) | 0.436 |  | -0.345 (-1.016, 0.326) | 0.312 |
| CV (%) | | | | | | | | |
| Model 1 | -1.449 (-2.444, -0.454) | 0.004 |  | -1.369 (-2.394, -0.343) | 0.009 |  | -1.962 (-3.643, -0.281) | 0.022 |
| Model 2 | -1.368 (-2.373, -0.363) | 0.008 |  | -1.048 (-2.059, -0.037) | 0.042 |  | -1.751 (-3.429, -0.074) | 0.041 |
| Model 3 | -1.454 (-2.432, -0.475) | 0.004 |  | -1.114 (-2.133, -0.095) | 0.032 |  | -1.852 (-3.542, -0.162) | 0.032 |
| Model 4 | -1.665 (-2.799, -0.531) | 0.004 |  | -1.093 (-2.317, 0.130) | 0.080 |  | -1.129 (-3.171, 0.913) | 0.277 |
| Model 5 | -1.525 (-2.653, -0.398) | 0.008 |  | -1.063 (-2.298, 0.171) | 0.091 |  | -1.174 (-3.233, 0.884) | 0.262 |
| MAGE (mmol/L) | | | | | | | | |
| Model 1 | -1.588 (-2.678, -0.499) | 0.004 |  | -1.483 (-2.614, -0.353) | 0.010 |  | -2.149 (-3.944, -0.353) | 0.019 |
| Model 2 | -1.664 (-2.755, -0.574) | 0.003 |  | -1.333 (-2.437, -0.228) | 0.018 |  | -1.961 (-3.744, -0.179) | 0.031 |
| Model 3 | -1.486 (-2.550, -0.421) | 0.006 |  | -1.311 (-2.421, -0.202) | 0.021 |  | -1.907 (-3.694, -0.121) | 0.037 |
| Model 4 | -1.611 (-3.411, 0.189) | 0.079 |  | -1.336 (-3.272, 0.600) | 0.175 |  | -1.172 (-4.423, 2.080) | 0.479 |
| Model 5 | -1.367 (-3.161, 0.426) | 0.135 |  | -1.237 (-3.194, 0.720) | 0.214 |  | -1.102 (-4.396, 2.192) | 0.511 |
| TIR (%): 3.9–10.0 mmol/L | | | | | | | | |
| Model 1 | 1.648 (0.554, 2.743) | 0.003 |  | 1.643 (0.503, 2.783) | 0.005 |  | 1.781 (-0.057, 3.618) | 0.058 |
| Model 2 | 1.706 (0.609, 2.802) | 0.002 |  | 1.408 (0.291, 2.525) | 0.014 |  | 1.545 (-0.281, 3.371) | 0.097 |
| Model 3 | 1.539 (0.463, 2.615) | 0.005 |  | 1.409 (0.282, 2.537) | 0.014 |  | 1.515 (-0.325, 3.355) | 0.106 |
| Model 4 | 2.090 (0.397, 3.783) | 0.016 |  | 1.750 (-0.063, 3.564) | 0.058 |  | -0.129 (-3.188, 2.929) | 0.934 |
| Model 5 | 2.014 (0.336, 3.693) | 0.019 |  | 1.727 (-0.098, 3.552) | 0.064 |  | -0.087 (-3.161, 2.987) | 0.956 |
| TAR (%): >10.0 mmol/L | | | | | | | | |
| Model 1 | -0.168 (-0.274, -0.062) | 0.002 |  | -0.167 (-0.277, -0.057) | 0.003 |  | -0.204 (-0.382, -0.026) | 0.025 |
| Model 2 | -0.173 (-0.279, -0.067) | 0.001 |  | -0.147 (-0.255, -0.040) | 0.008 |  | -0.185 (-0.361, -0.008) | 0.040 |
| Model 3 | -0.157 (-0.261, -0.054) | 0.003 |  | -0.147 (-0.256, -0.039) | 0.008 |  | -0.183 (-0.360, -0.006) | 0.043 |
| Model 4 | -0.206 (-0.372, -0.041) | 0.015 |  | -0.190 (-0.369, -0.012) | 0.036 |  | -0.093 (-0.394, 0.207) | 0.541 |
| Model 5 | -0.185 (-0.350, -0.020) | 0.028 |  | -0.186 (-0.365, -0.006) | 0.043 |  | -0.090 (-0.393, 0.212) | 0.557 |
| TAR (%): >13.9 mmol/L | | | | | | | | |
| Model 1 | -0.187 (-0.403, 0.030) | 0.091 |  | -0.203 (-0.425, 0.018) | 0.072 |  | -0.345 (-0.687, -0.002) | 0.049 |
| Model 2 | -0.194 (-0.410, 0.023) | 0.079 |  | -0.172 (-0.388, 0.044) | 0.118 |  | -0.314 (-0.654, 0.026) | 0.070 |
| Model 3 | -0.205 (-0.414, 0.005) | 0.055 |  | -0.181 (-0.397, 0.035) | 0.100 |  | -0.330 (-0.670, 0.010) | 0.057 |
| Model 4 | -0.184 (-0.503, 0.135) | 0.256 |  | -0.157 (-0.495, 0.181) | 0.361 |  | -0.065 (-0.613, 0.483) | 0.815 |
| Model 5 | -0.212 (-0.529, 0.105) | 0.189 |  | -0.151 (-0.493, 0.191) | 0.386 |  | -0.084 (-0.639, 0.470) | 0.765 |
| TBR (%): <3.9 mmol/L | | | | | | | | |
| Model 1 | 0.310 (-0.215, 0.836) | 0.246 |  | 0.298 (-0.247, 0.843) | 0.283 |  | 0.942 (0.035, 1.849) | 0.042 |
| Model 2 | 0.310 (-0.215, 0.834) | 0.247 |  | 0.368 (-0.161, 0.897) | 0.172 |  | 1.021 (0.126, 1.916) | 0.026 |
| Model 3 | 0.334 (-0.175, 0.842) | 0.198 |  | 0.379 (-0.151, 0.910) | 0.161 |  | 1.079 (0.178, 1.980) | 0.019 |
| Model 4 | 0.056 (-0.464, 0.575) | 0.833 |  | 0.203 (-0.354, 0.760) | 0.474 |  | 1.073 (0.126, 2.021) | 0.027 |
| Model 5 | -0.093 (-0.616, 0.429) | 0.725 |  | 0.178 (-0.391, 0.747) | 0.538 |  | 1.054 (0.074, 2.034) | 0.035 |
| TBR (%): <3.0 mmol/L | | | | | | | | |
| Model 1 | -0.968 (-2.892, 0.955) | 0.323 |  | -0.473 (-2.432, 1.486) | 0.635 |  | 0.252 (-3.330, 3.833) | 0.890 |
| Model 2 | -1.016 (-2.939, 0.906) | 0.299 |  | -0.213 (-2.119, 1.693) | 0.826 |  | 0.594 (-2.948, 4.137) | 0.741 |
| Model 3 | -0.920 (-2.788, 0.949) | 0.334 |  | -0.200 (-2.114, 1.714) | 0.837 |  | 0.831 (-2.742, 4.405) | 0.647 |
| Model 4 | -1.446 (-3.314, 0.422) | 0.129 |  | -0.462 (-2.432, 1.508) | 0.645 |  | 0.811 (-2.919, 4.542) | 0.669 |
| Model 5 | -1.602 (-3.465, 0.261) | 0.092 |  | -0.506 (-2.503, 1.492) | 0.619 |  | 0.480 (-3.336, 4.297) | 0.804 |
| HBGI | | | | | | | | |
| Model 1 | -0.560 (-0.981, -0.139) | 0.009 |  | -0.531 (-0.963, -0.098) | 0.016 |  | -0.796 (-1.476, -0.116) | 0.022 |
| Model 2 | -0.560 (-0.981, -0.139) | 0.009 |  | -0.439 (-0.862, -0.017) | 0.042 |  | -0.719 (-1.393, -0.044) | 0.037 |
| Model 3 | -0.557 (-0.967, -0.147) | 0.008 |  | -0.455 (-0.879, -0.031) | 0.036 |  | -0.744 (-1.421, -0.068) | 0.031 |
| Model 4 | -0.772 (-1.451, -0.092) | 0.026 |  | -0.556 (-1.283, 0.171) | 0.133 |  | -0.386 (-1.593, 0.821) | 0.529 |
| Model 5 | -0.747 (-1.422, -0.072) | 0.030 |  | -0.531 (-1.264, 0.203) | 0.156 |  | -0.423 (-1.641, 0.794) | 0.494 |
| LBGI | | | | | | | | |
| Model 1 | 1.202 (-0.248, 2.653) | 0.104 |  | 1.470 (-0.042, 2.983) | 0.057 |  | 1.880 (-0.659, 4.419) | 0.146 |
| Model 2 | 1.282 (-0.165, 2.729) | 0.082 |  | 1.620 (0.153, 3.086) | 0.031 |  | 1.910 (-0.601, 4.421) | 0.135 |
| Model 3 | 1.252 (-0.156, 2.659) | 0.081 |  | 1.612 (0.136, 3.087) | 0.032 |  | 1.969 (-0.574, 4.512) | 0.129 |
| Model 4 | 0.144 (-1.385, 1.674) | 0.853 |  | 1.013 (-0.645, 2.672) | 0.230 |  | 1.984 (-0.895, 4.863) | 0.176 |
| Model 5 | -0.258 (-1.797, 1.281) | 0.742 |  | 1.005 (-0.689, 2.698) | 0.244 |  | 1.805 (-1.190, 4.800) | 0.236 |
| MODD (mmol/L) | | | | | | | | |
| Model 1 | -3.570 (-6.586, -0.555) | 0.020 |  | -3.922 (-7.019, -0.825) | 0.013 |  | -7.780 (-12.788, -2.772) | 0.002 |
| Model 2 | -3.752 (-6.761, -0.743) | 0.015 |  | -3.800 (-6.809, -0.791) | 0.013 |  | -7.745 (-12.692, -2.799) | 0.002 |
| Model 3 | -3.356 (-6.328, -0.383) | 0.027 |  | -3.902 (-6.965, -0.839) | 0.013 |  | -7.822 (-12.855, -2.789) | 0.002 |
| Model 4 | -5.019 (-9.139, -0.898) | 0.017 |  | -4.525 (-8.916, -0.133) | 0.043 |  | -8.385 (-16.022, -0.749) | 0.032 |
| Model 5 | -4.804 (-8.898, -0.711) | 0.022 |  | -4.445 (-8.875, -0.015) | 0.049 |  | -8.724 (-16.423, -1.024) | 0.027 |
| IQR (mmol/L) | | | | | | | | |
| Model 1 | -2.448 (-4.869, -0.028) | 0.047 |  | -2.660 (-5.148, -0.171) | 0.036 |  | -5.869 (-9.884, -1.854) | 0.004 |
| Model 2 | -2.648 (-5.066, -0.229) | 0.032 |  | -2.568 (-4.989, -0.147) | 0.038 |  | -5.809 (-9.784, -1.834) | 0.004 |
| Model 3 | -2.450 (-4.837, -0.063) | 0.044 |  | -2.687 (-5.153, -0.221) | 0.033 |  | -5.911 (-9.959, -1.863) | 0.004 |
| Model 4 | -3.436 (-6.706, -0.167) | 0.039 |  | -2.882 (-6.353, 0.590) | 0.103 |  | -5.562 (-11.640, 0.517) | 0.073 |
| Model 5 | -3.215 (-6.460, 0.030) | 0.052 |  | -2.801 (-6.299, 0.697) | 0.116 |  | -5.720 (-11.842, 0.402) | 0.067 |

Univariable and multivariable linear regression analysis. Model 1 was not adjusted. Model 2 was adjusted for age and sex. Model 3 was adjusted for BMI, duration of diabetes, and the covariates in Model 2. Model 4 was adjusted for smoking status, HbA1c, systolic blood pressure, total cholesterol, HDL cholesterol, log-transformed triglycerides, uric acid, and the covariates in Model 3. Model 5 was adjusted for eGFR, log-transformed urine albumin-to-creatinine ratio, and the covariates in Model 4.

GSM, gray-scale median; SD, standard deviation; CV, coefficient of variation; MAGE, mean amplitude of glycemic excursion; TIR, time in range; TAR, time above range; TBR, time below range; HBGI, high blood glucose index; LBGI, low blood glucose index; MODD, mean of daily differences; IQR, interquartile range

**Table S26 Associations of the metrics of continuous glucose monitoring with intima-media thickness among participants using statins**

|  | mean-IMT (n = 320) | |  | CCA-max-IMT (n = 320) | |
| --- | --- | --- | --- | --- | --- |
|  | β (95% CI) | *P* value |  | β (95% CI) | *P* value |
| Mean glucose (mmol/L) | | | | | |
| Model 1 | -0.003 (-0.014, 0.008) | 0.564 |  | 0.010 (-0.022, 0.042) | 0.545 |
| Model 2 | -0.003 (-0.013, 0.007) | 0.567 |  | 0.011 (-0.020, 0.042) | 0.482 |
| Model 3 | -0.003 (-0.014, 0.007) | 0.569 |  | 0.008 (-0.023, 0.039) | 0.619 |
| Model 4 | 0.006 (-0.011, 0.023) | 0.477 |  | 0.017 (-0.033, 0.067) | 0.508 |
| Model 5 | 0.009 (-0.008, 0.026) | 0.315 |  | 0.021 (-0.030, 0.072) | 0.415 |
| Median glucose (mmol/L) | | | | | |
| Model 1 | -0.006 (-0.036, 0.024) | 0.703 |  | 0.046 (-0.044, 0.135) | 0.317 |
| Model 2 | -0.014 (-0.043, 0.016) | 0.363 |  | 0.027 (-0.060, 0.115) | 0.539 |
| Model 3 | -0.014 (-0.044, 0.016) | 0.352 |  | 0.024 (-0.066, 0.113) | 0.604 |
| Model 4 | -0.003 (-0.041, 0.035) | 0.871 |  | 0.035 (-0.077, 0.147) | 0.539 |
| Model 5 | 0.001 (-0.037, 0.039) | 0.956 |  | 0.041 (-0.073, 0.156) | 0.476 |
| SD (mmol/L) | | | | | |
| Model 1 | 0.000 (-0.003, 0.004) | 0.861 |  | 0.004 (-0.006, 0.014) | 0.415 |
| Model 2 | -0.001 (-0.004, 0.002) | 0.536 |  | 0.001 (-0.009, 0.011) | 0.875 |
| Model 3 | -0.001 (-0.004, 0.002) | 0.523 |  | 0.001 (-0.009, 0.011) | 0.816 |
| Model 4 | -0.001 (-0.004, 0.002) | 0.543 |  | 0.001 (-0.009, 0.011) | 0.833 |
| Model 5 | -0.001 (-0.004, 0.003) | 0.605 |  | 0.001 (-0.009, 0.012) | 0.802 |
| CV (%) | | | | | |
| Model 1 | 0.000 (-0.009, 0.009) | 0.966 |  | 0.022 (-0.006, 0.049) | 0.119 |
| Model 2 | -0.002 (-0.011, 0.007) | 0.611 |  | 0.016 (-0.011, 0.043) | 0.236 |
| Model 3 | -0.002 (-0.011, 0.007) | 0.604 |  | 0.018 (-0.009, 0.045) | 0.182 |
| Model 4 | 0.001 (-0.010, 0.011) | 0.925 |  | 0.029 (-0.003, 0.060) | 0.075 |
| Model 5 | 0.002 (-0.009, 0.013) | 0.704 |  | 0.032 (0.000, 0.064) | 0.050 |
| MAGE (mmol/L) | | | | | |
| Model 1 | -0.004 (-0.014, 0.007) | 0.483 |  | 0.007 (-0.024, 0.039) | 0.643 |
| Model 2 | -0.003 (-0.014, 0.007) | 0.515 |  | 0.009 (-0.021, 0.040) | 0.548 |
| Model 3 | -0.003 (-0.014, 0.007) | 0.517 |  | 0.005 (-0.026, 0.035) | 0.755 |
| Model 4 | 0.005 (-0.011, 0.022) | 0.545 |  | 0.009 (-0.040, 0.058) | 0.725 |
| Model 5 | 0.007 (-0.009, 0.024) | 0.385 |  | 0.013 (-0.037, 0.063) | 0.619 |
| TIR (%): 3.9–10.0 mmol/L | | | | | |
| Model 1 | 0.002 (-0.007, 0.012) | 0.624 |  | -0.004 (-0.033, 0.025) | 0.779 |
| Model 2 | 0.003 (-0.006, 0.013) | 0.520 |  | -0.003 (-0.032, 0.025) | 0.824 |
| Model 3 | 0.003 (-0.006, 0.013) | 0.521 |  | 0.001 (-0.027, 0.030) | 0.942 |
| Model 4 | -0.002 (-0.017, 0.012) | 0.751 |  | 0.008 (-0.035, 0.051) | 0.718 |
| Model 5 | -0.004 (-0.019, 0.010) | 0.575 |  | 0.005 (-0.039, 0.048) | 0.829 |
| TAR (%): >10.0 mmol/L | | | | | |
| Model 1 | 0.000 (-0.001, 0.001) | 0.492 |  | 0.001 (-0.002, 0.003) | 0.746 |
| Model 2 | 0.000 (-0.001, 0.001) | 0.461 |  | 0.001 (-0.002, 0.003) | 0.720 |
| Model 3 | 0.000 (-0.001, 0.001) | 0.462 |  | 0.000 (-0.003, 0.003) | 0.897 |
| Model 4 | 0.000 (-0.001, 0.002) | 0.750 |  | 0.000 (-0.004, 0.004) | 0.967 |
| Model 5 | 0.000 (-0.001, 0.002) | 0.562 |  | 0.000 (-0.004, 0.005) | 0.853 |
| TAR (%): >13.9 mmol/L | | | | | |
| Model 1 | -0.001 (-0.003, 0.001) | 0.355 |  | 0.001 (-0.006, 0.008) | 0.806 |
| Model 2 | -0.001 (-0.003, 0.001) | 0.374 |  | 0.001 (-0.005, 0.008) | 0.721 |
| Model 3 | -0.001 (-0.003, 0.001) | 0.377 |  | 0.001 (-0.006, 0.007) | 0.841 |
| Model 4 | 0.000 (-0.003, 0.003) | 0.884 |  | 0.001 (-0.009, 0.010) | 0.925 |
| Model 5 | 0.001 (-0.003, 0.004) | 0.743 |  | 0.001 (-0.009, 0.011) | 0.823 |
| TBR (%): <3.9 mmol/L | | | | | |
| Model 1 | 0.002 (-0.002, 0.007) | 0.309 |  | -0.002 (-0.015, 0.012) | 0.807 |
| Model 2 | 0.001 (-0.003, 0.006) | 0.589 |  | -0.005 (-0.018, 0.009) | 0.486 |
| Model 3 | 0.001 (-0.003, 0.006) | 0.582 |  | -0.007 (-0.020, 0.007) | 0.330 |
| Model 4 | 0.000 (-0.005, 0.005) | 0.989 |  | -0.009 (-0.023, 0.005) | 0.198 |
| Model 5 | 0.000 (-0.005, 0.004) | 0.939 |  | -0.009 (-0.023, 0.005) | 0.196 |
| TBR (%): <3.0 mmol/L | | | | | |
| Model 1 | 0.009 (-0.009, 0.027) | 0.317 |  | -0.006 (-0.059, 0.047) | 0.813 |
| Model 2 | 0.004 (-0.013, 0.021) | 0.638 |  | -0.018 (-0.070, 0.034) | 0.489 |
| Model 3 | 0.004 (-0.013, 0.022) | 0.630 |  | -0.027 (-0.079, 0.025) | 0.302 |
| Model 4 | 0.003 (-0.014, 0.021) | 0.700 |  | -0.024 (-0.075, 0.028) | 0.366 |
| Model 5 | 0.003 (-0.014, 0.020) | 0.730 |  | -0.025 (-0.077, 0.027) | 0.340 |
| HBGI | | | | | |
| Model 1 | -0.002 (-0.006, 0.003) | 0.405 |  | 0.004 (-0.009, 0.016) | 0.583 |
| Model 2 | -0.002 (-0.006, 0.002) | 0.364 |  | 0.004 (-0.009, 0.016) | 0.578 |
| Model 3 | -0.002 (-0.006, 0.002) | 0.363 |  | 0.003 (-0.010, 0.015) | 0.688 |
| Model 4 | 0.001 (-0.006, 0.008) | 0.828 |  | 0.006 (-0.014, 0.026) | 0.577 |
| Model 5 | 0.002 (-0.005, 0.008) | 0.633 |  | 0.007 (-0.013, 0.028) | 0.485 |
| LBGI | | | | | |
| Model 1 | 0.004 (-0.009, 0.016) | 0.582 |  | -0.009 (-0.047, 0.028) | 0.625 |
| Model 2 | 0.000 (-0.012, 0.013) | 0.945 |  | -0.018 (-0.055, 0.019) | 0.337 |
| Model 3 | 0.001 (-0.012, 0.013) | 0.941 |  | -0.022 (-0.059, 0.015) | 0.245 |
| Model 4 | -0.004 (-0.017, 0.009) | 0.576 |  | -0.028 (-0.066, 0.011) | 0.162 |
| Model 5 | -0.005 (-0.018, 0.009) | 0.494 |  | -0.030 (-0.069, 0.010) | 0.144 |
| MODD (mmol/L) | | | | | |
| Model 1 | -0.017 (-0.046, 0.013) | 0.263 |  | -0.010 (-0.098, 0.077) | 0.816 |
| Model 2 | -0.015 (-0.043, 0.014) | 0.314 |  | -0.004 (-0.089, 0.082) | 0.935 |
| Model 3 | -0.015 (-0.045, 0.014) | 0.303 |  | -0.014 (-0.101, 0.073) | 0.753 |
| Model 4 | -0.001 (-0.040, 0.038) | 0.964 |  | -0.024 (-0.141, 0.092) | 0.681 |
| Model 5 | 0.002 (-0.037, 0.042) | 0.908 |  | -0.022 (-0.141, 0.097) | 0.712 |
| IQR (mmol/L) | | | | | |
| Model 1 | -0.019 (-0.044, 0.006) | 0.132 |  | -0.020 (-0.093, 0.054) | 0.598 |
| Model 2 | -0.015 (-0.039, 0.009) | 0.224 |  | -0.009 (-0.081, 0.064) | 0.817 |
| Model 3 | -0.016 (-0.040, 0.009) | 0.214 |  | -0.019 (-0.092, 0.055) | 0.619 |
| Model 4 | -0.007 (-0.040, 0.026) | 0.670 |  | -0.043 (-0.141, 0.056) | 0.393 |
| Model 5 | -0.004 (-0.037, 0.030) | 0.838 |  | -0.041 (-0.141, 0.060) | 0.424 |

Univariable and multivariable linear regression analysis. Model 1 was not adjusted. Model 2 was adjusted for age and sex. Model 3 was adjusted for BMI, duration of diabetes, and the covariates in Model 2. Model 4 was adjusted for smoking status, HbA1c, systolic blood pressure, total cholesterol, HDL cholesterol, log-transformed triglycerides, uric acid, and the covariates in Model 3. Model 5 was adjusted for eGFR, log-transformed urine albumin-to-creatinine ratio, and the covariates in Model 4.

IMT, intima-media thickness; SD, standard deviation; CV, coefficient of variation; MAGE, mean amplitude of glycemic excursion; TIR, time in range; TAR, time above range; TBR, time below range; HBGI, high blood glucose index; LBGI, low blood glucose index; MODD, mean of daily differences; IQR, interquartile range

**Table S27 Associations of the metrics of continuous glucose monitoring with gray-scale median among participants using statins**

|  | mean-GSM  (n = 319) | |  | thickened lesion-GSM  (n = 305) | |  | plaque-GSM  (n = 268) | |
| --- | --- | --- | --- | --- | --- | --- | --- | --- |
|  | β (95 % CI) | *P* value |  | β (95 % CI) | *P* value |  | β (95 % CI) | *P* value |
| Mean glucose (mmol/L) | | | | | | | | |
| Model 1 | -1.665 (-2.778, -0.552) | 0.004 |  | -1.731 (-3.008, -0.455) | 0.008 |  | -3.026 (-5.133, -0.918) | 0.005 |
| Model 2 | -1.745 (-2.862, -0.629) | 0.002 |  | -1.633 (-2.898, -0.368) | 0.012 |  | -2.936 (-5.035, -0.836) | 0.006 |
| Model 3 | -1.610 (-2.727, -0.493) | 0.005 |  | -1.613 (-2.890, -0.336) | 0.013 |  | -2.819 (-4.939, -0.698) | 0.009 |
| Model 4 | -0.837 (-2.680, 1.006) | 0.372 |  | -0.190 (-2.287, 1.906) | 0.858 |  | -1.386 (-5.092, 2.321) | 0.462 |
| Model 5 | -0.432 (-2.274, 1.410) | 0.645 |  | 0.022 (-2.069, 2.114) | 0.983 |  | -0.847 (-4.542, 2.849) | 0.652 |
| Median glucose (mmol/L) | | | | | | | | |
| Model 1 | -5.185 (-8.310, -2.061) | 0.001 |  | -4.932 (-8.500, -1.365) | 0.007 |  | -6.657 (-12.718, -0.595) | 0.031 |
| Model 2 | -5.048 (-8.193, -1.903) | 0.002 |  | -4.611 (-8.140, -1.082) | 0.011 |  | -6.481 (-12.510, -0.451) | 0.035 |
| Model 3 | -4.857 (-8.065, -1.650) | 0.003 |  | -4.524 (-8.164, -0.884) | 0.015 |  | -6.185 (-12.419, 0.049) | 0.052 |
| Model 4 | -3.511 (-7.638, 0.616) | 0.095 |  | -2.255 (-6.959, 2.449) | 0.346 |  | -1.198 (-9.596, 7.200) | 0.779 |
| Model 5 | -3.088 (-7.219, 1.044) | 0.142 |  | -1.233 (-5.945, 3.479) | 0.607 |  | -0.262 (-8.667, 8.143) | 0.951 |
| SD (mmol/L) | | | | | | | | |
| Model 1 | -0.317 (-0.660, 0.027) | 0.070 |  | -0.266 (-0.660, 0.128) | 0.185 |  | -0.105 (-0.767, 0.556) | 0.754 |
| Model 2 | -0.282 (-0.633, 0.068) | 0.114 |  | -0.241 (-0.635, 0.153) | 0.230 |  | -0.092 (-0.757, 0.574) | 0.787 |
| Model 3 | -0.277 (-0.634, 0.080) | 0.127 |  | -0.212 (-0.620, 0.196) | 0.308 |  | -0.043 (-0.729, 0.643) | 0.902 |
| Model 4 | -0.265 (-0.635, 0.105) | 0.159 |  | -0.174 (-0.594, 0.246) | 0.415 |  | 0.067 (-0.660, 0.794) | 0.856 |
| Model 5 | -0.273 (-0.643, 0.097) | 0.147 |  | -0.085 (-0.505, 0.334) | 0.689 |  | 0.111 (-0.616, 0.838) | 0.764 |
| CV (%) | | | | | | | | |
| Model 1 | -1.781 (-2.725, -0.837) | <0.001 |  | -1.630 (-2.703, -0.556) | 0.003 |  | -1.905 (-3.732, -0.079) | 0.041 |
| Model 2 | -1.770 (-2.719, -0.820) | <0.001 |  | -1.473 (-2.537, -0.409) | 0.007 |  | -1.750 (-3.568, 0.069) | 0.059 |
| Model 3 | -1.809 (-2.759, -0.859) | <0.001 |  | -1.434 (-2.513, -0.354) | 0.009 |  | -1.742 (-3.588, 0.105) | 0.064 |
| Model 4 | -1.551 (-2.704, -0.398) | 0.009 |  | -0.916 (-2.231, 0.400) | 0.172 |  | -0.507 (-2.793, 1.778) | 0.662 |
| Model 5 | -1.421 (-2.578, -0.263) | 0.016 |  | -0.646 (-1.967, 0.675) | 0.337 |  | -0.305 (-2.601, 1.992) | 0.794 |
| MAGE (mmol/L) | | | | | | | | |
| Model 1 | -1.599 (-2.691, -0.508) | 0.004 |  | -1.669 (-2.923, -0.415) | 0.009 |  | -2.893 (-4.953, -0.832) | 0.006 |
| Model 2 | -1.690 (-2.785, -0.594) | 0.003 |  | -1.580 (-2.823, -0.336) | 0.013 |  | -2.803 (-4.857, -0.750) | 0.008 |
| Model 3 | -1.522 (-2.622, -0.422) | 0.007 |  | -1.568 (-2.826, -0.311) | 0.015 |  | -2.669 (-4.748, -0.590) | 0.012 |
| Model 4 | -0.587 (-2.399, 1.224) | 0.524 |  | -0.062 (-2.121, 1.997) | 0.953 |  | -1.003 (-4.622, 2.616) | 0.586 |
| Model 5 | -0.244 (-2.052, 1.564) | 0.791 |  | 0.042 (-2.006, 2.090) | 0.968 |  | -0.584 (-4.178, 3.011) | 0.749 |
| TIR (%): 3.9–10.0 mmol/L | | | | | | | | |
| Model 1 | 1.515 (0.498, 2.532) | 0.004 |  | 1.475 (0.303, 2.647) | 0.014 |  | 1.766 (-0.194, 3.726) | 0.077 |
| Model 2 | 1.548 (0.530, 2.566) | 0.003 |  | 1.349 (0.190, 2.508) | 0.023 |  | 1.647 (-0.303, 3.596) | 0.097 |
| Model 3 | 1.391 (0.366, 2.416) | 0.008 |  | 1.330 (0.154, 2.506) | 0.027 |  | 1.499 (-0.476, 3.473) | 0.136 |
| Model 4 | 0.723 (-0.861, 2.306) | 0.370 |  | 0.035 (-1.768, 1.838) | 0.970 |  | -1.293 (-4.396, 1.811) | 0.413 |
| Model 5 | 0.541 (-1.037, 2.119) | 0.500 |  | -0.195 (-1.989, 1.600) | 0.831 |  | -1.576 (-4.667, 1.514) | 0.316 |
| TAR (%): >10.0 mmol/L | | | | | | | | |
| Model 1 | -0.145 (-0.244, -0.046) | 0.004 |  | -0.144 (-0.259, -0.030) | 0.013 |  | -0.227 (-0.417, -0.037) | 0.019 |
| Model 2 | -0.149 (-0.248, -0.050) | 0.003 |  | -0.135 (-0.248, -0.022) | 0.020 |  | -0.218 (-0.407, -0.029) | 0.024 |
| Model 3 | -0.136 (-0.236, -0.037) | 0.007 |  | -0.133 (-0.247, -0.019) | 0.023 |  | -0.207 (-0.397, -0.016) | 0.034 |
| Model 4 | -0.070 (-0.227, 0.087) | 0.384 |  | -0.007 (-0.186, 0.172) | 0.935 |  | -0.011 (-0.323, 0.301) | 0.945 |
| Model 5 | -0.043 (-0.200, 0.113) | 0.587 |  | 0.014 (-0.164, 0.192) | 0.875 |  | 0.023 (-0.287, 0.333) | 0.883 |
| TAR (%): >13.9 mmol/L | | | | | | | | |
| Model 1 | -0.266 (-0.499, -0.032) | 0.026 |  | -0.269 (-0.535, -0.004) | 0.046 |  | -0.360 (-0.793, 0.074) | 0.104 |
| Model 2 | -0.279 (-0.513, -0.045) | 0.020 |  | -0.257 (-0.519, 0.005) | 0.055 |  | -0.353 (-0.785, 0.078) | 0.108 |
| Model 3 | -0.258 (-0.491, -0.026) | 0.030 |  | -0.254 (-0.518, 0.009) | 0.058 |  | -0.337 (-0.770, 0.096) | 0.127 |
| Model 4 | -0.045 (-0.395, 0.306) | 0.801 |  | 0.047 (-0.348, 0.442) | 0.815 |  | 0.230 (-0.449, 0.908) | 0.505 |
| Model 5 | -0.017 (-0.364, 0.331) | 0.924 |  | 0.047 (-0.344, 0.438) | 0.813 |  | 0.251 (-0.422, 0.923) | 0.464 |
| TBR (%): <3.9 mmol/L | | | | | | | | |
| Model 1 | 0.027 (-0.459, 0.513) | 0.913 |  | 0.103 (-0.446, 0.652) | 0.712 |  | 1.324 (0.434, 2.213) | 0.004 |
| Model 2 | 0.062 (-0.427, 0.551) | 0.802 |  | 0.152 (-0.392, 0.695) | 0.584 |  | 1.380 (0.495, 2.264) | 0.002 |
| Model 3 | 0.140 (-0.348, 0.628) | 0.572 |  | 0.174 (-0.376, 0.723) | 0.535 |  | 1.449 (0.561, 2.336) | 0.001 |
| Model 4 | -0.015 (-0.519, 0.490) | 0.955 |  | 0.040 (-0.527, 0.607) | 0.890 |  | 1.332 (0.389, 2.276) | 0.006 |
| Model 5 | -0.104 (-0.608, 0.401) | 0.687 |  | 0.049 (-0.518, 0.617) | 0.864 |  | 1.311 (0.359, 2.263) | 0.007 |
| TBR (%): <3.0 mmol/L | | | | | | | | |
| Model 1 | -0.724 (-2.595, 1.148) | 0.447 |  | 0.092 (-2.019, 2.204) | 0.932 |  | 2.756 (-0.603, 6.115) | 0.107 |
| Model 2 | -0.656 (-2.539, 1.226) | 0.493 |  | 0.406 (-1.683, 2.494) | 0.703 |  | 3.145 (-0.202, 6.492) | 0.065 |
| Model 3 | -0.335 (-2.214, 1.545) | 0.726 |  | 0.443 (-1.665, 2.551) | 0.679 |  | 3.448 (0.082, 6.814) | 0.045 |
| Model 4 | -0.605 (-2.494, 1.284) | 0.529 |  | 0.178 (-1.938, 2.294) | 0.868 |  | 3.201 (-0.254, 6.655) | 0.069 |
| Model 5 | -0.760 (-2.643, 1.123) | 0.428 |  | 0.316 (-1.794, 2.425) | 0.768 |  | 3.265 (-0.198, 6.729) | 0.065 |
| HBGI | | | | | | | | |
| Model 1 | -0.713 (-1.156, -0.269) | 0.002 |  | -0.710 (-1.217, -0.204) | 0.006 |  | -1.071 (-1.908, -0.235) | 0.012 |
| Model 2 | -0.722 (-1.166, -0.279) | 0.001 |  | -0.682 (-1.181, -0.182) | 0.008 |  | -1.055 (-1.886, -0.225) | 0.013 |
| Model 3 | -0.679 (-1.123, -0.234) | 0.003 |  | -0.670 (-1.175, -0.165) | 0.010 |  | -1.014 (-1.853, -0.175) | 0.018 |
| Model 4 | -0.482 (-1.219, 0.255) | 0.199 |  | -0.230 (-1.069, 0.609) | 0.590 |  | -0.273 (-1.757, 1.211) | 0.718 |
| Model 5 | -0.384 (-1.119, 0.351) | 0.305 |  | -0.112 (-0.948, 0.725) | 0.793 |  | -0.093 (-1.571, 1.386) | 0.902 |
| LBGI | | | | | | | | |
| Model 1 | 0.314 (-1.022, 1.650) | 0.644 |  | 0.512 (-1.006, 2.031) | 0.507 |  | 3.091 (0.637, 5.545) | 0.014 |
| Model 2 | 0.450 (-0.899, 1.798) | 0.512 |  | 0.573 (-0.936, 2.081) | 0.456 |  | 3.115 (0.664, 5.565) | 0.013 |
| Model 3 | 0.604 (-0.737, 1.944) | 0.376 |  | 0.625 (-0.895, 2.145) | 0.419 |  | 3.254 (0.799, 5.709) | 0.010 |
| Model 4 | -0.047 (-1.482, 1.388) | 0.948 |  | 0.021 (-1.599, 1.642) | 0.979 |  | 2.771 (0.072, 5.470) | 0.044 |
| Model 5 | -0.370 (-1.812, 1.072) | 0.614 |  | 0.109 (-1.516, 1.734) | 0.895 |  | 2.674 (-0.055, 5.402) | 0.055 |
| MODD (mmol/L) | | | | | | | | |
| Model 1 | -4.993 (-8.050, -1.936) | 0.001 |  | -5.352 (-8.813, -1.891) | 0.003 |  | -6.743 (-12.606, -0.880) | 0.024 |
| Model 2 | -5.144 (-8.204, -2.084) | 0.001 |  | -5.394 (-8.805, -1.982) | 0.002 |  | -7.078 (-12.909, -1.246) | 0.018 |
| Model 3 | -4.827 (-7.958, -1.697) | 0.003 |  | -5.391 (-8.913, -1.868) | 0.003 |  | -6.718 (-12.733, -0.703) | 0.029 |
| Model 4 | -3.902 (-8.185, 0.381) | 0.074 |  | -2.857 (-7.724, 2.011) | 0.249 |  | -1.136 (-9.865, 7.594) | 0.798 |
| Model 5 | -3.910 (-8.214, 0.393) | 0.075 |  | -2.003 (-6.893, 2.886) | 0.421 |  | -0.421 (-9.174, 8.333) | 0.925 |
| IQR (mmol/L) | | | | | | | | |
| Model 1 | -3.676 (-6.256, -1.096) | 0.005 |  | -3.644 (-6.575, -0.712) | 0.015 |  | -4.472 (-9.425, 0.482) | 0.077 |
| Model 2 | -3.858 (-6.445, -1.270) | 0.004 |  | -3.797 (-6.691, -0.904) | 0.010 |  | -4.849 (-9.790, 0.093) | 0.054 |
| Model 3 | -3.527 (-6.170, -0.885) | 0.009 |  | -3.748 (-6.730, -0.766) | 0.014 |  | -4.443 (-9.532, 0.646) | 0.087 |
| Model 4 | -2.249 (-5.879, 1.380) | 0.224 |  | -0.967 (-5.078, 3.145) | 0.644 |  | 1.037 (-6.325, 8.399) | 0.782 |
| Model 5 | -2.015 (-5.654, 1.624) | 0.277 |  | -0.190 (-4.313, 3.933) | 0.928 |  | 1.840 (-5.534, 9.214) | 0.623 |

Univariable and multivariable linear regression analysis. Model 1 was not adjusted. Model 2 was adjusted for age and sex. Model 3 was adjusted for BMI, duration of diabetes, and the covariates in Model 2. Model 4 was adjusted for smoking status, HbA1c, systolic blood pressure, total cholesterol, HDL cholesterol, log-transformed triglycerides, uric acid, and the covariates in Model 3. Model 5 was adjusted for eGFR, log-transformed urine albumin-to-creatinine ratio, and the covariates in Model 4.

GSM, gray-scale median; SD, standard deviation; CV, coefficient of variation; MAGE, mean amplitude of glycemic excursion; TIR, time in range; TAR, time above range; TBR, time below range; HBGI, high blood glucose index; LBGI, low blood glucose index; MODD, mean of daily differences; IQR, interquartile range

**Table S28 Associations of the metrics of continuous glucose monitoring with intima-media thickness among participants not using statins**

|  | mean-IMT (n = 278) | |  | CCA-max-IMT (n = 278) | |
| --- | --- | --- | --- | --- | --- |
|  | β (95% CI) | *P* value |  | β (95% CI) | *P* value |
| Mean glucose (mmol/L) | | | | | |
| Model 1 | 0.001 (-0.009, 0.011) | 0.851 |  | -0.002 (-0.029, 0.025) | 0.873 |
| Model 2 | -0.001 (-0.010, 0.008) | 0.881 |  | -0.006 (-0.032, 0.020) | 0.666 |
| Model 3 | -0.001 (-0.009, 0.008) | 0.908 |  | -0.006 (-0.032, 0.020) | 0.663 |
| Model 4 | 0.002 (-0.013, 0.017) | 0.768 |  | 0.009 (-0.034, 0.053) | 0.673 |
| Model 5 | 0.002 (-0.013, 0.017) | 0.768 |  | 0.012 (-0.033, 0.056) | 0.609 |
| Median glucose (mmol/L) | | | | | |
| Model 1 | 0.011 (-0.017, 0.038) | 0.445 |  | 0.024 (-0.051, 0.099) | 0.528 |
| Model 2 | -0.002 (-0.027, 0.022) | 0.854 |  | 0.003 (-0.070, 0.076) | 0.936 |
| Model 3 | -0.002 (-0.027, 0.024) | 0.892 |  | 0.008 (-0.067, 0.082) | 0.841 |
| Model 4 | 0.001 (-0.029, 0.031) | 0.951 |  | 0.033 (-0.054, 0.120) | 0.455 |
| Model 5 | 0.001 (-0.029, 0.031) | 0.951 |  | 0.033 (-0.056, 0.122) | 0.465 |
| SD (mmol/L) | | | | | |
| Model 1 | 0.002 (-0.001, 0.004) | 0.259 |  | 0.005 (-0.002, 0.012) | 0.148 |
| Model 2 | 0.000 (-0.002, 0.003) | 0.874 |  | 0.003 (-0.004, 0.010) | 0.352 |
| Model 3 | 0.000 (-0.002, 0.003) | 0.842 |  | 0.004 (-0.003, 0.011) | 0.265 |
| Model 4 | 0.000 (-0.002, 0.003) | 0.897 |  | 0.004 (-0.003, 0.011) | 0.292 |
| Model 5 | 0.000 (-0.002, 0.003) | 0.897 |  | 0.004 (-0.004, 0.012) | 0.314 |
| CV (%) | | | | | |
| Model 1 | 0.004 (-0.005, 0.012) | 0.394 |  | 0.002 (-0.021, 0.025) | 0.860 |
| Model 2 | -0.001 (-0.008, 0.007) | 0.894 |  | -0.005 (-0.027, 0.017) | 0.660 |
| Model 3 | 0.000 (-0.008, 0.007) | 0.925 |  | -0.004 (-0.027, 0.019) | 0.725 |
| Model 4 | 0.001 (-0.008, 0.009) | 0.878 |  | 0.000 (-0.026, 0.025) | 0.995 |
| Model 5 | 0.000 (-0.009, 0.009) | 0.935 |  | 0.000 (-0.027, 0.026) | 0.975 |
| MAGE (mmol/L) | | | | | |
| Model 1 | 0.001 (-0.009, 0.010) | 0.861 |  | -0.003 (-0.029, 0.023) | 0.823 |
| Model 2 | -0.001 (-0.009, 0.008) | 0.886 |  | -0.006 (-0.031, 0.019) | 0.630 |
| Model 3 | -0.001 (-0.009, 0.008) | 0.915 |  | -0.006 (-0.032, 0.019) | 0.620 |
| Model 4 | 0.002 (-0.012, 0.016) | 0.782 |  | 0.006 (-0.036, 0.048) | 0.782 |
| Model 5 | 0.002 (-0.013, 0.017) | 0.807 |  | 0.008 (-0.036, 0.052) | 0.713 |
| TIR (%): 3.9–10.0 mmol/L | | | | | |
| Model 1 | -0.005 (-0.014, 0.005) | 0.337 |  | -0.016 (-0.043, 0.010) | 0.227 |
| Model 2 | -0.002 (-0.010, 0.007) | 0.728 |  | -0.011 (-0.036, 0.015) | 0.416 |
| Model 3 | -0.002 (-0.011, 0.007) | 0.687 |  | -0.011 (-0.037, 0.015) | 0.398 |
| Model 4 | -0.005 (-0.018, 0.007) | 0.415 |  | -0.037 (-0.074, -0.001) | 0.045 |
| Model 5 | -0.004 (-0.017, 0.009) | 0.521 |  | -0.038 (-0.076, -0.001) | 0.046 |
| TAR (%): >10.0 mmol/L | | | | | |
| Model 1 | 0.000 (-0.001, 0.001) | 0.699 |  | 0.000 (-0.002, 0.003) | 0.853 |
| Model 2 | 0.000 (-0.001, 0.001) | 0.919 |  | 0.000 (-0.003, 0.002) | 0.875 |
| Model 3 | 0.000 (-0.001, 0.001) | 0.953 |  | 0.000 (-0.003, 0.002) | 0.873 |
| Model 4 | 0.000 (-0.001, 0.002) | 0.729 |  | 0.002 (-0.002, 0.006) | 0.375 |
| Model 5 | 0.000 (-0.001, 0.002) | 0.778 |  | 0.002 (-0.002, 0.006) | 0.364 |
| TAR (%): >13.9 mmol/L | | | | | |
| Model 1 | -0.001 (-0.003, 0.001) | 0.522 |  | -0.001 (-0.007, 0.004) | 0.664 |
| Model 2 | -0.001 (-0.003, 0.001) | 0.282 |  | -0.002 (-0.007, 0.004) | 0.485 |
| Model 3 | -0.001 (-0.003, 0.001) | 0.291 |  | -0.002 (-0.007, 0.004) | 0.502 |
| Model 4 | -0.001 (-0.003, 0.002) | 0.527 |  | 0.001 (-0.007, 0.009) | 0.825 |
| Model 5 | -0.001 (-0.004, 0.002) | 0.409 |  | 0.001 (-0.007, 0.009) | 0.831 |
| TBR (%): <3.9 mmol/L | | | | | |
| Model 1 | 0.003 (0.000, 0.006) | 0.079 |  | 0.013 (0.005, 0.021) | 0.001 |
| Model 2 | 0.002 (-0.001, 0.005) | 0.161 |  | 0.012 (0.004, 0.020) | 0.002 |
| Model 3 | 0.002 (-0.001, 0.005) | 0.154 |  | 0.013 (0.005, 0.020) | 0.002 |
| Model 4 | 0.002 (-0.001, 0.005) | 0.244 |  | 0.012 (0.004, 0.021) | 0.005 |
| Model 5 | 0.001 (-0.002, 0.004) | 0.362 |  | 0.012 (0.004, 0.021) | 0.006 |
| TBR (%): <3.0 mmol/L | | | | | |
| Model 1 | 0.007 (-0.003, 0.016) | 0.163 |  | 0.042 (0.016, 0.068) | 0.001 |
| Model 2 | 0.006 (-0.003, 0.015) | 0.167 |  | 0.043 (0.018, 0.068) | <0.001 |
| Model 3 | 0.006 (-0.002, 0.015) | 0.153 |  | 0.045 (0.019, 0.070) | <0.001 |
| Model 4 | 0.006 (-0.003, 0.015) | 0.217 |  | 0.043 (0.017, 0.069) | 0.001 |
| Model 5 | 0.005 (-0.005, 0.014) | 0.302 |  | 0.045 (0.017, 0.072) | 0.001 |
| HBGI | | | | | |
| Model 1 | 0.000 (-0.004, 0.004) | 0.950 |  | 0.000 (-0.011, 0.010) | 0.978 |
| Model 2 | -0.001 (-0.005, 0.002) | 0.557 |  | -0.002 (-0.012, 0.008) | 0.662 |
| Model 3 | -0.001 (-0.004, 0.003) | 0.579 |  | -0.002 (-0.012, 0.008) | 0.688 |
| Model 4 | 0.000 (-0.005, 0.005) | 0.902 |  | 0.004 (-0.011, 0.019) | 0.568 |
| Model 5 | -0.001 (-0.006, 0.005) | 0.820 |  | 0.005 (-0.011, 0.020) | 0.558 |
| LBGI | | | | | |
| Model 1 | 0.003 (-0.006, 0.011) | 0.563 |  | 0.020 (-0.004, 0.044) | 0.094 |
| Model 2 | 0.001 (-0.007, 0.009) | 0.858 |  | 0.019 (-0.005, 0.042) | 0.118 |
| Model 3 | 0.001 (-0.007, 0.009) | 0.826 |  | 0.021 (-0.003, 0.044) | 0.089 |
| Model 4 | 0.000 (-0.009, 0.009) | 0.930 |  | 0.020 (-0.006, 0.046) | 0.130 |
| Model 5 | -0.001 (-0.010, 0.009) | 0.865 |  | 0.020 (-0.007, 0.047) | 0.150 |
| MODD (mmol/L) | | | | | |
| Model 1 | 0.003 (-0.024, 0.029) | 0.853 |  | 0.005 (-0.068, 0.079) | 0.891 |
| Model 2 | 0.003 (-0.022, 0.027) | 0.834 |  | 0.004 (-0.067, 0.075) | 0.907 |
| Model 3 | 0.004 (-0.021, 0.029) | 0.776 |  | 0.008 (-0.065, 0.081) | 0.833 |
| Model 4 | 0.013 (-0.019, 0.044) | 0.442 |  | 0.048 (-0.046, 0.142) | 0.312 |
| Model 5 | 0.010 (-0.023, 0.043) | 0.543 |  | 0.049 (-0.048, 0.145) | 0.322 |
| IQR (mmol/L) | | | | | |
| Model 1 | -0.005 (-0.026, 0.016) | 0.666 |  | -0.005 (-0.062, 0.053) | 0.871 |
| Model 2 | -0.004 (-0.023, 0.015) | 0.659 |  | -0.006 (-0.062, 0.050) | 0.837 |
| Model 3 | -0.004 (-0.023, 0.016) | 0.700 |  | -0.003 (-0.060, 0.054) | 0.916 |
| Model 4 | 0.001 (-0.025, 0.026) | 0.957 |  | 0.028 (-0.047, 0.103) | 0.464 |
| Model 5 | -0.001 (-0.027, 0.025) | 0.968 |  | 0.029 (-0.048, 0.105) | 0.465 |

Univariable and multivariable linear regression analysis. Model 1 was not adjusted. Model 2 was adjusted for age and sex. Model 3 was adjusted for BMI, duration of diabetes, and the covariates in Model 2. Model 4 was adjusted for smoking status, HbA1c, systolic blood pressure, total cholesterol, HDL cholesterol, log-transformed triglycerides, uric acid, and the covariates in Model 3. Model 5 was adjusted for eGFR, log-transformed urine albumin-to-creatinine ratio, and the covariates in Model 4.

IMT, intima-media thickness; SD, standard deviation; CV, coefficient of variation; MAGE, mean amplitude of glycemic excursion; TIR, time in range; TAR, time above range; TBR, time below range; HBGI, high blood glucose index; LBGI, low blood glucose index; MODD, mean of daily differences; IQR, interquartile range

**Table S29 Associations of the metrics of continuous glucose monitoring with gray-scale median among participants not using statins**

|  | mean-GSM  (n = 278) | |  | thickened lesion-GSM  (n = 259) | |  | plaque-GSM  (n = 220) | |
| --- | --- | --- | --- | --- | --- | --- | --- | --- |
|  | β (95 % CI) | *P* value |  | β (95 % CI) | *P* value |  | β (95 % CI) | *P* value |
| Mean glucose (mmol/L) | | | | | | | | |
| Model 1 | -1.875 (-3.316, -0.433) | 0.011 |  | -1.794 (-3.187, -0.400) | 0.012 |  | -2.594 (-4.807, -0.381) | 0.022 |
| Model 2 | -1.956 (-3.384, -0.529) | 0.007 |  | -1.747 (-3.115, -0.379) | 0.013 |  | -2.553 (-4.780, -0.326) | 0.025 |
| Model 3 | -1.738 (-3.150, -0.327) | 0.016 |  | -1.604 (-2.983, -0.224) | 0.023 |  | -2.415 (-4.647, -0.183) | 0.034 |
| Model 4 | -3.611 (-5.891, -1.332) | 0.002 |  | -2.387 (-4.713, -0.061) | 0.044 |  | -4.212 (-8.094, -0.331) | 0.034 |
| Model 5 | -3.628 (-5.933, -1.324) | 0.002 |  | -2.407 (-4.782, -0.033) | 0.047 |  | -4.360 (-8.324, -0.397) | 0.031 |
| Median glucose (mmol/L) | | | | | | | | |
| Model 1 | -6.365 (-10.379, -2.351) | 0.002 |  | -5.862 (-9.744, -1.980) | 0.003 |  | -8.050 (-14.369, -1.731) | 0.013 |
| Model 2 | -5.845 (-9.855, -1.836) | 0.004 |  | -5.022 (-8.860, -1.183) | 0.011 |  | -7.980 (-14.363, -1.597) | 0.015 |
| Model 3 | -6.312 (-10.296, -2.328) | 0.002 |  | -4.937 (-8.839, -1.036) | 0.013 |  | -7.740 (-14.193, -1.288) | 0.019 |
| Model 4 | -7.990 (-12.575, -3.404) | <0.001 |  | -5.590 (-10.250, -0.929) | 0.019 |  | -8.053 (-15.683, -0.423) | 0.039 |
| Model 5 | -7.896 (-12.466, -3.326) | <0.001 |  | -5.915 (-10.582, -1.249) | 0.013 |  | -8.608 (-16.274, -0.943) | 0.028 |
| SD (mmol/L) | | | | | | | | |
| Model 1 | -0.380 (-0.770, 0.010) | 0.056 |  | -0.278 (-0.661, 0.104) | 0.153 |  | -0.339 (-0.966, 0.288) | 0.288 |
| Model 2 | -0.305 (-0.696, 0.087) | 0.126 |  | -0.175 (-0.555, 0.205) | 0.365 |  | -0.333 (-0.969, 0.303) | 0.304 |
| Model 3 | -0.414 (-0.805, -0.023) | 0.038 |  | -0.193 (-0.582, 0.196) | 0.330 |  | -0.328 (-0.980, 0.324) | 0.323 |
| Model 4 | -0.493 (-0.888, -0.097) | 0.015 |  | -0.267 (-0.673, 0.139) | 0.196 |  | -0.357 (-1.035, 0.321) | 0.301 |
| Model 5 | -0.507 (-0.905, -0.109) | 0.013 |  | -0.316 (-0.727, 0.095) | 0.132 |  | -0.425 (-1.115, 0.265) | 0.226 |
| CV (%) | | | | | | | | |
| Model 1 | -1.552 (-2.791, -0.312) | 0.014 |  | -1.366 (-2.574, -0.157) | 0.027 |  | -1.546 (-3.579, 0.487) | 0.135 |
| Model 2 | -1.414 (-2.652, -0.176) | 0.025 |  | -1.108 (-2.302, 0.085) | 0.069 |  | -1.489 (-3.540, 0.562) | 0.154 |
| Model 3 | -1.501 (-2.724, -0.278) | 0.016 |  | -1.044 (-2.250, 0.162) | 0.089 |  | -1.388 (-3.451, 0.674) | 0.186 |
| Model 4 | -1.674 (-3.030, -0.319) | 0.016 |  | -0.979 (-2.365, 0.408) | 0.166 |  | -0.940 (-3.298, 1.419) | 0.433 |
| Model 5 | -1.682 (-3.039, -0.324) | 0.015 |  | -1.048 (-2.445, 0.350) | 0.141 |  | -1.031 (-3.408, 1.346) | 0.393 |
| MAGE (mmol/L) | | | | | | | | |
| Model 1 | -1.665 (-3.077, -0.253) | 0.021 |  | -1.625 (-2.994, -0.257) | 0.020 |  | -2.312 (-4.477, -0.147) | 0.036 |
| Model 2 | -1.741 (-3.140, -0.342) | 0.015 |  | -1.583 (-2.926, -0.240) | 0.021 |  | -2.271 (-4.449, -0.093) | 0.041 |
| Model 3 | -1.481 (-2.866, -0.095) | 0.036 |  | -1.429 (-2.785, -0.072) | 0.039 |  | -2.108 (-4.295, 0.079) | 0.059 |
| Model 4 | -2.942 (-5.185, -0.698) | 0.010 |  | -1.931 (-4.225, 0.363) | 0.099 |  | -3.276 (-7.071, 0.519) | 0.090 |
| Model 5 | -2.986 (-5.270, -0.702) | 0.011 |  | -1.908 (-4.269, 0.454) | 0.113 |  | -3.389 (-7.293, 0.516) | 0.089 |
| TIR (%): 3.9–10.0 mmol/L | | | | | | | | |
| Model 1 | 2.502 (1.089, 3.914) | <0.001 |  | 2.475 (1.112, 3.838) | <0.001 |  | 2.474 (0.248, 4.699) | 0.030 |
| Model 2 | 2.467 (1.065, 3.869) | <0.001 |  | 2.284 (0.943, 3.624) | <0.001 |  | 2.426 (0.187, 4.666) | 0.034 |
| Model 3 | 2.273 (0.879, 3.667) | 0.001 |  | 2.140 (0.777, 3.503) | 0.002 |  | 2.187 (-0.071, 4.445) | 0.058 |
| Model 4 | 4.065 (2.153, 5.977) | <0.001 |  | 3.200 (1.239, 5.162) | 0.001 |  | 2.907 (-0.367, 6.181) | 0.082 |
| Model 5 | 4.192 (2.278, 6.107) | <0.001 |  | 3.208 (1.227, 5.189) | 0.002 |  | 2.918 (-0.411, 6.247) | 0.085 |
| TAR (%): >10.0 mmol/L | | | | | | | | |
| Model 1 | -0.243 (-0.382, -0.103) | <0.001 |  | -0.240 (-0.375, -0.106) | <0.001 |  | -0.277 (-0.495, -0.059) | 0.013 |
| Model 2 | -0.245 (-0.382, -0.107) | <0.001 |  | -0.229 (-0.361, -0.097) | <0.001 |  | -0.273 (-0.492, -0.054) | 0.015 |
| Model 3 | -0.221 (-0.358, -0.084) | 0.002 |  | -0.214 (-0.348, -0.080) | 0.002 |  | -0.255 (-0.475, -0.034) | 0.024 |
| Model 4 | -0.466 (-0.677, -0.254) | <0.001 |  | -0.375 (-0.593, -0.158) | <0.001 |  | -0.451 (-0.820, -0.082) | 0.017 |
| Model 5 | -0.460 (-0.672, -0.248) | <0.001 |  | -0.373 (-0.592, -0.154) | <0.001 |  | -0.455 (-0.827, -0.082) | 0.017 |
| TAR (%): >13.9 mmol/L | | | | | | | | |
| Model 1 | -0.276 (-0.582, 0.031) | 0.077 |  | -0.278 (-0.571, 0.014) | 0.062 |  | -0.441 (-0.891, 0.009) | 0.055 |
| Model 2 | -0.282 (-0.585, 0.021) | 0.068 |  | -0.260 (-0.547, 0.026) | 0.075 |  | -0.432 (-0.884, 0.021) | 0.061 |
| Model 3 | -0.280 (-0.578, 0.017) | 0.064 |  | -0.252 (-0.538, 0.034) | 0.084 |  | -0.431 (-0.882, 0.020) | 0.061 |
| Model 4 | -0.437 (-0.844, -0.030) | 0.035 |  | -0.305 (-0.714, 0.105) | 0.144 |  | -0.474 (-1.117, 0.169) | 0.148 |
| Model 5 | -0.482 (-0.889, -0.075) | 0.021 |  | -0.294 (-0.707, 0.120) | 0.163 |  | -0.439 (-1.091, 0.214) | 0.186 |
| TBR (%): <3.9 mmol/L | | | | | | | | |
| Model 1 | 0.003 (-0.444, 0.450) | 0.989 |  | -0.003 (-0.442, 0.436) | 0.990 |  | 0.371 (-0.310, 1.052) | 0.284 |
| Model 2 | 0.062 (-0.381, 0.506) | 0.782 |  | 0.074 (-0.359, 0.506) | 0.738 |  | 0.379 (-0.307, 1.066) | 0.277 |
| Model 3 | 0.021 (-0.415, 0.458) | 0.924 |  | 0.071 (-0.364, 0.505) | 0.749 |  | 0.438 (-0.253, 1.128) | 0.213 |
| Model 4 | -0.153 (-0.611, 0.305) | 0.511 |  | -0.091 (-0.558, 0.376) | 0.702 |  | 0.305 (-0.440, 1.049) | 0.421 |
| Model 5 | -0.238 (-0.704, 0.228) | 0.316 |  | -0.098 (-0.576, 0.379) | 0.685 |  | 0.351 (-0.421, 1.124) | 0.371 |
| TBR (%): <3.0 mmol/L | | | | | | | | |
| Model 1 | -0.905 (-2.334, 0.524) | 0.214 |  | -0.077 (-1.444, 1.290) | 0.912 |  | -0.167 (-2.356, 2.022) | 0.881 |
| Model 2 | -0.676 (-2.102, 0.751) | 0.352 |  | 0.011 (-1.339, 1.360) | 0.988 |  | -0.235 (-2.459, 1.988) | 0.835 |
| Model 3 | -0.772 (-2.181, 0.636) | 0.281 |  | 0.036 (-1.322, 1.393) | 0.959 |  | -0.022 (-2.269, 2.226) | 0.985 |
| Model 4 | -1.085 (-2.516, 0.346) | 0.137 |  | -0.298 (-1.724, 1.127) | 0.680 |  | -0.519 (-2.858, 1.820) | 0.662 |
| Model 5 | -1.217 (-2.671, 0.238) | 0.101 |  | -0.312 (-1.774, 1.149) | 0.674 |  | -0.557 (-2.978, 1.864) | 0.651 |
| HBGI | | | | | | | | |
| Model 1 | -0.813 (-1.374, -0.252) | 0.005 |  | -0.696 (-1.235, -0.158) | 0.012 |  | -0.909 (-1.754, -0.065) | 0.035 |
| Model 2 | -0.802 (-1.358, -0.246) | 0.005 |  | -0.629 (-1.159, -0.100) | 0.020 |  | -0.890 (-1.740, -0.040) | 0.040 |
| Model 3 | -0.775 (-1.323, -0.227) | 0.006 |  | -0.592 (-1.124, -0.060) | 0.029 |  | -0.849 (-1.699, 0.001) | 0.050 |
| Model 4 | -1.428 (-2.212, -0.645) | <0.001 |  | -0.807 (-1.610, -0.004) | 0.049 |  | -1.067 (-2.345, 0.212) | 0.102 |
| Model 5 | -1.472 (-2.255, -0.690) | <0.001 |  | -0.821 (-1.629, -0.013) | 0.046 |  | -1.077 (-2.370, 0.215) | 0.102 |
| LBGI | | | | | | | | |
| Model 1 | -0.050 (-1.361, 1.261) | 0.940 |  | 0.423 (-0.866, 1.711) | 0.519 |  | 0.967 (-1.070, 3.003) | 0.351 |
| Model 2 | 0.193 (-1.113, 1.499) | 0.771 |  | 0.636 (-0.637, 1.909) | 0.326 |  | 0.962 (-1.098, 3.023) | 0.358 |
| Model 3 | 0.039 (-1.258, 1.336) | 0.953 |  | 0.654 (-0.635, 1.943) | 0.319 |  | 1.238 (-0.870, 3.347) | 0.248 |
| Model 4 | -0.466 (-1.874, 0.943) | 0.516 |  | 0.223 (-1.224, 1.670) | 0.761 |  | 1.039 (-1.348, 3.427) | 0.392 |
| Model 5 | -0.671 (-2.107, 0.766) | 0.359 |  | 0.216 (-1.269, 1.701) | 0.775 |  | 1.189 (-1.291, 3.669) | 0.346 |
| MODD (mmol/L) | | | | | | | | |
| Model 1 | -5.492 (-9.463, -1.520) | 0.007 |  | -4.906 (-8.773, -1.039) | 0.013 |  | -8.813 (-15.040, -2.587) | 0.006 |
| Model 2 | -5.667 (-9.588, -1.746) | 0.005 |  | -4.930 (-8.708, -1.152) | 0.011 |  | -8.807 (-15.055, -2.558) | 0.006 |
| Model 3 | -5.556 (-9.490, -1.622) | 0.006 |  | -4.617 (-8.480, -0.754) | 0.019 |  | -8.194 (-14.564, -1.825) | 0.012 |
| Model 4 | -9.223 (-14.147, -4.299) | <0.001 |  | -5.498 (-10.551, -0.446) | 0.033 |  | -10.464 (-18.884, -2.045) | 0.015 |
| Model 5 | -9.222 (-14.181, -4.263) | <0.001 |  | -5.411 (-10.532, -0.290) | 0.038 |  | -10.767 (-19.316, -2.218) | 0.014 |
| IQR (mmol/L) | | | | | | | | |
| Model 1 | -3.705 (-6.819, -0.590) | 0.020 |  | -3.286 (-6.304, -0.267) | 0.033 |  | -7.001 (-11.831, -2.172) | 0.005 |
| Model 2 | -4.026 (-7.106, -0.946) | 0.011 |  | -3.404 (-6.357, -0.451) | 0.024 |  | -7.010 (-11.864, -2.156) | 0.005 |
| Model 3 | -4.096 (-7.175, -1.018) | 0.009 |  | -3.198 (-6.213, -0.184) | 0.038 |  | -6.607 (-11.550, -1.665) | 0.009 |
| Model 4 | -7.124 (-11.057, -3.190) | <0.001 |  | -3.696 (-7.721, 0.329) | 0.072 |  | -8.840 (-15.549, -2.131) | 0.010 |
| Model 5 | -7.135 (-11.066, -3.203) | <0.001 |  | -3.768 (-7.814, 0.279) | 0.068 |  | -9.252 (-16.027, -2.476) | 0.008 |

Univariable and multivariable linear regression analysis. Model 1 was not adjusted. Model 2 was adjusted for age and sex. Model 3 was adjusted for BMI, duration of diabetes, and the covariates in Model 2. Model 4 was adjusted for smoking status, HbA1c, systolic blood pressure, total cholesterol, HDL cholesterol, log-transformed triglycerides, uric acid, and the covariates in Model 3. Model 5 was adjusted for eGFR, log-transformed urine albumin-to-creatinine ratio, and the covariates in Model 4.

GSM, gray-scale median; SD, standard deviation; CV, coefficient of variation; MAGE, mean amplitude of glycemic excursion; TIR, time in range; TAR, time above range; TBR, time below range; HBGI, high blood glucose index; LBGI, low blood glucose index; MODD, mean of daily differences; IQR, interquartile range

**Table S30 List of sites and investigators**

| Site | Investigator |
| --- | --- |
| Ashiya Central Hospital | Koji Matsushita, Manabu Narisawa |
| Aso Clinic | Katsumi Aso, Yuko Ando, Fumihiko Sato |
| Hagiwara Central Hospital | Emiko Morita, Keiichi Torimoto |
| Hayashi Clinic | Isao Hayashi |
| Inokuchi Clinic | Nobuo Inokuchi |
| Japan Community Health Care Organization Osaka Hospital | Masahiro Hatazaki, Arichika Deguchi, Azusa Shiraki |
| Juntendo Tokyo Koto Geriatric Medical Center (Department of Medicine, Diabetology and Endocrinology) | Ayako Kitamura, Eri Tanabe, Hidenori Yoshii, Tomio Onuma, Tomo Nakajima |
| Juntendo University Graduate School of Medicine (Department of Metabolism & Endocrinology) | Eisuke Yasunari, Hideyoshi Kaga, Hiroaki Sato, Hirotaka Watada, Kagemi Takeno, Luka Suzuki, Miwa Himuro, Syuhei Aoyama, Takashi Funayama, Takehiro Katahira, Takeshi Miyatsuka, Tomoya Mita, Yuya Nishida |
| Juntendo University Nerima Hospital (Department of Medicine, Diabetes and Endocrinology) | Koji Komiya |
| Kanda Naika Clinic | Satoshi Kawashima |
| Kansai Rosai Hospital (Diabetes and Endocrinology) | Tsunehiko Yamamoto |
| Kihara Diabetes Clinic | Yasuyuki Kihara |
| Kosugi Medical Clinic | Keisuke Kosugi |
| Kawasaki Hospital (Department of Internal Medicine) | Akihito Otsuka, Jun Murai |
| Matsuoka Medical Clinic | Hirofumi Matsuoka |
| Misaki Naika Clinic | Nobuichi Kuribayashi |
| Japan Labour Health and Safety Organization Kyushu Rosai Hospital, Moji Medical Center (Department of Internal Medicine) | Tadashi Arao, Kei Sugai |
| Musashino Family Clinic | Yuichi Kojima |
| Nakakinen Clinic | Maiko Nakata, Miyoko Saito, Takeshi Osonoi, Yusuke Osonoi |
| Nakama Municipal hospital | Kohei Uriu, Yosifumi Inada, Kanako Suzuka, Ichiro Takagi |
| National Hospital Organization Osaka National Hospital (Diabetes Center) | Ken Kato |
| Nishida Keiko Diabetes Clinic | Keiko Nishida, Akira Kurozumi, Fumi Uemura, Keiichi Torimoto, Maiko Hajime, Manabu Narisawa, Satomi Sonoda, Kumiko Tidiwa |
| Osaka General Medical Center (Department of Diabetes and Endocrinology) | Youhei Fujita, Sayoko Shimizu, Masahisa Hata, Yutaka Umayahara |
| Osaka Police Hospital (Department of Endocrinology and Metabolism) | Tetsuyuki Yasuda |
| Osaka Rosai Hospital | Ryomoto Kayoko |
| Osaka University Graduate School of Medicine (Department of Metabolic Medicine) | Iichiro Shimomura, Naoto Katakami, Takaaki Matasuoka, Mitsuyoshi Takahara, Kazuyuki Miyashita, Hiroyo Ninomiya, Naohiro Taya |
| Sasaki Hospital | Shinichiro Mine, Kenji Koikawa |
| School of Medicine, University of Occupational and Environmental Health, Japan (First Department of Internal Medicine) | Yosuke Okada, Akira Kurozumi, Manabu Narisawa, Maiko Hajime, Fumi Uemura, Satomi Sonoda, Kenichi Tanaka, Takashi Otsuka |
| Secomedic Hospital | Satomi Wakasugi, Tomoya Mita |
| Shiraiwa Medical Clinic | Toshihiko Shiraiwa |
| Taneda Clinic | Yoshinobu Taneda |
| Takaishi Naika Ichoka Clinic | Tomoya Mita |
| Tobata General Hospital (Department of Internal Medicine) | Kazuko Kanda |
| Wakamatsu Hospital of the University of Occupational and Environmental Health | Torimoto Keiichi, Nishio Kousuke |

Sites and investigators are listed in alphabetical order.
